# Comparative two-dimensional polyacrylamide gel electrophoresis of the salivary proteome of children with autism spectrum disorder

**DOI:** 10.1111/jcmm.12658

**Published:** 2015-08-20

**Authors:** Armand G Ngounou Wetie, Kelly L Wormwood, Laci Charette, Jeanne P Ryan, Alisa G Woods, Costel C Darie

**Affiliations:** a Biochemistry & Proteomics Group, Department of Chemistry & Biomolecular Science, Clarkson UniversityPotsdam, NY, USA; b SUNY Plattsburgh Neuropsychology Clinic and Psychoeducation ServicesPlattsburgh, NY, USA; c Department of Psychology, SUNY PlattsburghPlattsburgh, NY, USA

**Keywords:** autism spectrum disorder, 2D-PAGE, nanoLC-MS/MS, early diagnosis

## Abstract

In the last decades, prevalence of autism spectrum disorder (ASD) has been on the rise. However, clear aetiology is still elusive and improvements in early diagnosis are needed. To uncover possible biomarkers present in ASD, we used two-dimensional polyacrylamide gel electrophoresis and nanoliquid chromatography-tandem mass spectrometry (nanoLC-MS/MS), to compare salivary proteome profiling of children with ASD and controls. A total of 889 spots were compared and only those spots with a fold change ≥1.7 and a *P*-value <0.05 or a fold change of ≥3.0 between ASD cases and controls were analysed by nanoLC-MS/MS. Alpha-amylase, CREB-binding protein, p532, Transferrin, Zn alpha2 glycoprotein, Zymogen granule protein 16, cystatin D and plasminogen were down-regulated in ASD. Increased expression of proto-oncogene Frequently rearranged in advanced T-cell lymphomas 1 (FRAT1), Kinesin family member 14, Integrin alpha6 subunit, growth hormone regulated TBC protein 1, parotid secretory protein, Prolactin-inducible protein precursor, Mucin-16, Ca binding protein migration inhibitory factor-related protein 14 (MRP14) was observed in individuals with ASD. Many of the identified proteins have previously been linked to ASD or were proposed as risk factors of ASD at the genetic level. Some others are involved in pathological pathways implicated in ASD causality such as oxidative stress, lipid and cholesterol metabolism, immune system disturbances and inflammation. These data could contribute to protein signatures for ASD presence, risk and subtypes, and advance understanding of ASD cause as well as provide novel treatment targets for ASD.

## Introduction

Autism spectrum disorders (ASDs) are a group of neurodevelopmental heterogeneous disorders 1 characterized by impaired social interaction, impaired verbal and non-verbal communication and stereotyped and rigid patterns of behaviour and interest 2. The classification of ASD under the Diagnostic Statistical Manual Version IV Text Revision (DSM-IV-TR) included autistic disorder, Asperger syndrome and pervasive developmental disorder-not otherwise specified (PDD-NOS), whereas the new revision (DSM-5) does not differentiate between these subtypes anymore and groups communication and social deficits into one symptom class 2. It is estimated that 1/68 children in the United States (1 in 42 boys) is diagnosed with ASD with boys 3–4 times more affected than girls 3. It has been observed that the prevalence of ASD is on the rise with possible explanations suggested such as broader diagnostic criteria 4,5, improved awareness/screening 6, environmental pollution/pesticides 7, *in utero* risk factors 8 and paternal/maternal age 9,10. The heterogeneous nature of ASD is also reflected in the range of symptoms developed by people with ASD. While some individuals with ASD have only mild symptoms, others present with severe symptoms. Several conditions are frequently comorbid with ASD, including intellectual disability, epilepsy 11 and gastrointestinal problems 12. Most people with ASD have motor abnormalities (*e.g*. hypotonia, poor motor planning, poor coordination and toe walking) 13 and increased rates of somatic problems (*e.g*. sensory integration issues, overweight and immunological problems) 13–16. Additionally; by contrast with age- and sex-matched controls, ASD patients show above-average mortality risks associated with comorbid medical conditions and intellectual disability 17. However, about 5% of people with ASD display exceptional aptitudes (memorization) and increased perception and attention in comparison with the general population 18.

Presently, risperidone and aripiprazole (antipsychotics) are the only two FDA-approved medications for ASD. However, these drugs only address irritability in individuals with ASD, which is not a core symptom of ASD 19,20. Behaviorial treatments are used for core ASD symptoms, to improve social skills, communication and to reduce non-productive repetitive behaviours 5,21. Studies have shown that early intensive behaviorial intervention has a better outcome when initiated during toddlerhood or preschool age and continued for 2–3 years 22. Treatments at all ages can help with ASD symptoms 5. The exact aetiology of ASD is unknown. Although many studies have pointed to a high heritability in ASD (about 80%), genetic causes or genomic risk factors of autism are still yet to be identified in people with ASD 18. Further, several current studies have proposed that the dysregulation of certain physiological and metabolic processes (redox, mitochondrial and cholesterol metabolisms) may play a role in ASD pathophysiology 23–25. Therefore, genetics alone will not be able to fully provide answers that are needed with regard to the aetiology, diagnosis and treatment of ASD. Recently, there has been an intense effort to search for biological markers that will help in early diagnosis, prognosis and treatment response 26. Proteomics, and in particular, mass spectrometry is the method of choice for biomarker discovery in human biofluids for many indications 27–32. Our group has used Tricine-PAGE and nanoLC-MS/MS for the identification of putative biomarkers of ASD in serum of children with ASD compared with their matched controls. We reported increased levels of apolipoproteins (Apos) ApoA1 and ApoA4 and of serum paraoxanase/arylesterase 1 supporting current theories that ASDs may involve dysregulated cholesterol metabolism and oxidative stress 33. Presently, we are probing different proteomics strategies for investigating biomarkers of ASD not only in serum, but also in saliva. Using nano liquid chromatography-tandem mass spectrometry (nanoLC-MS/MS), we found statistically significant differences in several salivary proteins, including elevated prolactin-inducible protein, lactotransferrin, Ig kappa chain C region, Ig gamma-1 chain C region, Ig lambda-2 chain C regions, neutrophil elastase, polymeric immunoglobulin receptor and deleted in malignant brain tumours 1. We further found decrease in ASD saliva in the proteins statherin, histatin, and acidic proline rich peptide relative to typically developing controls 34. These results need to be confirmed in larger case numbers and complemented using additional protein-discovery techniques.

Therefore, in this study, we used a two-dimensional (2D)-PAGE approach coupled with nanoLC-MS/MS to further explore the salivary proteome of children with ASD at the search for potential, reliable and robust markers that could potentially be used for diagnosis. 2D-PAGE is particularly useful in identifying proteins and protein isoforms that may not necessarily be identified by other methods such as 1D-PAGE and nanoLC-MS/MS or in-solution digestion and nanoLC-MS/MS. Therefore, 2D-PAGE is not only a different way of investigating the proteins for biomarker discovery, especially helpful in identifying protein isoforms, truncated proteins or post-translationally modified proteins, but it is also complementary to other approaches used in our previous experiments 35–37. 2D-PAGE offers many advantages as a mass spectrometric screening method including high-throughput, broad dynamic range, adequate sensitivity and good reproducibility. Although 2D-PAGE coupled with mass spectrometry is a common proteomic approach for the screening of putative biomarkers in several conditions such as cancer, autoimmune and neurodevelopmental disorders 38–40, only very few studies have used this approach in ASD biomarker research 41–45. However; of these few studies most utilized MALDI-MS/MS and none of them investigated the salivary proteome using 2D-PAGE coupled with LC-MS/MS.

In this study, we performed a comparative analysis of the salivary proteome of children with ASD and matched typically developing control participants using two dimensional gel electrophoresis (2D-GE) and nanoLC-ESI-MS/MS. We found several proteins that were differentially expressed in the saliva of people with ASD related to typically developing cases, some of which were identified in our prior study 34 and others that were novel. This innovative approach of screening potential biomarkers of ASD led to novel identification of proteins that could reveal different protein signatures indicating ASD risk, diagnosis and subtype. Bioinformatics analyses using Database for Annotation, Visualization, and Integrated Discovery (DAVID) 46, Protein ANalysis THrough Evolutionary Relationships (PANTHER) 47 and Search Tool for the Retrieval of Interacting genes (STRING) 48 databases allowed for the functional classification of the detected proteins and highlighted ASD-relevant biological pathways.

## Materials and methods

### Ethics statement

This study was carried out in accordance with the declaration of Helsinki and was approved and reviewed by the Institutional Review Board of the State University of New York Neuropsychology clinic where the samples were collected. All participants or caregivers provided written informed consent.

### Sample collection

About 1–2 ml saliva were obtained from ASD patients diagnosed based on the DSM-IV-TR *via* passive drool into a straw and collection cup. Control participants were healthy individuals with no known history of any diagnosis with ASD or other neurodevelopmental disorder. A detailed description of the participants of this study has been summarized in Table1. Upon collection, samples were centrifuged for 10 min. at 14,000 rpm. in a bench centrifuge (for removal of cell debris) and the resulting supernatant was frozen at −20°C until use.

**Table 1:** Participant demographics

Participant no.	Diagnosis	Gender	Age	Language use	Comorbidities	Medication
A1	Autism	M	12	Verbal, mild to moderate	ADHD, anxiety	Strattera, citalopram
A2	Autism	M	16	Severe delays in functioning, language	ADHD, behaviorial disturbances	Risperidone, Concerta, sertraline
A3	Autism	M	8	Verbal, mild to moderate	Allergies	Claritin, multi-vitamin
A4	PDD-NOS	M	13	Verbal, mild to moderate	Epilepsy	Lamictal
A6	Autism, possible Asperger's	M	10	High functioning, verbal	None reported	None
A7	Autism	M	11	Verbal, mild to moderate	None reported	Multi-vitamin
B1	None	M	9	N/A	None	None
B2	None	M	6	N/A	None	None
B3	None	M	13	N/A	None	None
B4	None	M	10	N/A	None	None
B5	None	M	11	N/A	None	None
B6	None	M	8	N/A	None	None

A5 was removed because of an insufficient sample.

### Two-dimensional PAGE

First, equal amounts of protein from the saliva samples from six individual donors were pooled altogether in one sample for each ASD participant and controls.

Two-dimensional polyacrylamide gel electrophoresis was performed with the carrier ampholine method of isoelectric focusing 49,50. The samples were diluted with 250 μl of SDS boiling buffer without reducing agents (BB) and dialysed overnight against 5 mM Tris pH 6.8 using 6–8000 MWCO membranes at 4°C. The samples were lyophilized, dissolved in 100 μl of BB and 300 μl water and the protein concentrations determined using the BCA assay (Pierce Chemical Co., Rockford, IL, USA) 51. Samples were lyophilized again and dissolved to 0.67 mg/ml and 3.33 mg/ml in 1:1 diluted BB:urea sample buffer (with reducing agents) before loading. Isoelectric focusing was carried out in a glass tube of inner diameter 3.3 mm using 2% pH 3–10 Isodalt Servalytes (Serva, Heidelberg, Germany) for 20,000 volt-hrs. One hundred nanograms of an IEF internal standard, tropomyosin, was added to the sample. This protein migrates as a doublet with lower polypeptide spot of MW 33,000 and pI 5.2. After equilibration for 10 min. in Buffer ‘O’ (10% glycerol, 50 mM dithiothreitol, 2.3% SDS and 0.0625 M Tris, pH 6.8), each tube gel was sealed to the top of a stacking gel that overlaid a 10% acrylamide slab gel (1.00 mm thick). SDS slab gel electrophoresis was carried out for about 5 hrs at 25 mA/gel. The following proteins (Sigma Chemical Co., St. Louis, MO, USA and EMD Millipore, Billerica, MA, USA) were used as molecular weight standards: myosin (220,000), phosphorylase A (94,000), catalase (60,000), actin (43,000), carbonic anhydrase (29,000) and lysozyme (14,000). These standards appear along the basic edge of the silver-stained 10% acrylamide slab gel. The silver-stained gels were dried between sheets of cellophane with the acid edge to the left 52.

### Computerized comparisons

Duplicate gels were obtained from each sample to reduce sources of variability and to detect differences with real statistical significance. The gels were scanned with a laser densitometer (Model PDSI; Molecular Dynamics Inc, Sunnyvale, CA, USA). The scanner was checked for linearity prior to scanning with a calibrated Neutral Density Filter Set (Melles Griot, Irvine, CA, USA). The images were analysed using Progenesis Same Spots software (version 4.5, 2011; Nonlinear Dynamics, Durham, NC, USA) and Progenesis PG240 software (version 2006; Nonlinear Dynamics). The general method of computerized analysis for these pairs included image warping followed by spot finding, background subtraction (average on boundary), matching, and quantification in conjunction with detailed manual checking. A *P*-value (Student's *t*-test, *n* = 2 gels/sample) is calculated to help assess whether corresponding spots are different. As background is a factor, spot differences are checked by eye. Spot % is equal to spot integrated density above background (volume) expressed as a percentage of total density above background of all spots measured. Difference is defined as fold-change of spot percentages. For example, if corresponding protein spots from different samples (*e.g*. ASD *versus* controls) have the same spot %, the difference field will show 1.0; if the spot % from ASD is twice as large as controls, the difference field will display 2.0 indicating twofold up-regulation. If the spot % from ASD has a value half as large, the difference field will display – 2.0 indicating a twofold down-regulation.

### Spot picking and in-gel digestion

Protein spots of interest were selected based on a fold increase or decrease of ≥1.7 and *P*-value <0.05 or a fold increase or decrease of ≥3.0. Picked spots were excised and subjected to in-gel tryptic digestion and peptide extraction for protein identification by nanoLC-MS/MS analysis, as described before 33,35. Briefly, spots were washed in HPLC grade water, 50 mM ammonium bicarbonate (ABC), 50% acetonitrile (ACN)/50% ABC for each 15 min. under moderate shaking at room temperature (RT). Next, the gel pieces were dehydrated with 100% ACN and dried under speed vac. Reduction and alkylation were carried out with 10 mM dithiothreitol (DTT) in 25 mM ABC for 30 min. at 60°C and with 100 mM iodoacetamide in 25 mM ABC for 45 min. in the dark respectively. Gel pieces were then dehydrated again, dried and rehydrated in 20 μl of a trypsin solution (10 ng/μl) overnight at 37°C. After incubation, peptide extraction was carried out with 5% formic acid (FA)/50 mM ABC/50% ACN and with 5% FA/100% ACN (20 min. each). Extracted peptides were dried and submitted to a zip tip step for purification (EMD Millipore).

### Mass spectrometry and data analysis

Extracted peptides were analysed using a NanoAcquity UPLC (Waters Corp., Milford, MA, USA) coupled to a Q-TOF API MS (Waters/Micromass, Milford, MA, USA). Chromatographic separation of peptides was performed on a C18 1.7 μm, 150 μm × 100 mm reversed phase column (Waters Corp.) and eluted over a 60 min. gradient of 2–100% ACN in 0.1% FA at a flow rate of 400 nl/min. MS/MS spectra were obtained in a data-dependent acquisition mode consisted of survey MS scans of the five most intense peaks and automatic data-dependent MS/MS of 2+, 3+ and 4+ ions. The MS/MS was triggered when the MS signal intensity exceeded 13 counts per second and lasted until the total MS/MS ion counts reached 999,999 or for up to 6.3 sec. The full MS scan covered the m/z range from 350 to 1800. Calibration of the mass spectrometer was performed for both precursor and product ions using 100 fmol GluFib standard peptide (Glu1-Fibrinopeptide B) with the amino acid sequence EGVNDNEEGFFSAR and a calculated mass for the monoisotopic m/z peak of 1570.68. The precursor ion monitored had an m/z of 785.84 (2+).

The raw data were processed using ProteinLynx Global Server (PLGS, version 2.4, Waters Corporation, Milford, MA, USA) software with the following parameters: background subtraction of polynomial order 5 adaptive with a threshold of 35%, two smoothings with a window of three channels in Savitzky–Golay mode, and centroid calculation of the top 80% of peaks based on a minimum peak width of 4 channels at half-height. The MASCOT search engine (www.matrixscience.com) was employed for database searches. Search parameters were: propionamide as fixed modification for cysteine, methionine oxidation as variable modification, and precursor and product/fragment mass tolerance were set to 1.3 Da and 0.8 Da respectively; NCBInr human database, one missed cleavage.

### Bioinformatics analysis

A series of analyses was undertaken with the differentially expressed proteins identified by 2D-PAGE. Interaction analysis was performed with STRING (http://string-db.org/newstring_cgi/show_input_page.pl) 48. Further, PANTHER v9.0 and DAVID v6.7 (Database for Annotation, Visualization and Integrated Discovery) were employed for functi-onal annotation analysis to understand the biological significance (*e.g*. physiological pathways) associated with large lists of genes or proteins 53.

## Results and discussion

### 2D-PAGE of pooled samples ASD *versus* pooled samples controls

**Figure 1:**
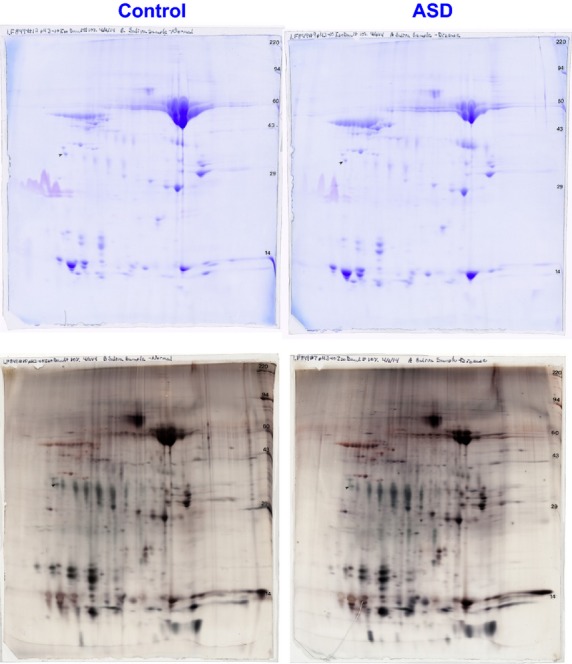
Image of coomassie- and silver-stained 2D-PAGE gels of control and autism spectrum disorder (ASD) saliva samples.

**Figure 2:**
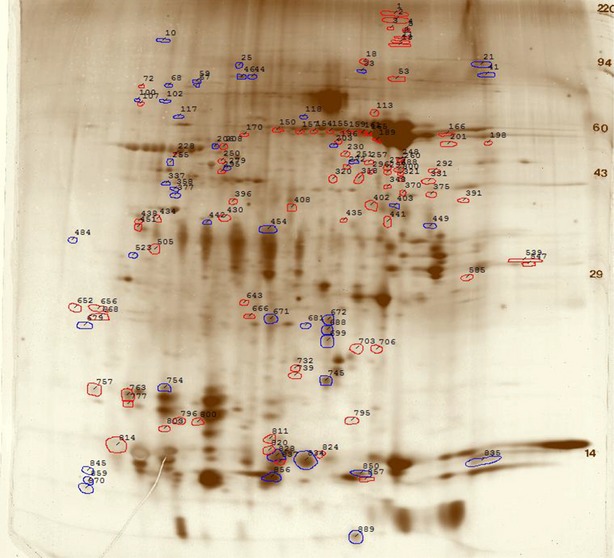
2D Gel difference image of averaged autism spectrum disorder (ASD) *versus* typically developing. Polypeptide spots increased in ASD *versus* control are outlined in blue, while spots decreased in ASD *versus* control are outlined in red. See Table S1 for spot data and measurements.

Pooled samples provide a rapid screening method for evaluating the possibility of finding markers in a specific biological fluid; an approach generally employed in cancer biomarker research 54–56. This approach can be used in ASD, although with the caveat that ASD is a known heterogeneous disorder 57, and therefore, ultimately individual comparisons are needed. Pooled ASD samples from 6 individuals with ASD and pooled control samples from 6 age-matched typically developing cases were generated for identifying putative salivary biomarkers in ASD using 2D-PAGE combined with nanoLC-LC-MS/MS. In all, 2 Coomassie and 4 silver-stained (Fig.1) gels of 2D-PAGE of the saliva samples (250 μg protein in each gel) were run for reasons of reproducibility. A total of 889 spots were compared (Fig.2) and only those spots with a fold change ≥1.7 and a *P*-value <0.05 or a fold-change of ≥3.0 between ASD patients and controls were picked for in-gel tryptic digestion. We do not know, however, whether there are any unique spots specific to ASD or controls, simply because we first calculated the ratio between the spots on ASD and controls and then tested the ratio for statistical significance.

A list of all the picked spots with their pI, MW, spot percentage, fold change and p-value is found in Table S1. NanoLC-MS/MS analysis of the differentially expressed spots led to identification of several proteins that are up- or down-regulated in the saliva of ASD participants (Table2). Table2 contains proteins that were identified by submitting the pkl files generated from the MS raw data to the MASCOT database as well as proteins that were identified by *de novo* sequencing. By using *de novo* sequencing, we were able to expand the number of proteins identified in our experiments and thereby increasing the pool of theoretically available biological markers (Fig. S1).

**Table 2:** Summary of proteins identified by LC-MS/MS from the picked 2D gel spots which are differentially expressed between participants with ASD and controls. Positive fold changes represent an up-regulation while negative values signify a down-regulation of protein expression in ASD *versus* controls

Spot no.	Protein name	NCBInr accession	Protein MW (kD)	Protein score	Fold-change (ASD *versus* control)	*P*-value
59	Proto-oncogene FRAT1	gi¦31317236	29,093	26	8.3	0.004
118	Ig alpha-1 chain C region	gi¦113584	38,486	64	2.8	0.033
	Immunoglobulin heavy chain constant region alpha-2 subunit	gi¦3819788	24,238	60		
206	V-type proton ATPase subunit C 1	gi¦4502315	43,942	40	3.9	0.04
255	V-type proton ATPase subunit C 1	gi¦4502315	43,942	42	3.4	0.029
403	Carbonic anhydrase VI nirs variant 3	gi¦58737051	28,737	161	2.4	0.005
	Kinesin family member 14	gi¦109730619	186,492	21		
	Integrin alpha 6 subunit	gi¦33942	126,606	21		
449	Carbonic anhydrase isozyme VI	gi¦179732	35,469	94	1.7	0.048
699	GRTP1 protein	gi¦34783442	38,554	22	2.1	0.037
671	Parotid secretory protein	gi¦16755850	27,265	56	2.3	0.013
672	Prolactin-inducible protein precursor	gi¦4505821	16,572	39	2.5	0.005
745	Parotid secretory protein	gi¦16755850	27,265	72	1.8	0.000
754	Mucin-16	gi¦74716283	2,353,428	23	2.3	0.021
834	Ca binding protein MRP14	gi¦225793	13,291	75	1.7	0.017
845	Ca binding protein MRP14	gi¦225793	13,291	109	4.1	0.001
1	Alpha-amylase	gi¦178585	58,398	212	−3.9	0.195
	Spectrin, beta, non-erythrocytic 5	gi¦119612929	416,750	40		
2	Alpha-amylase	gi¦178585	58,398	584	−4.9	0.05
3	Alpha-amylase	gi¦178585	58,398	68	−3.5	0.255
4	Alpha-amylase	gi¦178585	58,398	246	−8.1	0.054
8	Alpha-amylase	gi¦178585	58,398	128	−5.8	0.000
11	Alpha-amylase	gi¦178585	58,398	198	−4.0	0.019
	CBP	gi¦33150676	265,351	28		
	p532	gi¦1477565	40,766	12		
13	Alpha-amylase	gi¦178585	58,398	277	−4.0	0.001
53	Transferrin variant	gi¦62897069	77,080	51	−2.0	0.046
113	Alpha-amylase	gi¦178585	58,398	487	−3.6	0.106
	Chain A, Human Pancreatic Alpha-Amylase In Complex With Myricetin	gi¦409974028	56,462	348		
	Ig alpha-1 chain C region	gi¦113584	38,486	102		
	Protein Tro alpha1 H,myeloma	gi¦223069	52,010	102		
	Ig Aalpha1 Bur	gi¦223099	51,551	80		
	Ig A1 Bur	gi¦229585	74,642	78		
150	Amylase, alpha 2A; pancreatic precursor variant	gi¦62898658	58,368	65	−3.4	0.057
154	AMY1A protein	gi¦47124258	56,859	223	−14.0	0.332
155	Alpha-amylase	gi¦178585	58,398	121	−7.5	0.474
157	AMY1A protein	gi¦47124258		23	−4.5	0.418
159	Alpha-amylase	gi¦178585	58,398	82	−5.9	0.037
	Protein-L-isoaspartate O-methyltransferase domain-containing protein 1 isoform 3	gi¦557948029	24,636	23		
161	Alpha-amylase	gi¦178585	58,398	418	−2.7	0.002
	Chain A, Human Pancreatic Alpha-Amylase In Complex With Myricetin	gi¦409974028	56,462	284		
165	Alpha-amylase	gi¦178585	58,398	1083	−2.0	0.000
	Chain A, Human Pancreatic Alpha-Amylase In Complex With Myricetin	gi¦409974028	56,462	867		
189	Alpha-amylase	gi¦178585	58,398	850	−2.3	0.012
	Chain X, Structural Studies Of Phe256trp Of Human Salivary Alpha- Amylase	gi¦47168614	56,523	828		
	Chain A, Human Pancreatic Alpha-Amylase In Complex With Myricetin	gi¦409974028	56,462	705		
	Chain A, Structure Of Human Pancreatic Alpha-Amylase In Complex With The Carbohydrate Inhibitor Acarbose	gi¦7245760	56,479	701		
208	V-type proton ATPase subunit C 1	gi¦4502315	43,942	47	−4.1	0.005
250	AMY1A protein	gi¦47124258	56,859	101	−4.4	0.036
257	Alpha-amylase	gi¦178585	58,398	224	−18.2	0.074
277	Alpha-amylase	gi¦178585	58,398	470	−3.5	0.079
292	Alpha-amylase	gi¦178585	58,398	112	−7.9	0.033
318	Alpha-amylase	gi¦178585	58,398	114	−3.1	0.185
370	Carbonic anhydrase isozyme VI	gi¦179732	35,469	59	−4.2	0.125
	Alpha-amylase	gi¦178585	58,398	57		
	Squamous cell carcinoma antigen 1	gi¦25005272	44,565	18		
402	Carbonic anhydrase isozyme VI	gi¦179732	35,469	146	−1.8	0.049
408	Zn alpha2 glycoprotein	gi¦228099	34,942	77	−2.4	0.049
	Glutamate-rich protein 6B	gi¦210147567	75,255	20		
	Immunoglobulin heavy chain variable region	gi¦112702600	12,582	14		
539	Alpha-amylase	gi¦178585	58,398	164	−14.1	0.292
547	Alpha-amylase	gi¦178585	58,398	242	−5.5	0.071
643	ALB protein	gi¦23241675	45,160	16	−2.1	0.028
656	Ig J-chain	gi¦532598	160,41	98	−2.1	0.017
666	Sperm activating protein subunit I- apolipoprotein A1-SPAP- subunit I	gi¦235865	–	24	−2.2	0.019
	Zymogen granule protein 16 homologue B precursor	gi¦94536866	22,739	22		
668	Ig J-chain	gi¦532598	16,041	63	−8.4	0.001
732	Alpha-amylase	gi¦178585	58,398	106	−2.0	0.009
739	Alpha-amylase	gi¦178585	58,398	82	−2.3	0.014
777	Prolactin-inducible protein precursor	gi¦4505821	16,572	45	−4.2	0.197
795	Alpha-amylase	gi¦178585	58,398	110	−1.8	0.016
796	Putative lipocalin 1-like protein 1	gi¦74746821	18,078	69	−1.8	0.028
800	Putative lipocalin 1-like protein 1	gi¦74746821	18,078	74	−2.1	0.013
814	Cystatin SA-III=potential precursor of acquired enamel pellicle	gi¦235948	14,409	79	−3.1	0.406
820	Cystatin D	gi¦398711	16,351	53	−2.0	0.035
	Plasminogen	gi¦38051823	90,569	14		
837	Alpha-amylase	gi¦178585	58,398	67	−1.9	0.01

### Down-regulated proteins in ASD patients

Several proteins were found to be decreased in ASD compared to controls. Only proteins that were found in spots with a *P*-value <0.05 and a fold change >1.7 were considered significant. This stringent criterion allows for true changes to be considered and for artefacts to be ignored. The following proteins were significantly reduced in ASD: alpha-amylase, CREB-binding protein (CBP), p532, Transferrin variant, Protein-l-isoaspartate O-methyltransferase domain-containing protein 1 isoform 3, Chain A of Human Pancreatic Alpha-Amylase In Complex With Myricetin, V-type proton ATPase subunit C 1, Ig J-chain, Zn alpha2 glycoprotein (ZAG), Glutamate-rich protein 6B, Immunoglobulin heavy chain variable region, Albumin (ALB) protein, Sperm activating protein subunit I-Apo A1-SPAP-subunit I, Zymogen granule protein 16 homologue B precursor, Putative lipocalin 1-like protein 1, cystatin D and plasminogen. Some of these proteins could be potential novel biomarkers. In the following, we will highlight only those proteins that are ASD-relevant and are potential ASD biomarkers.

Alpha-amylase was overwhelmingly present in the controls and necessitates therefore some attention. Amylase, a putative correlate of norepinephrine, is an enzyme secreted from the parotid salivary gland that plays a role in the digestion of starch in oral cavity 58. It increases with stress and follows a diurnal profile characterized by a decrease shortly after awakening and a progressive increase during the day 59. These results correspond with a recent study, in which lower levels of salivary alpha-amylase was found in children with ASD compared to typical and clinical age-matched controls and these levels correlated with larger tonic pupil size 60. This study also observed that children with ASD do not follow the diurnal profile seen in typically developing individuals. Additionally, salivary alpha-amylase has been advanced before as a potential early and non-invasive biomarker of ASD 61. We also note that in our previous study, other salivary proteins were decreased in ASD, including statherin, histatin and acidic proline rich peptide 34.

The CBP, a major neural activity-dependent transcriptional co-activator with intrinsic histone acetylase activity involved in various signal transduction pathways, is another protein which mRNA level was found to be reduced by 77% in ASD patients relative to controls in a recent study 62. Further, mutations and deletions of the CBP gene (CREBBP) lead to cognitive impairment, autistic features and seizures 63,64. Patients with these mutations or deletions present a mild behaviorial phenotype comprising ASD, speech deficits and moderate mental retardation 65,66.

p532 protein, also known as HECT And RLD Domain Containing E3 Ubiquitin Protein Ligase Family Member 1 (HERC1) is a guanine nucleotide exchange factor that also possesses E3 ubiquitin-protein ligase function and is involved in membrane trafficking 67. p532 is a tuberous sclerosis complex 2 (TSC2)-interacting protein and this interaction is inhibited by TSC1. Tuberous sclerosis complex was the first identified cause of autism and is a leading cause of syndromic autism with a prevalence of 26–61% of autism in TSC 68–70. Interestingly, mutations of TSC2 or TSC1 can lead to complications such as renal failure, seizures, mental retardation and autism 71,72. Furthermore, missense mutation in HERC2 was found to lead to global developmental delay and ASD 73.

Transferrin is an iron-binding major antioxidant protein that transports iron to proliferating cells and also acts as a growth factor 74. A study by Chauhan and colleagues found decreased transferrin serum levels in autism in a comparative study of 19 children with autism and their typically developing siblings 75. Notably, our prior analysis of salivary proteins in ASD identified lactotransferrin as a protein that is significantly increased in ASD, in the same group of participants as analysed here 34. Lactotransferrin also transport iron, and elevated levels of this protein may therefore act to compensate for the deficits in transferrin observed.

Zn alpha2 glycoprotein is a protein associated with lipid mobilization, a biological process regulated by FASN and other metabolic pathways such as mTOR signalling. As cholesterol and lipid metabolism is involved in ASD pathophysiology 76, ZAG could play a role in this disorder. Our prior study analysing blood serum supported the possibility that disturbances in lipid transport, specifically elevations of Apos, may be present in ASD 77.

Zymogen granule protein 16 homologue B precursor (ZG16B) is a secretory protein involved in extracellular carbohydrate binding that has been identified as a recurrent copy number variant in ASD in at least 10 reports so far 78.

Cystatin D belongs to the family of cystatin proteins, which are cysteine protease inhibitors (endosomal/lysosomal) of both host and microbial origin. Therefore, these proteins protect the oral cavity from harmful proteolysis 79. Cystatin D was identified among the genes selectively dysregulated in autism in a study comparing mRNA expression profile in lymphoblastoid cells from males with autism because of a fragile X mutation (FMR1-FM) or a 15q11-q13 duplication related to non-autistic controls. It is known that about 15–33% of people with Fragile X syndrome develop ASD 80.

Plasminogen has been indirectly associated with autism at the genetic level by investigating the contribution to ASD risk of urokinase plasminogen activator receptor which is a cofactor for plasminogen activation by urokinase plasminogen activator 81.

As for Protein-l-isoaspartate O-methyltransferase domain-containing protein 1 isoform 3, V-type proton ATPase subunit C 1, Ig J-chain, Glutamate-rich protein 6B, Immunoglobulin heavy chain variable region, ALB protein, Sperm activating protein subunit I-Apo A1-SPAP-subunit I, Putative lipocalin 1-like protein 1; it is not quite clear how they relate to ASD; however, future studies utilizing addition cases with ASD may confirm whether these proteins are consistently down-regulated in ASD.

### Proteins up-regulated in ASD cases

The same selection criterion was applied for the selection of proteins found to be increased in ASD relative to controls. The following proteins were identified: proto-oncogene FRAT1, Ig alpha-1 chain C region, immunoglobulin heavy chain constant region alpha-2 subunit, V-type proton ATPase subunit C 1, Kinesin family member 14 (KIF14), Integrin alpha 6 subunit, growth hormone regulated TBC protein 1 (GRTP1 protein), parotid secretory protein, Prolactin-inducible protein precursor, Mucin-16, Ca binding protein MRP14. Similarly, we will first highlight candidates that have already been associated with ASD in the literature and then will discuss the potential novel candidates.

The proto-oncogene FRAT1 is known as a positive regulator of the Wnt signalling pathway; a pathway involved in cell fate determination, cell migration, cell polarity, neural patterning and organogenesis during embryonic development 82. As the Wnt signalling pathway is a major player in brain development, it might be relevant in neurodevelopmental disorders such as ASD. Interestingly, many genes of the Wnt signalling pathway have been associated with autism. It has been suggested that Wnt pathway hyperactivity could mediate ASD 83.

Kinesins are molecular motors that transport cargo in the cell. Kinesin family member 14 has not been mentioned in relation to ASD, but other members of the kinesin family such as *KIF22*,* KIF1A*,* KIF5C*,* KLC2* have been linked to ASD previously 84,85.

Integrins are heterodimeric alpha- and beta-subunit containing membrane receptor proteins that interact with the extracellular matrix and play a role in tissue repair, hemostasis, immune response, embryogenesis and metastasis. Integrin alpha 6 is a receptor for laminin in epithelial cells and laminin (the *LAMC3* gene) has been suggested as a candidate gene for autism 86. A past study found evidence of an association between the Integrin alpha 4 gene and autism, but none with the Integrin alpha 6 gene in an Irish autism sample 87. Moreover, changes or loss of extracellular matrix in the brain of the BTBR T+ tf/J mouse model for autism have been reported 88.

The GRTP1 protein is a protein with a possible function as an activator of the GTPase, Rab 89. A rare single-gene copy number variants in the TBC1 domain family, member 5 were observed multiple times among 996 individuals of European ancestry with ASD but not in 1287 matched controls 90. Therefore, GRTP1 could be a potential novel biomarker in ASD.

The parotid secretory protein (PSP, C20orf70) is a soluble cargo protein related to bactericidal/permeability increasing protein (BPI) with suggested anti-bacterial and anti-inflammatory functions 91,92. PSP has also been identified as an HDL-associated protein and therefore could play a role in cholesterol metabolism, which has been previously shown to be dysregulated in ASD 76.

Prolactin-inducible protein (PIP) is a protein known to play a major role in immunoregulation, fertility, antimicrobial activity, apoptosis and tumour progression. Its expression is up-regulated by prolactin and androgens, and downregulated by estrogens. Increases in PIP are considered to be a biomarker for breast and prostate cancer 93, therefore, it may have immune system regulatory functions 93. We found significant elevations in this protein in our prior analysis of ASD saliva in the same individuals using nanoLC-MS/MS 34 and finding this protein elevated in ASD in the current study is confirmatory to our previous study. However, we would also like to mention that while this protein was up-regulated in ASD (spot #662 in Table2 and Fig.2), we also found that specific isoforms of this protein are downregulated in ASD (spot #777 in Table2 and Fig.2). Therefore, while *overall* PIP is increased, as demonstrated in our previous study 34, this PIP also has isoforms or post-translationally modified isoforms that should also be considered, thus demonstrating the utility of 2D-PAGE and of the complementarity of 2D-PAGE and nanoLC-MS/MS with the in-solution digestion and nanoLC-MS/MS performed in our previous study 34.

Mucin-16 belongs to the transmembrane group of mucins and is part of the defence barrier (mucus) covering epithelial cells in many organs including the respiratory and the gastrointestinal tract. Overexpression of transmembrane mucins has been observed in cancers such as digestive tract lesions. Mucin-16 is used worldwide to monitor patients with ovarian cancer. Furthermore, significant perturbations in gut microflora composition and activity have been found in ASD 94. There is therefore a strong connection between the protective protein mucin-16 and ASD.

MRP14, also called S100A9 is the major calcium-binding protein of neutrophils and monocytes belonging to the S-100 protein family of calcium-binding proteins and predicted to have important functions in inflammation. Upon neutrophil activation or endothelial adhesion of monocytes, it is released and may be detected in serum or body fluids as potentially useful clinical inflammatory marker. Its expression and release seems to be of particular importance in immune and immunopathological reactions 95–97.

Elevations in the immunoregulatory/inflammatory proteins Ig alpha-1 chain C region and immunoglobulin heavy chain constant region alpha-2 subunit are consistent with ASD being characterized by heightened inflammation and immune responses 15,16. Increases in these proteins correspond with our prior observations confirming statistically significant increases in proteins with similar function, Ig kappa chain C region, Ig gamma-1 chain C region and Ig lambda-2 chain C regions 34.

**Figure 3:**
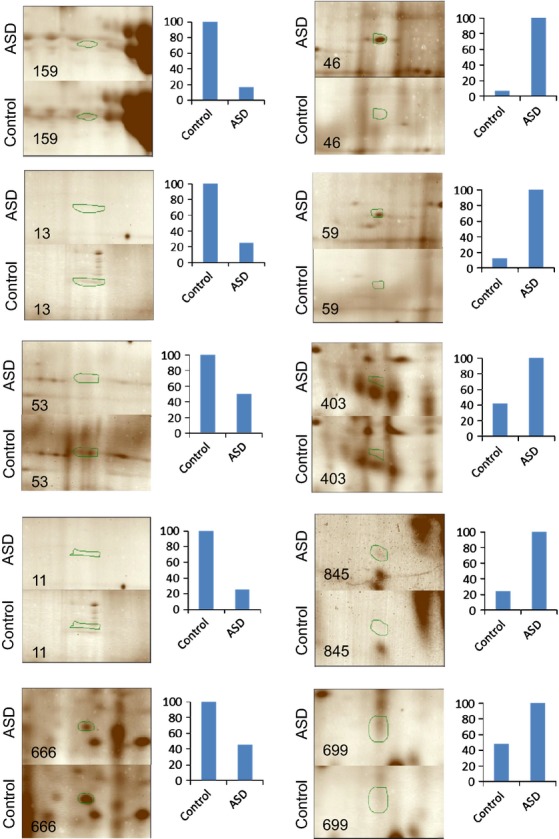
Montage images of protein spots differentially regulated between autism spectrum disorder (ASD) and control samples. Column charts show the ratio in percentage for each dysregulated protein. See Table S1 for spot data and measurements.

Several representative gel images with some of the dysregulated protein spots identified in this study between ASD and Control groups, along with their relative quantitative value is shown in Figure3.

### Biological classification of dysregulated proteins

**Figure 4:**
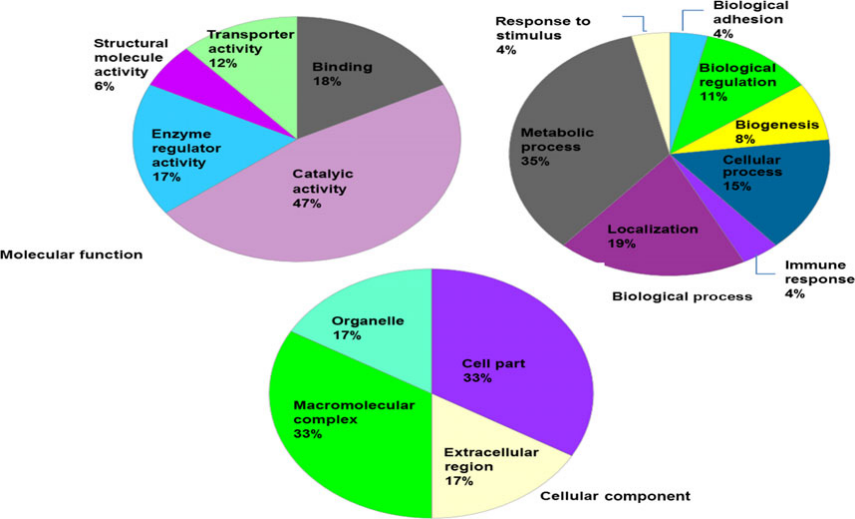
Functional classifications of the dysregulated proteins according to molecular function, biological process and cellular component by PANTHER.

**Figure 5:**
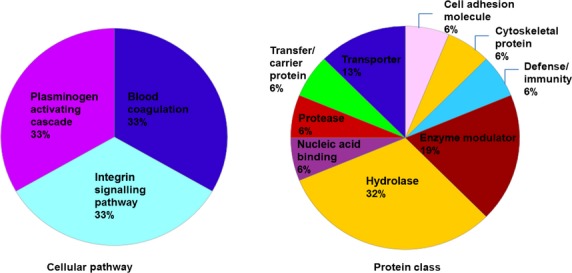
Functional classifications of the dysregulated proteins according to cellular pathway and protein class by PANTHER.

**Figure 6:**
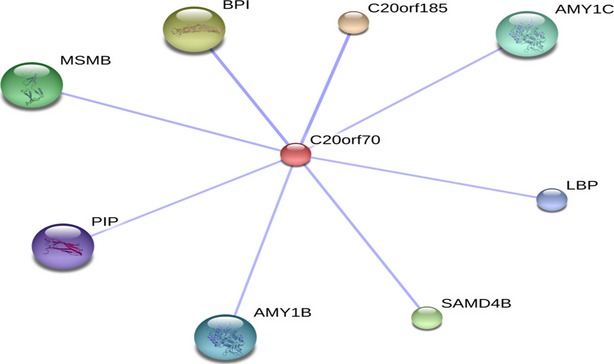
Interaction network of parotid secretory protein (PSP), prolactin-inducible protein (PIP), amylase, bactericidal/permeability increasing (BPI) protein and lipopolysaccharide binding protein (LBP) using STRING.

Dysregulated proteins were investigated with regard to their functional categories and their biological pathways. To this end, we imported these proteins into the PANTHER database 47. PANTHER allows classification and identification of the function of gene products. PANTHER analysis of the dysregulated proteins with regard to molecular function, biological process, cellular component, protein class and cellular pathway is represented in Figures4 and 5. Most dysregulated proteins are catalytically active (47%), whereas the predominant biological process is the metabolic process. For their cellular component, both macromolecular complex and cell part were equally represented (33%). Mostly three cellular pathways were affected: blood coagulation, integrin signalling and plasminogen activating cascade. The majority of the proteins belong to the protein class of hydrolases (32%). To extract additional information from our data set, the same list was submitted to the DAVID 46. DAVID renders functional annotation and interpretation of lists of protein identifications. According to DAVID, most proteins are associated with neurological disorders (Alzheimer's disease, Parkinson's disease, depression). Another interesting finding is that the two most frequent post-translational modifications are glycosylation (N-linked, O-linked) and disulphide formation. Additional pathways highlighted by DAVID (REACTOME and KEGG pathways) were: Wnt signalling pathway, signalling by platelet-derived growth factor, immune system, metabolism of lipids and lipoproteins, metabolism of carbohydrates. Protein–protein interaction (PPI) between the dysregulated proteins was also performed with the STRING 98 and DAVID. For example, PSP is predicted to interact with proteins identified in the present study such as PIP, amylase (AMY), BPI protein and lipopolysaccharide binding protein (Fig.6). In general, CBP seems to be at the heart of most PPIs suggesting that this protein might play a major role in ASD.

We note that medication effects could influence levels of proteins. For example, hyperprolactinemia is a common side effect in with long-term risperidone treatment 99. Only one participant in the present analysis was taking risperidone (A2, see Table1). Because this is a pooled analysis, we cannot comment on biomarkers in this individual based on the present analysis; however, in a prior publication, we analysed proteins at the individual level 34, using the same participants. We did not observe a distinct pattern of protein elevations in this individual that were characteristic of hyperprolactinemia. The participant A2 did have in the prior analysis^34^, the highest levels of lysozyme C and annexin A1; however, these are not characteristics of risperidone treatment to our knowledge and might have been related to the fact that this individual had the most severe case of autism (see Table1).

## Conclusion

The goal of this study was to investigate putative biomarker candidates in the salivary proteome of children with ASD in relation to typically developing controls. To this end, we used a proteomic strategy based on 2D-PAGE paired with nanoLC-MS/MS (nanoLC-MS/MS). Although this platform has been used in numerous biomarker cancer studies, there is no report on the application of this strategy in ASD. Therefore, we probed the use of 2D-PAGE salivary profiling to find putative ASD. As a result, significant differences were identified between the two groups and their biological relevance to ASD was highlighted. The current set of dysregulated proteins could provide a biomarker signature for ASD as the proteins identified are functionally and physiologically very diverse. Some of the proteins are associated with a subset of symptoms observed in ASD and might be possible markers for ASD subtyping. Most of the dysregulated proteins were already proposed at the gene level as potential risk factors or markers of ASD thus further confirming the role played by these factors in ASD pathophysiology and revealing a complementarity between genomic and proteomic biomarker discovery studies. Many of the differentially expressed proteins play a role in some of the suggested pathways implicated in ASD causality: immune response and inflammation, oxidative stress, cholesterol and lipid metabolism 23,76. In future studies, validation of the findings of the current study should be undertaken. The study could be repeated using a greater number of pooled samples (at least 20) to reduce the effects of inter-individual variability on the outcome of this study and to increase significance.

Investigating a protein or a protein mixture using one method may lead to identification and characterization of a protein, a protein isoform or a post-translationally modified protein. However, one approach does not identify and characterize all protein isoforms in one experiment and screening, and to examine whether furthermore, complementary approaches are needed, as demonstrated in this study. For example, our current 2D-PAGE study confirms some of the proteins identified in our previous study on the same saliva protein samples using a different proteomics approach. However, the 2D-PAGE also identified new dysregulated proteins and protein isoforms, thus demonstrating its complementarity to other recently published results obtained by other methods 34. Overall, our data suggest that 2D-PAGE coupled to LC-MS/MS is employable as a diagnostic screening tool of putative biomarkers of neurodevelopmental disorders such as ASD. The results obtained in this study may contribute to a protein signature of ASD risk and subtype as well as possible therapeutic targets.

## Supporting Information

**Figure S1** The Mascot MS/MS spectra and their corresponding MS/MS spectra from the original raw data (.raw).

**Table S1** Summary results for 2D gel comparison of saliva samples.
