# Supplementary material for: Comparative two-dimensional polyacrylamide gel electrophoresis of the salivary proteome of children with autism spectrum disorder
Source: J Cell Mol Med. 2015 Aug 20;19(11):2664–78. doi: 10.1111/jcmm.12658 (PMC4627571; doi:10.1111/jcmm.12658)
Supplement: Supplementary file 1 — Figure S1 The Mascot MS/MS spectra and their corresponding MS/MS spectra from the original raw data (.raw). [file jcmm0019-2664-sd1.pptx]

## Slide 1
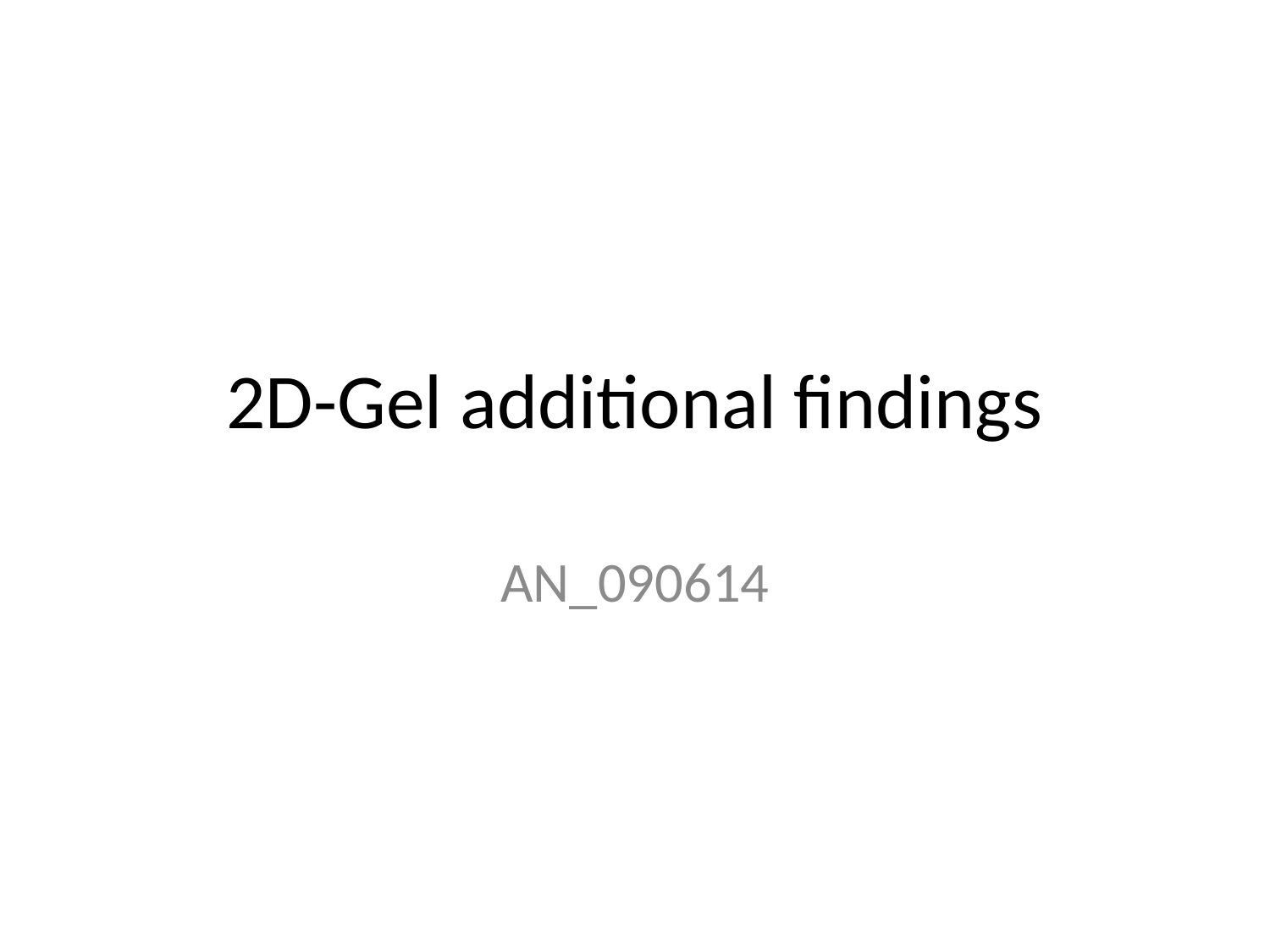

# 2D-Gel additional findings
AN_090614

## Slide 2
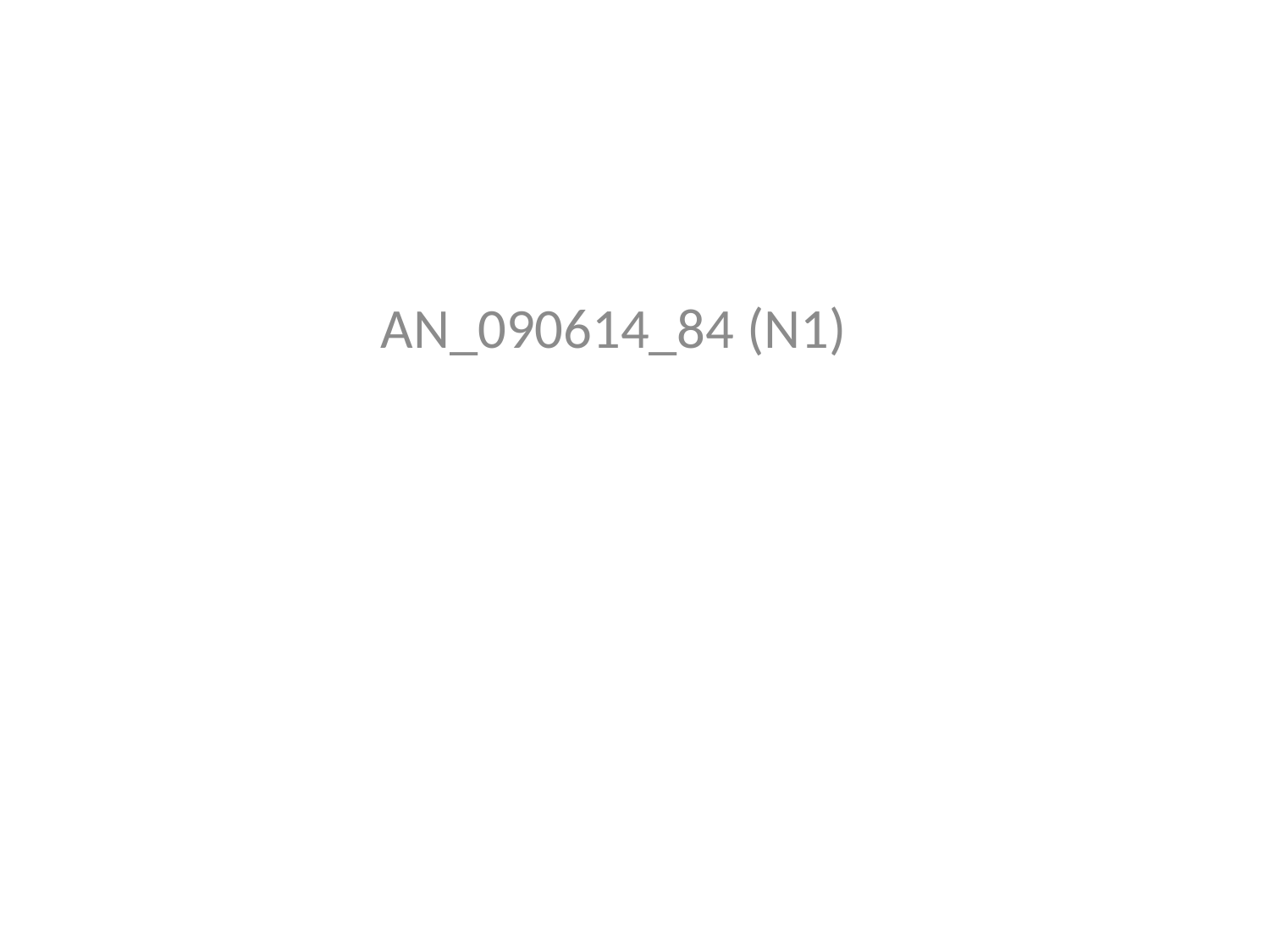

AN_090614_84 (N1)

## Slide 3
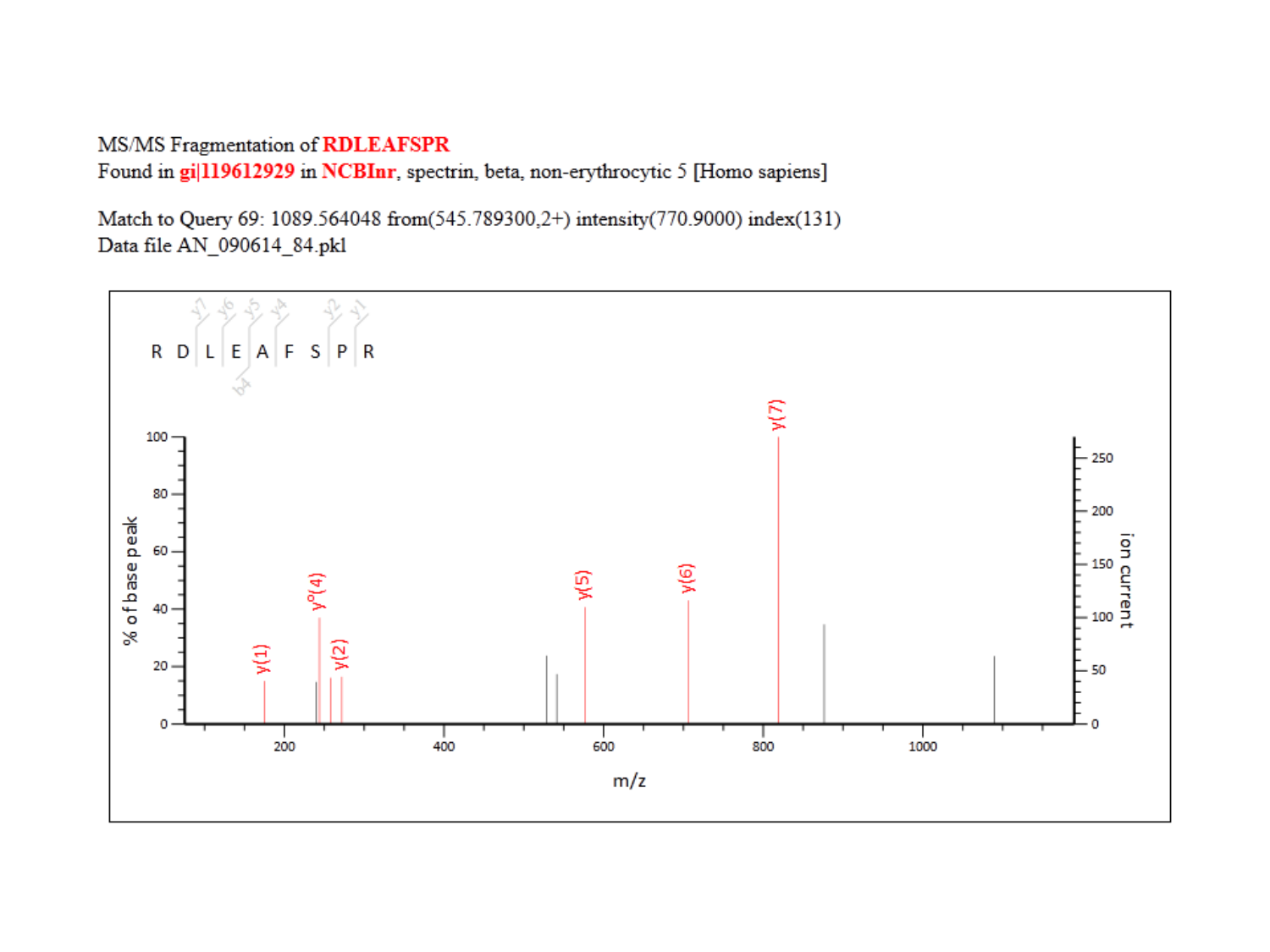

## Slide 4
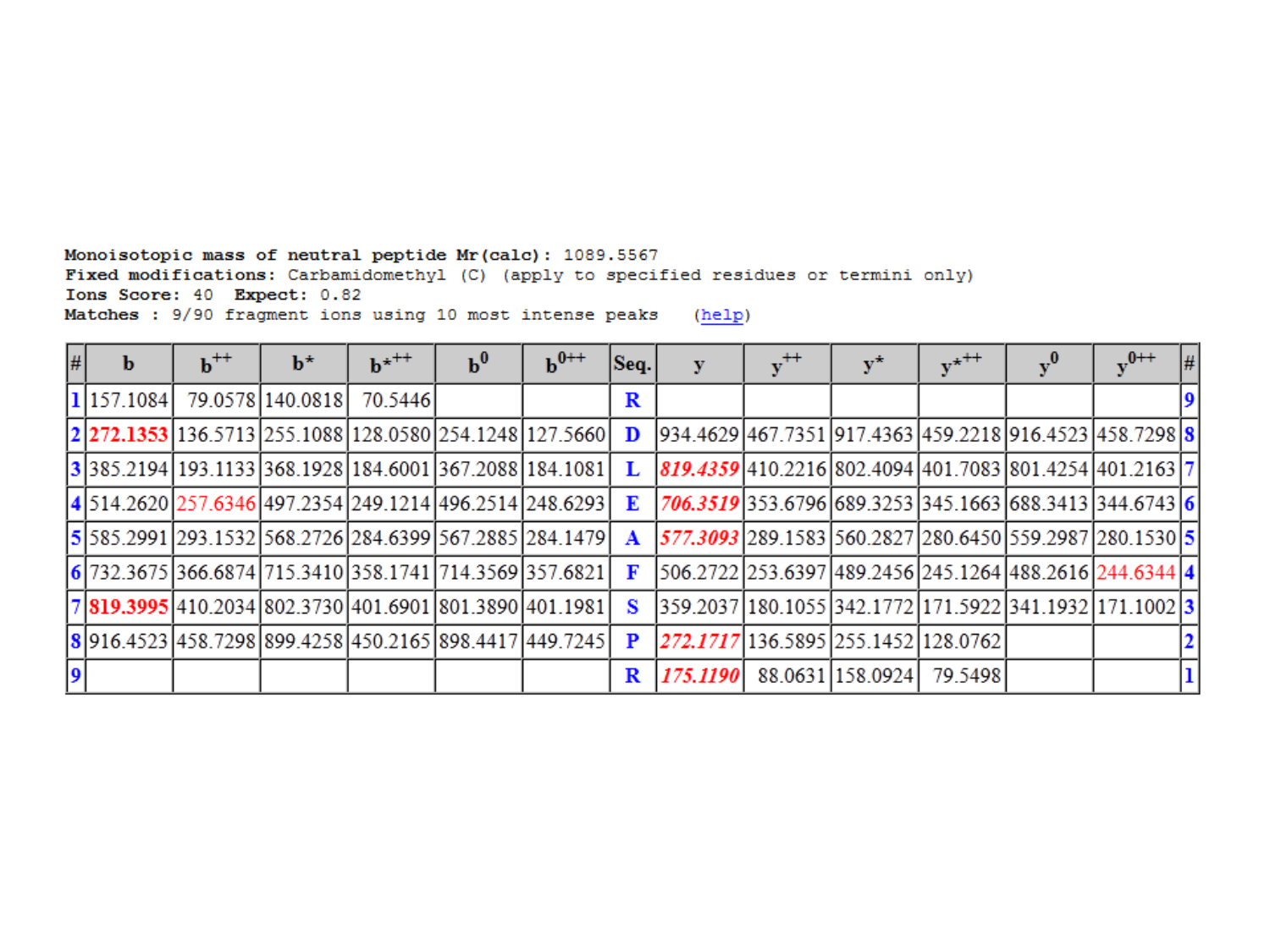

## Slide 5
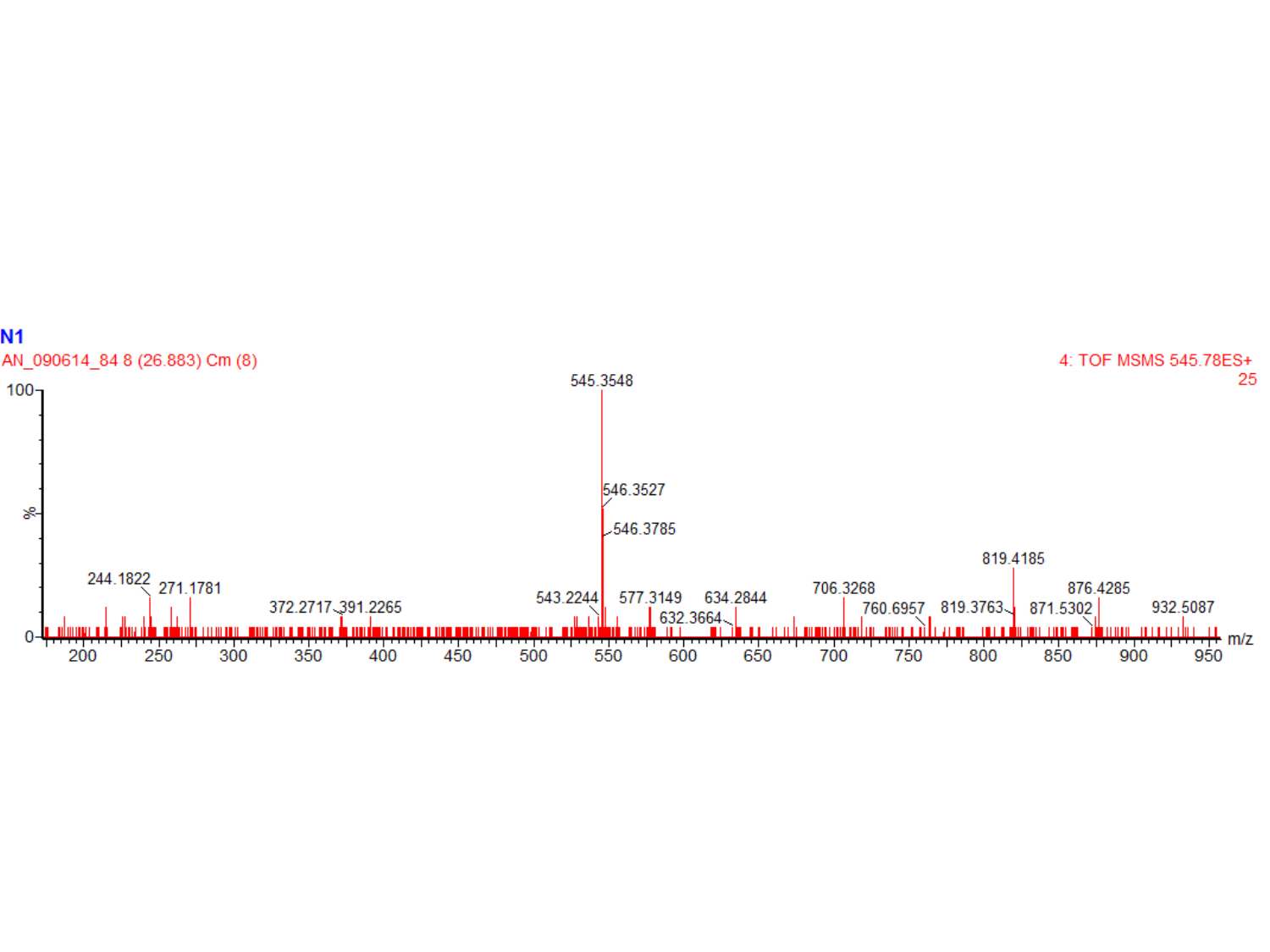

## Slide 6
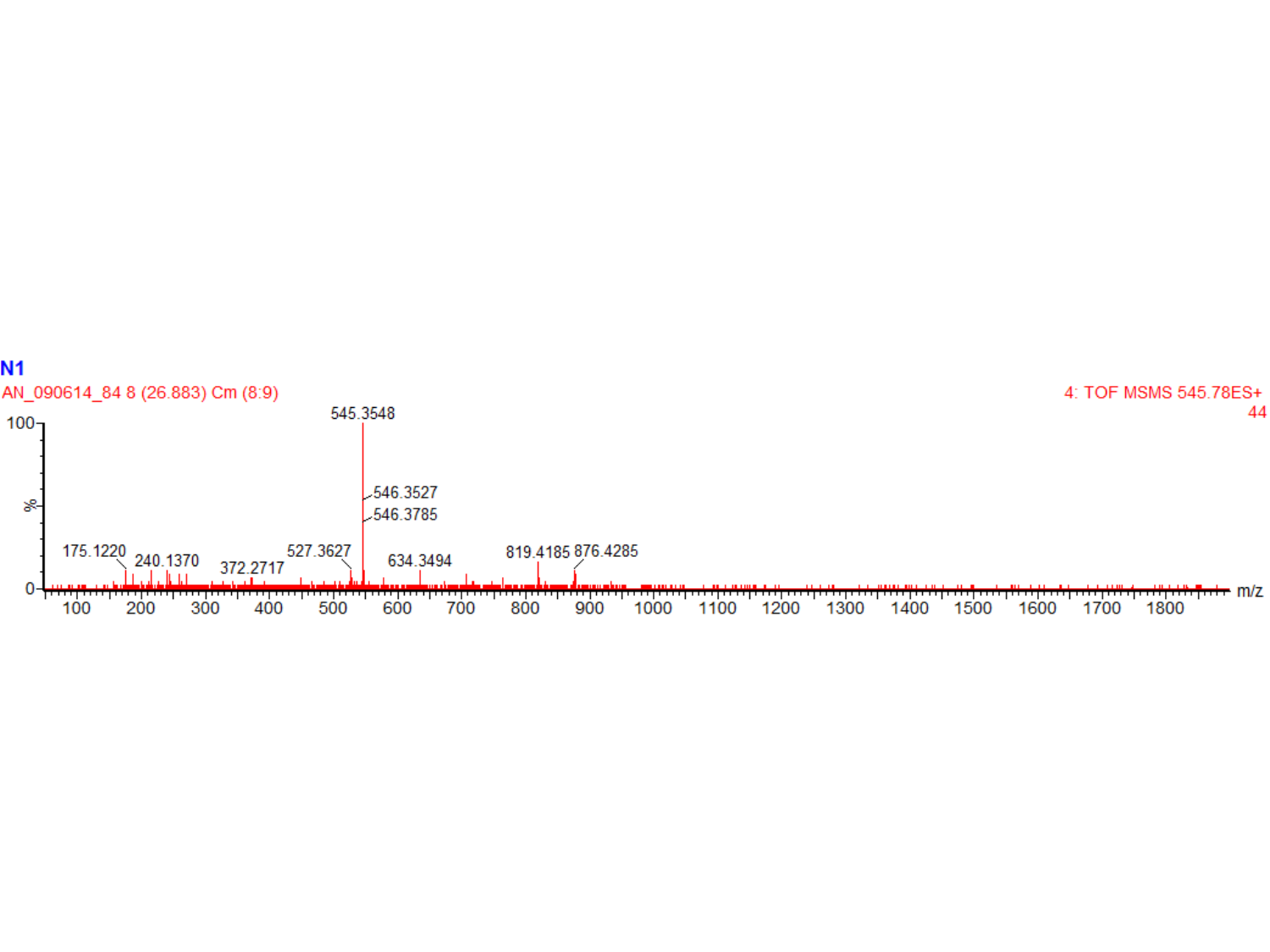

## Slide 7
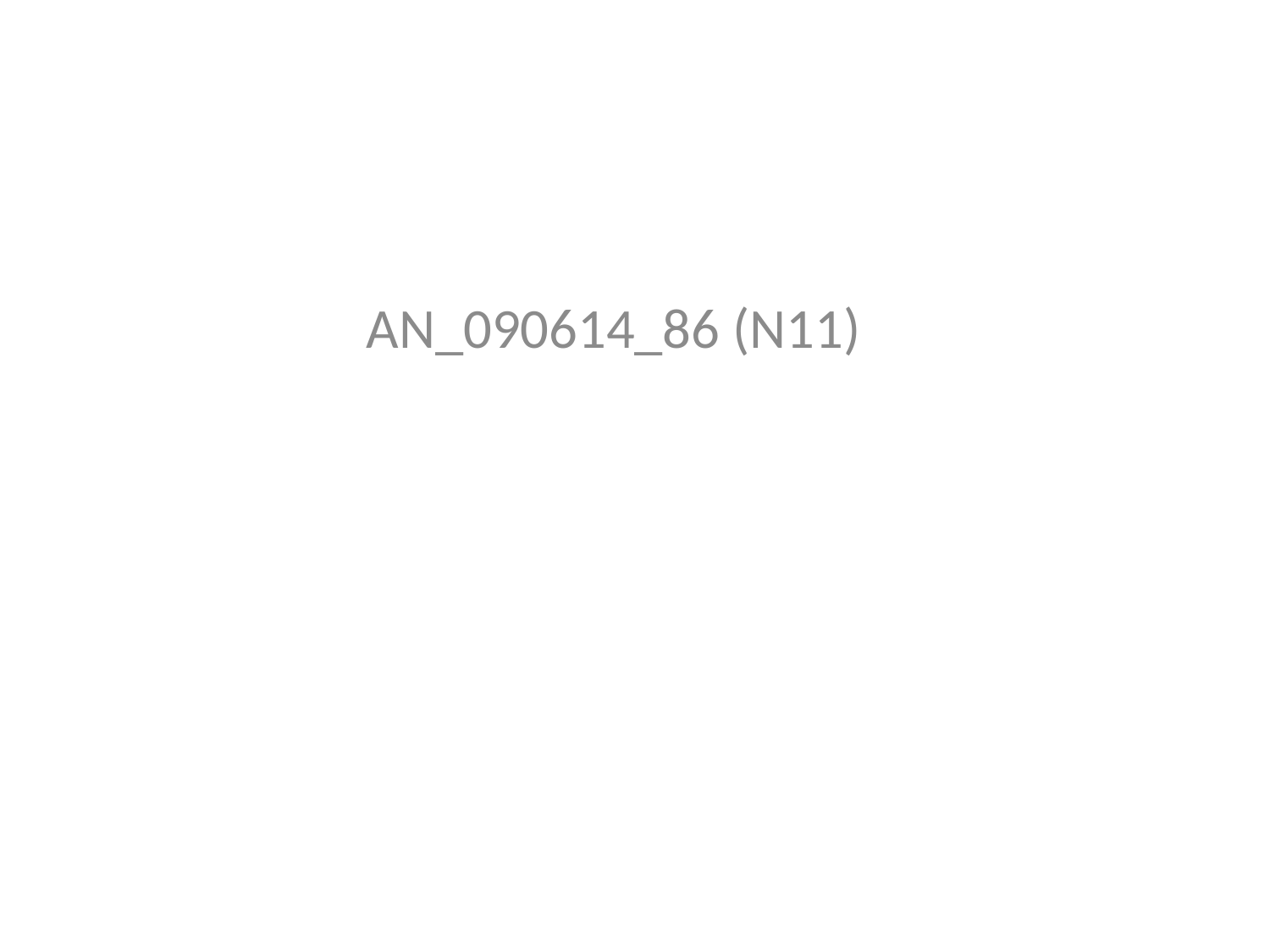

AN_090614_86 (N11)

## Slide 8
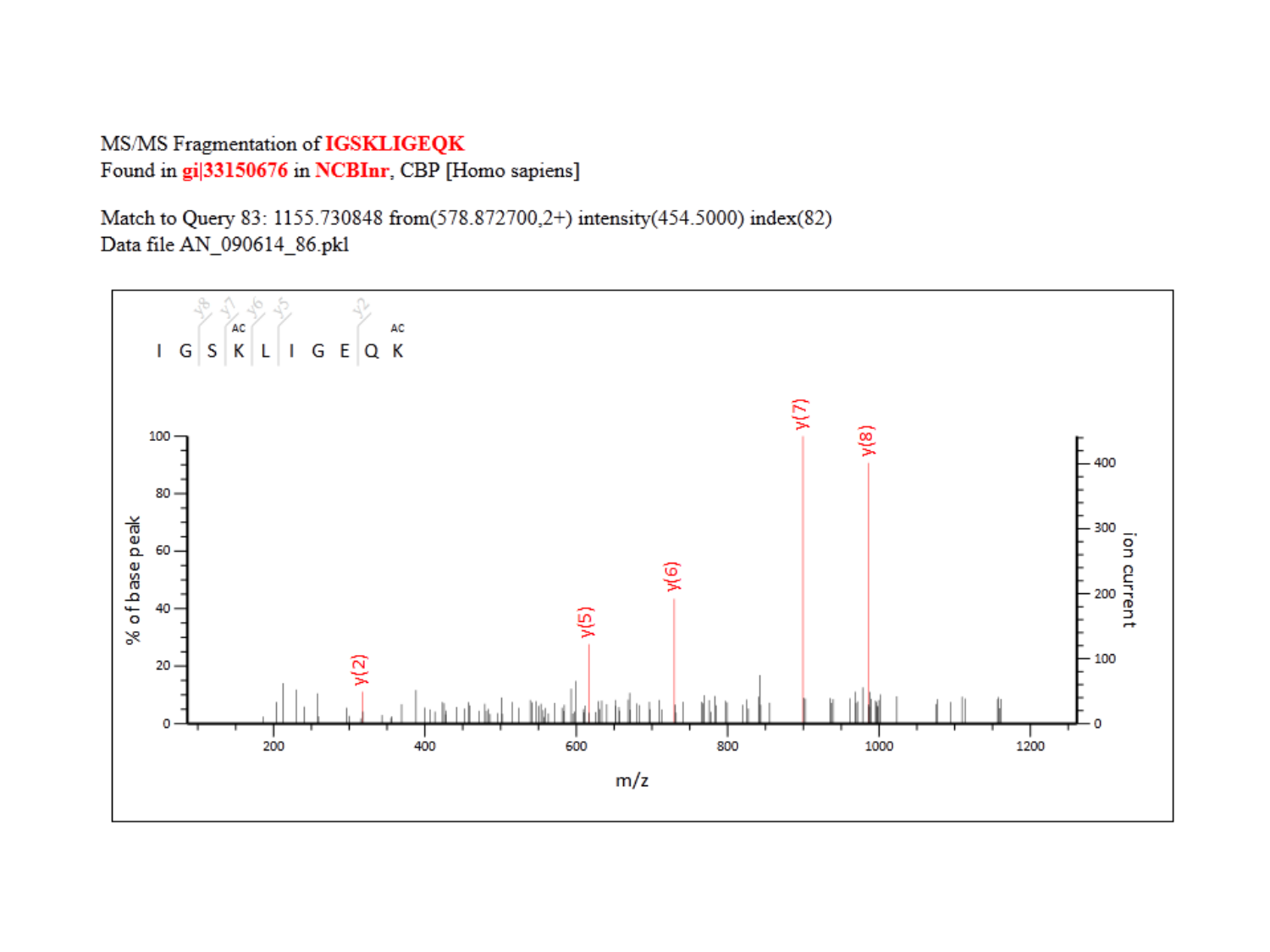

## Slide 9
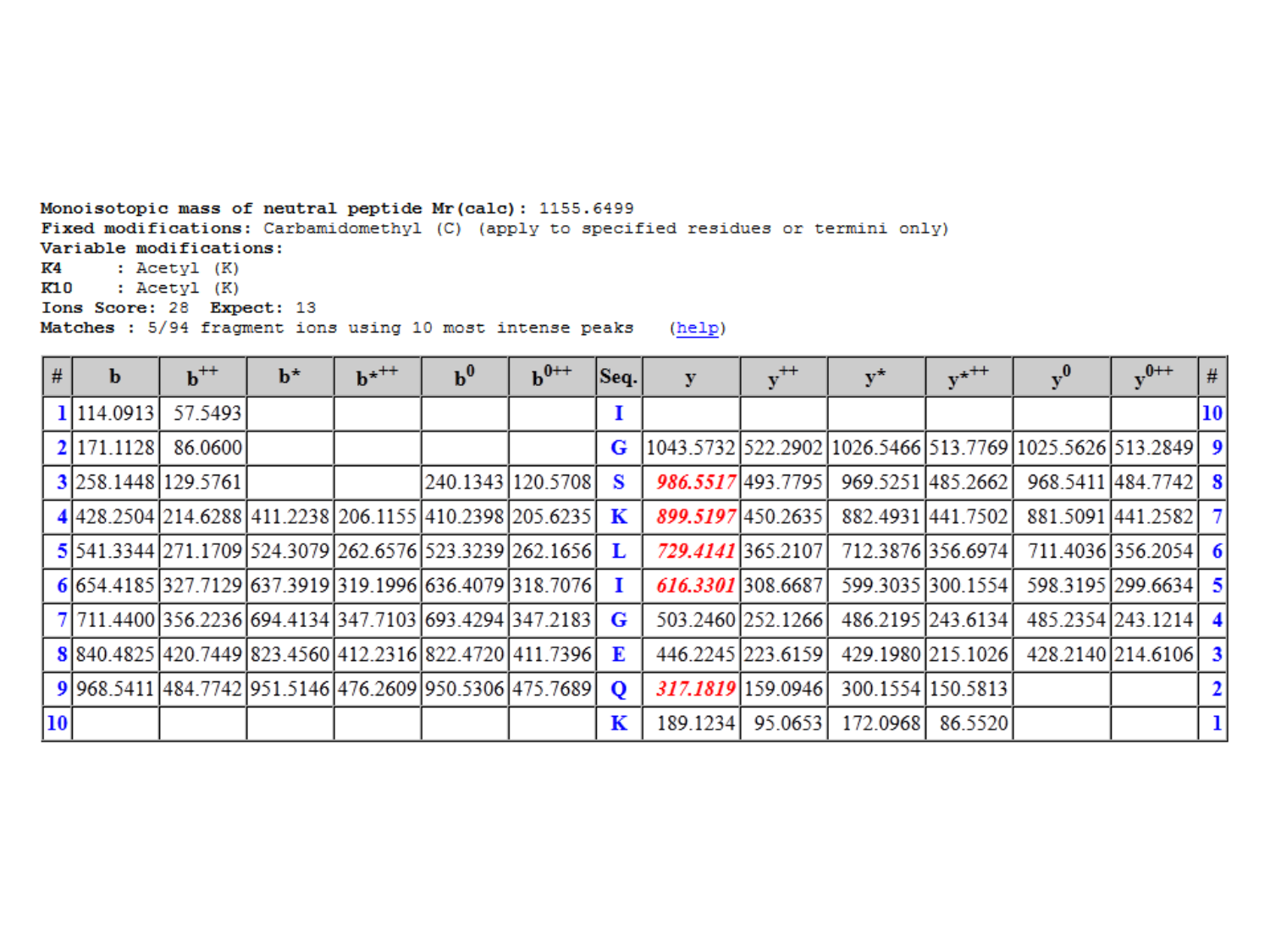

## Slide 10
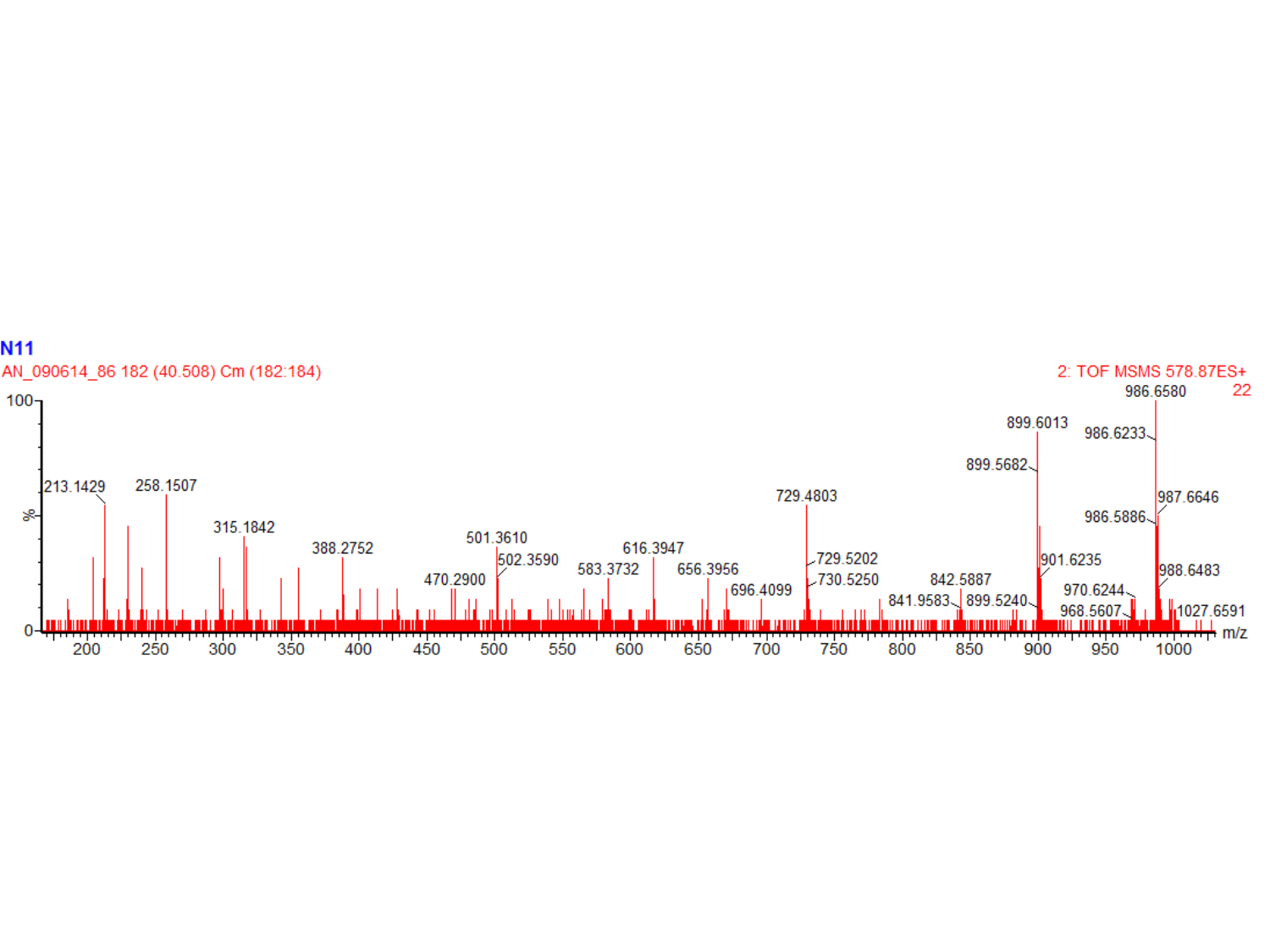

## Slide 11
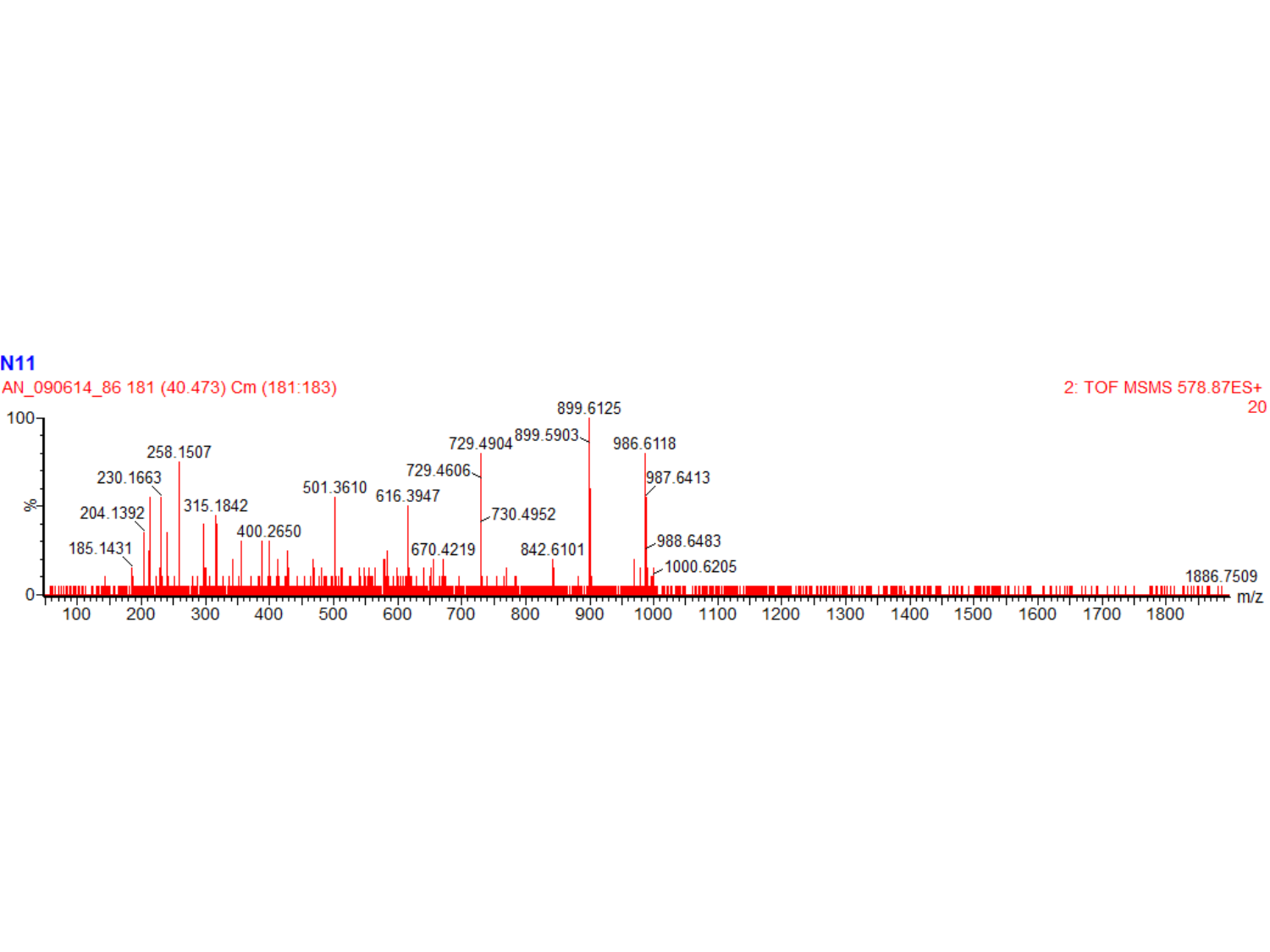

## Slide 12
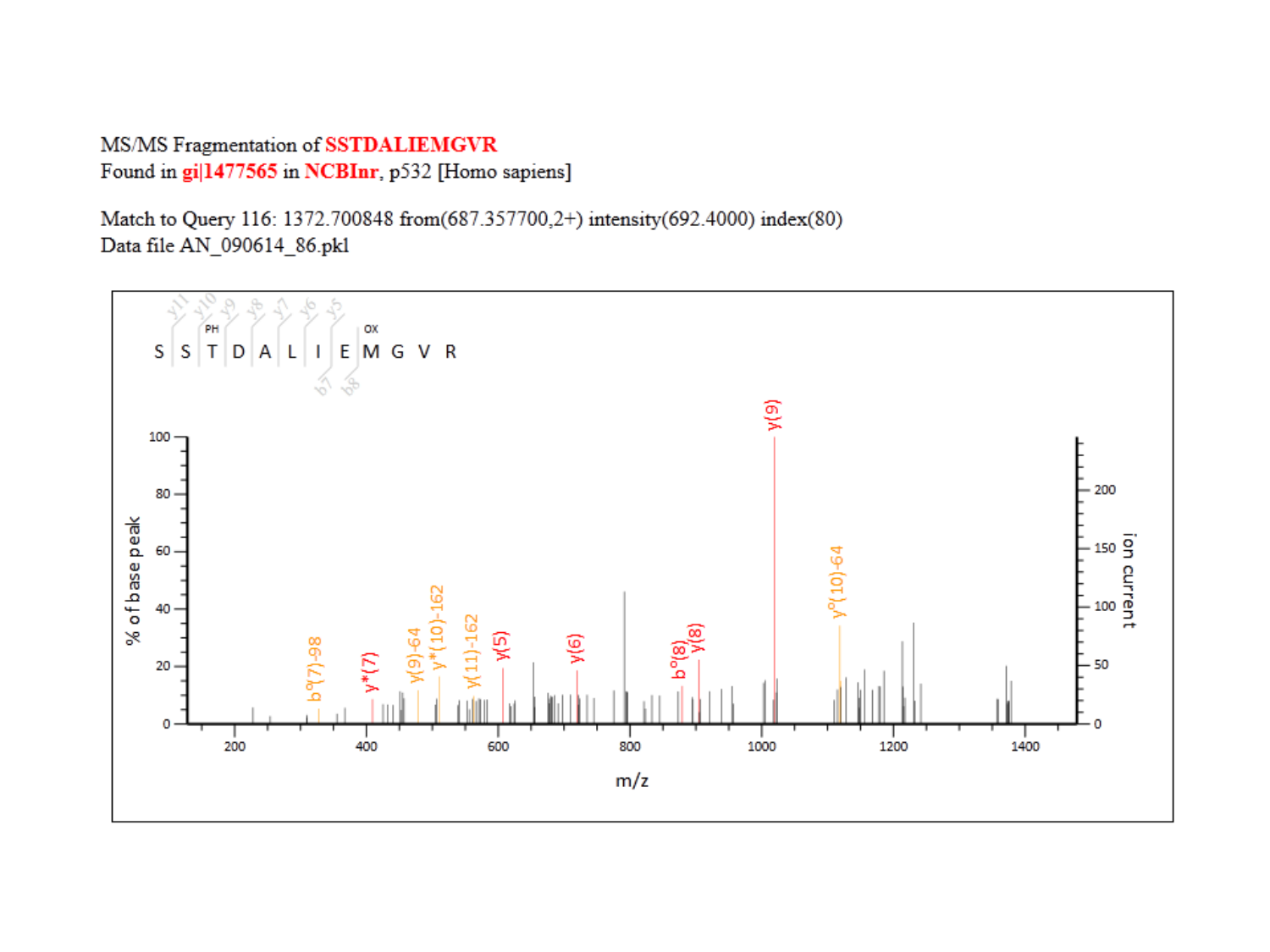

## Slide 13
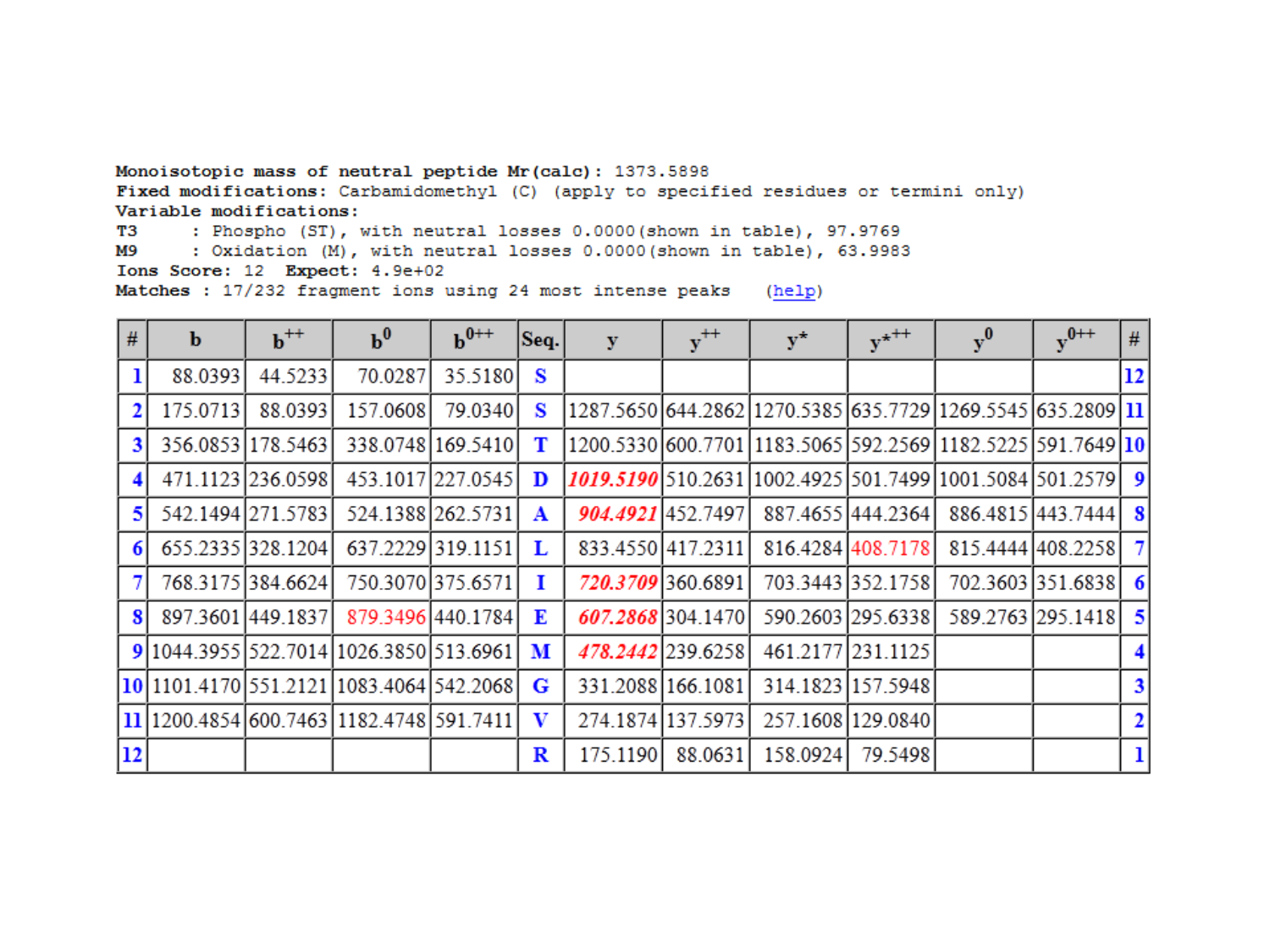

## Slide 14
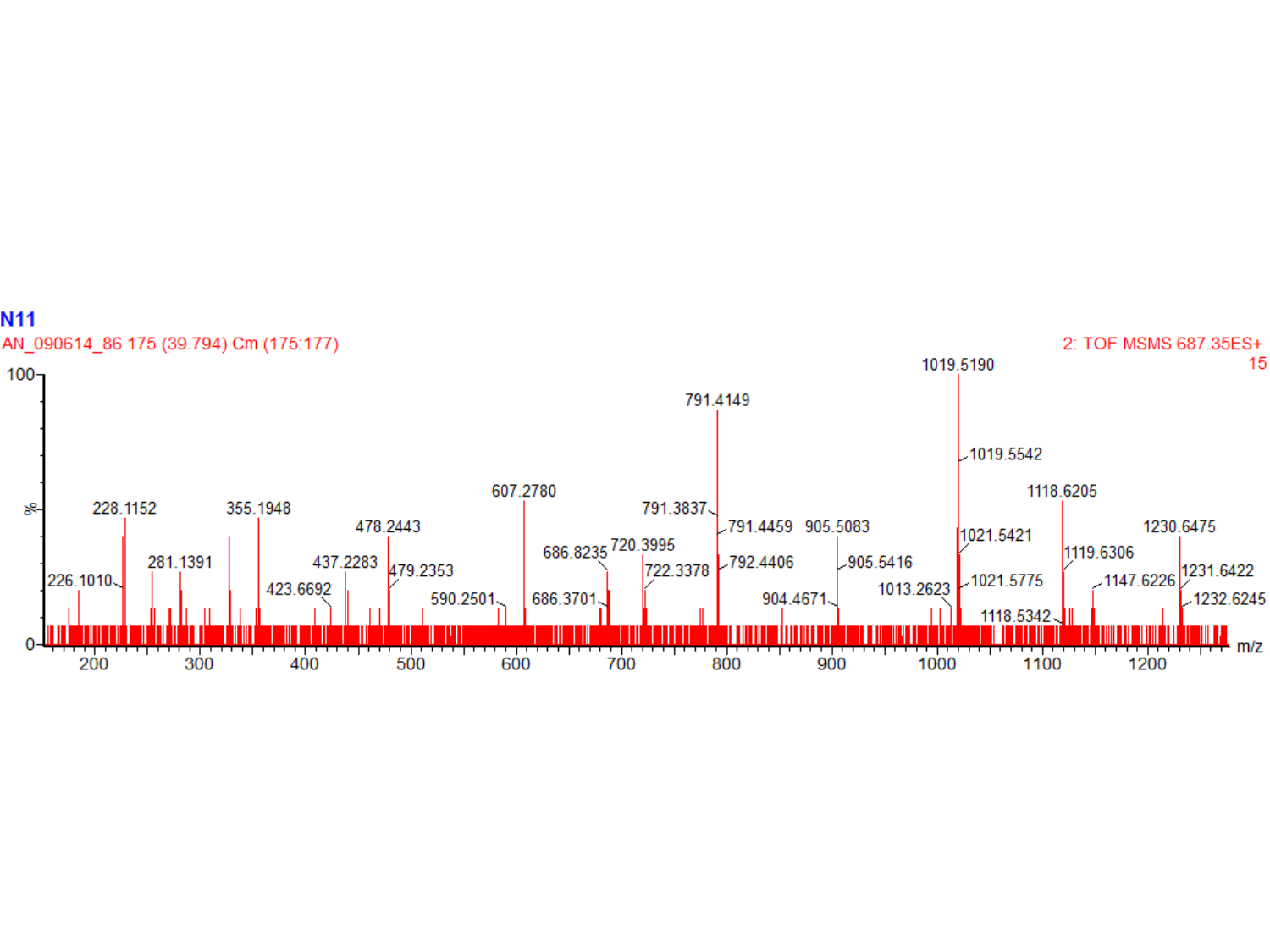

## Slide 15
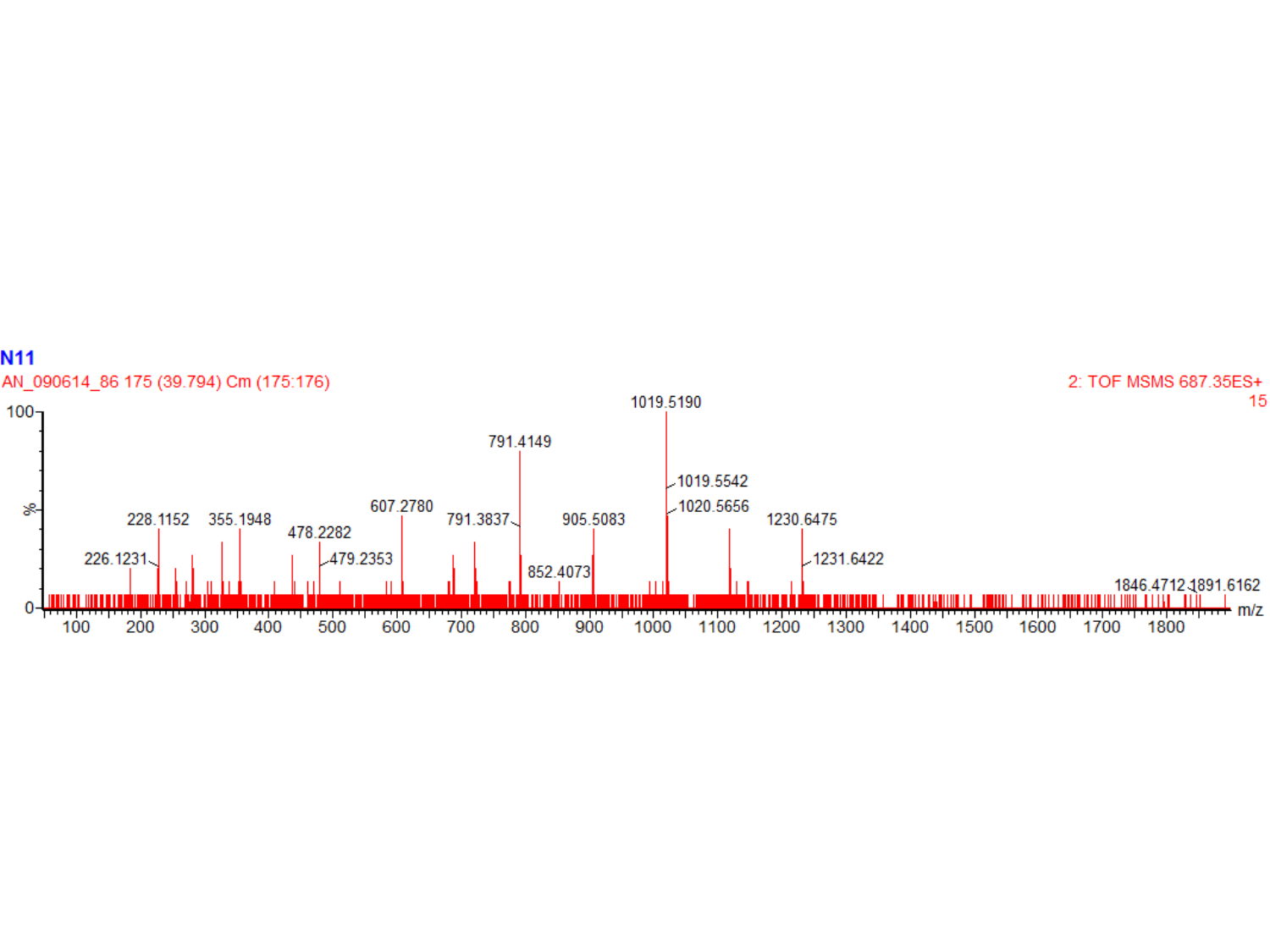

## Slide 16
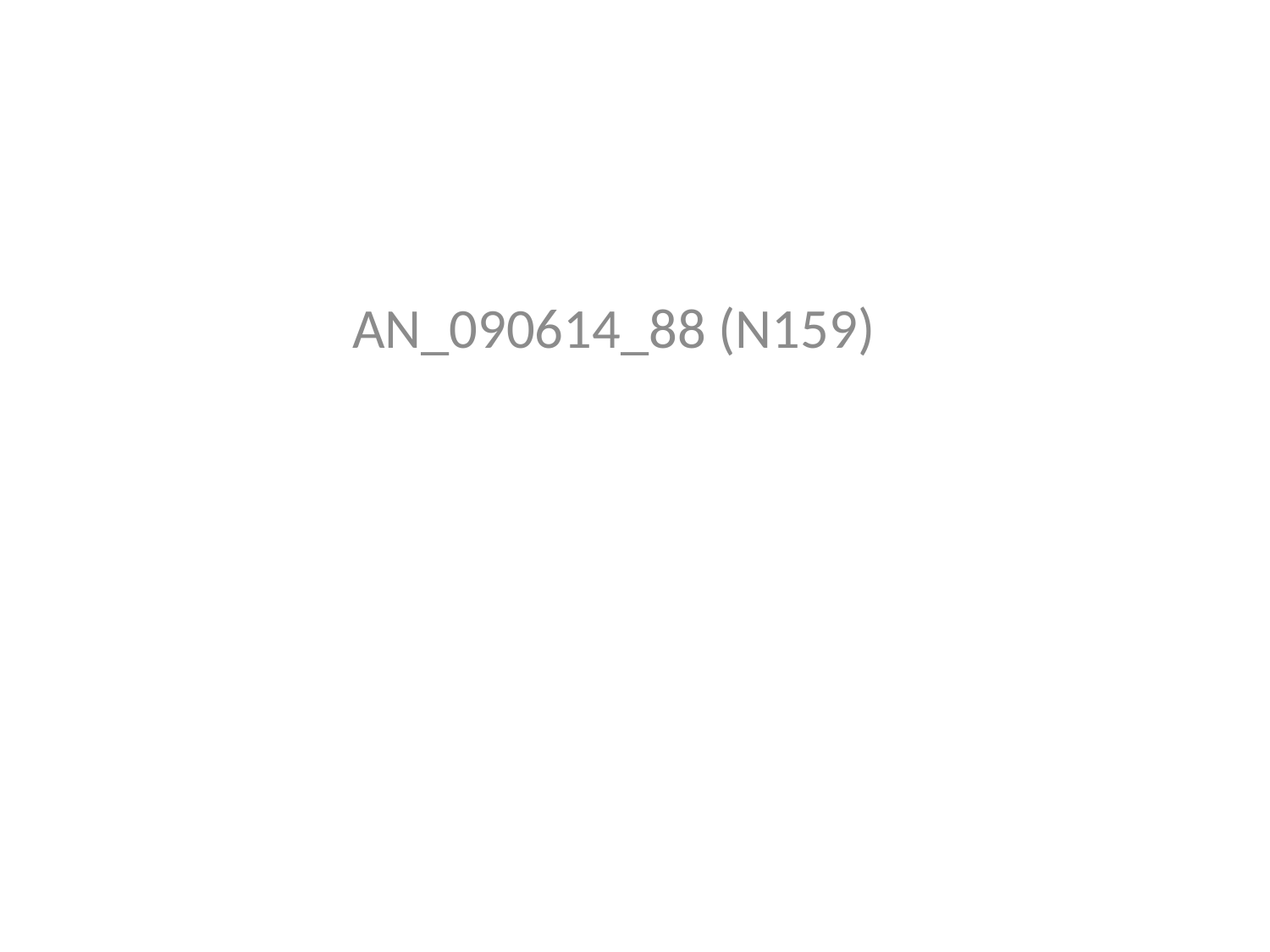

AN_090614_88 (N159)

## Slide 17
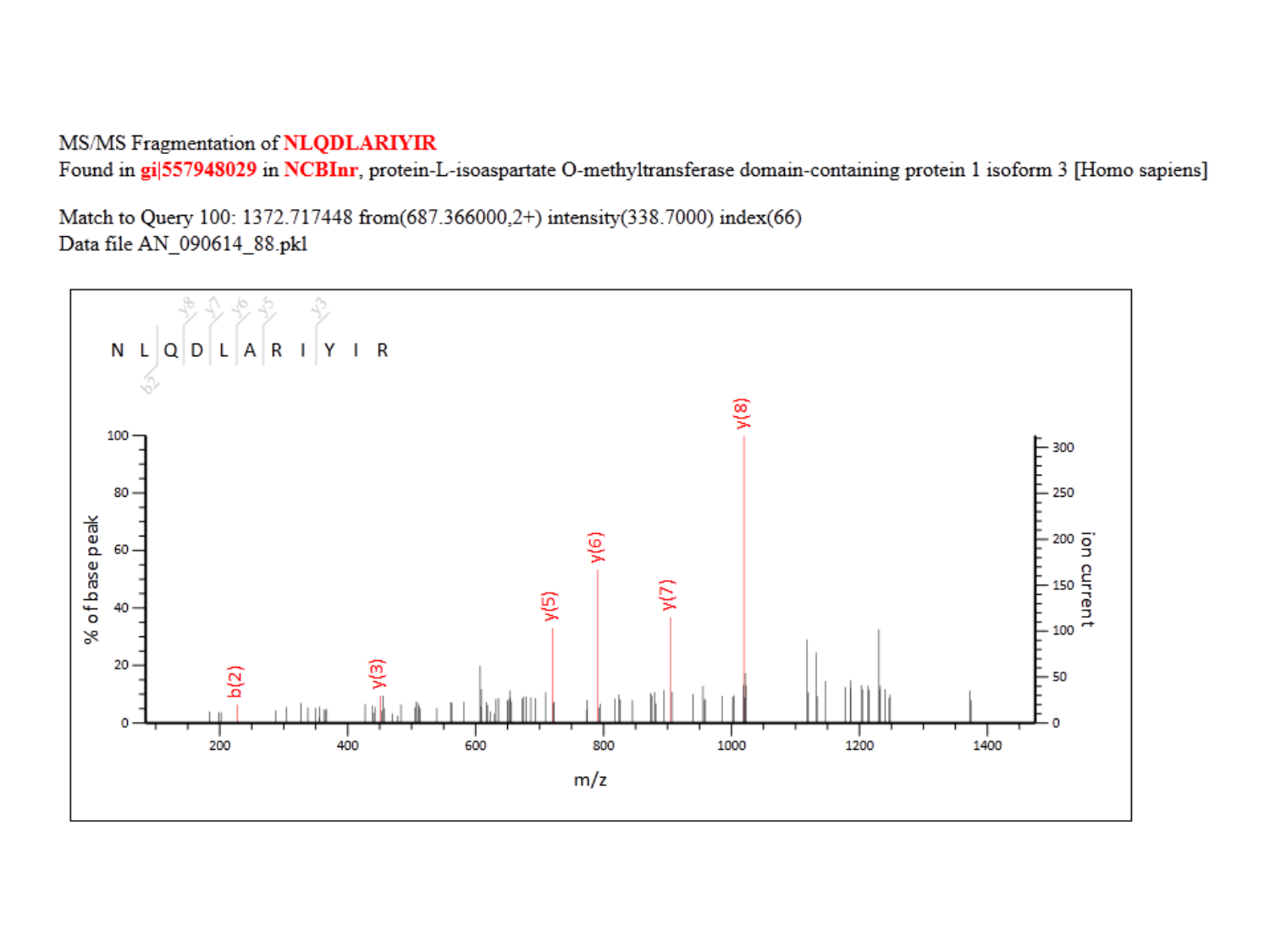

## Slide 18
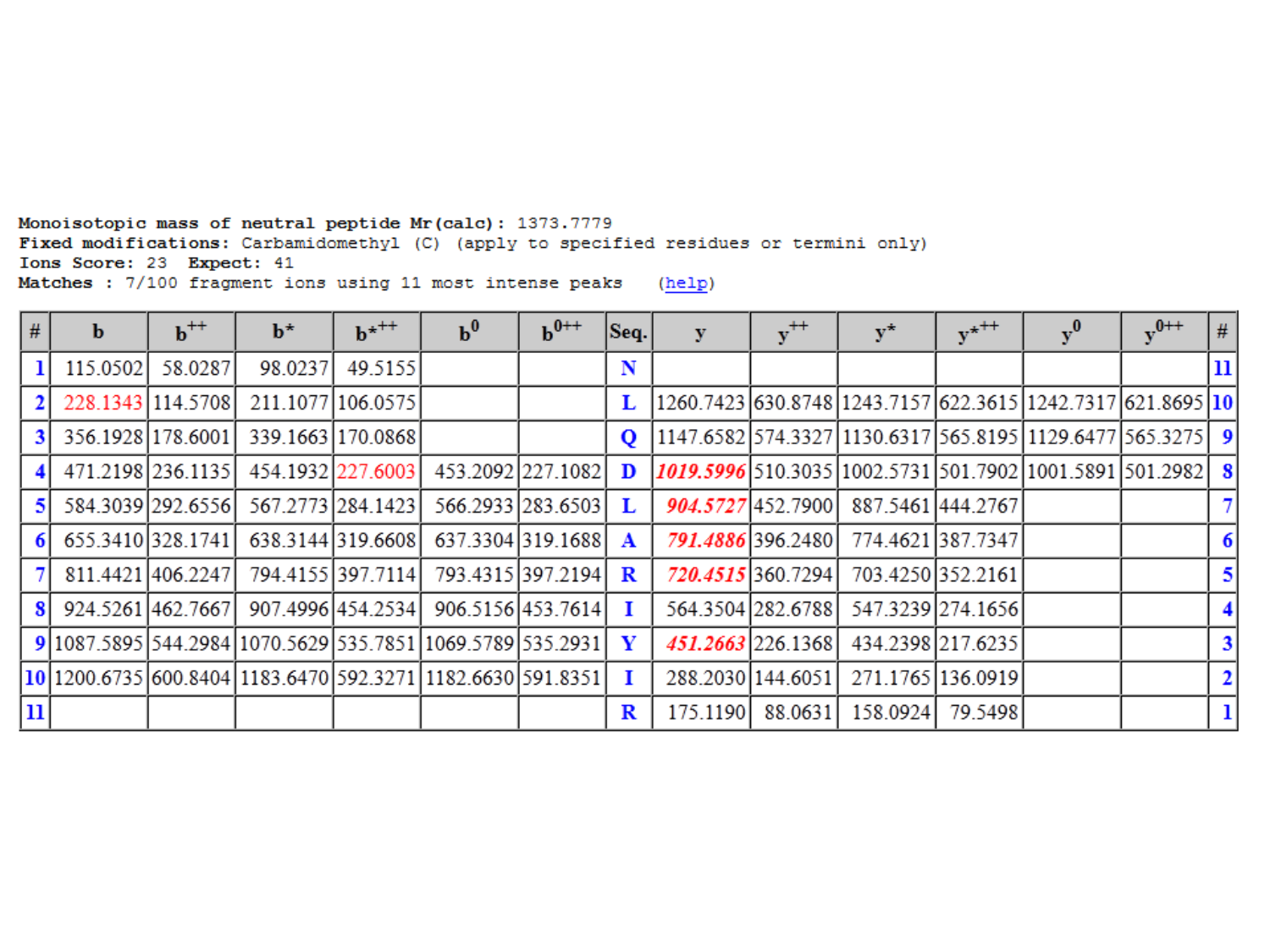

## Slide 19
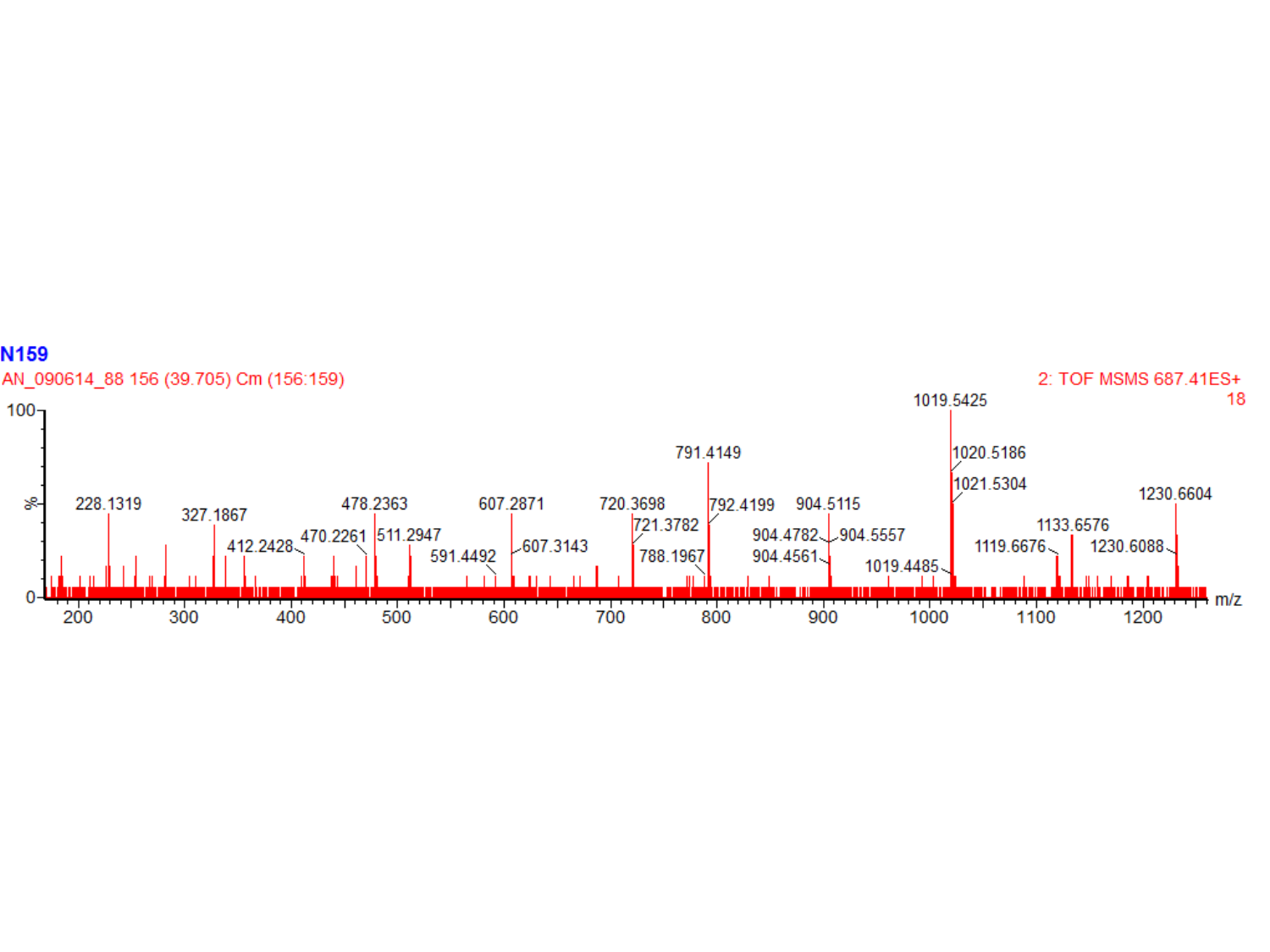

## Slide 20
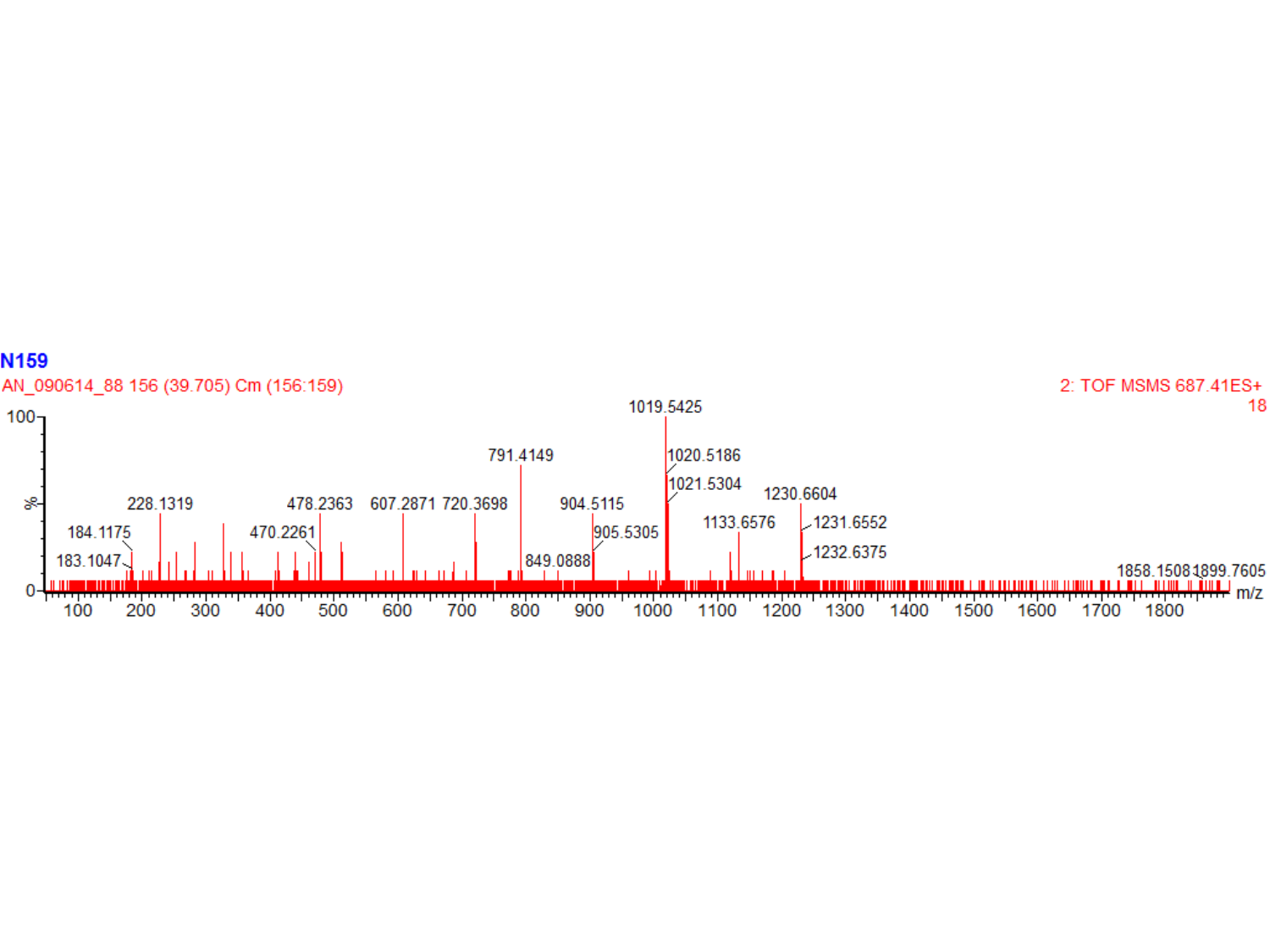

## Slide 21
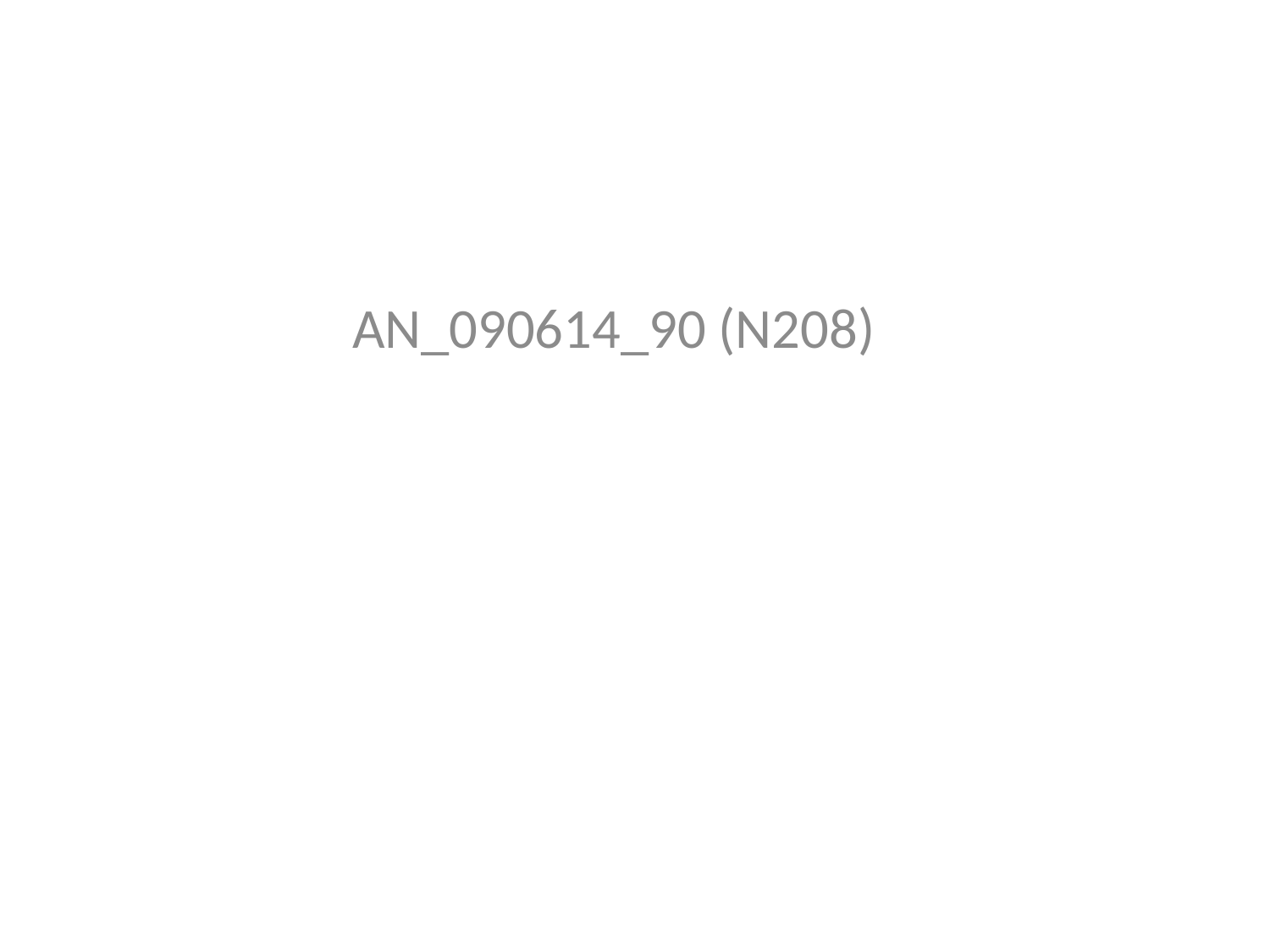

AN_090614_90 (N208)

## Slide 22
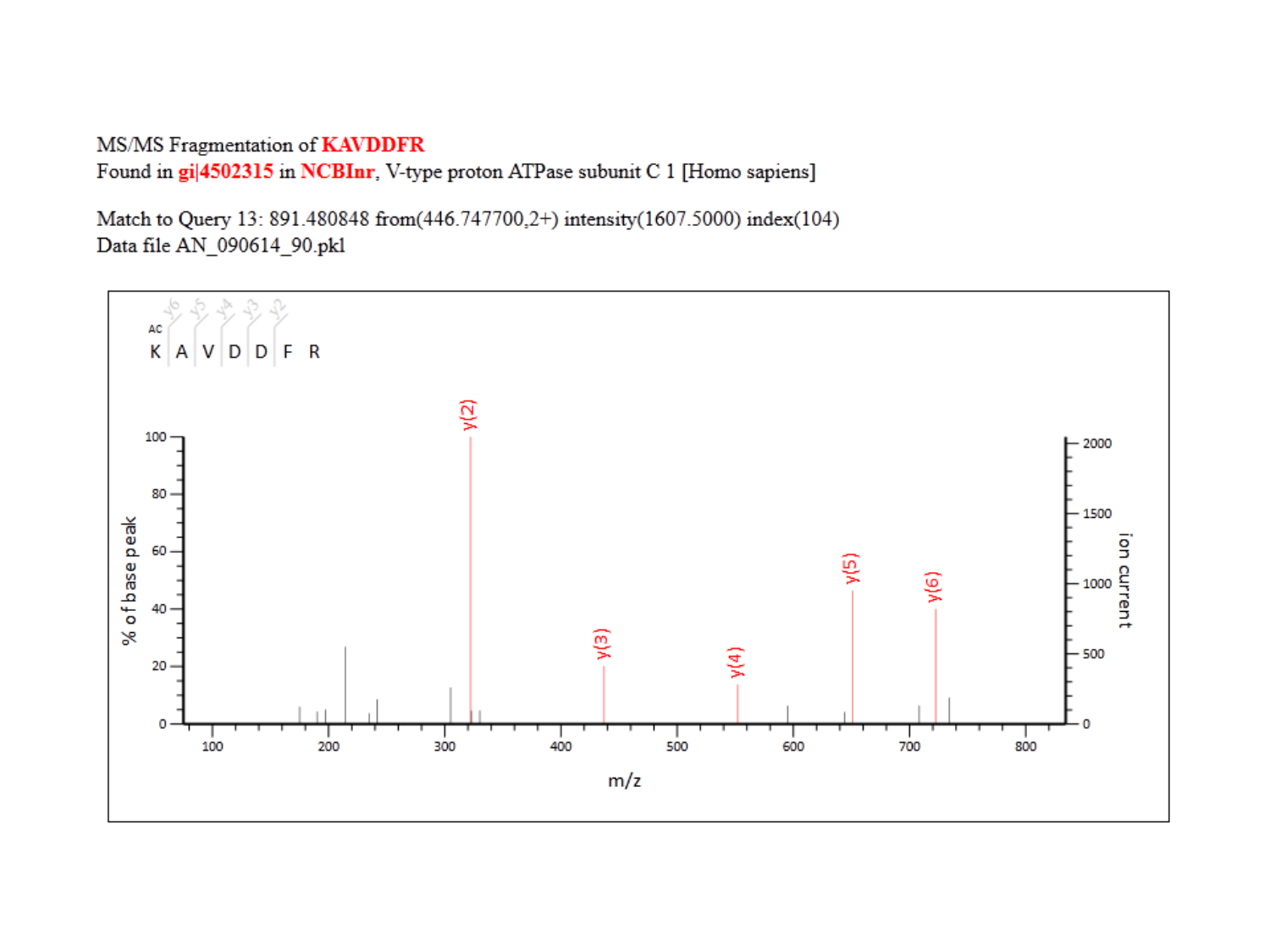

## Slide 23
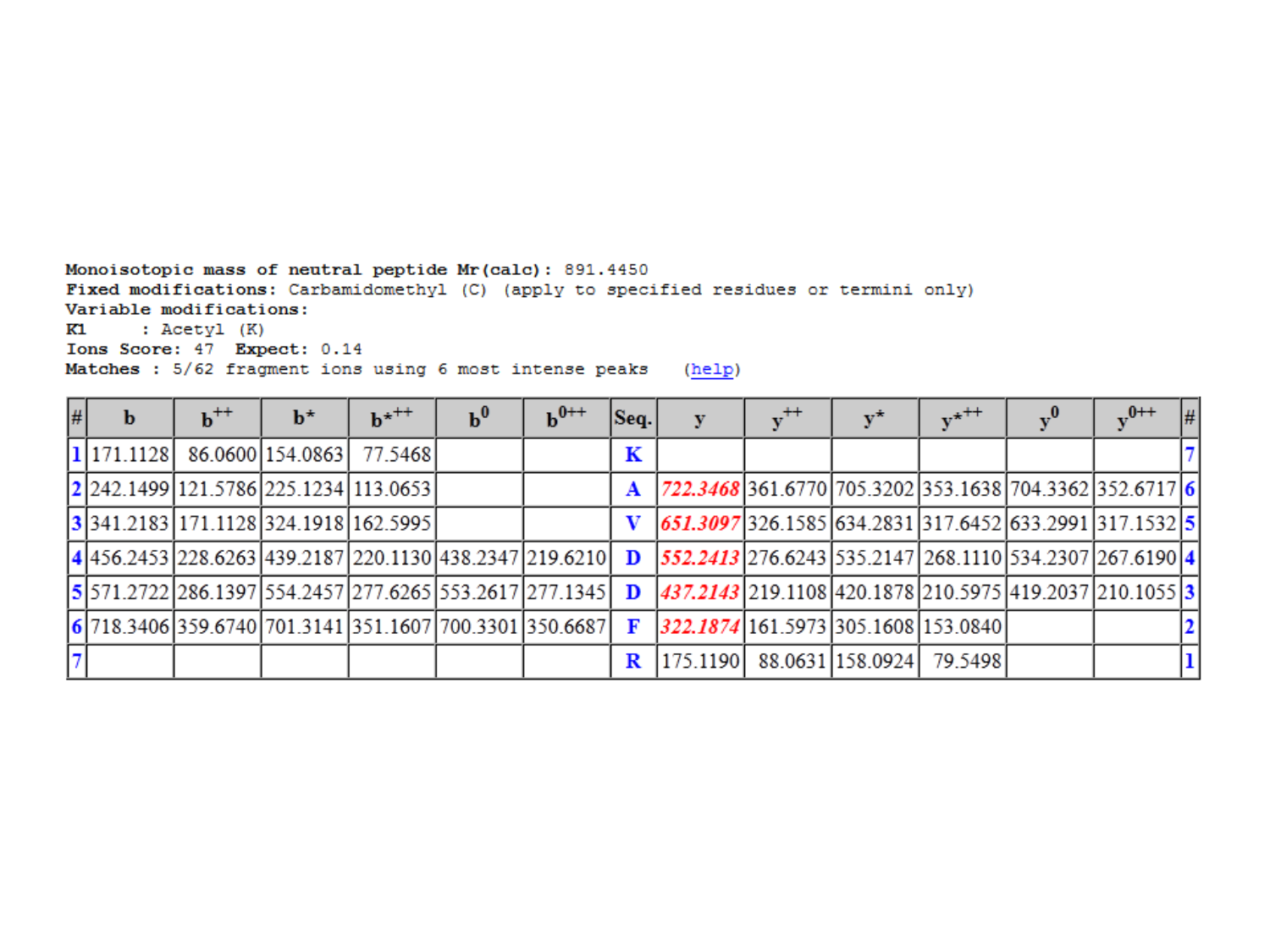

## Slide 24
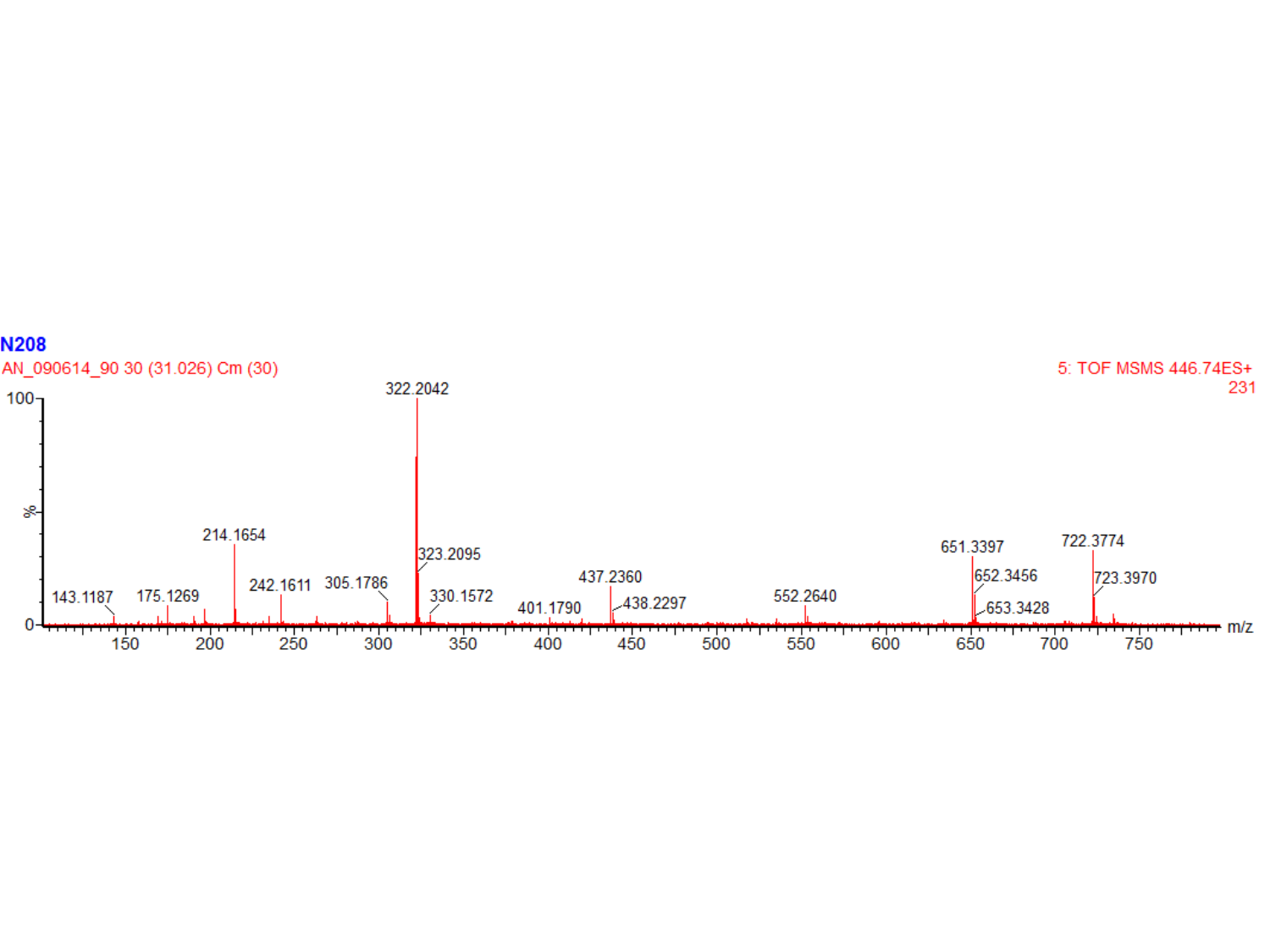

## Slide 25
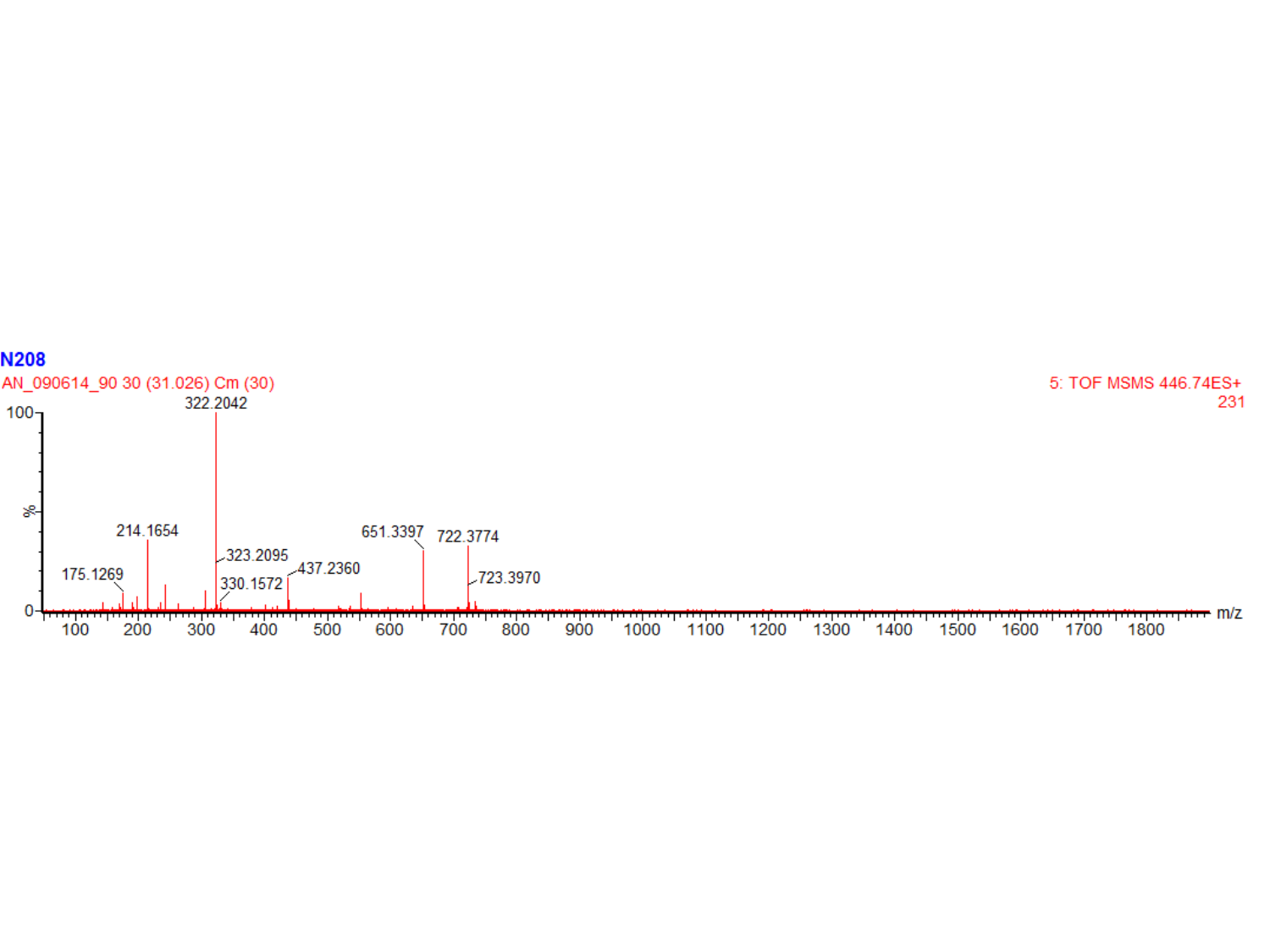

## Slide 26
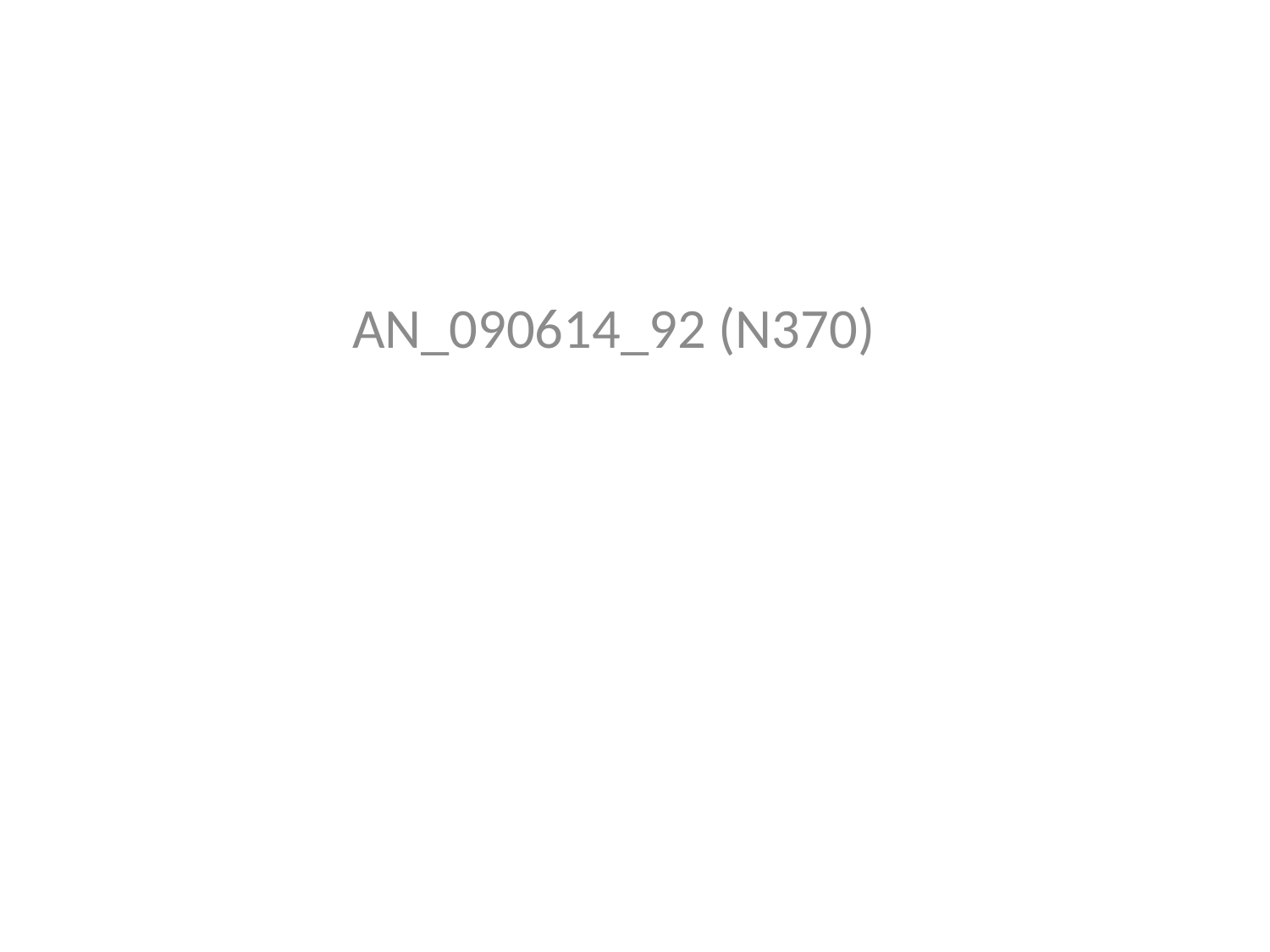

AN_090614_92 (N370)

## Slide 27
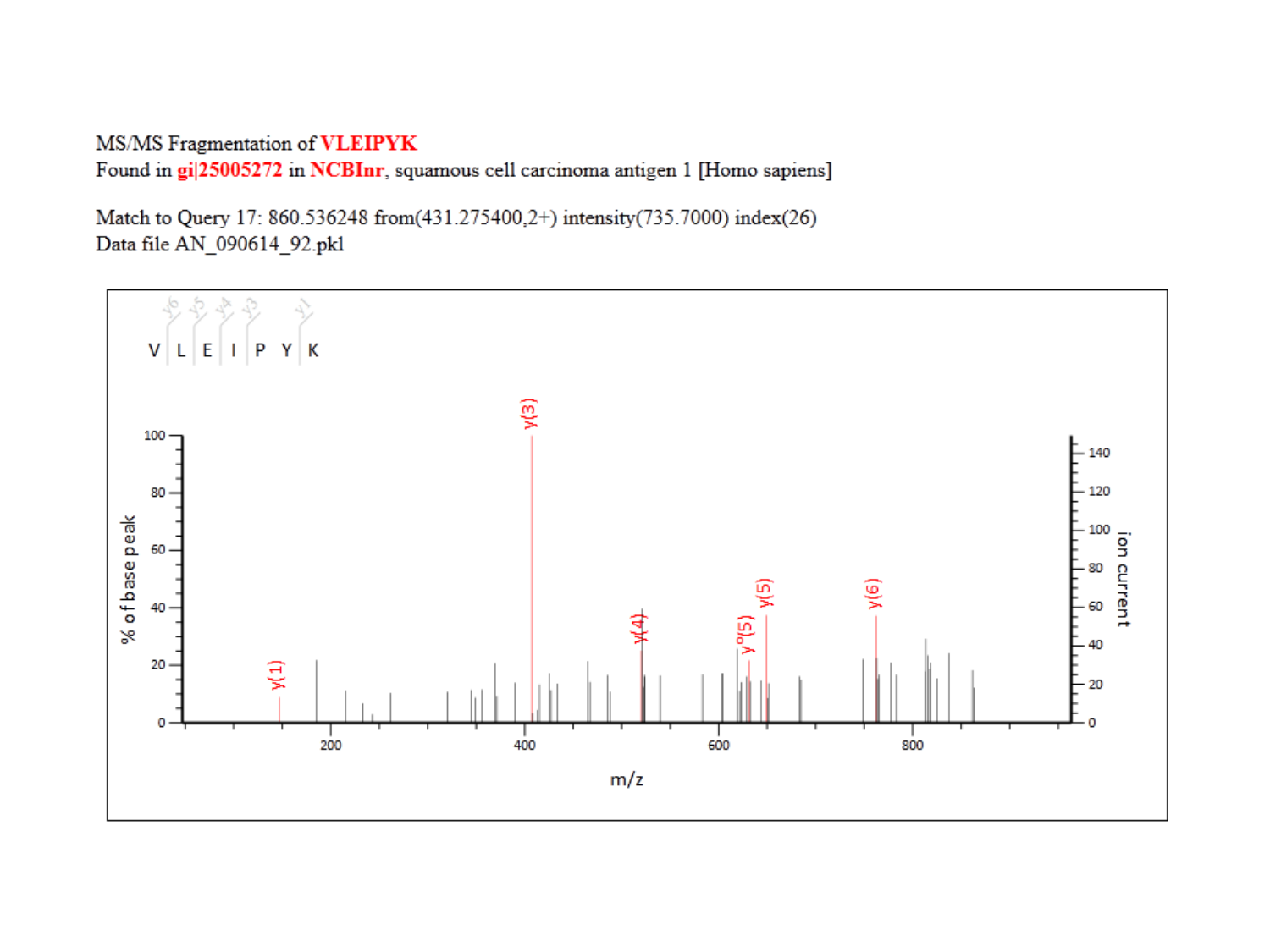

## Slide 28
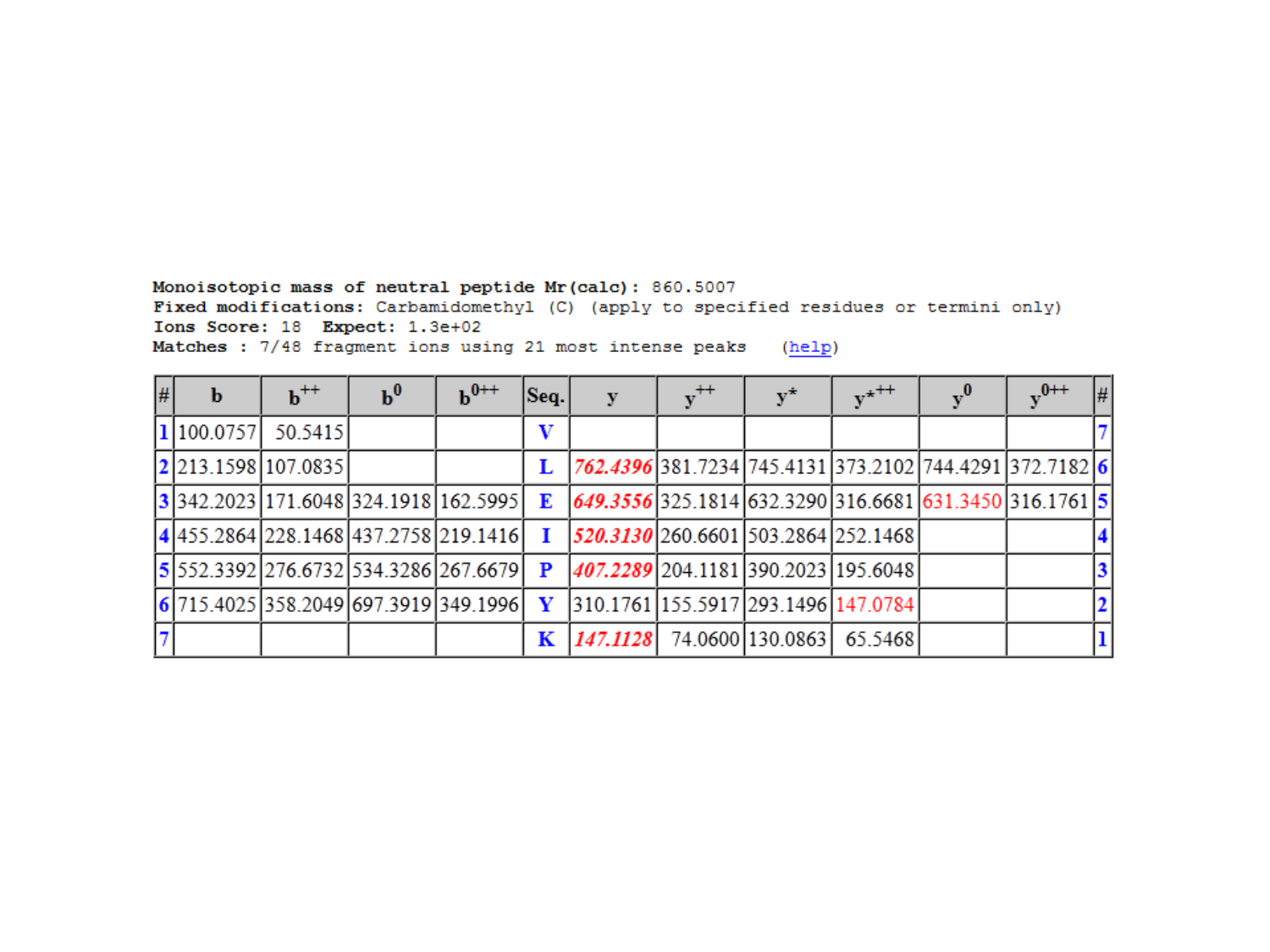

## Slide 29
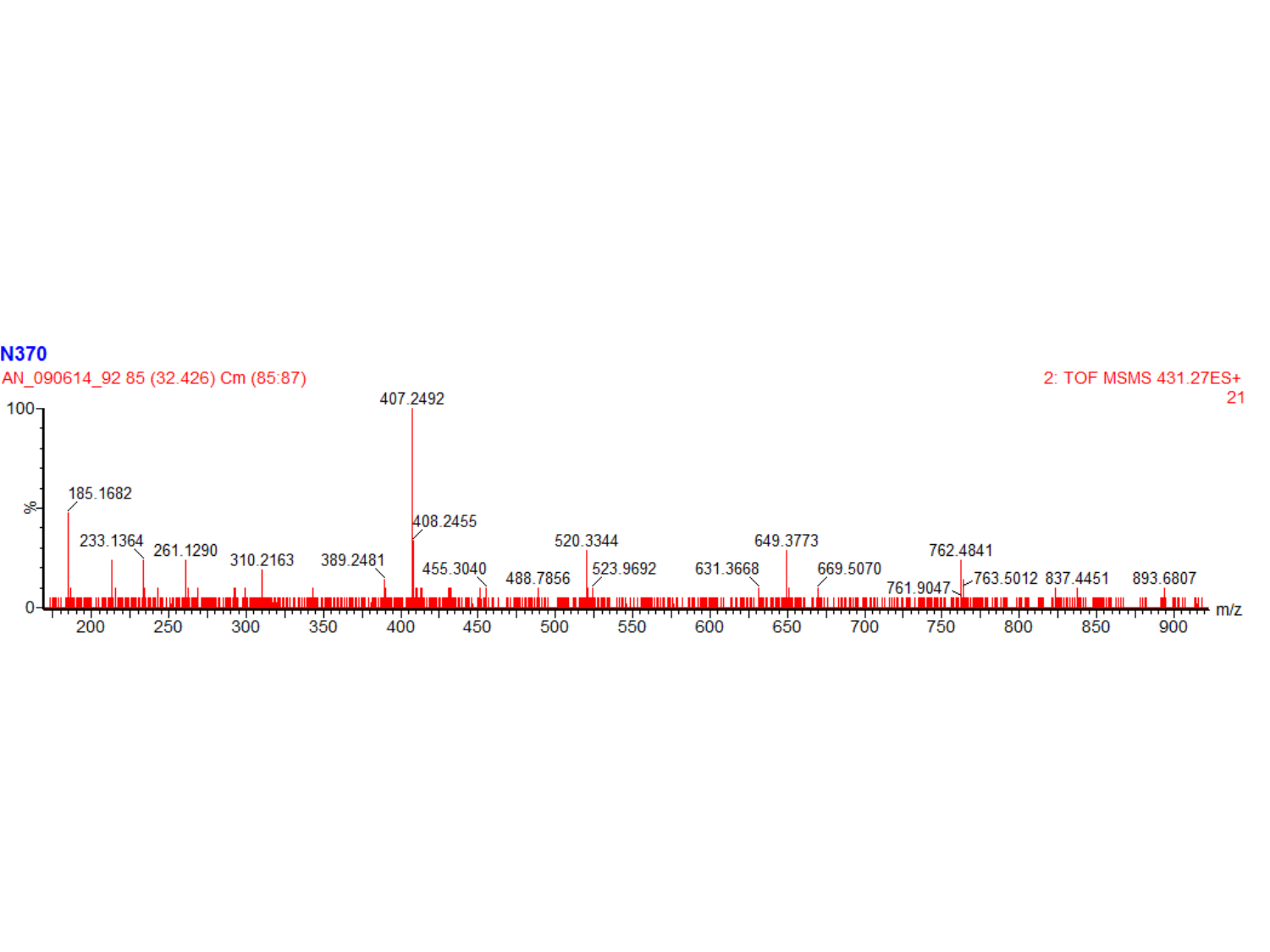

## Slide 30
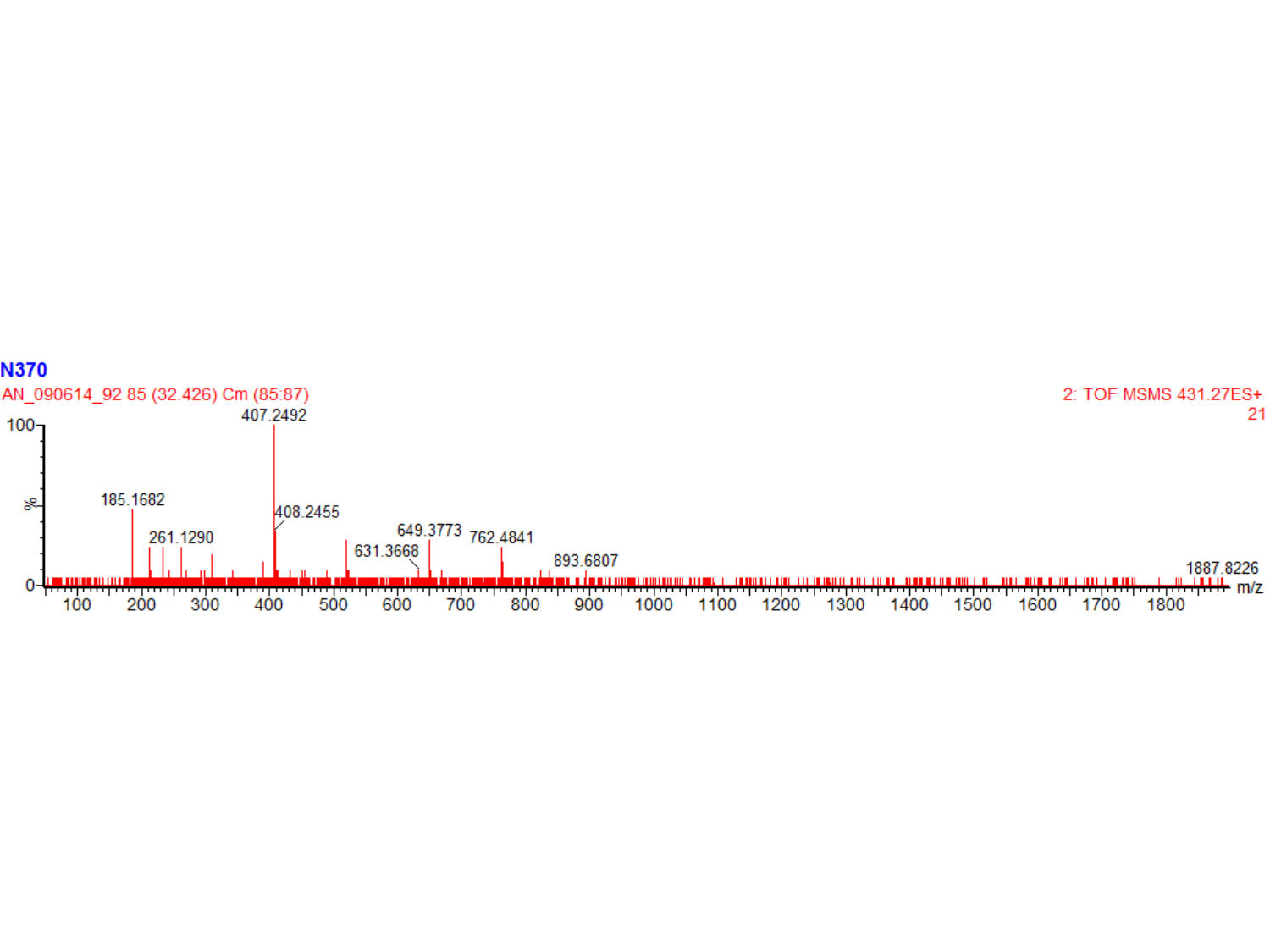

## Slide 31
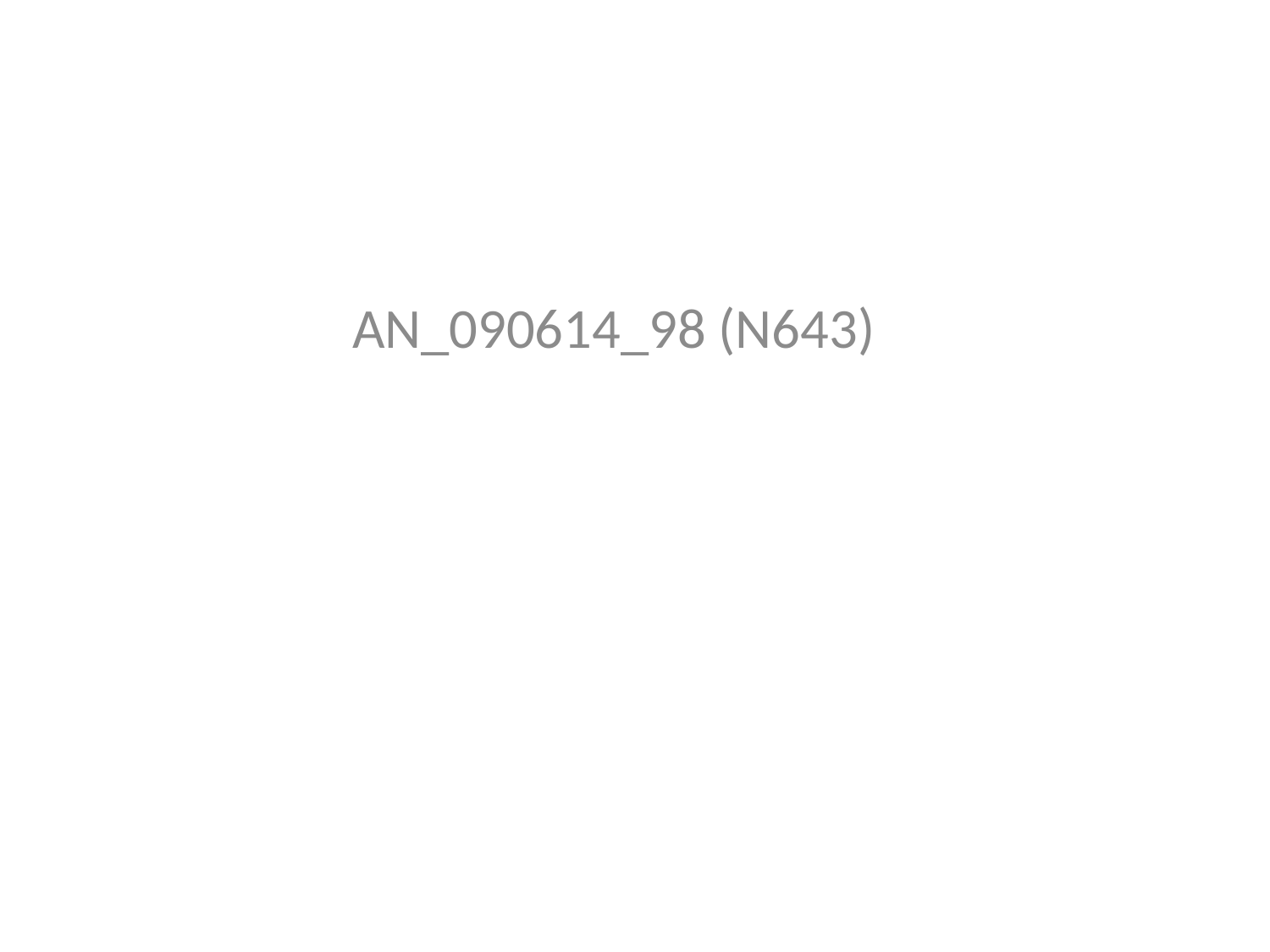

AN_090614_98 (N643)

## Slide 32
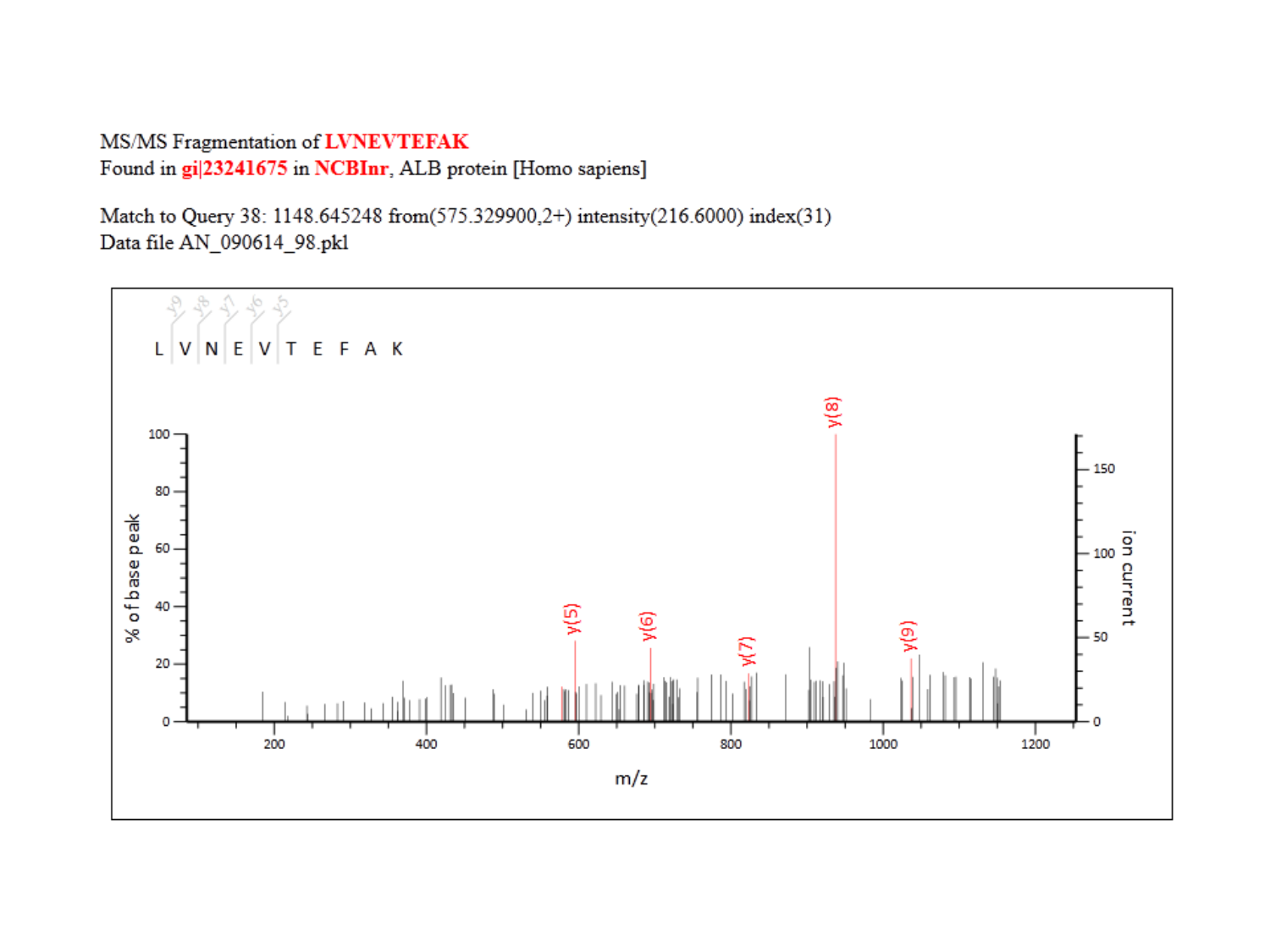

## Slide 33
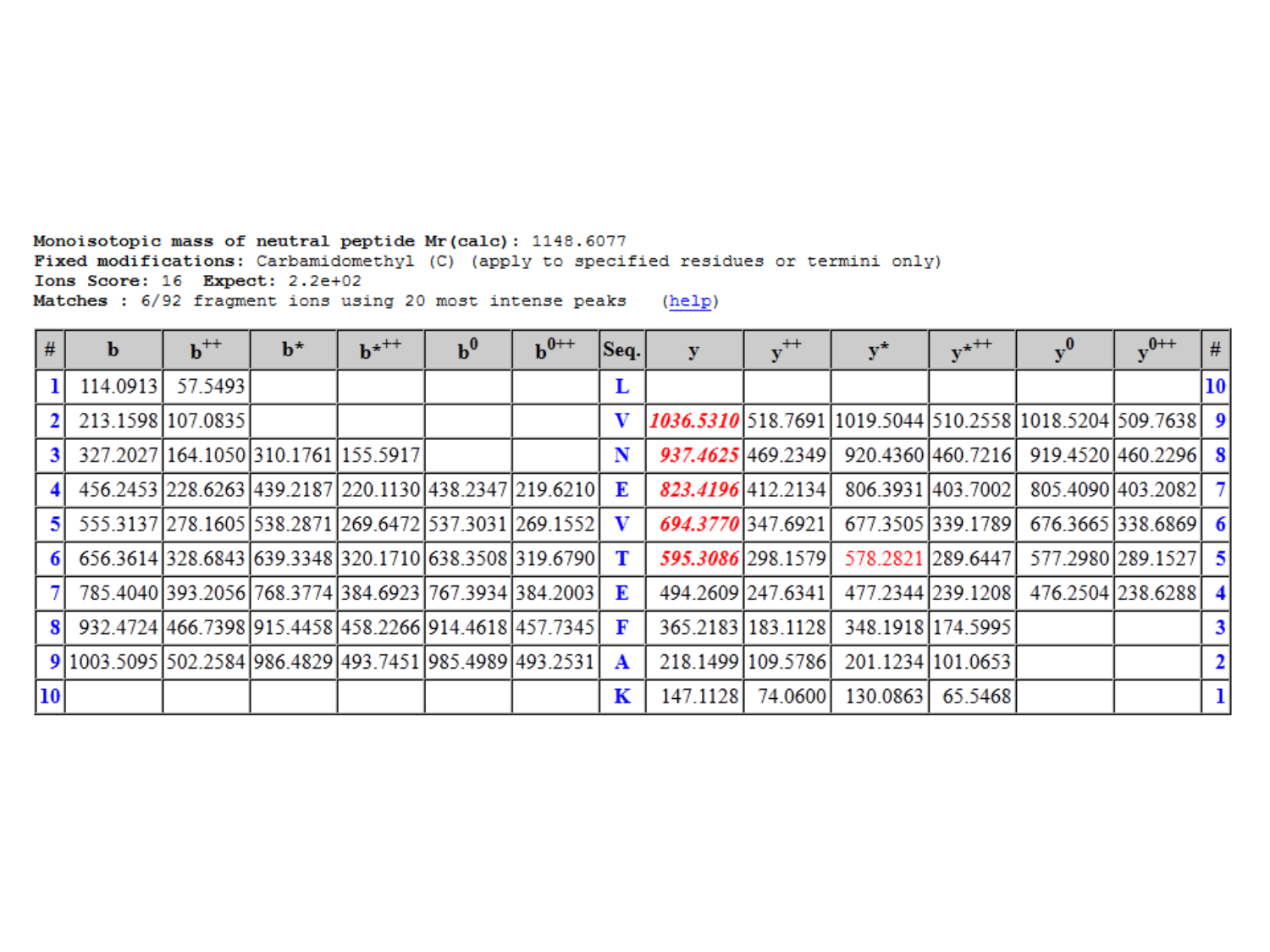

## Slide 34
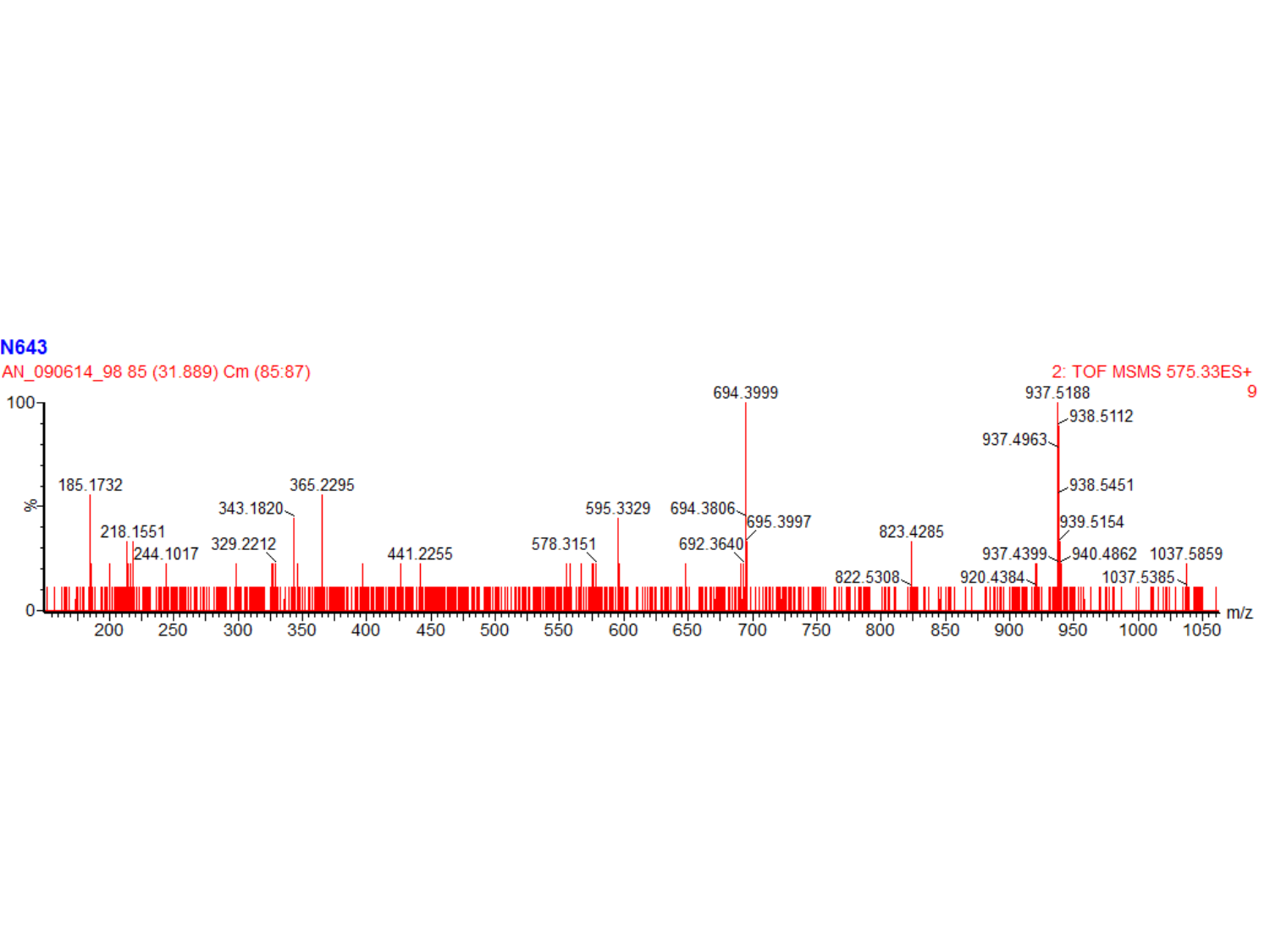

## Slide 35
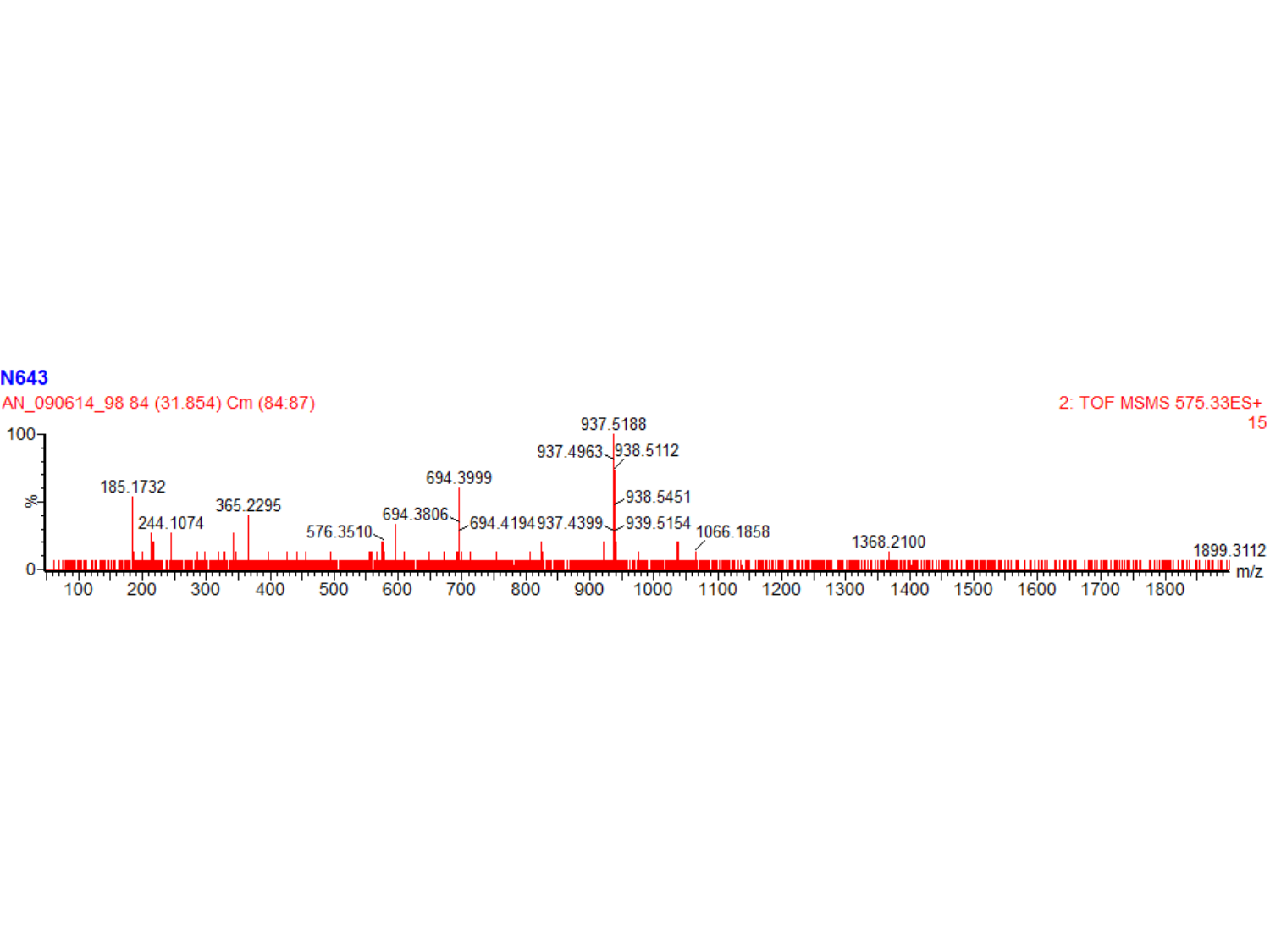

## Slide 36
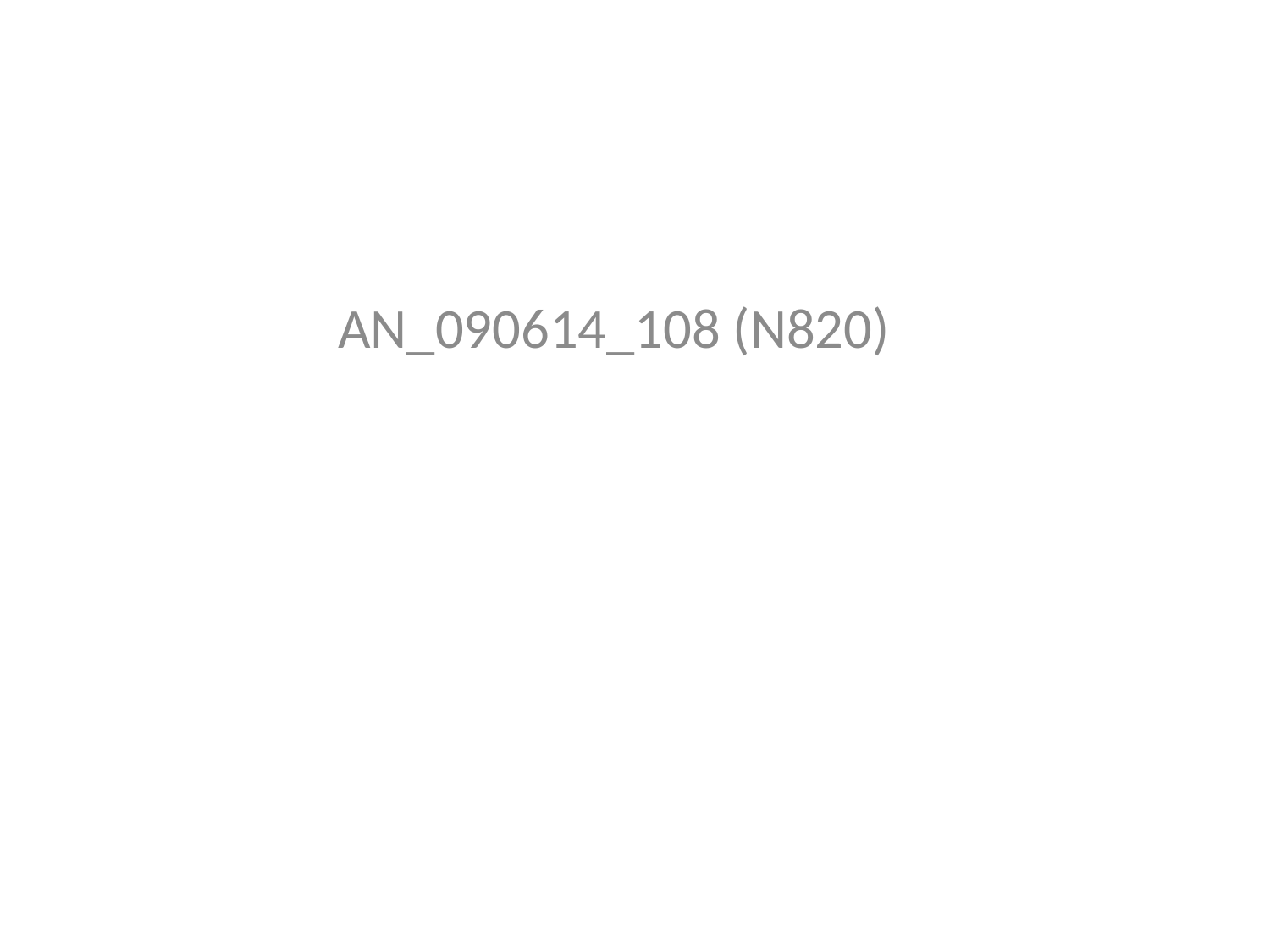

AN_090614_108 (N820)

## Slide 37
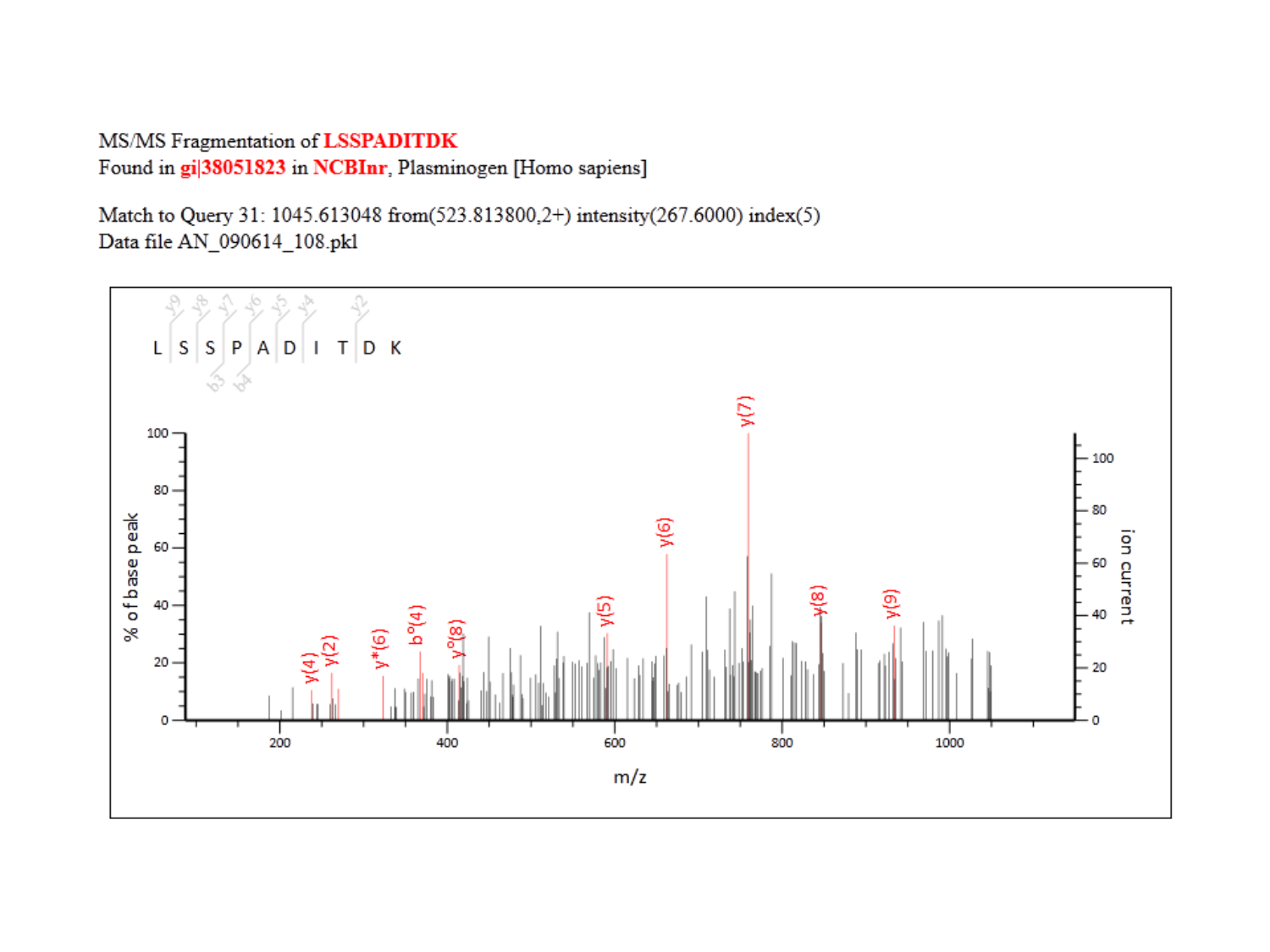

## Slide 38
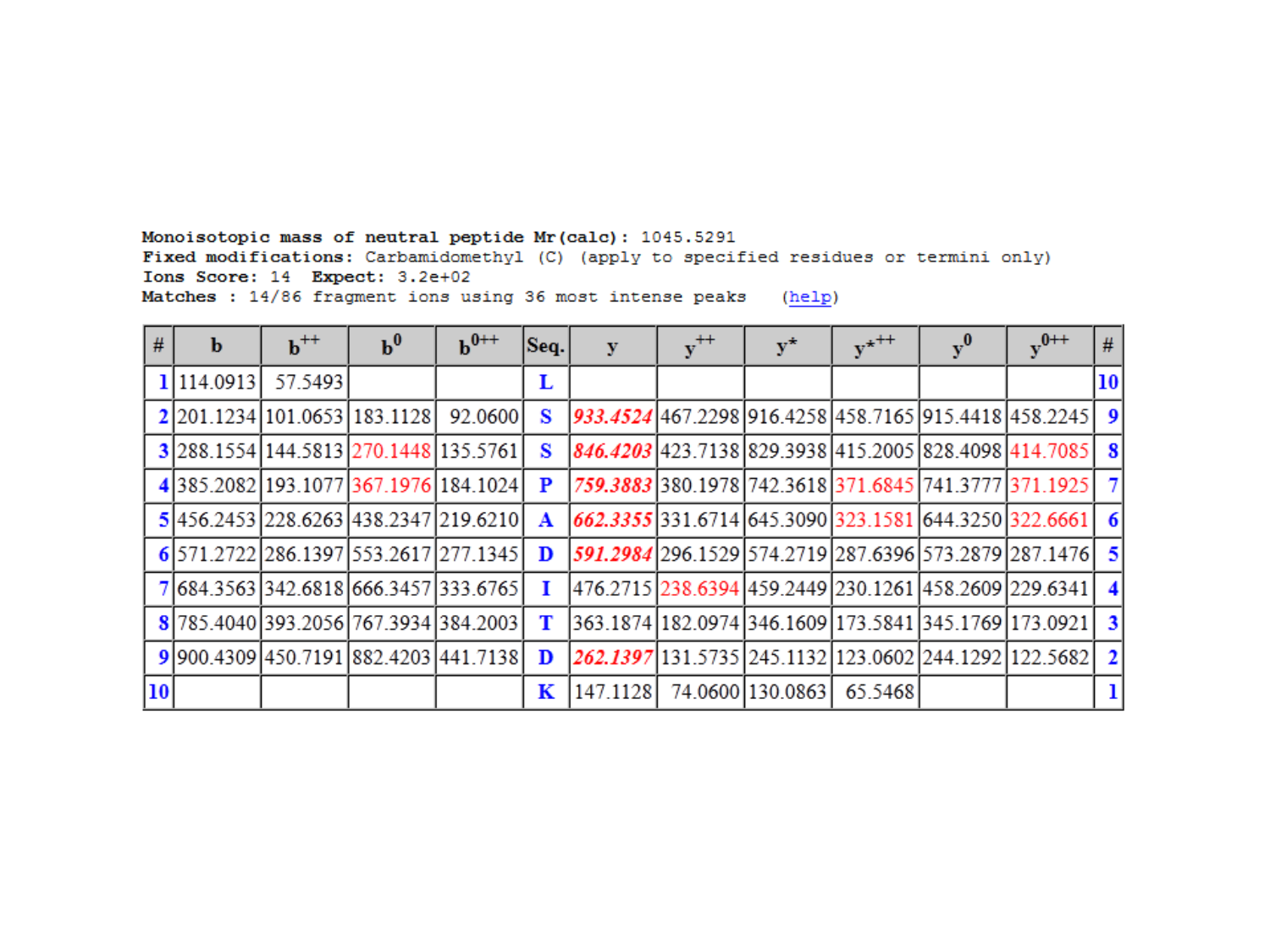

## Slide 39
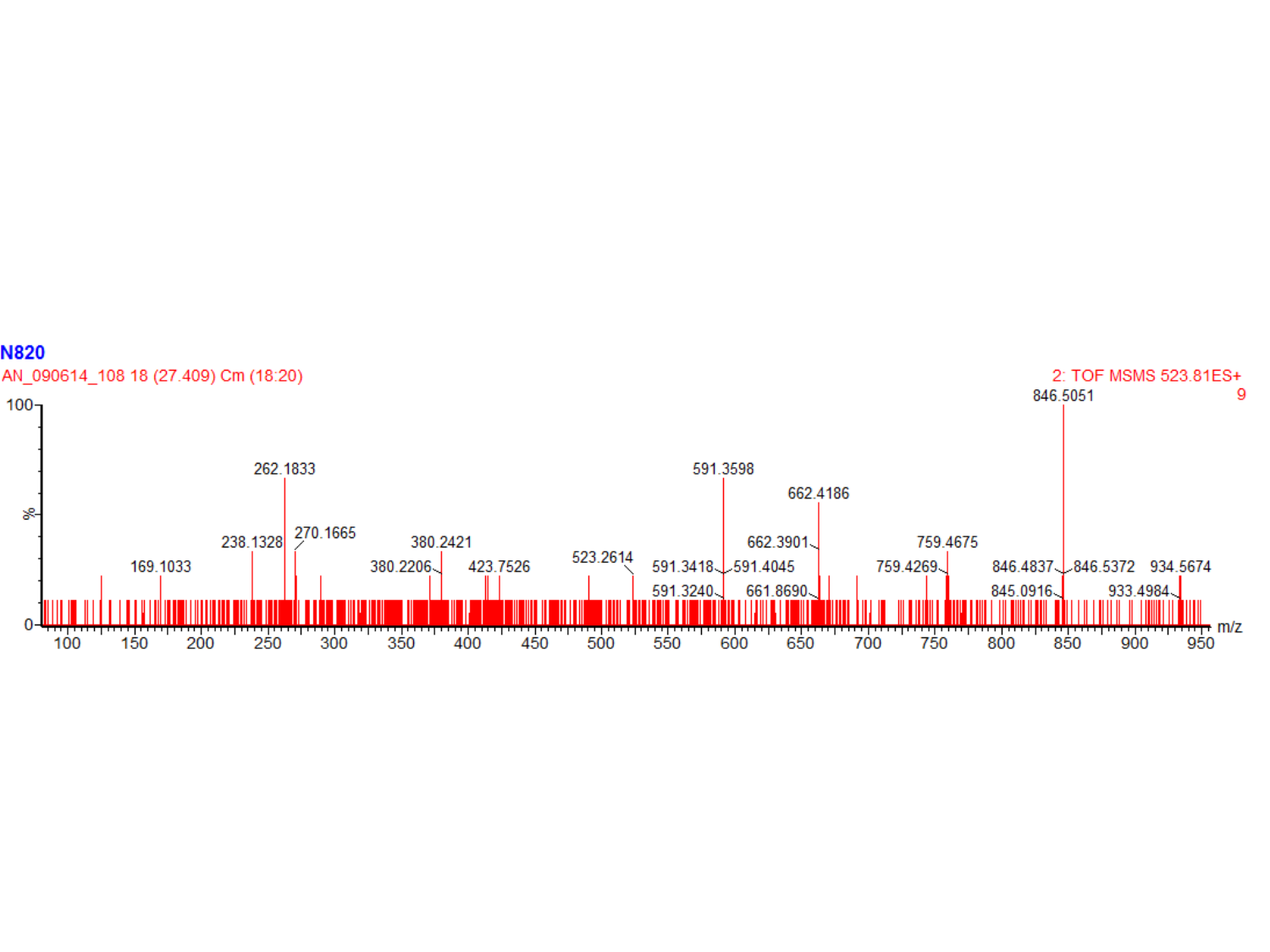

## Slide 40
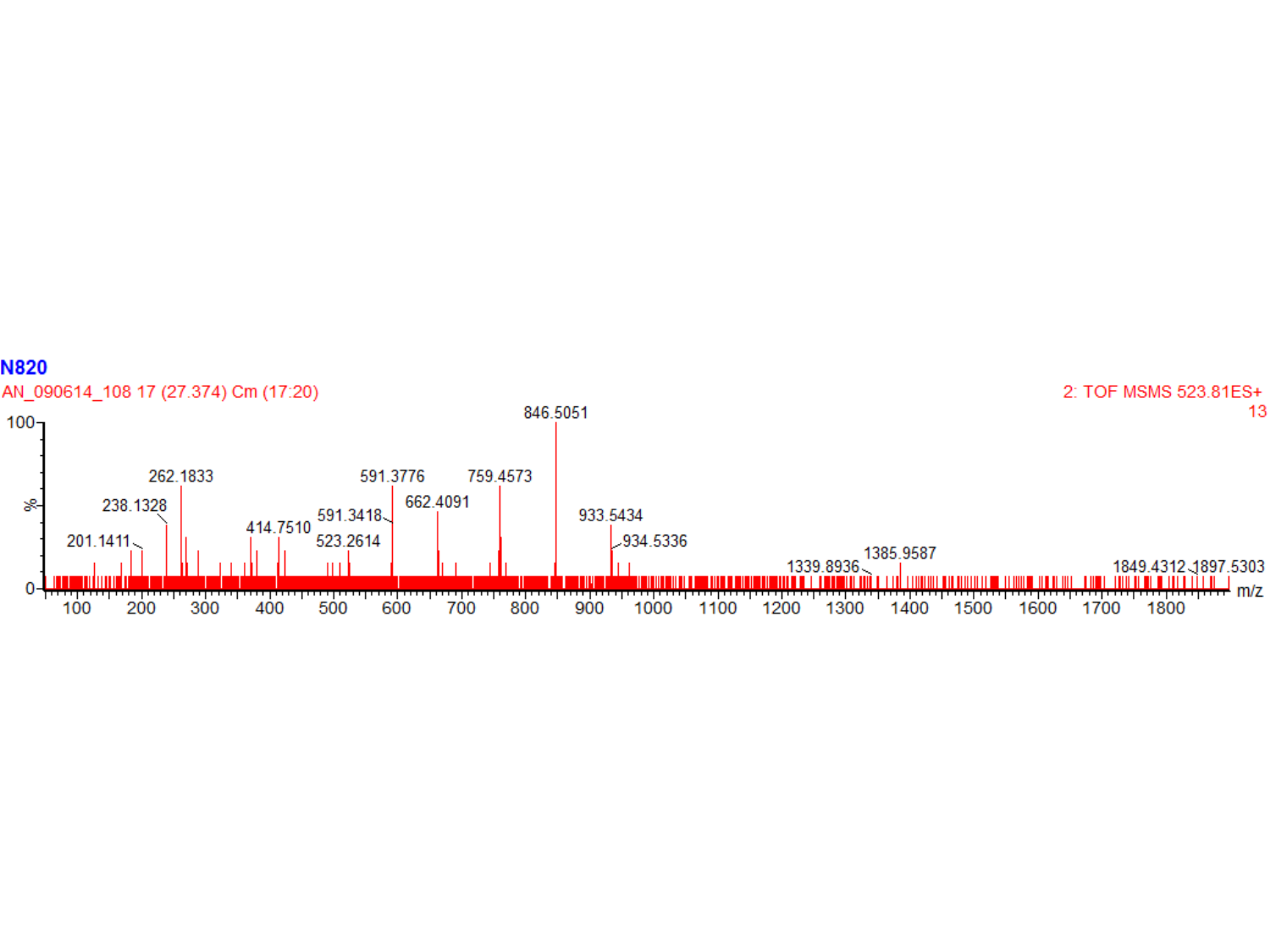

## Slide 41
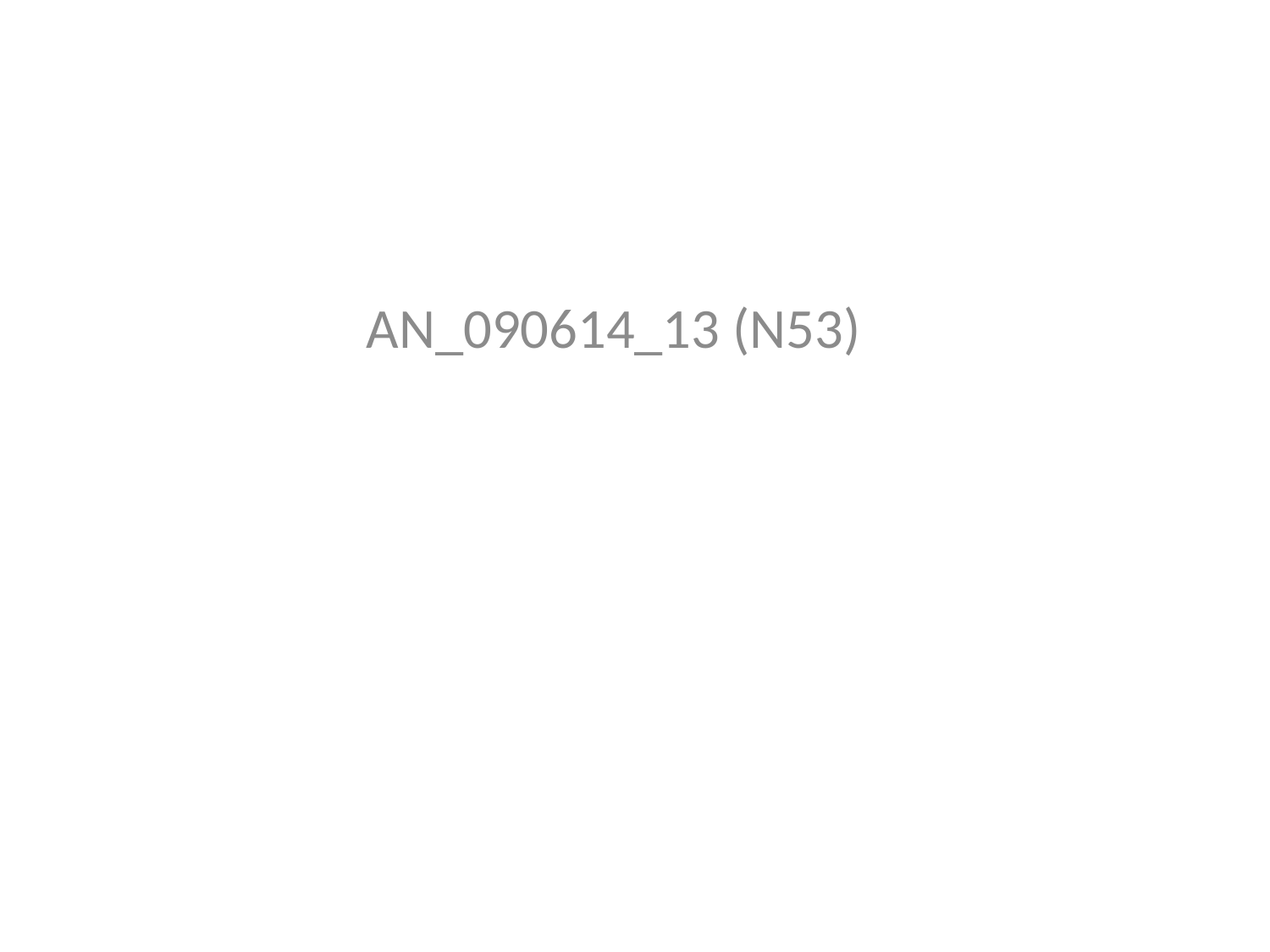

AN_090614_13 (N53)

## Slide 42
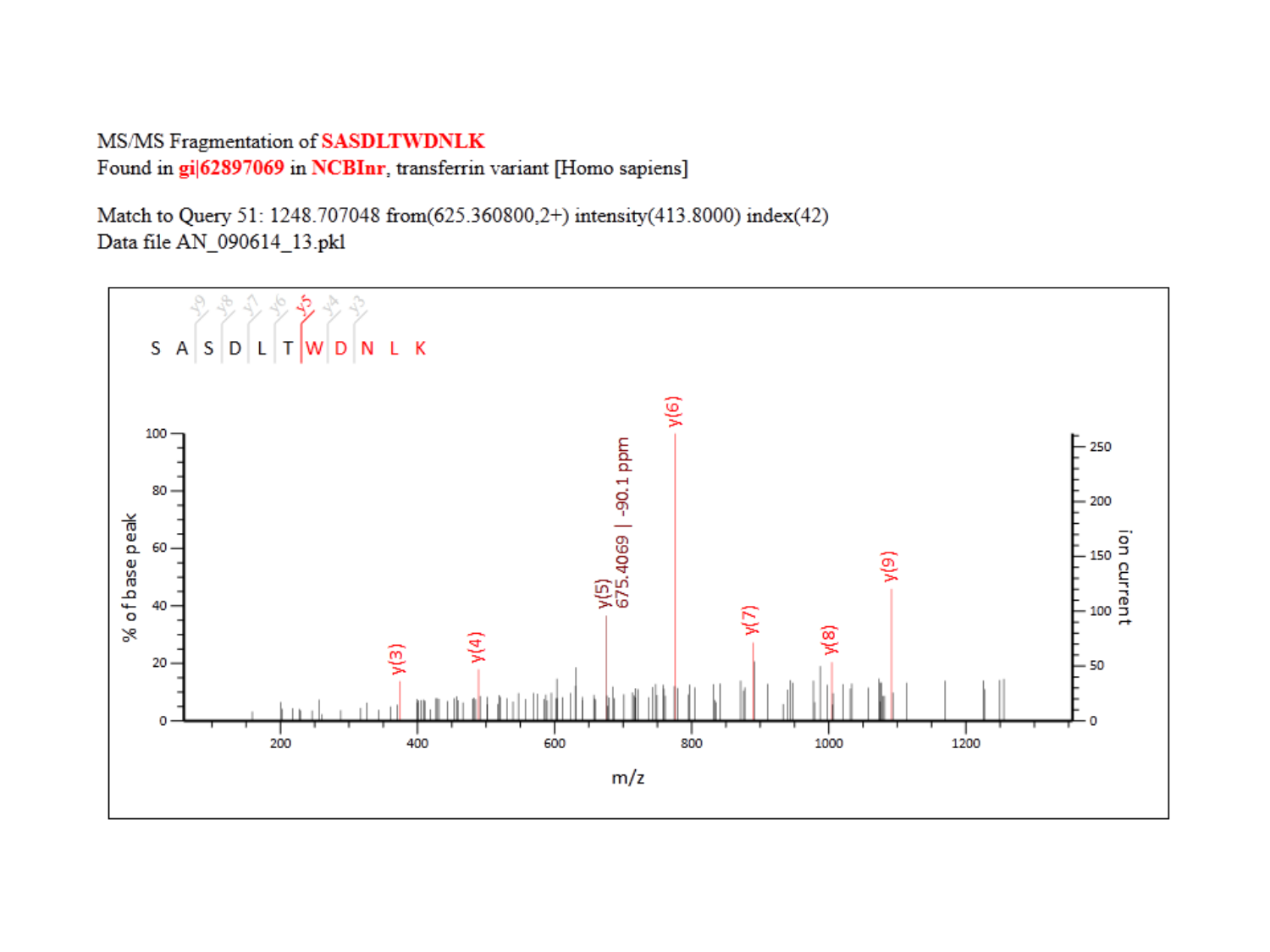

## Slide 43
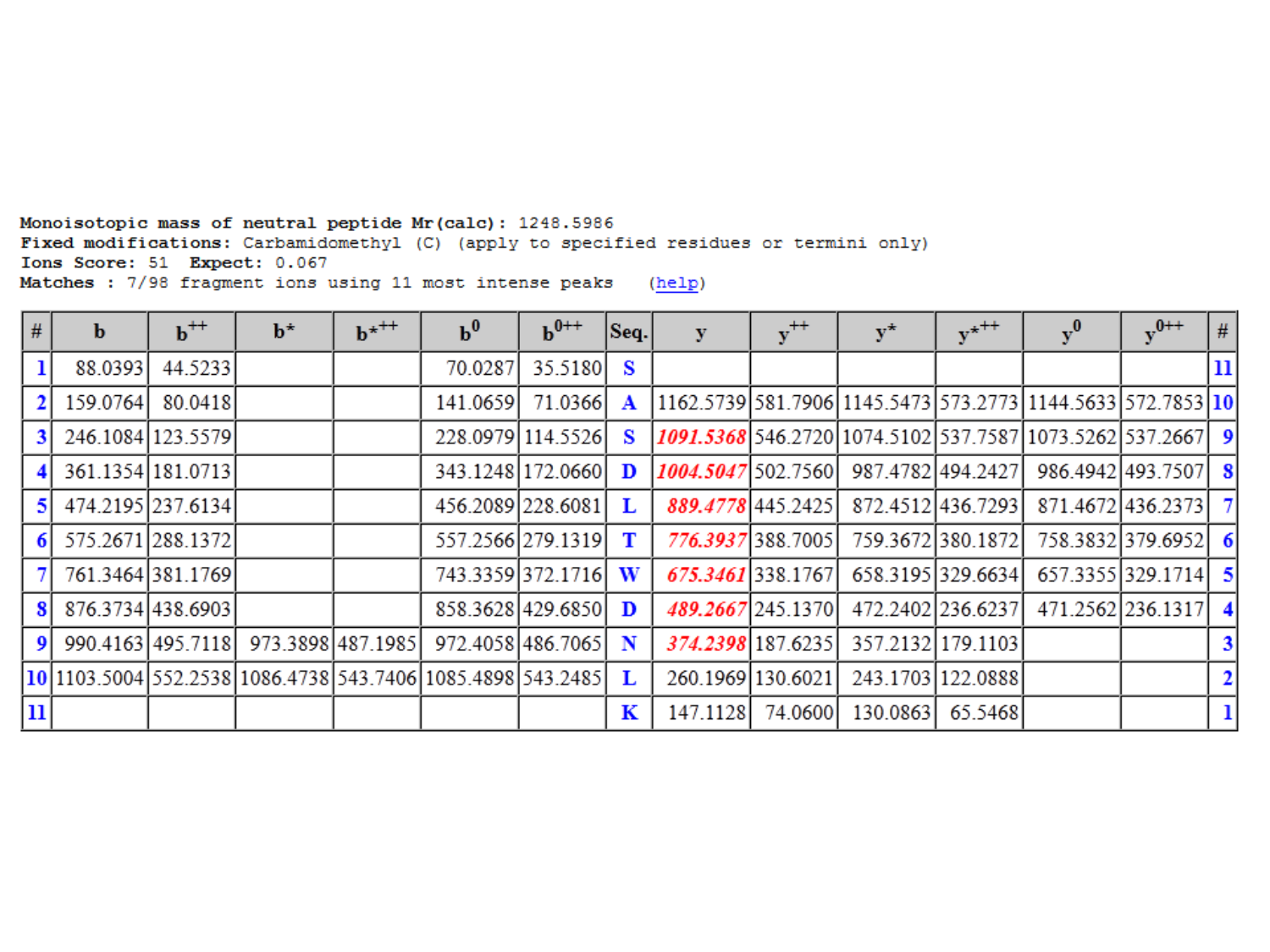

## Slide 44
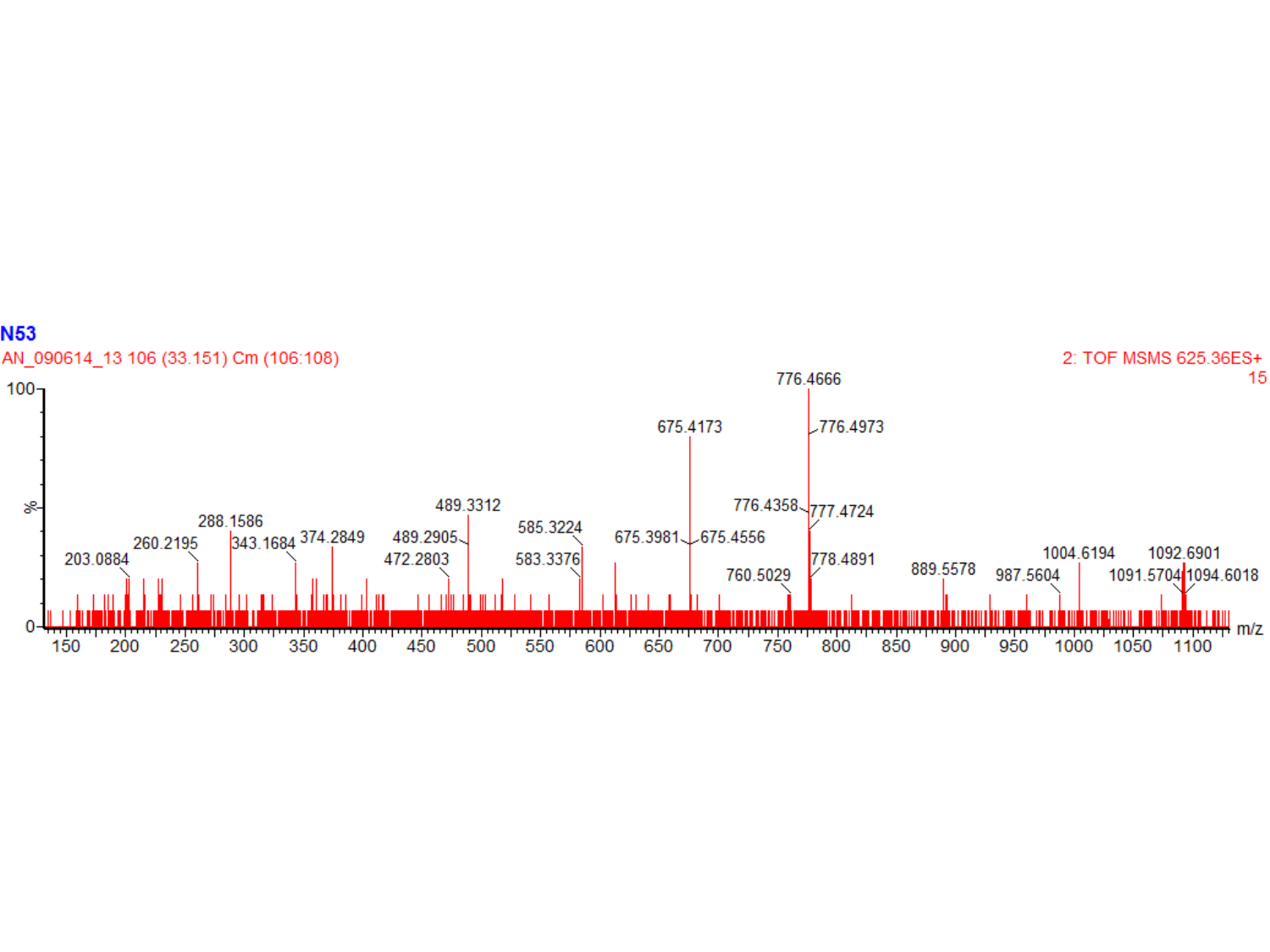

## Slide 45
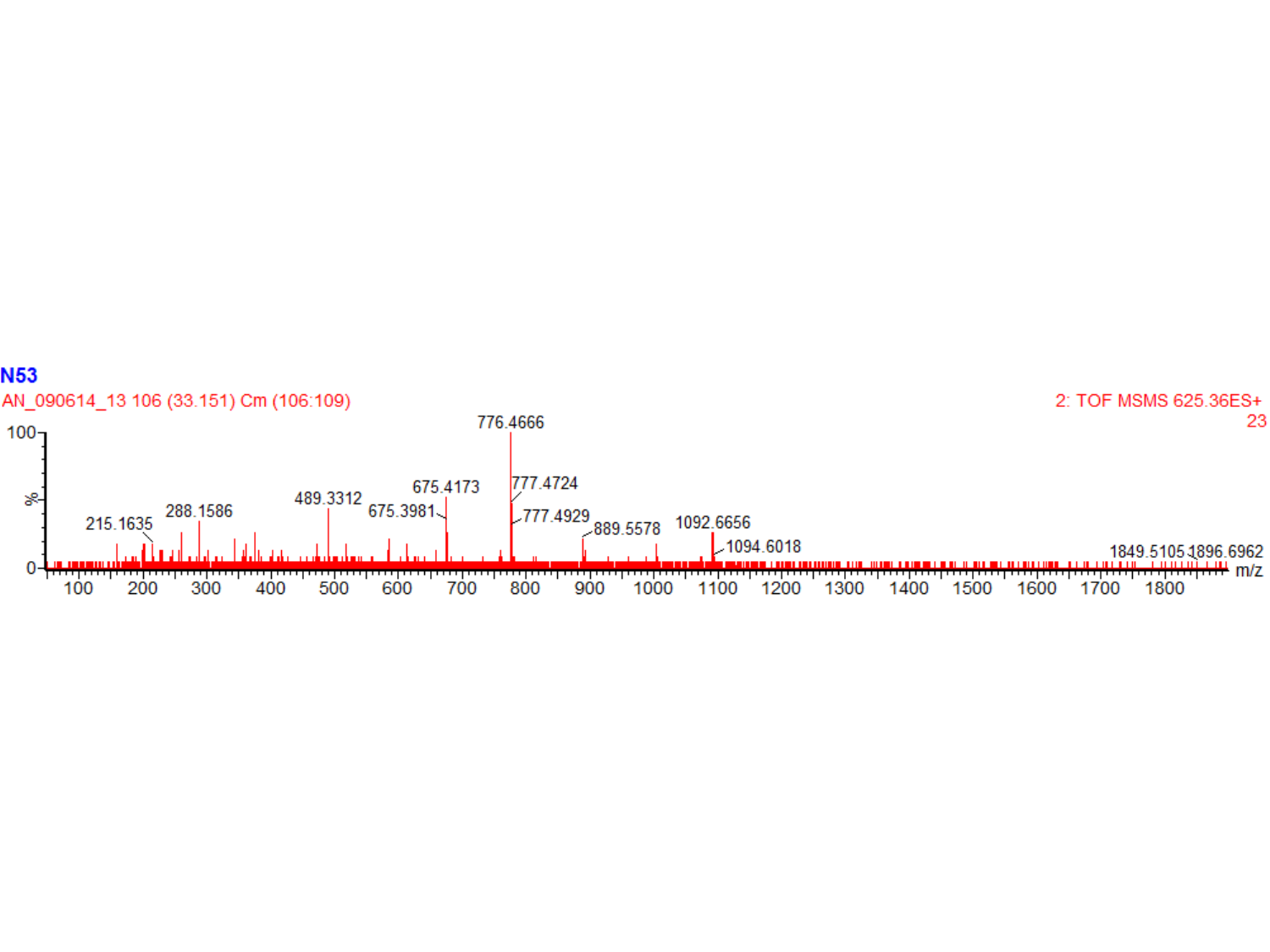

## Slide 46
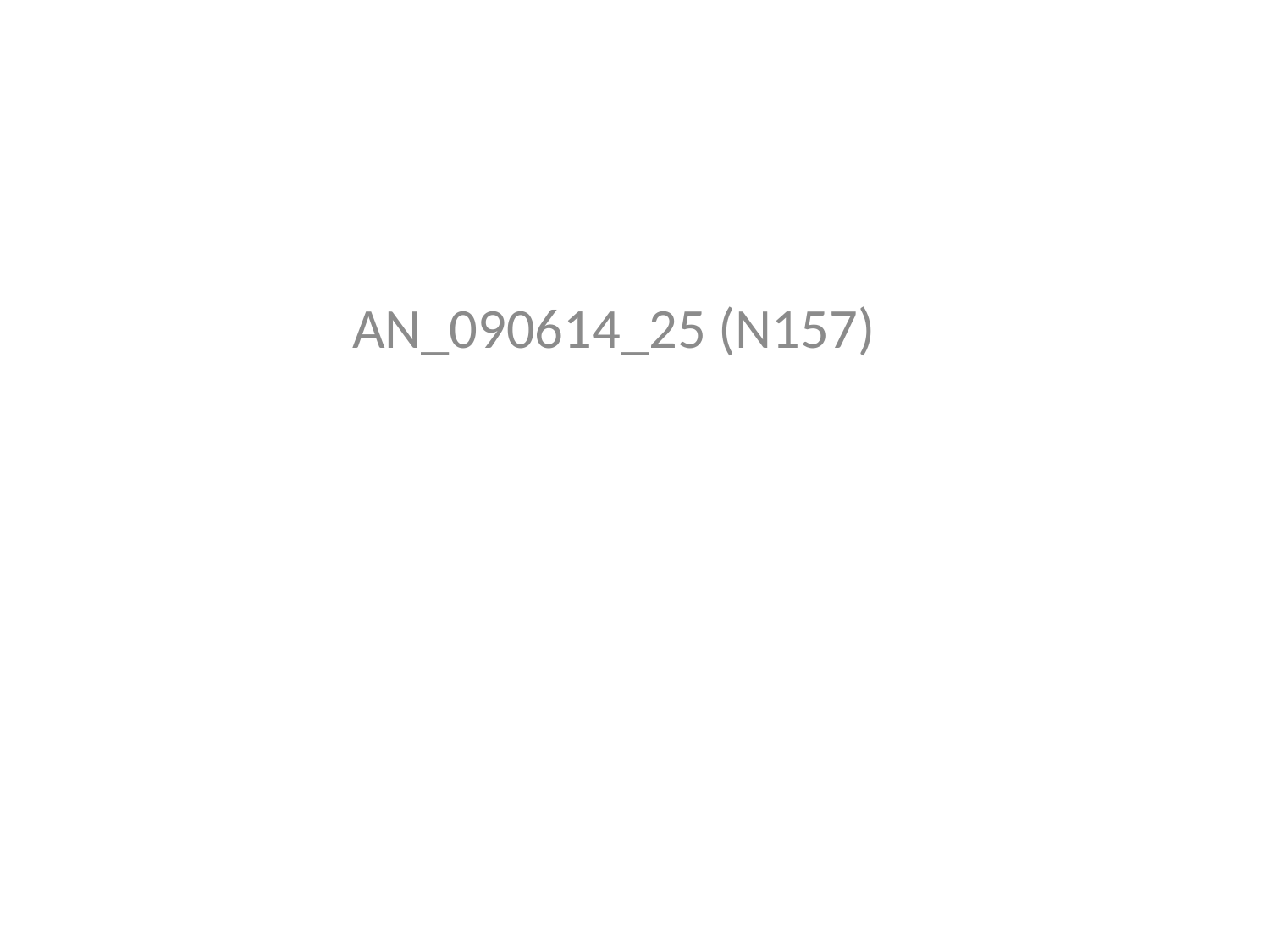

AN_090614_25 (N157)

## Slide 47
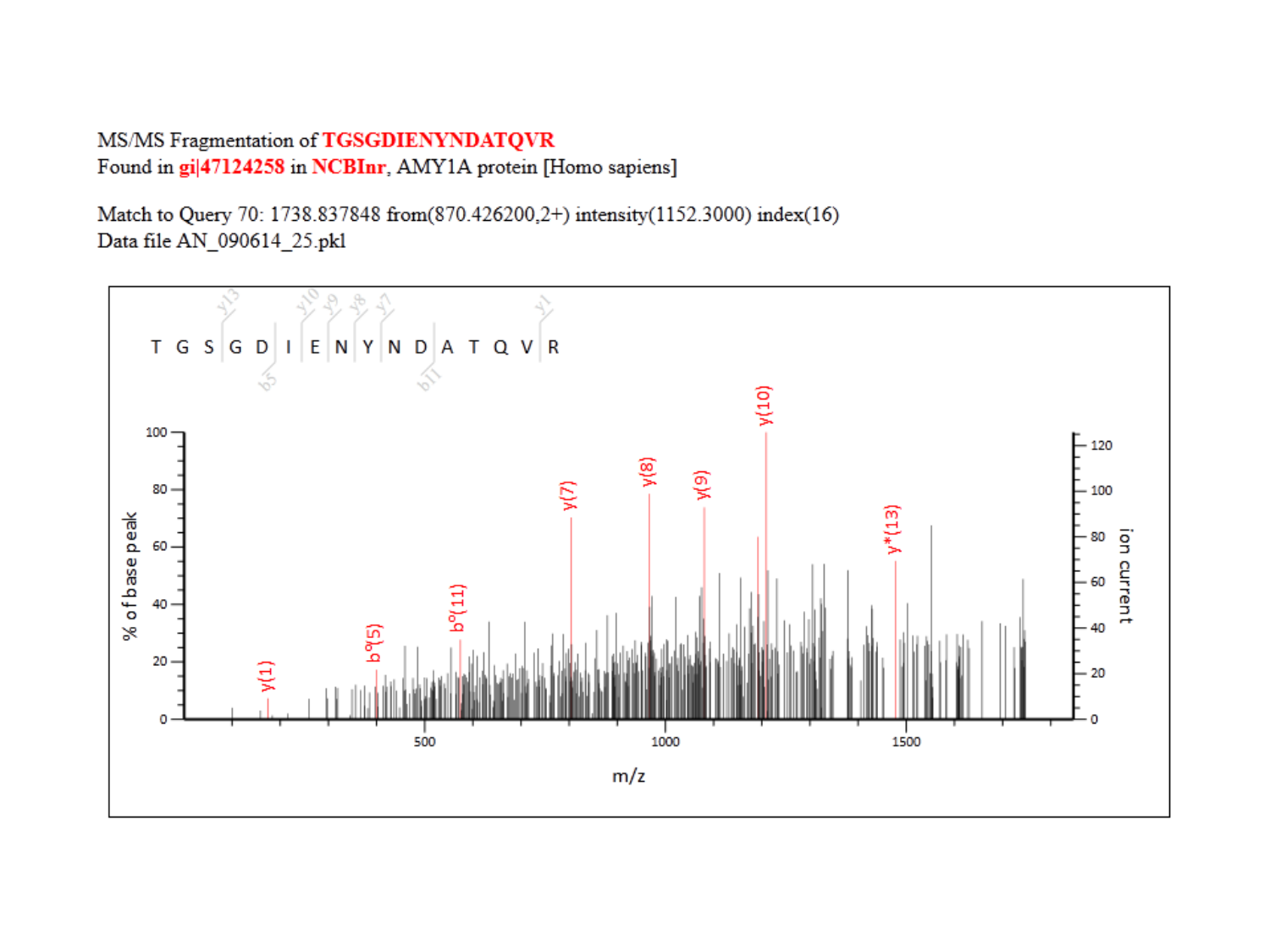

## Slide 48
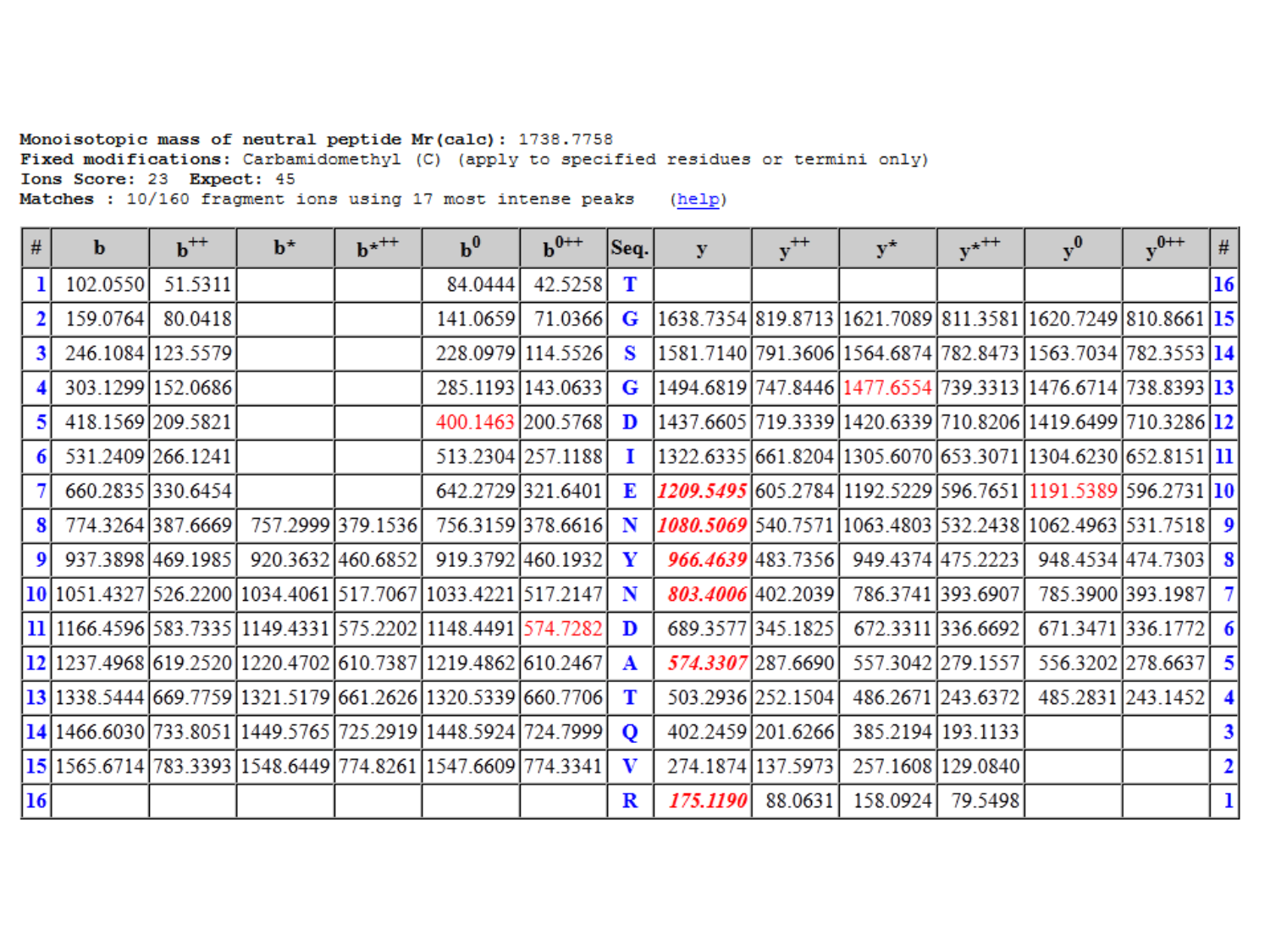

## Slide 49
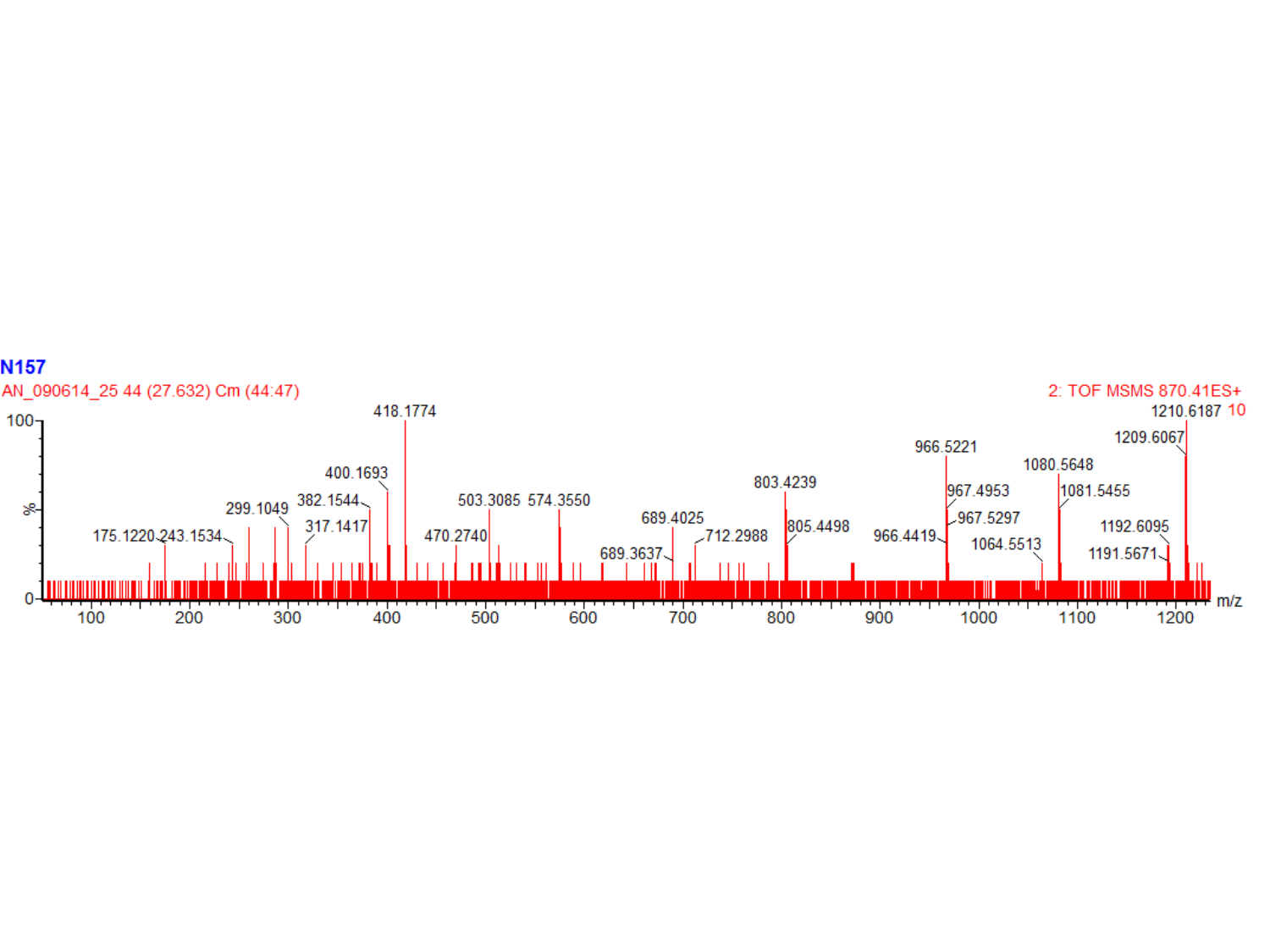

## Slide 50
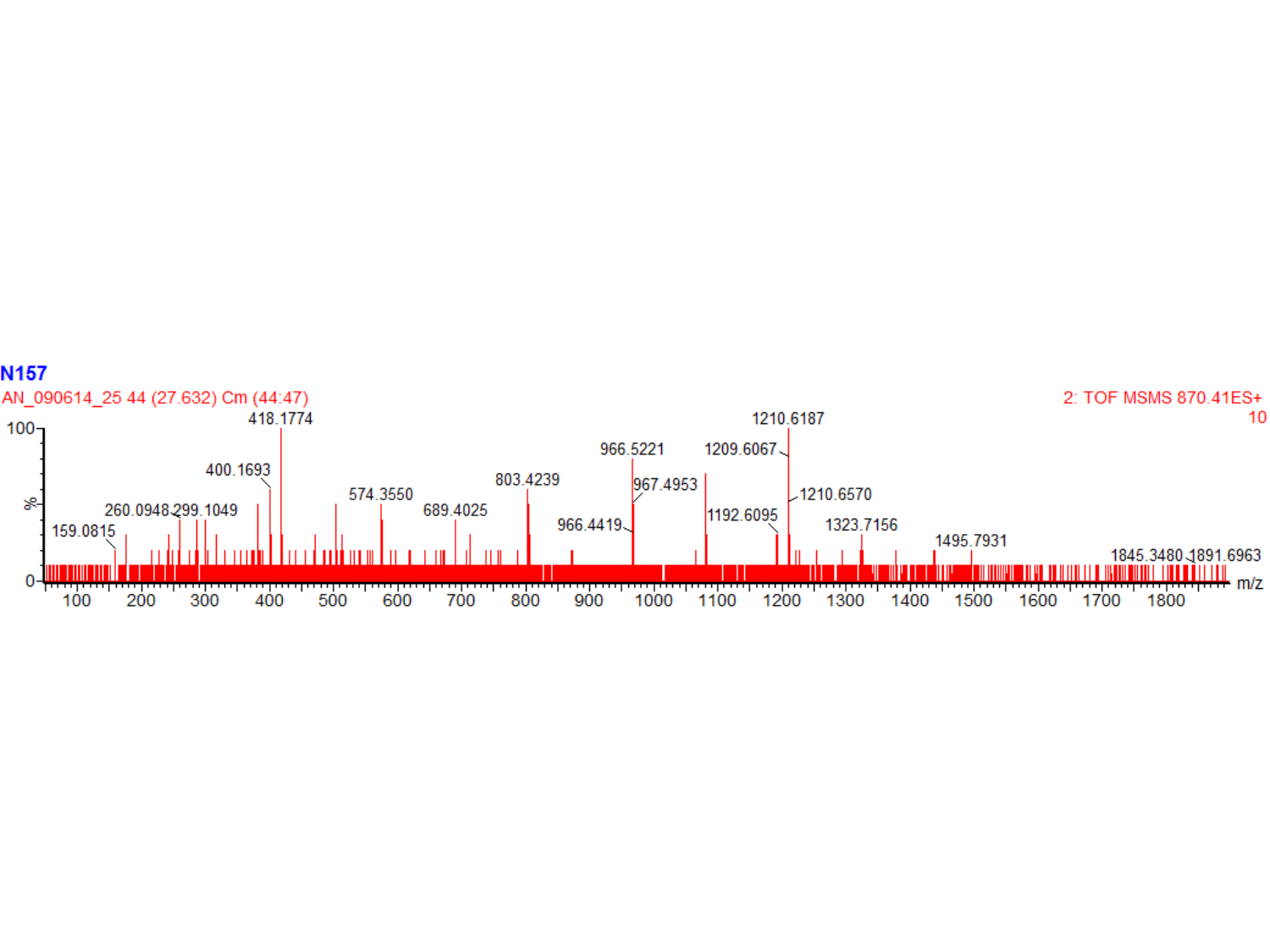

## Slide 51
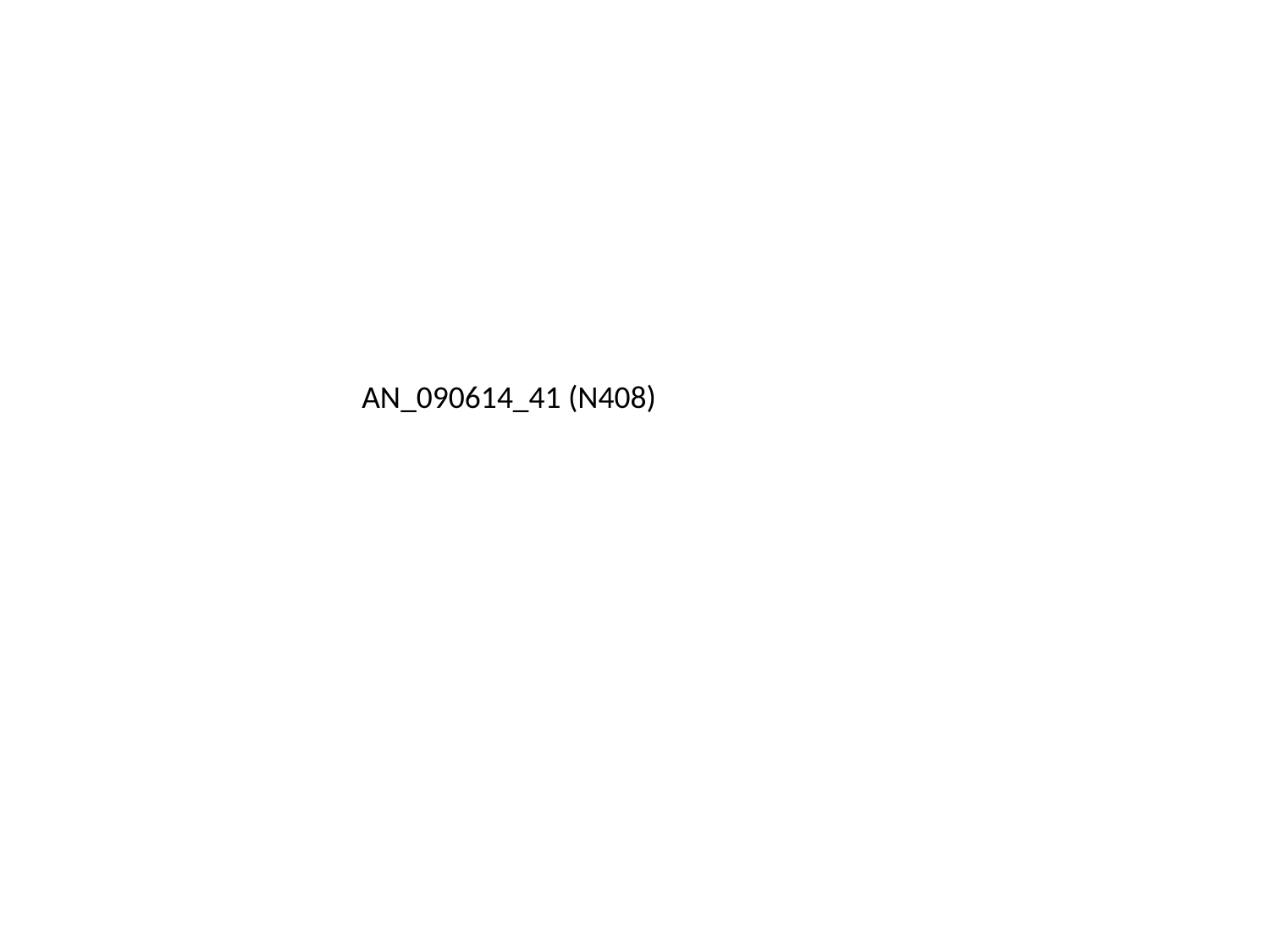

AN_090614_41 (N408)

## Slide 52
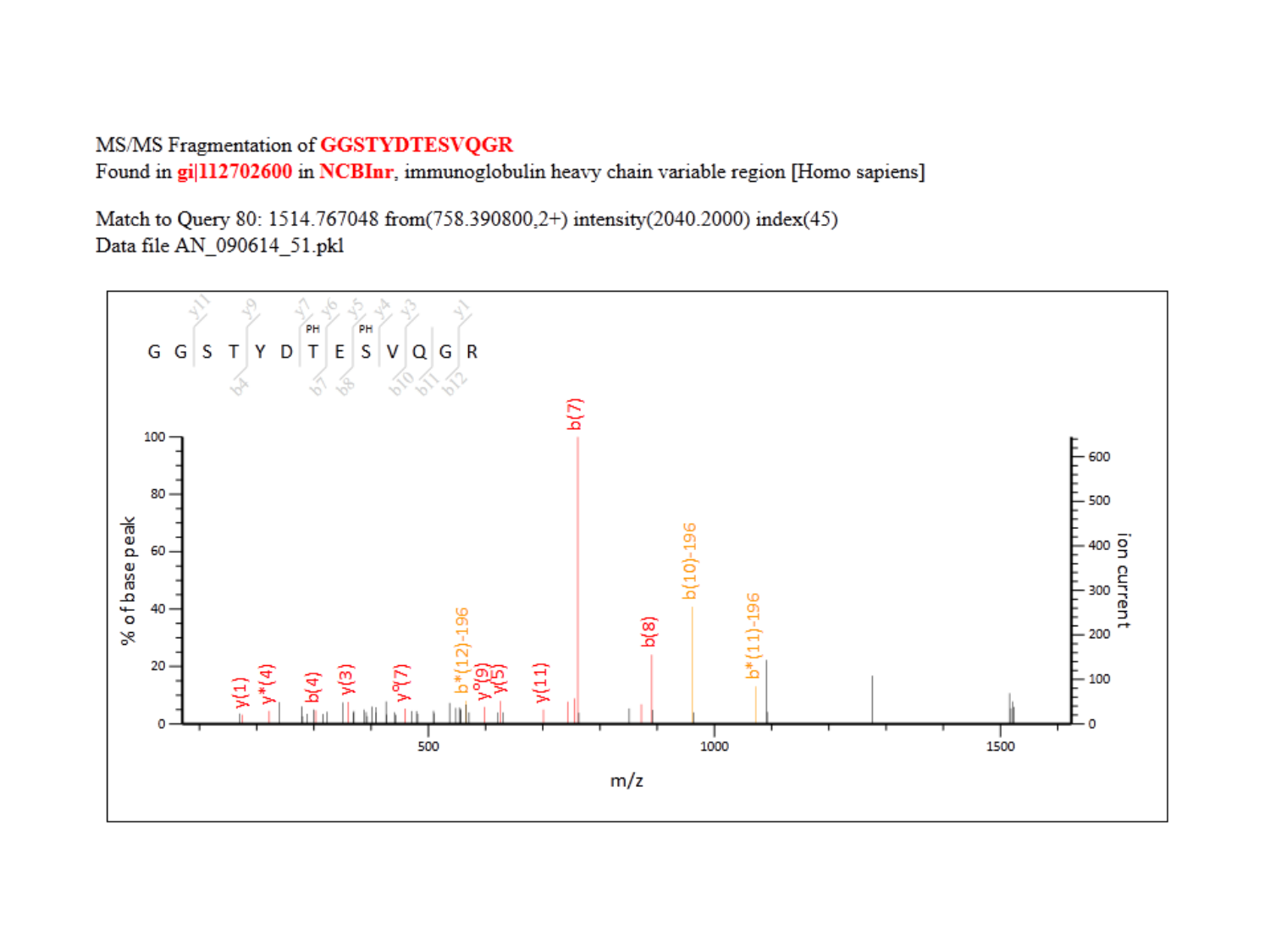

## Slide 53
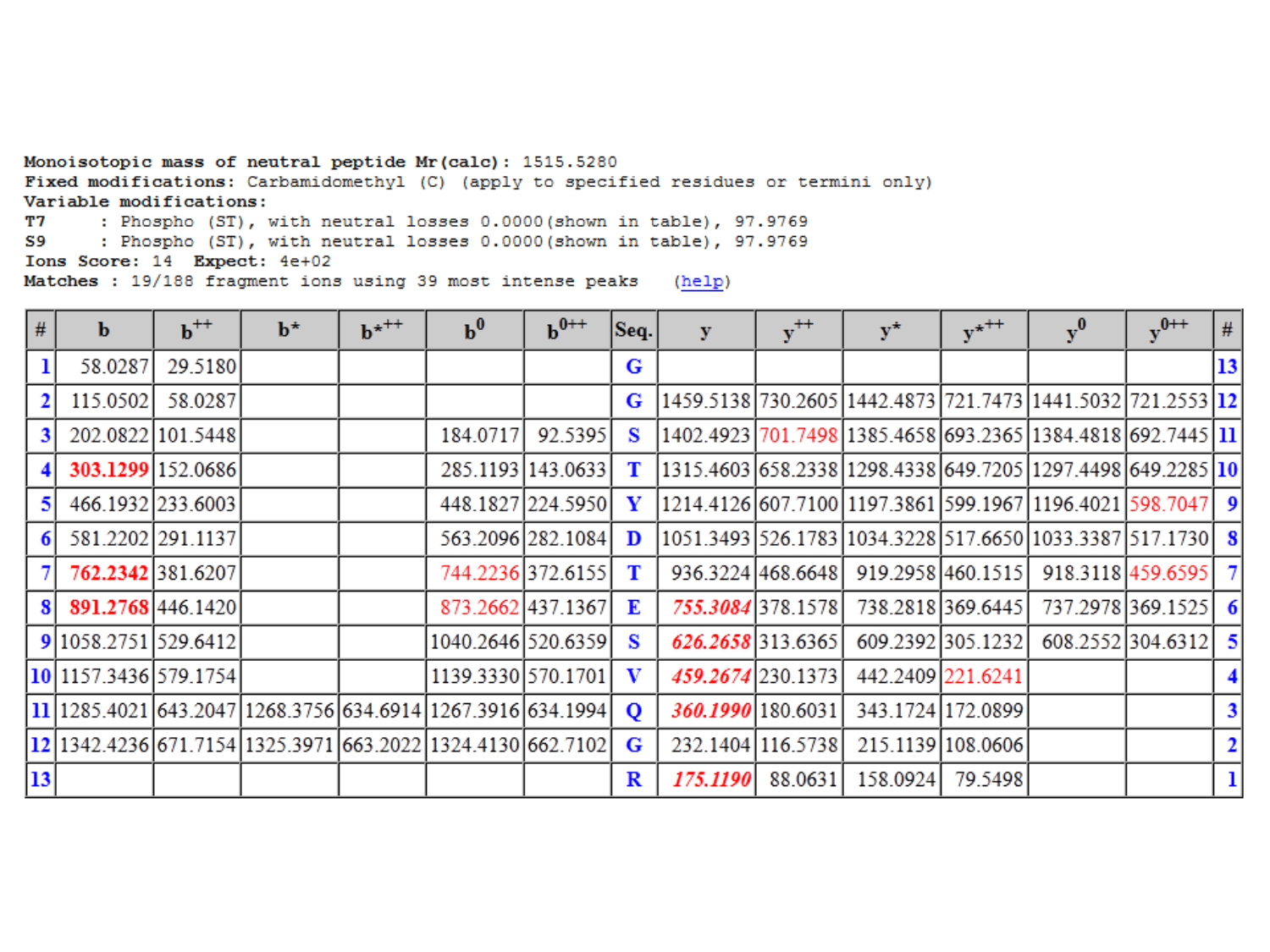

## Slide 54
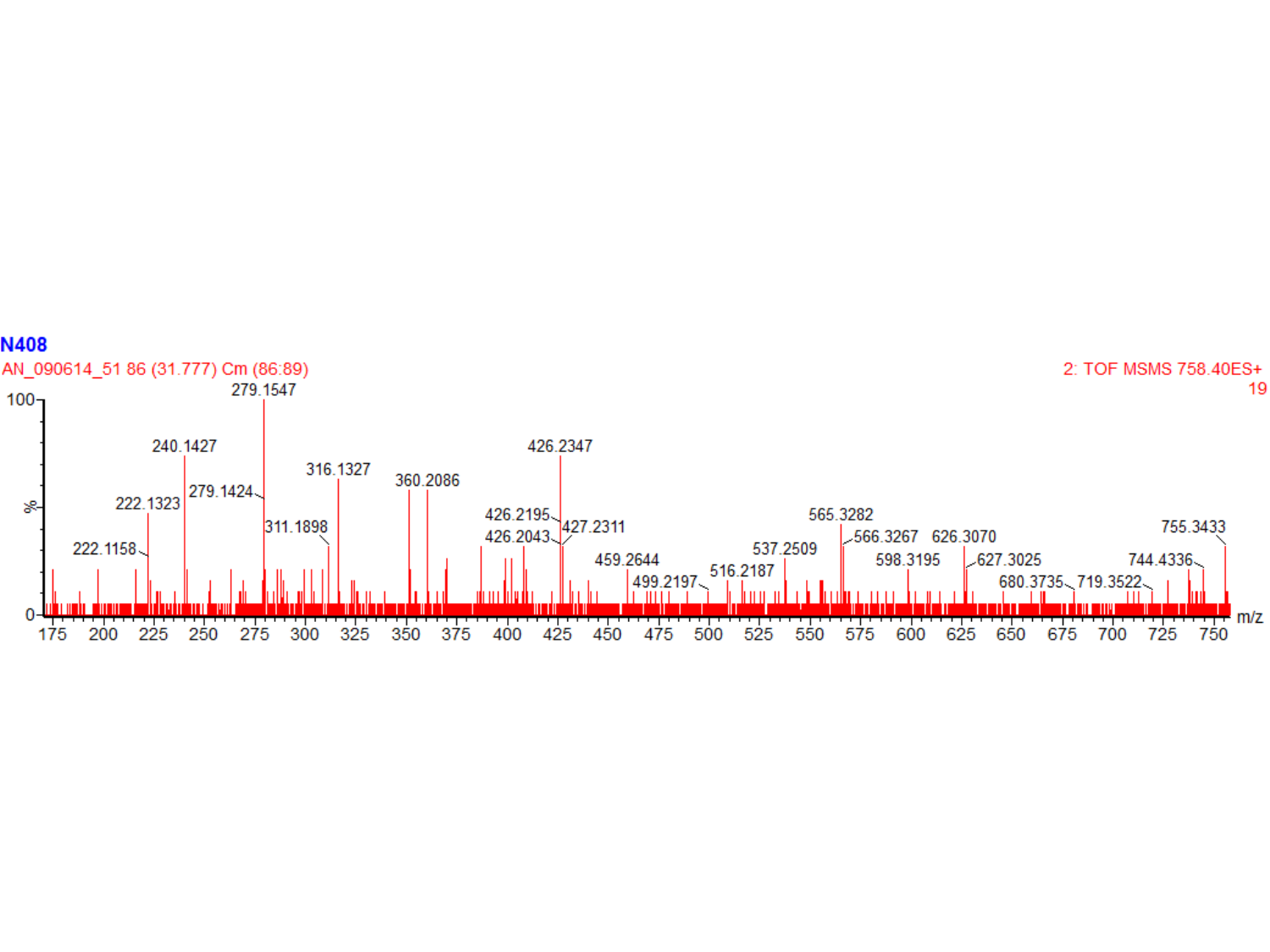

## Slide 55
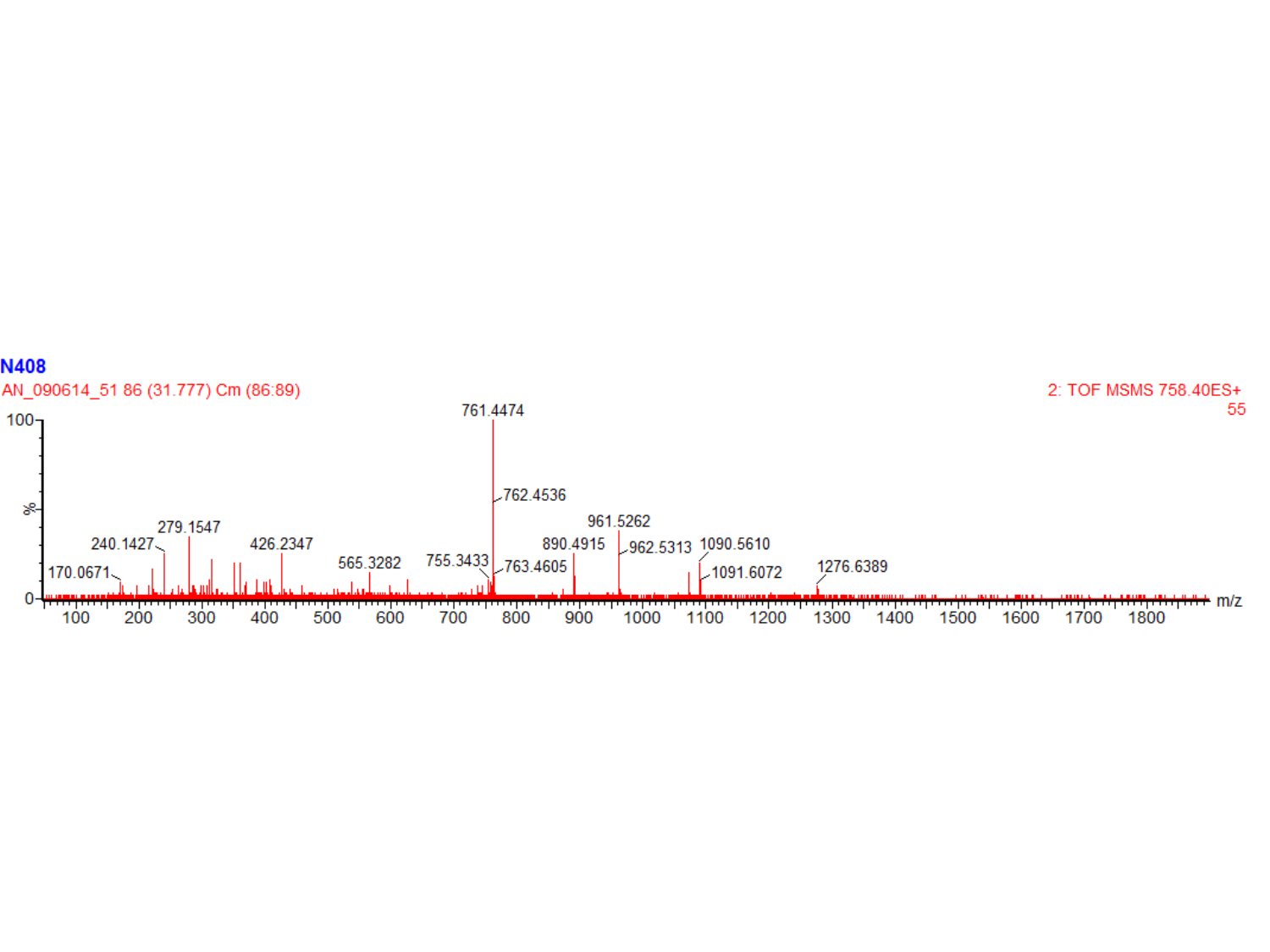

## Slide 56
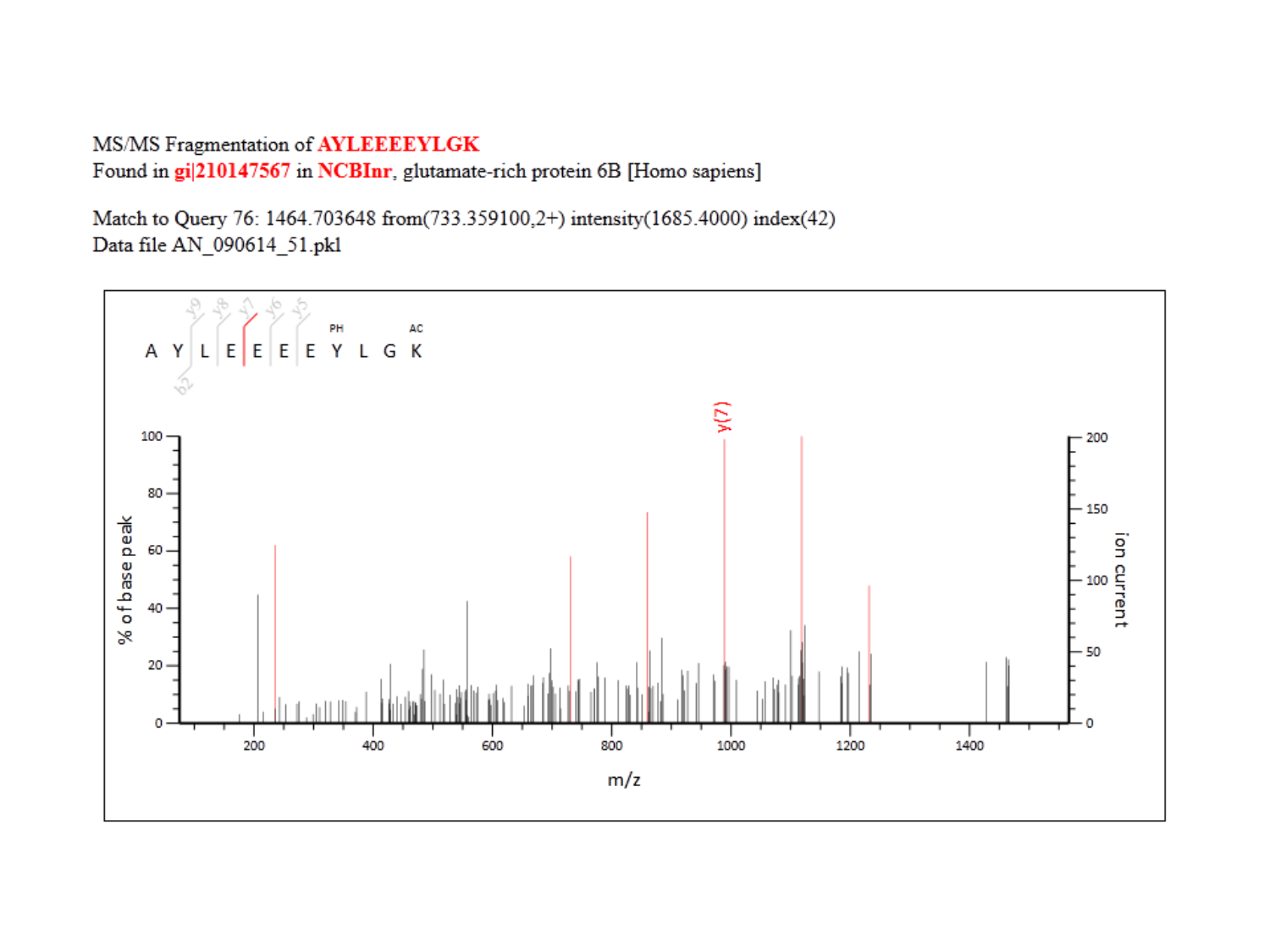

## Slide 57
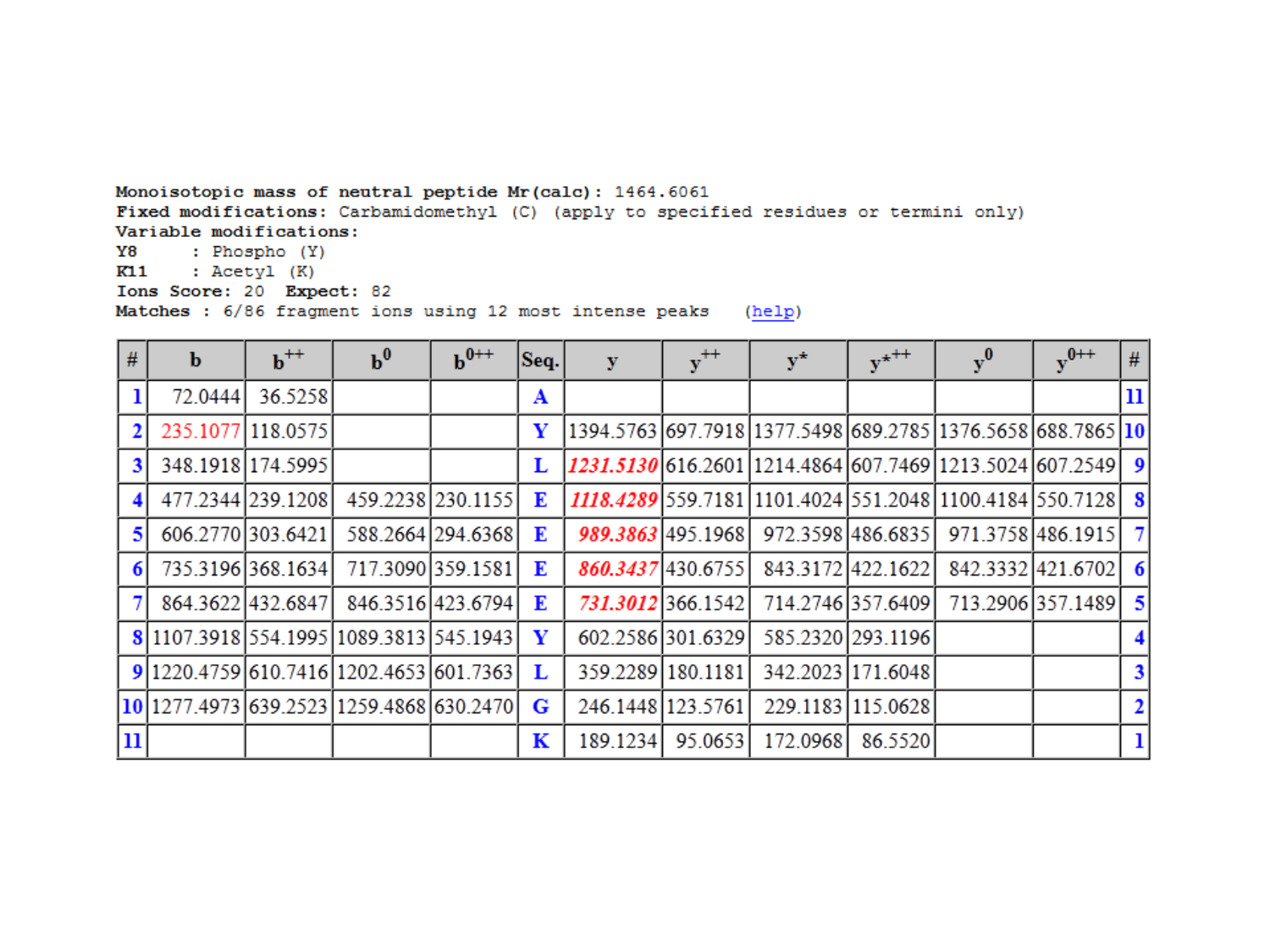

## Slide 58
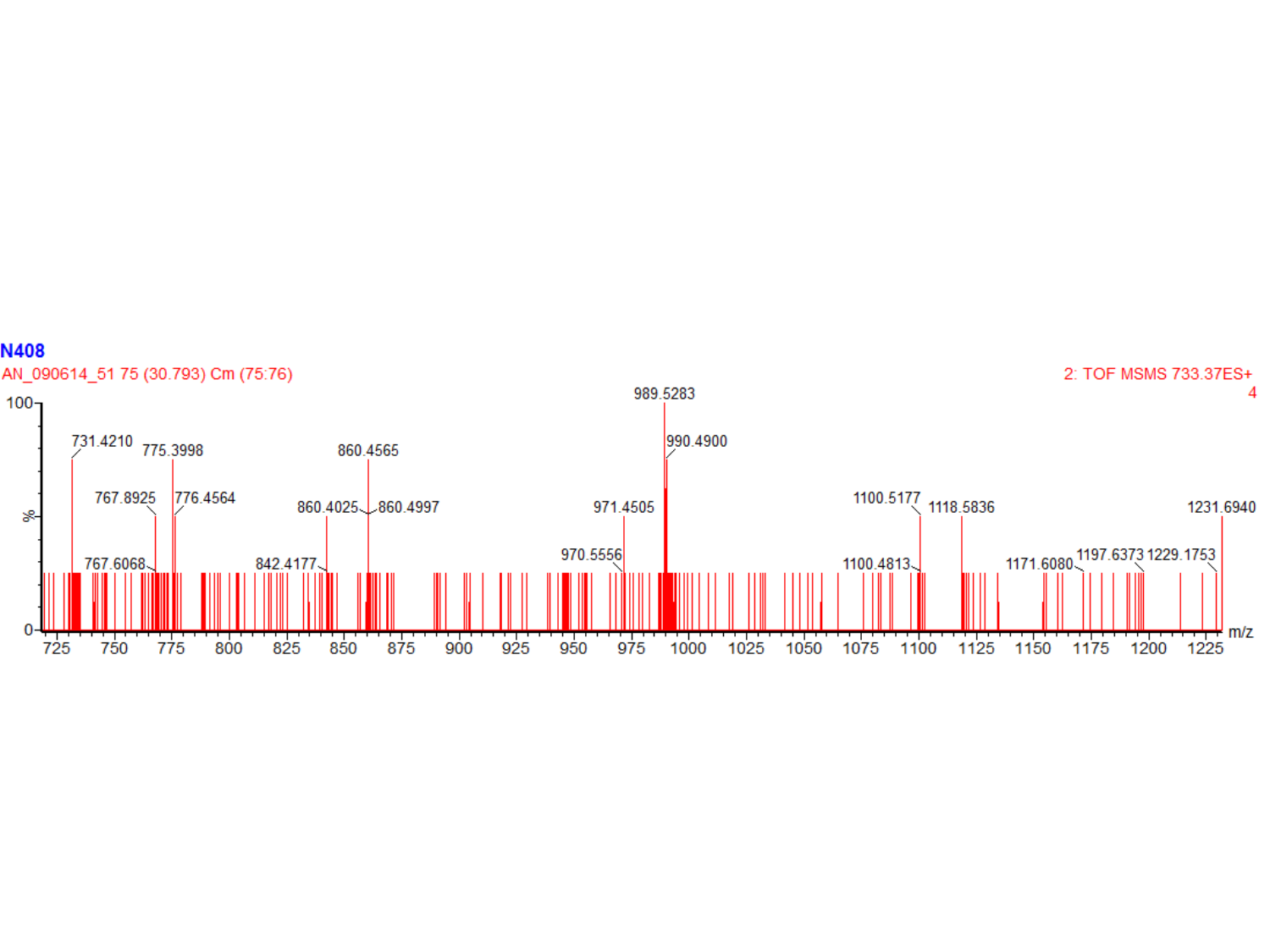

## Slide 59
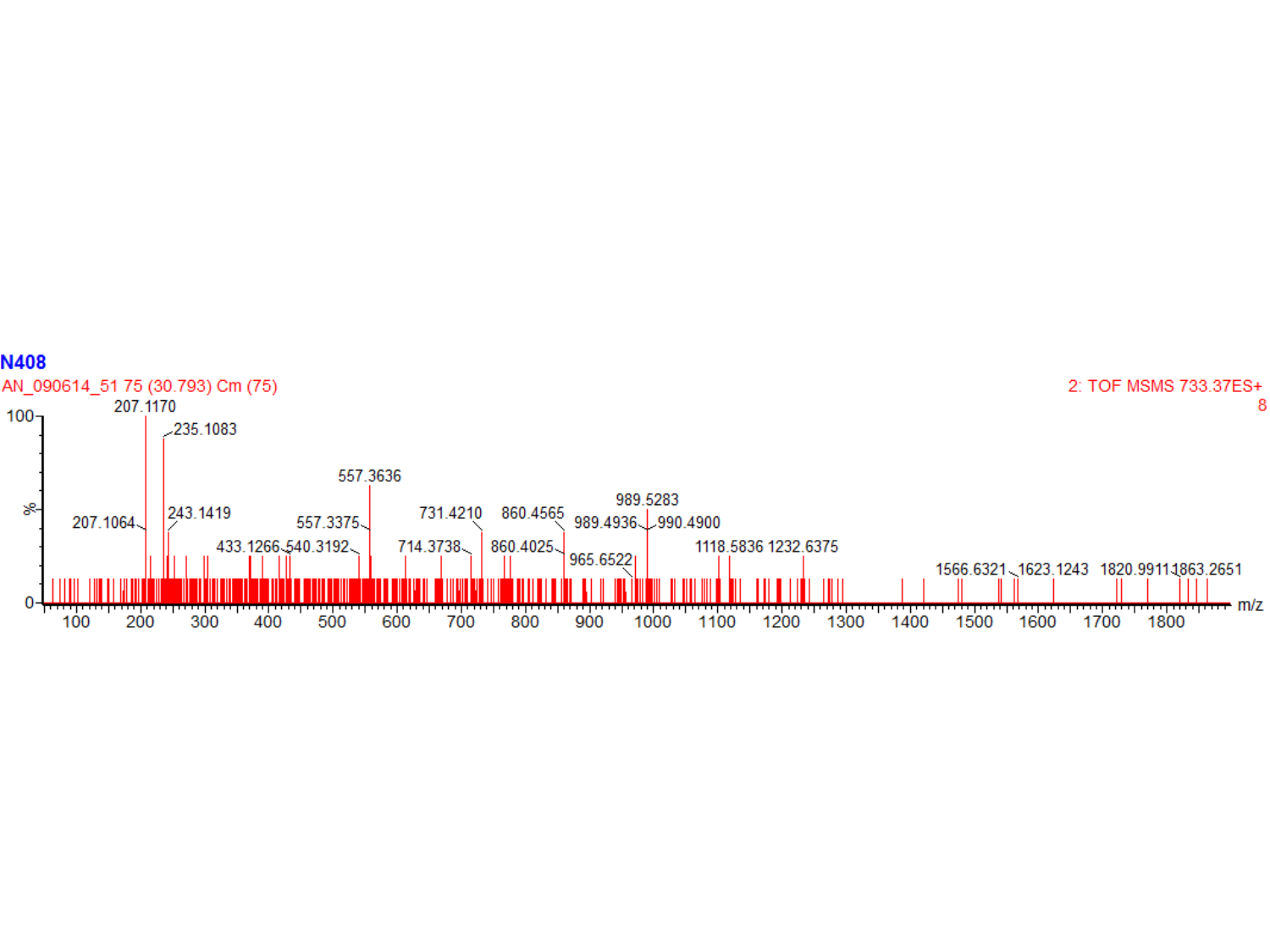

## Slide 60
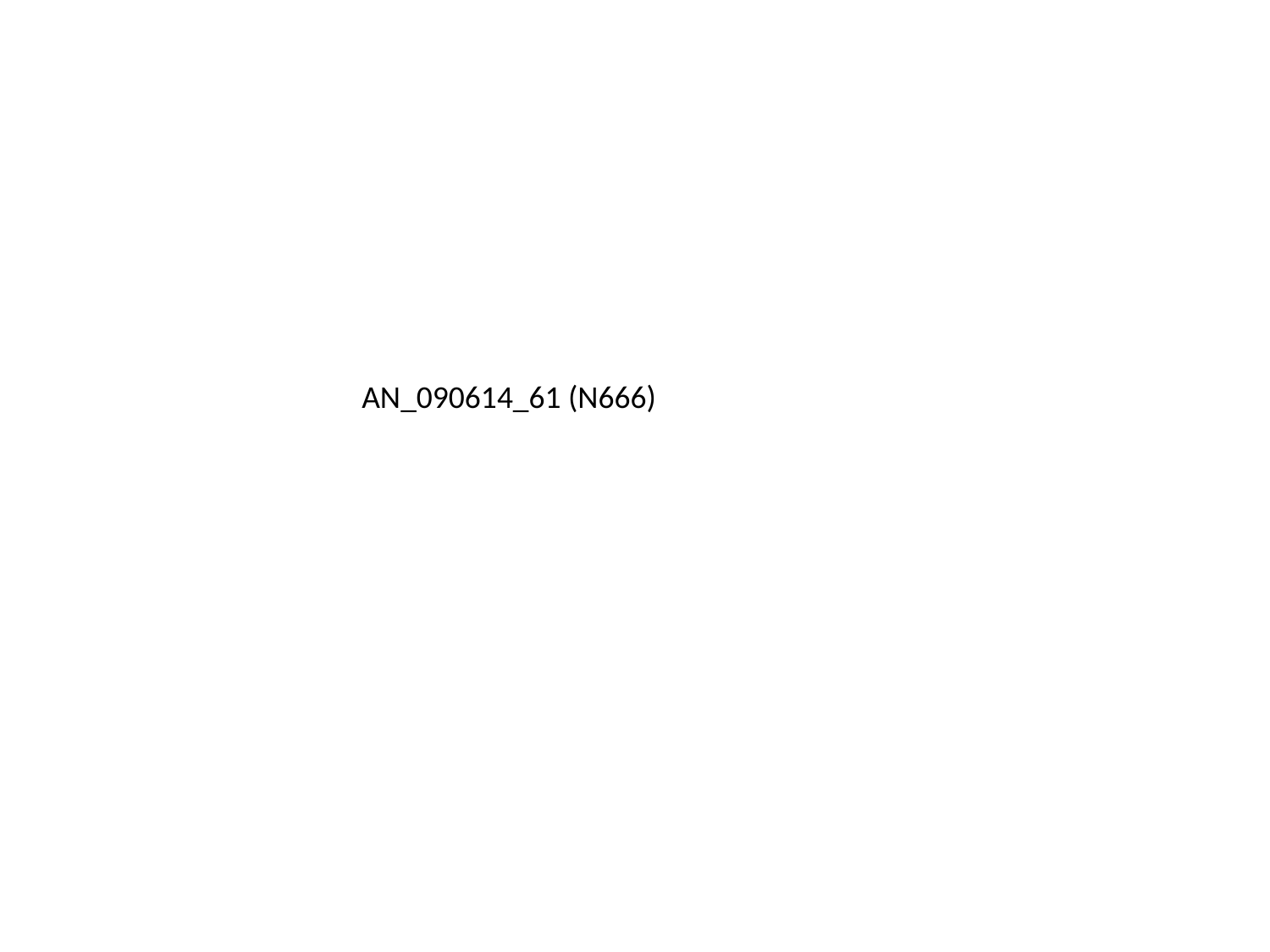

AN_090614_61 (N666)

## Slide 61
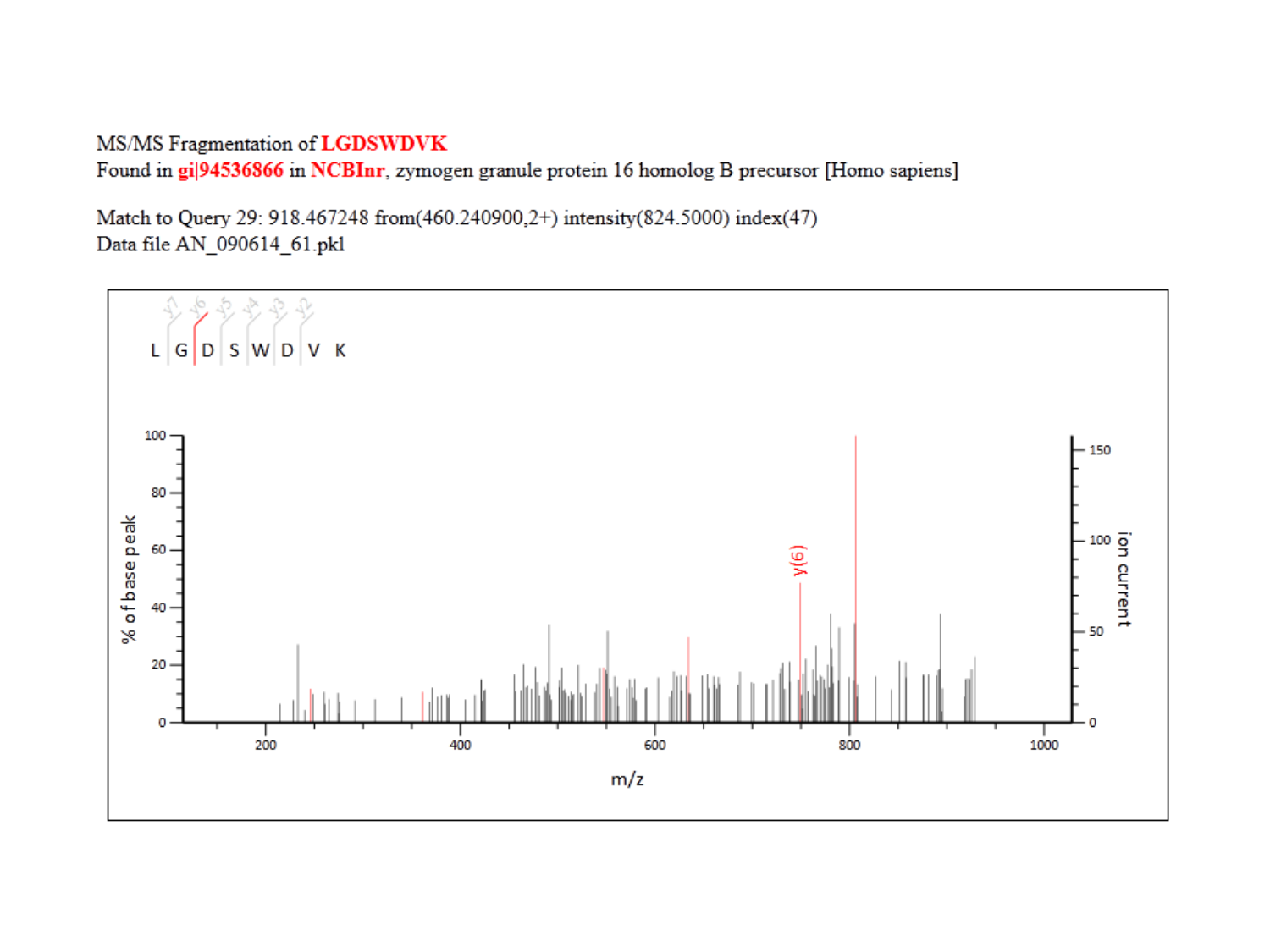

## Slide 62
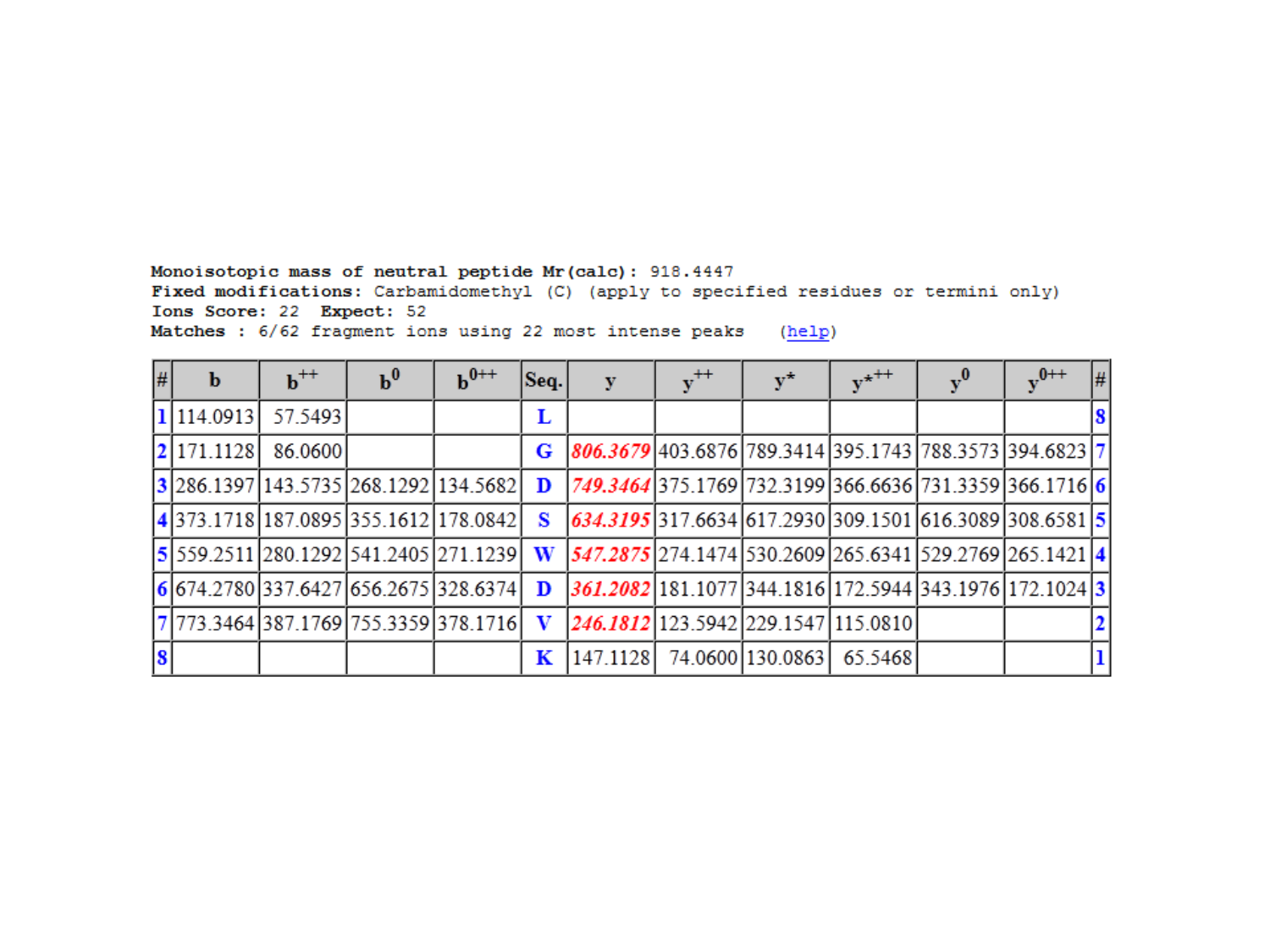

## Slide 63
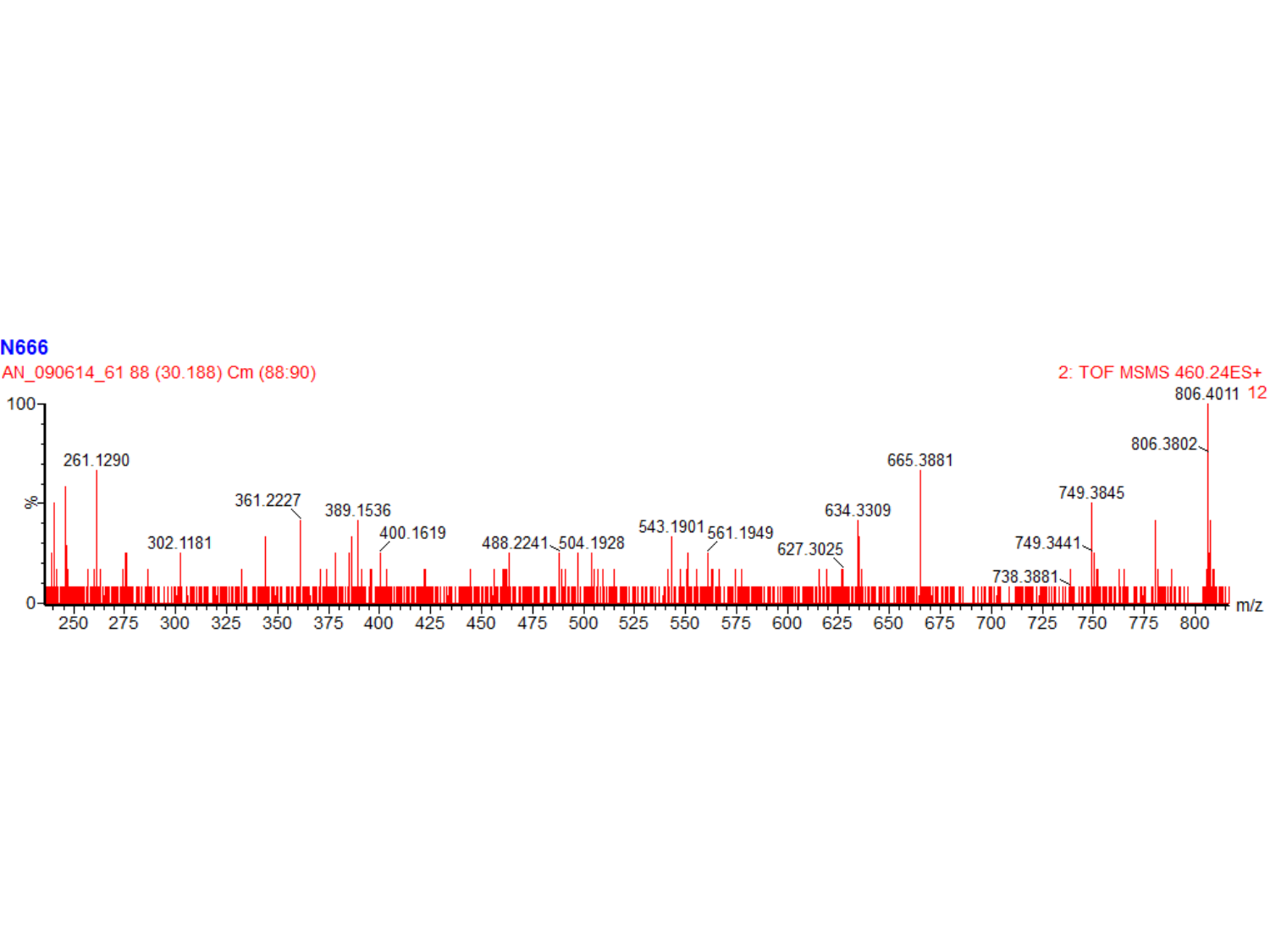

## Slide 64
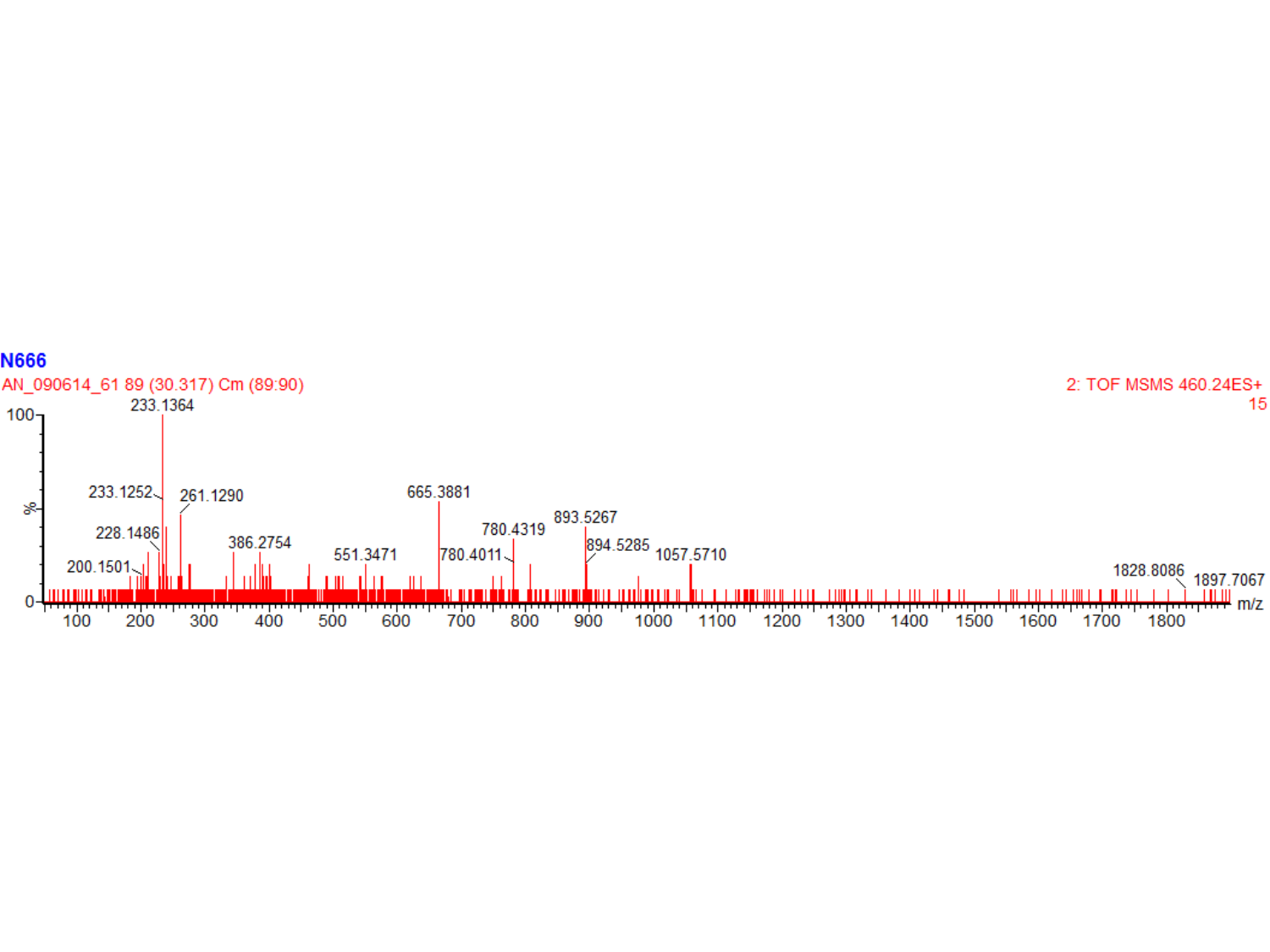

## Slide 65
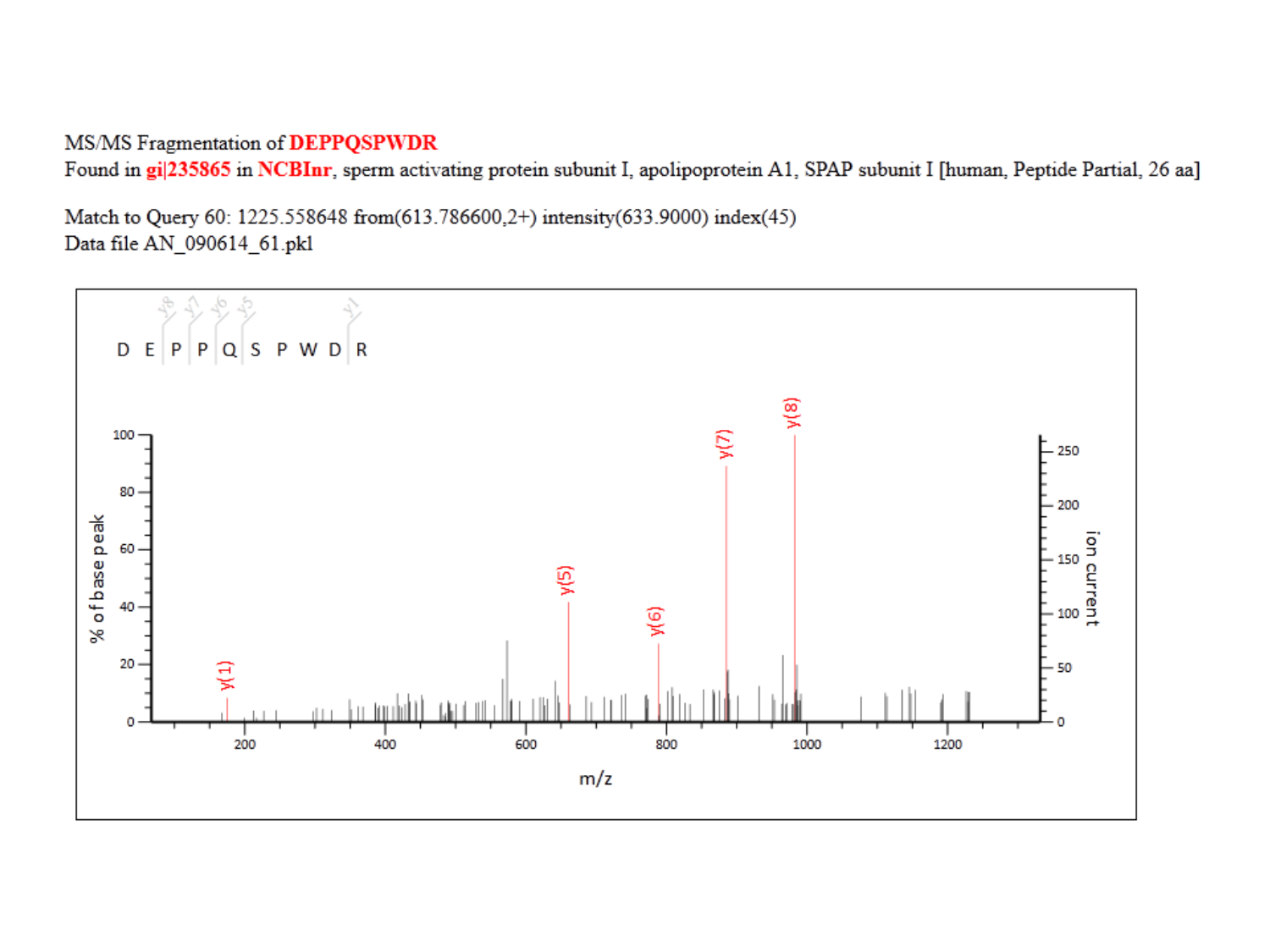

## Slide 66
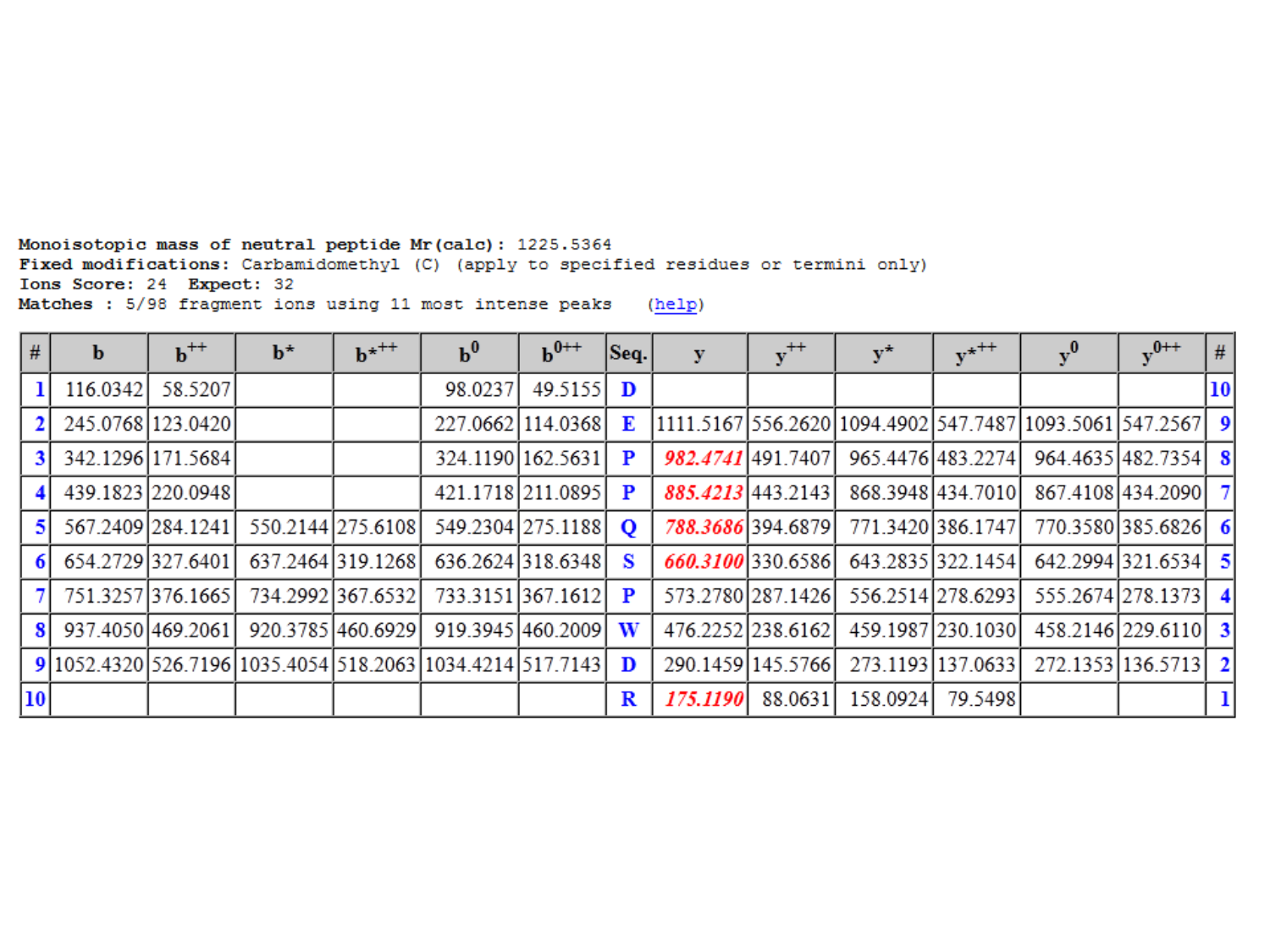

## Slide 67
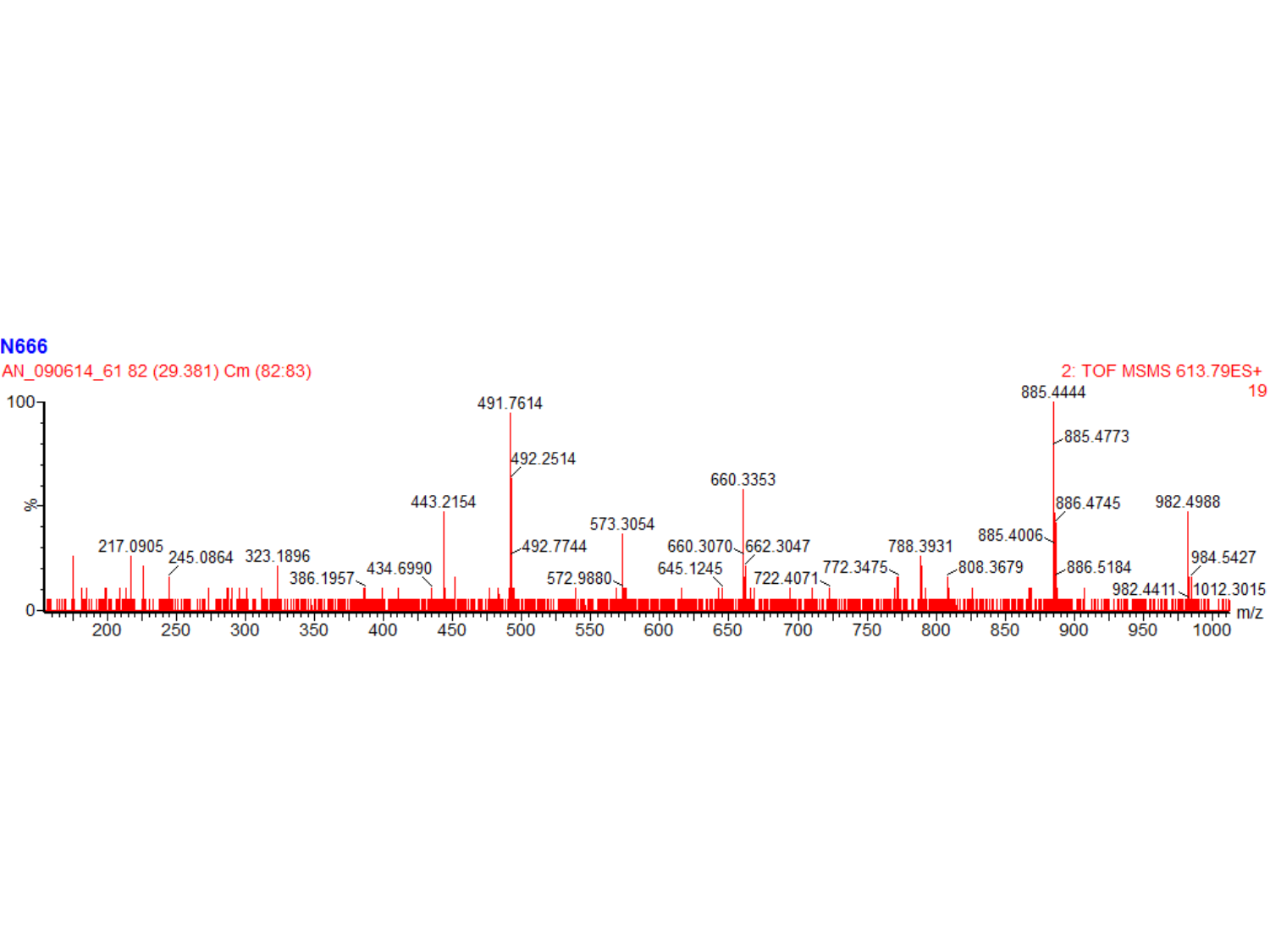

## Slide 68
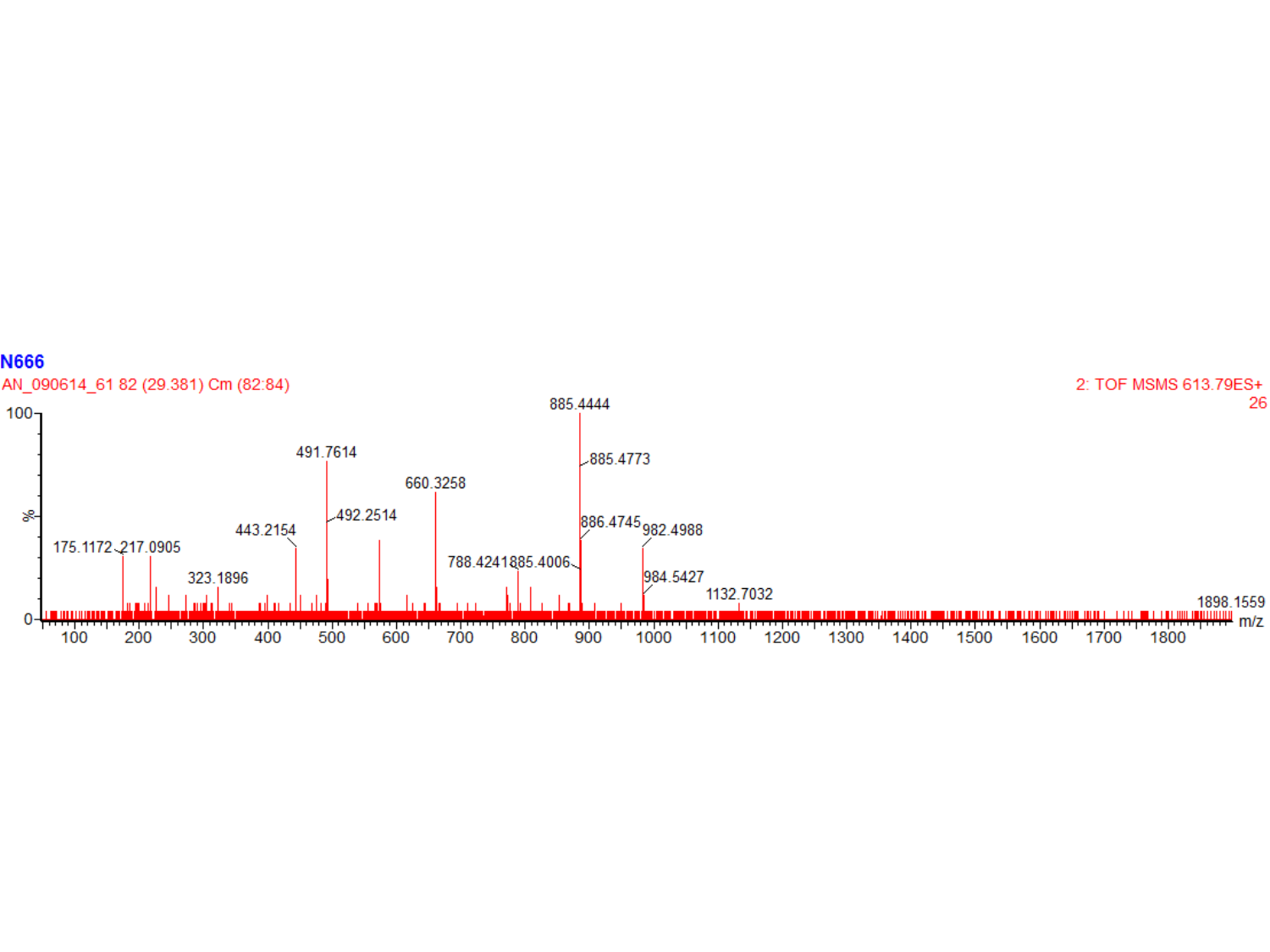

## Slide 69
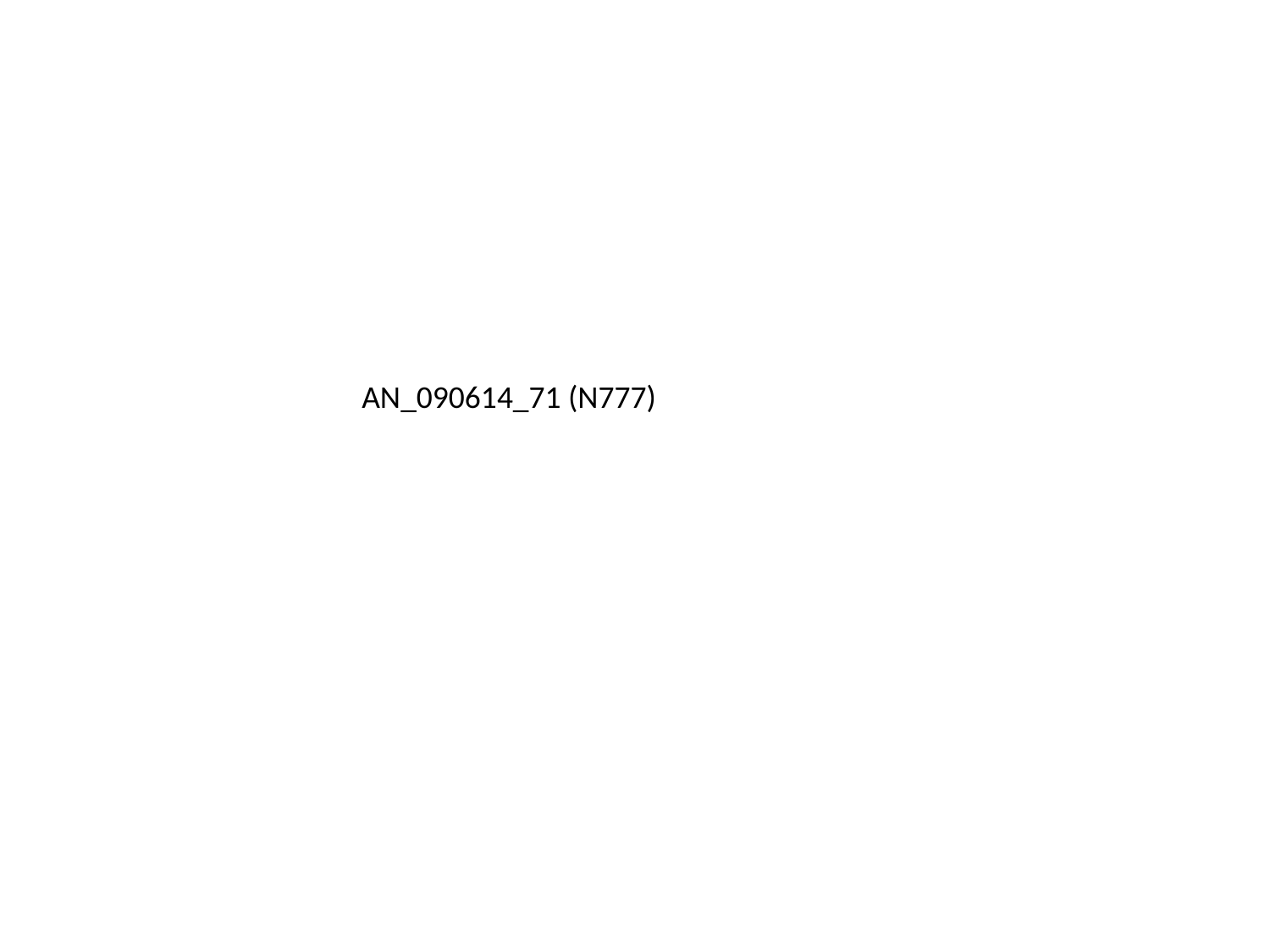

AN_090614_71 (N777)

## Slide 70
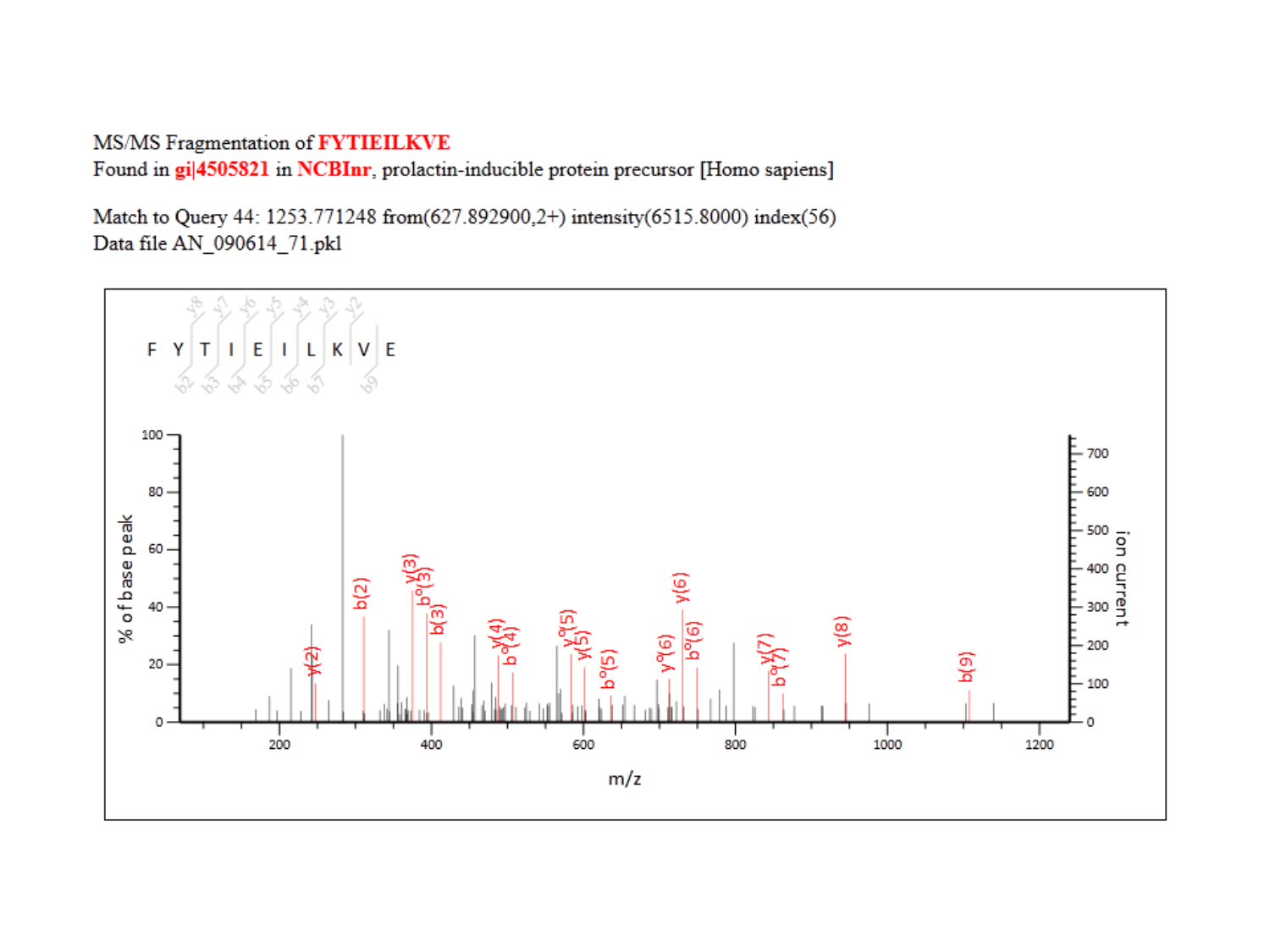

## Slide 71
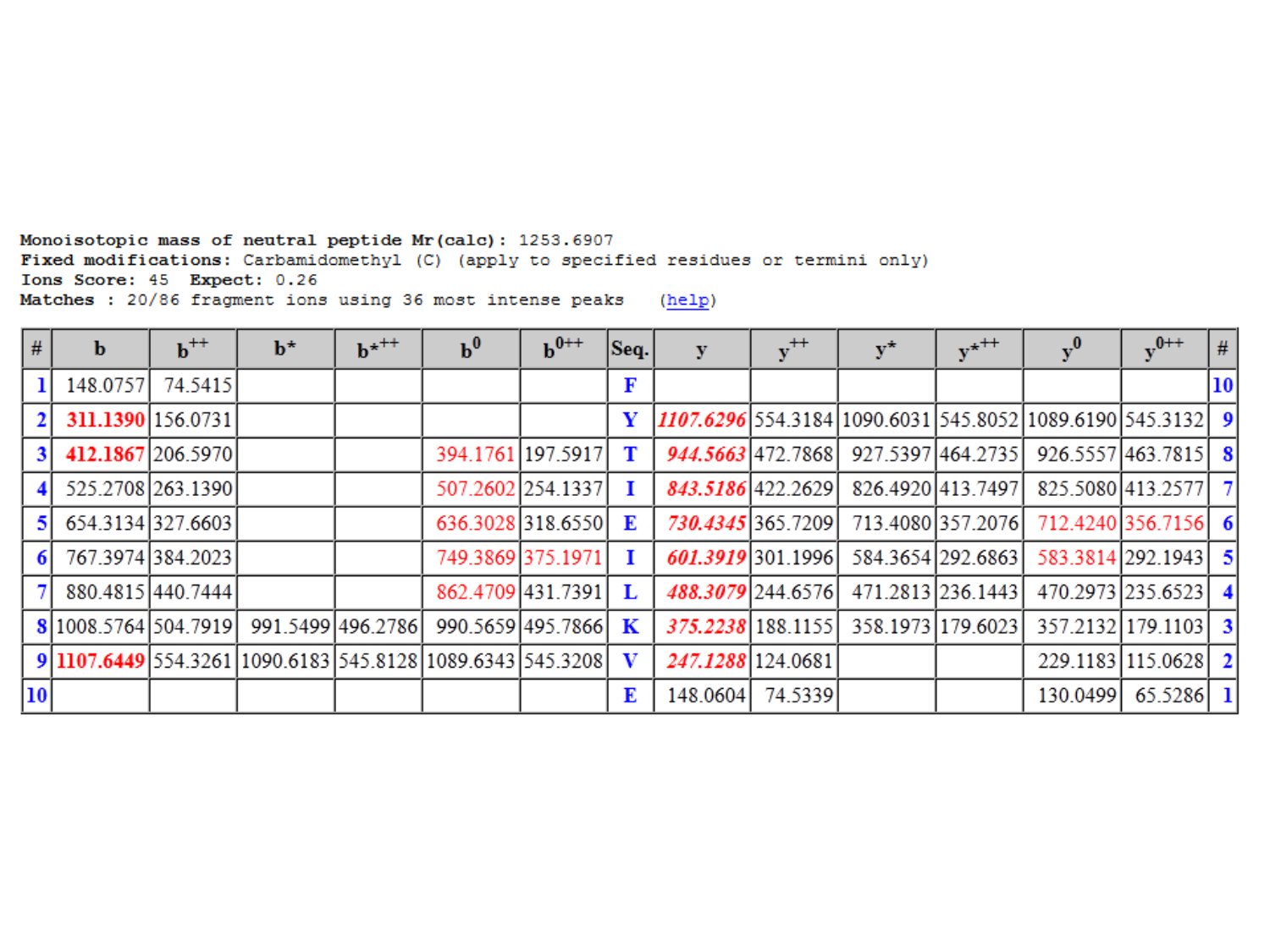

## Slide 72
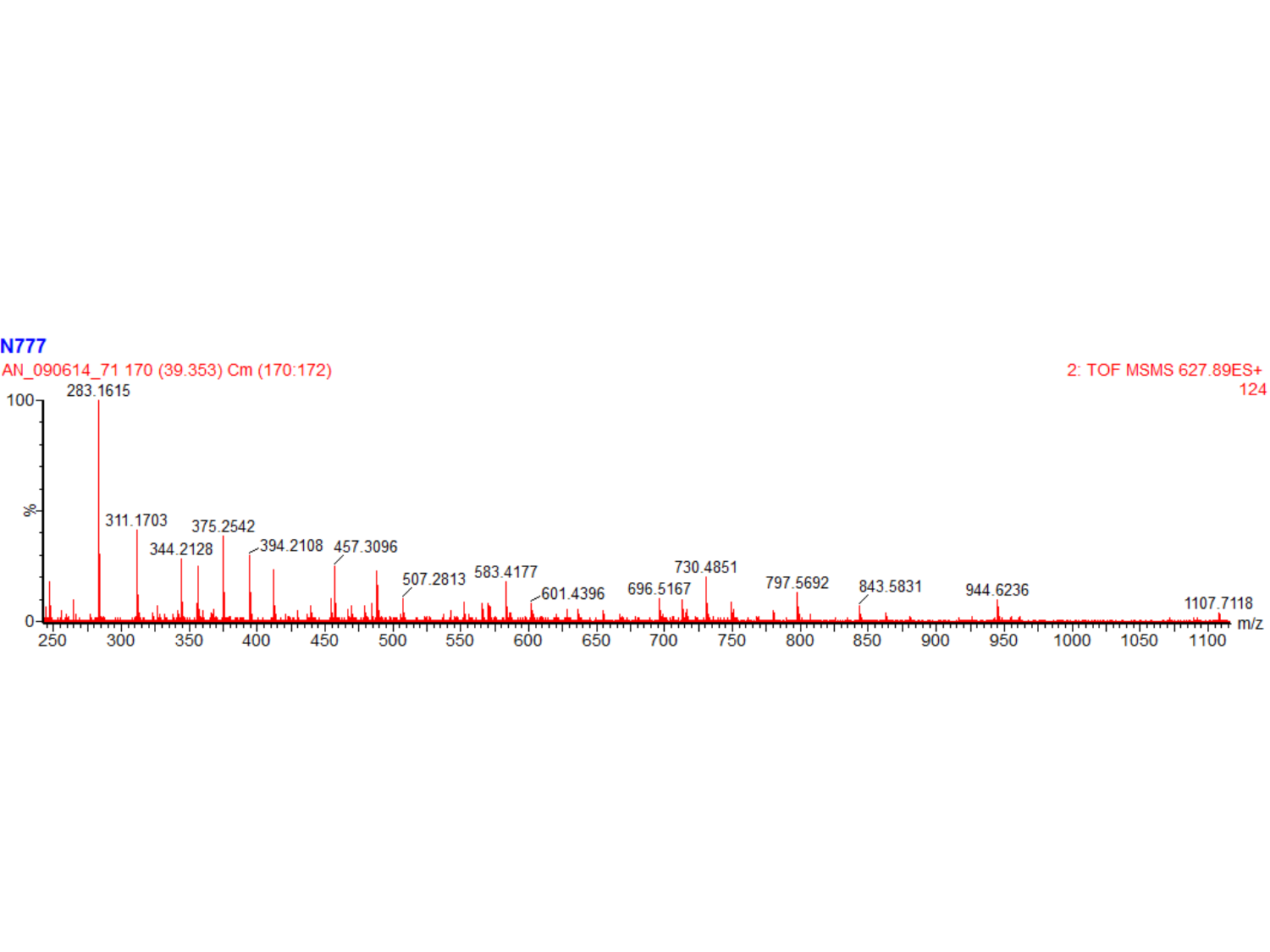

## Slide 73
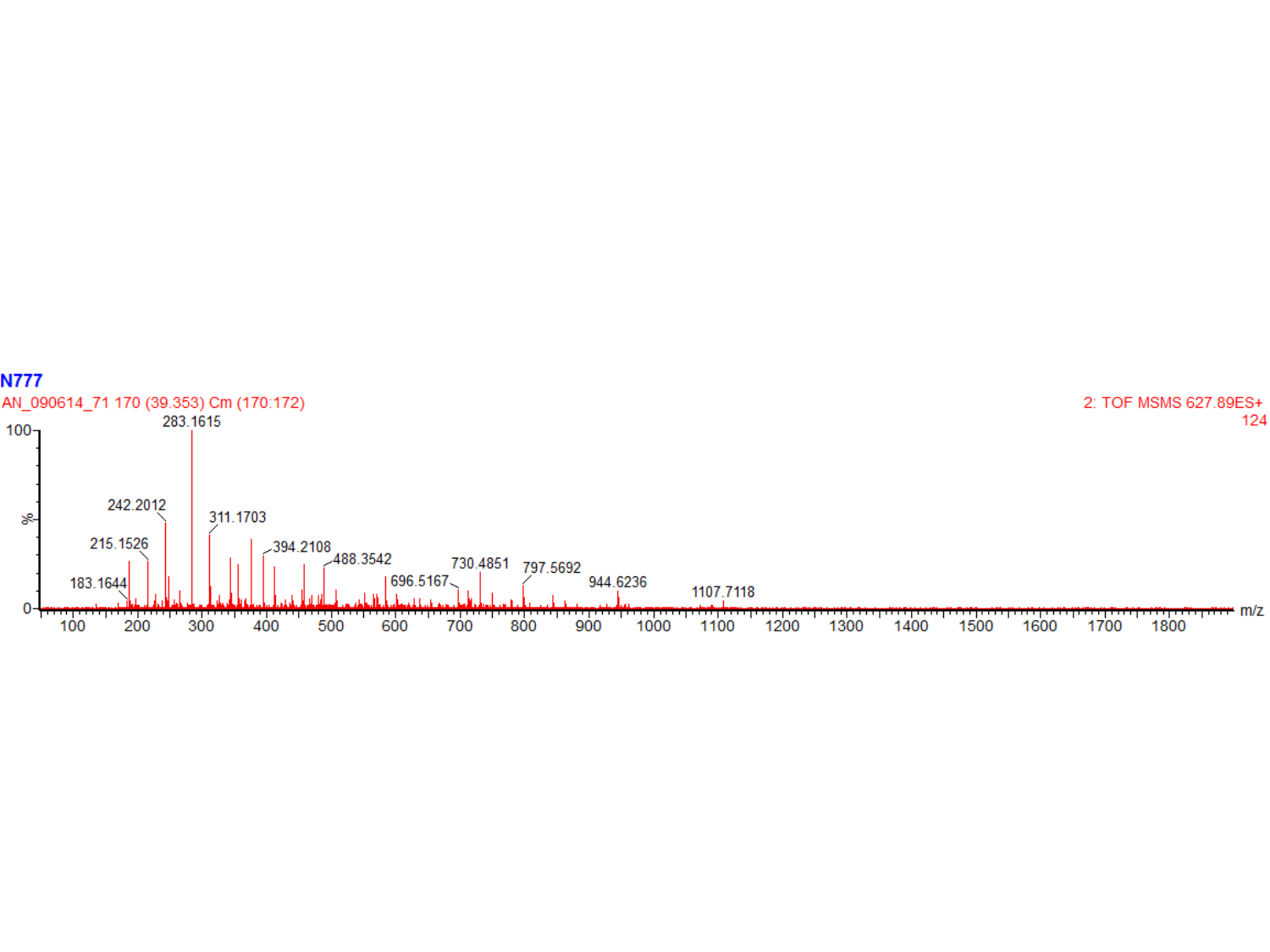

## Slide 74
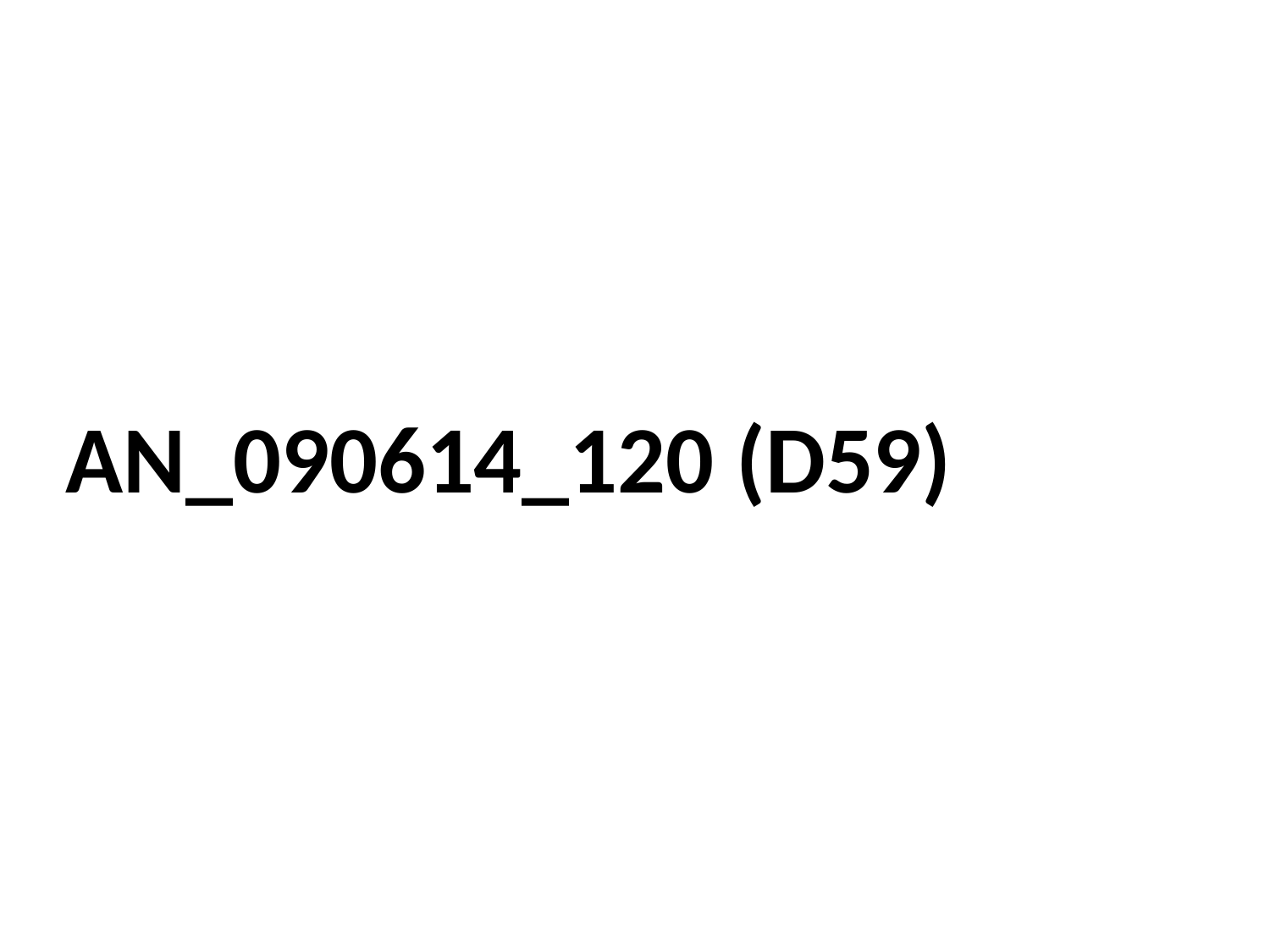

#
AN_090614_120 (D59)

## Slide 75
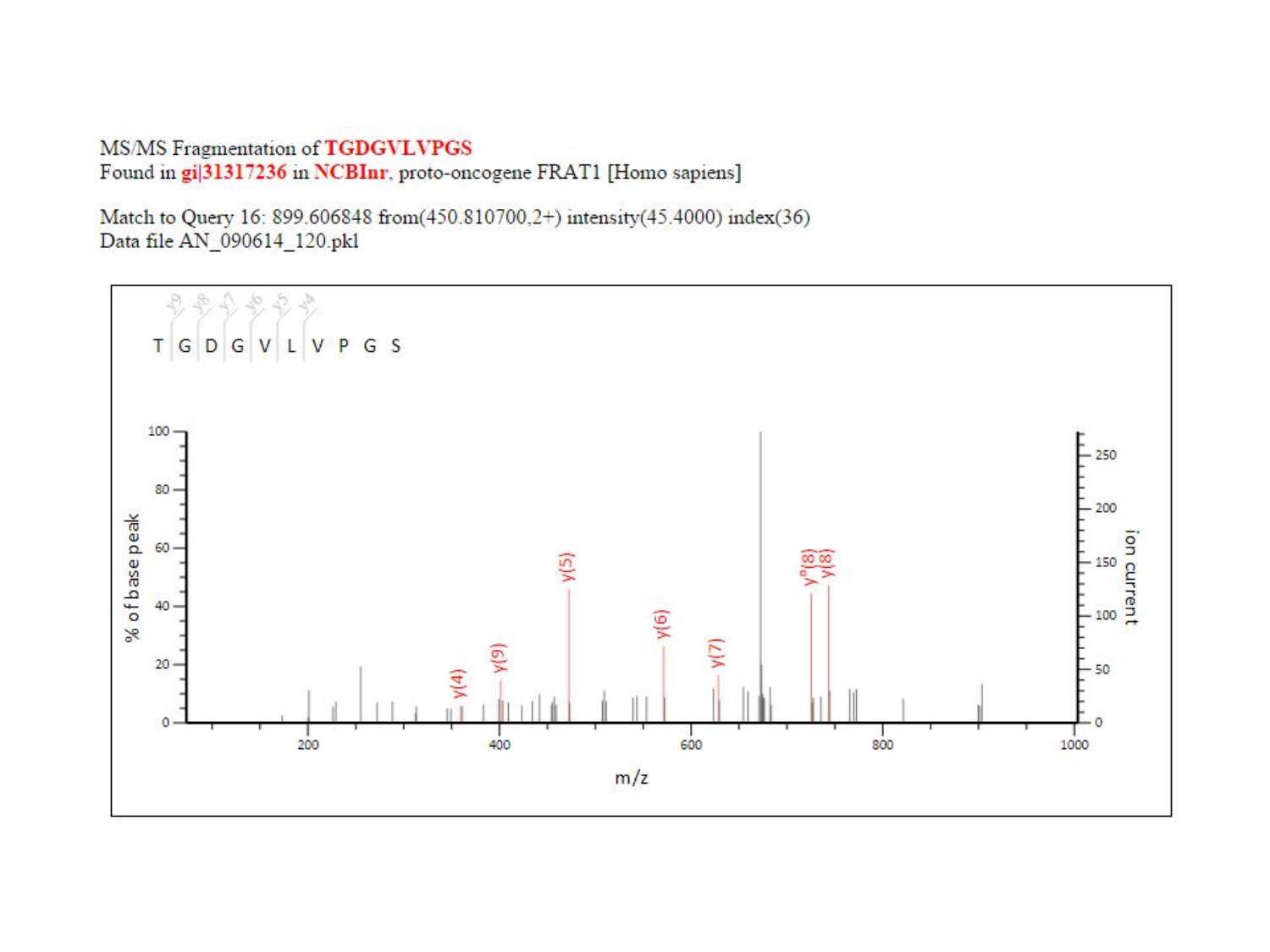

#

## Slide 76
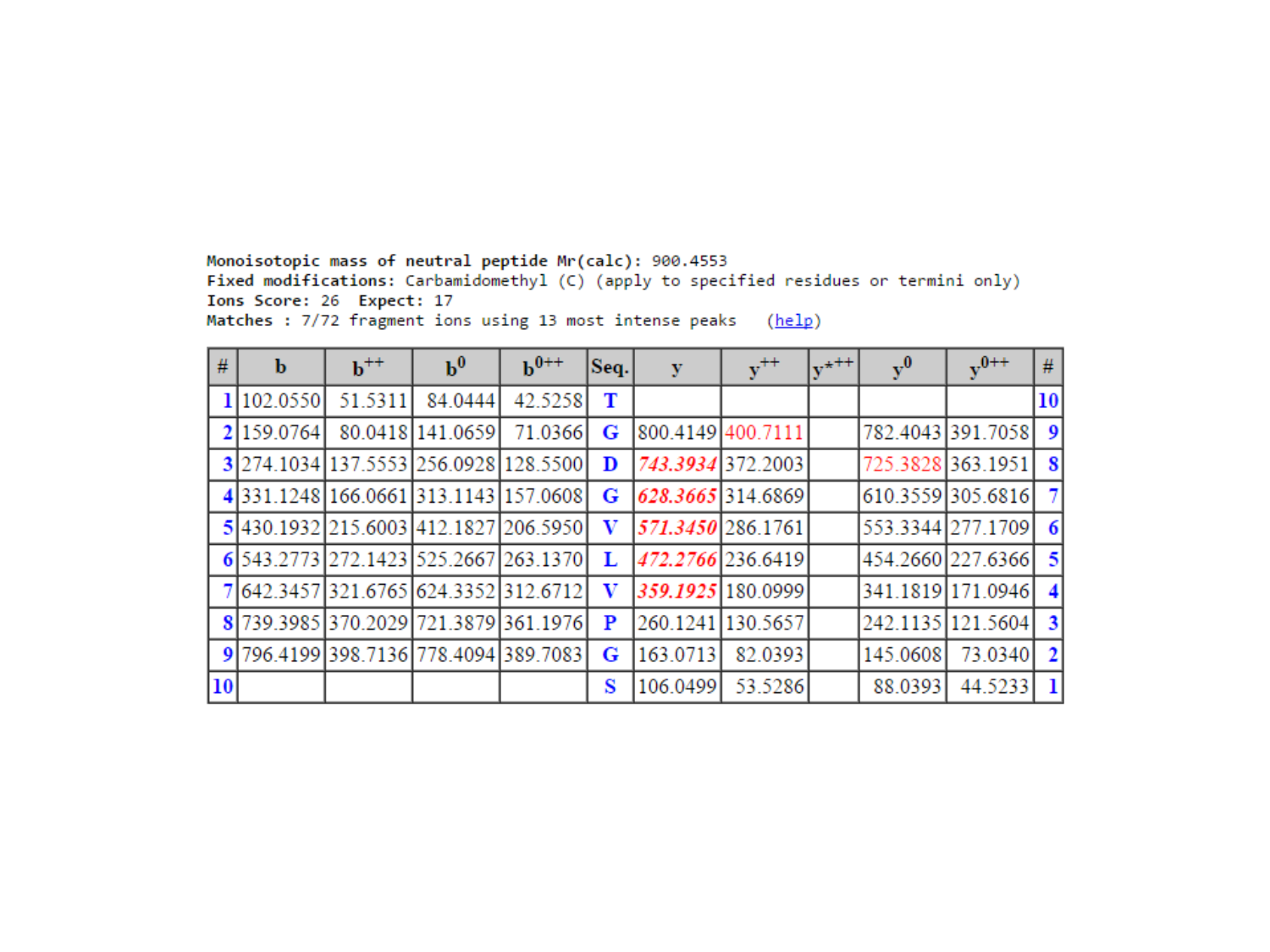

#

## Slide 77
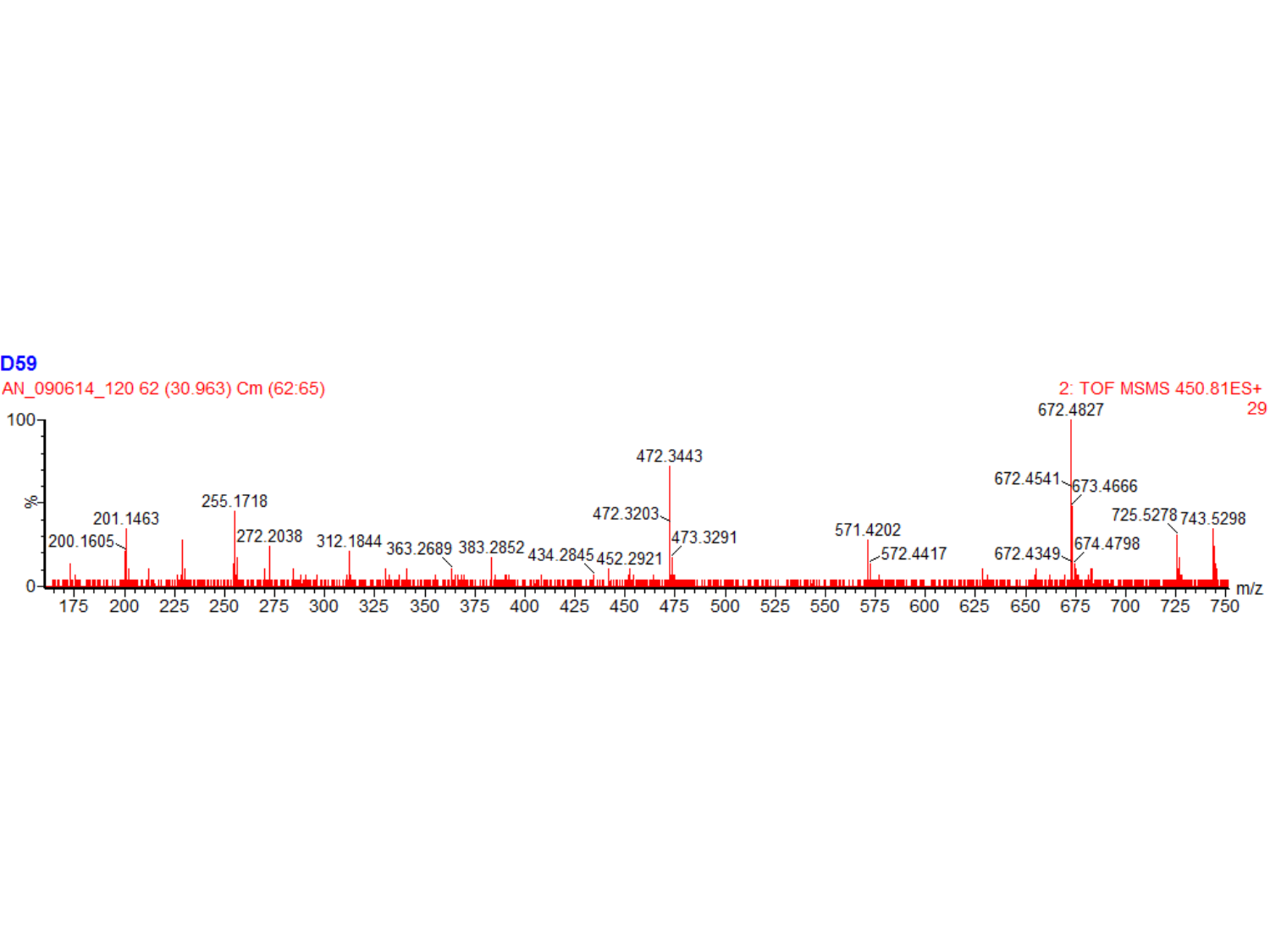

#

## Slide 78
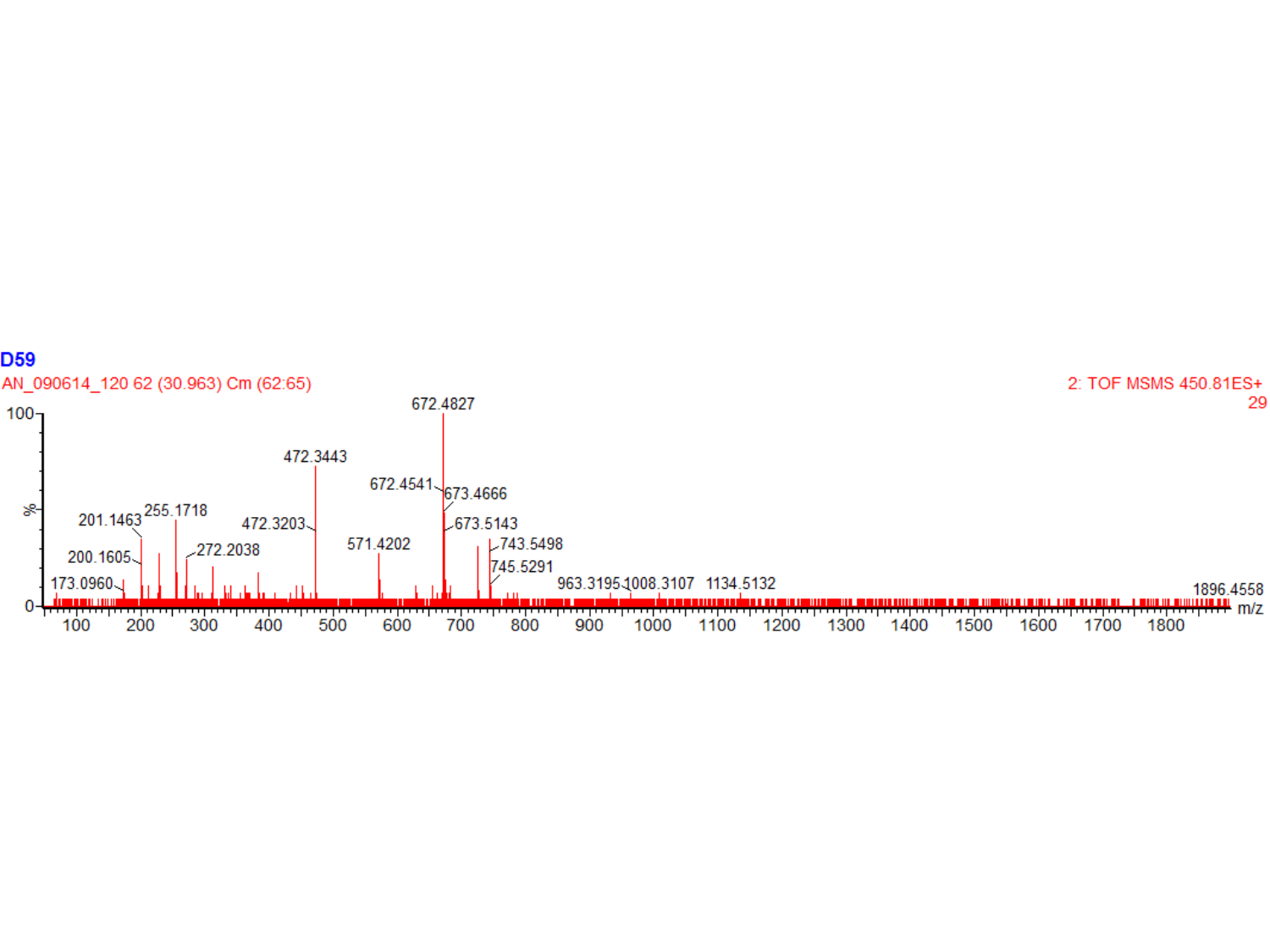

#

## Slide 79
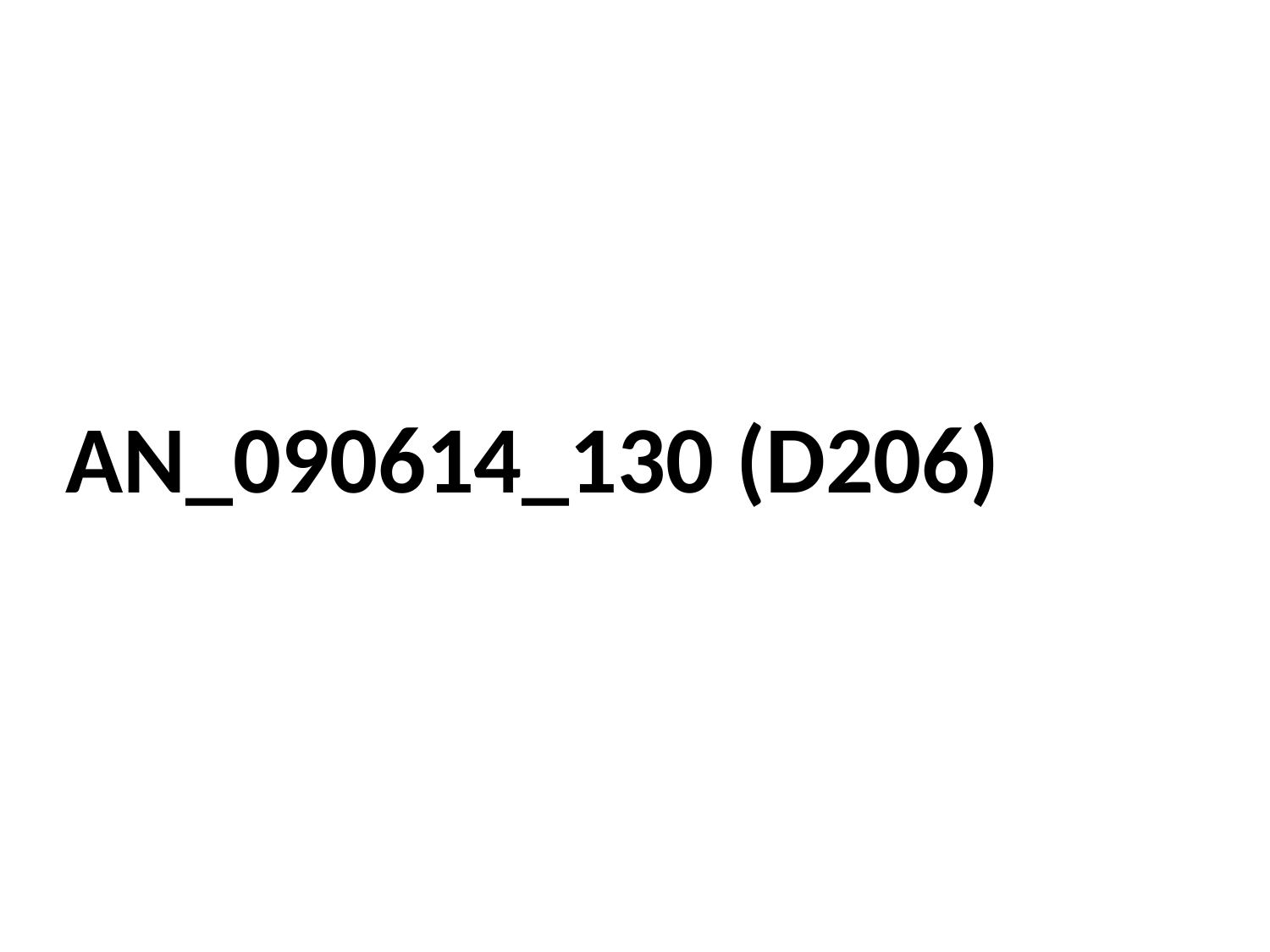

#
AN_090614_130 (D206)

## Slide 80
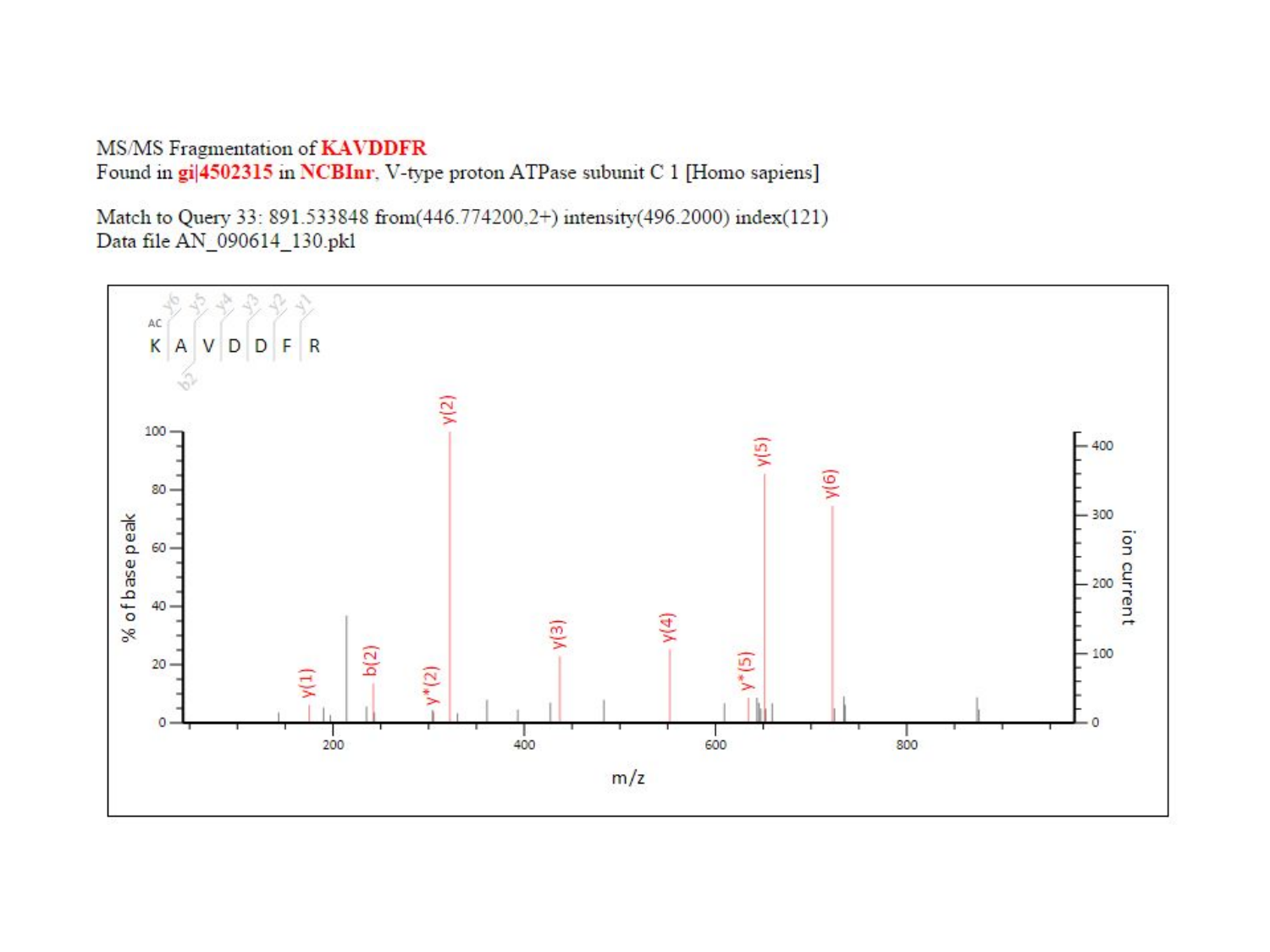

#

## Slide 81
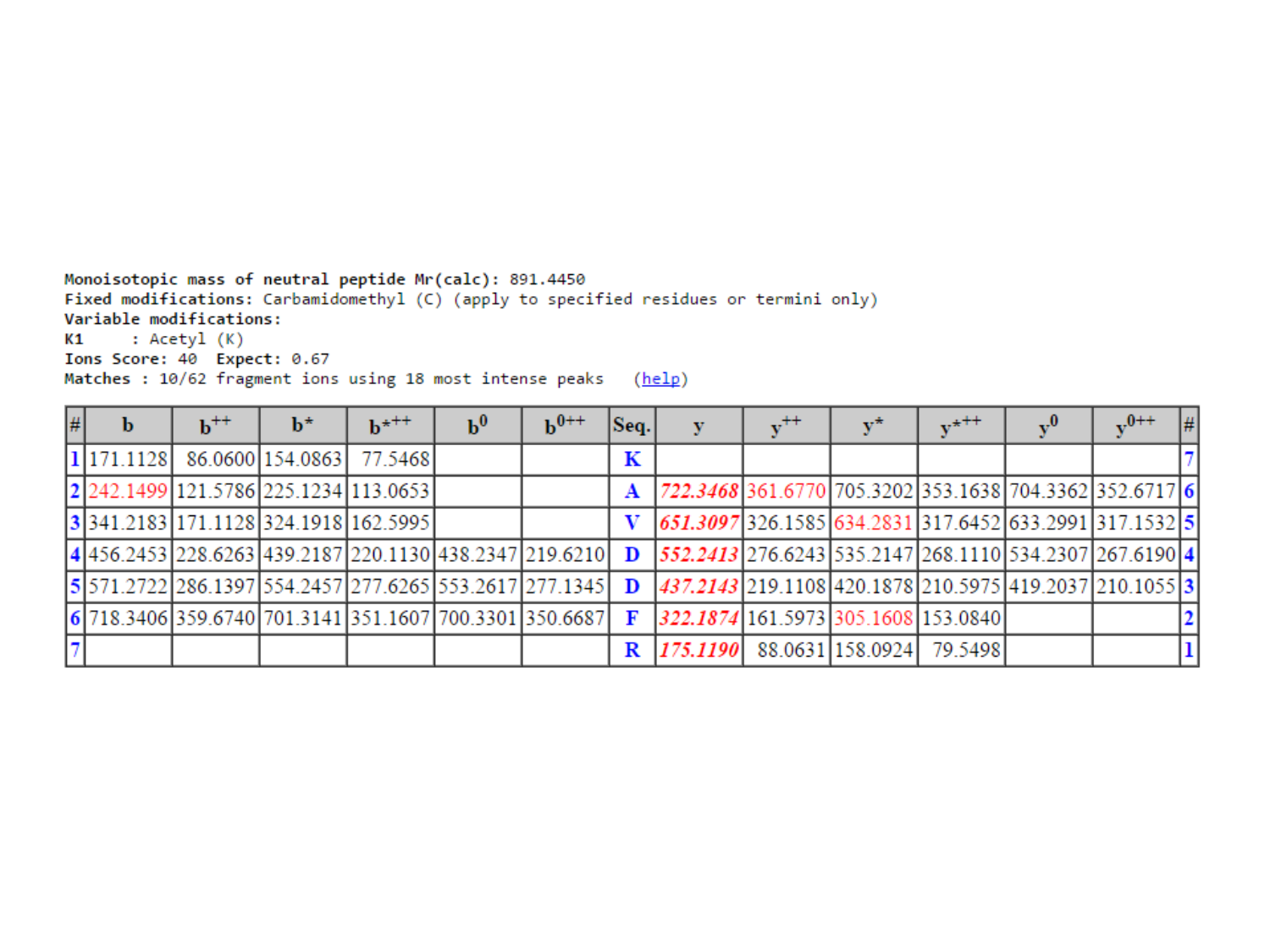

#

## Slide 82
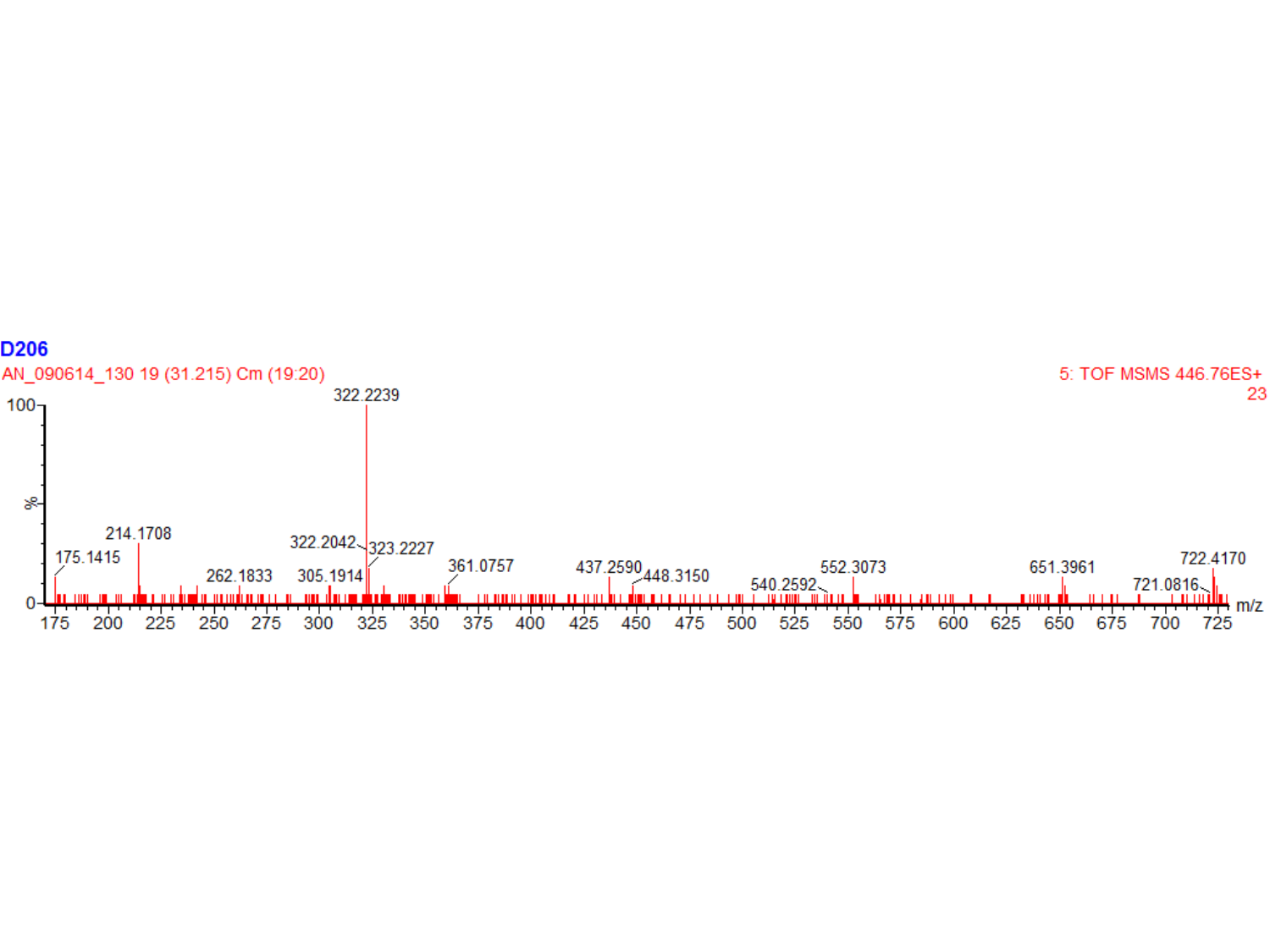

#

## Slide 83
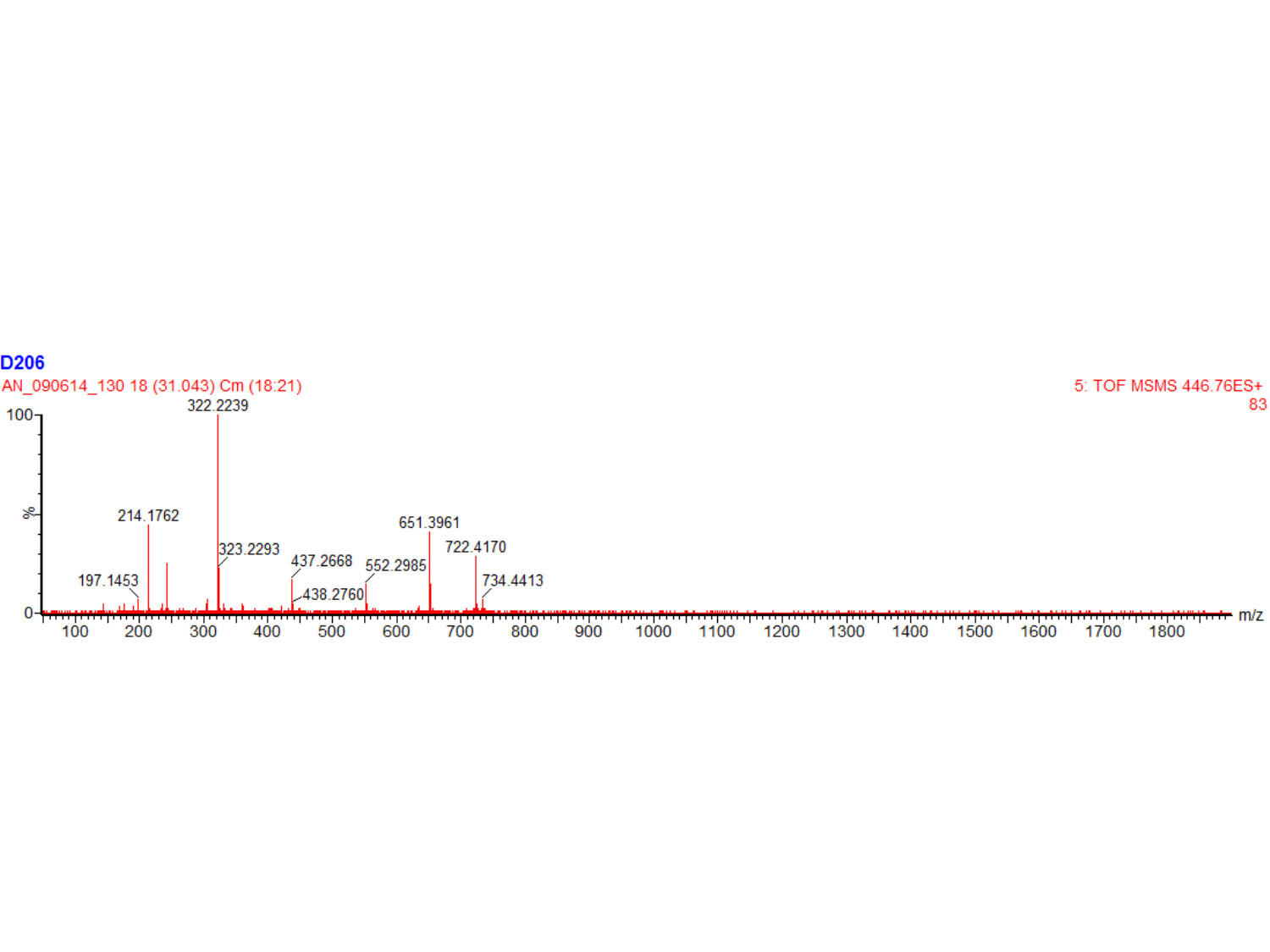

#

## Slide 84
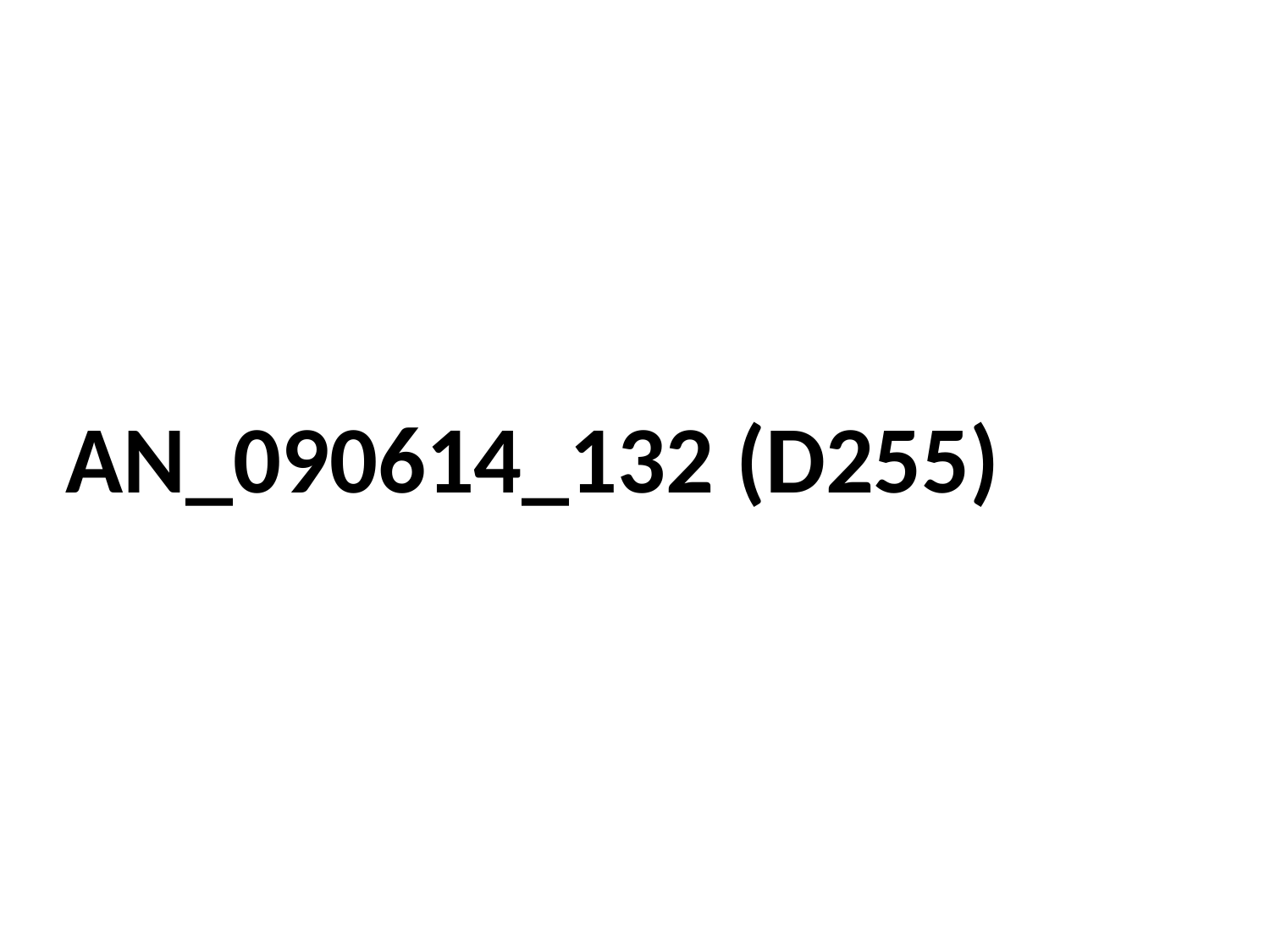

#
AN_090614_132 (D255)

## Slide 85
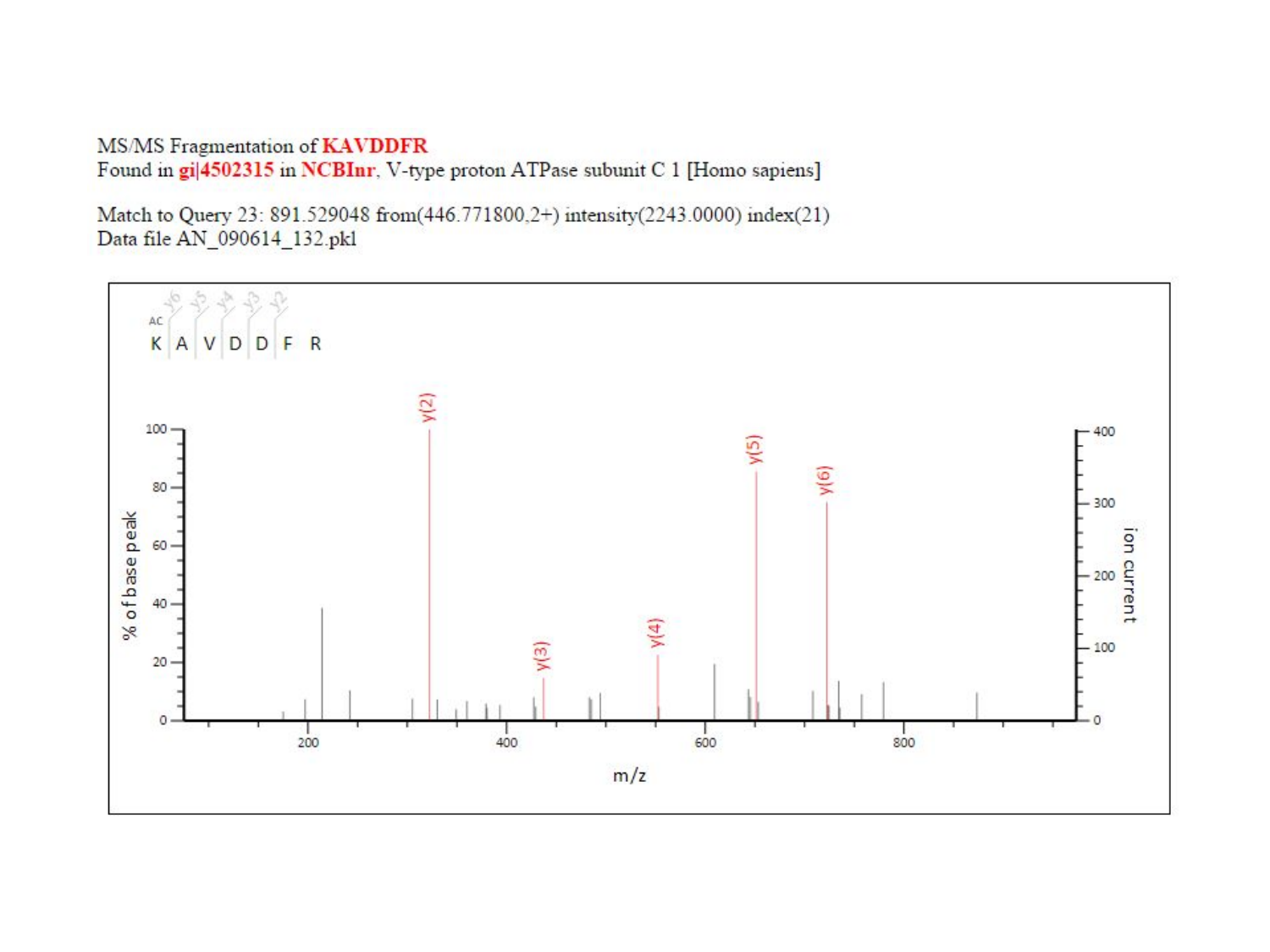

#

## Slide 86
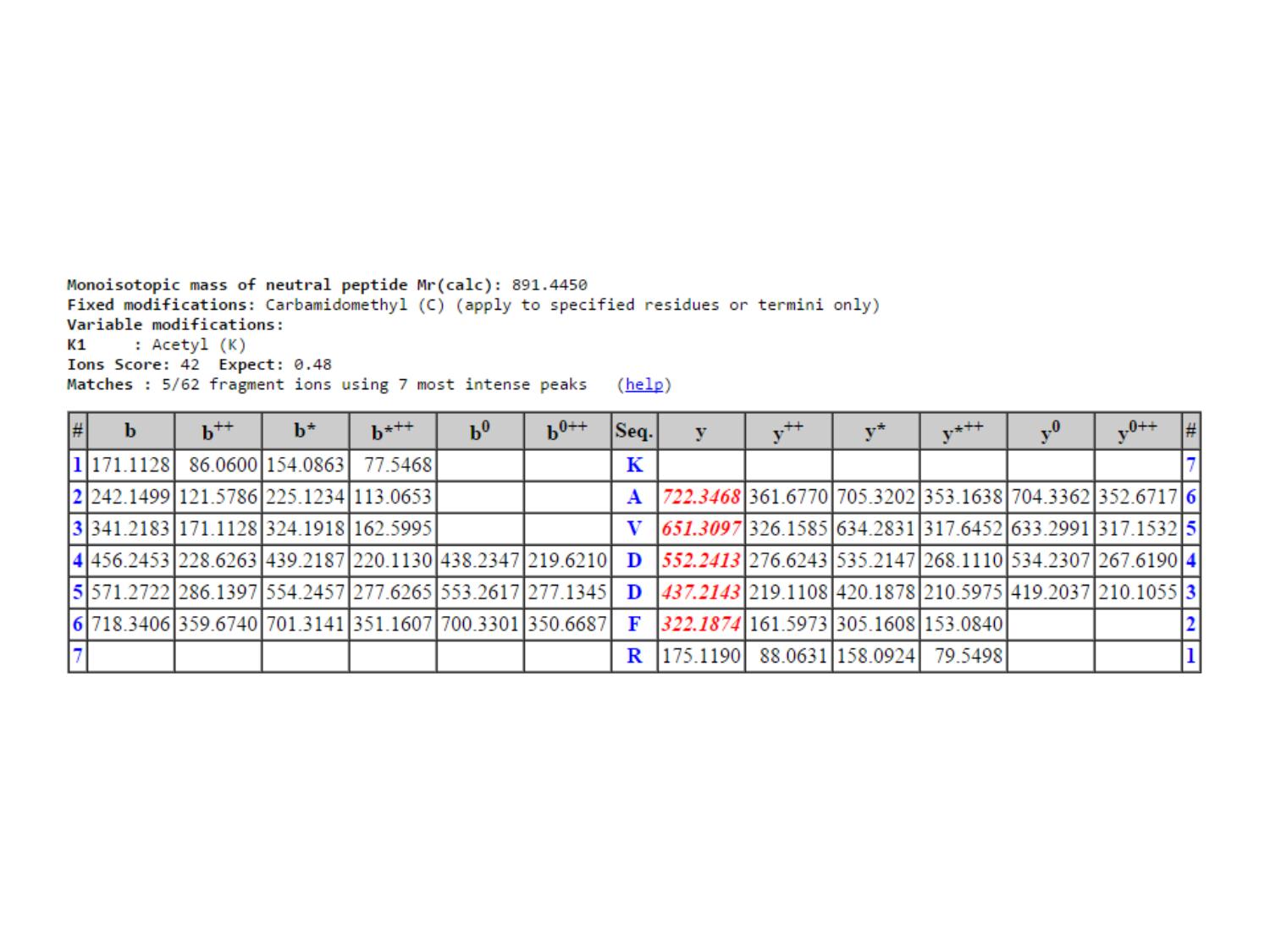

#

## Slide 87
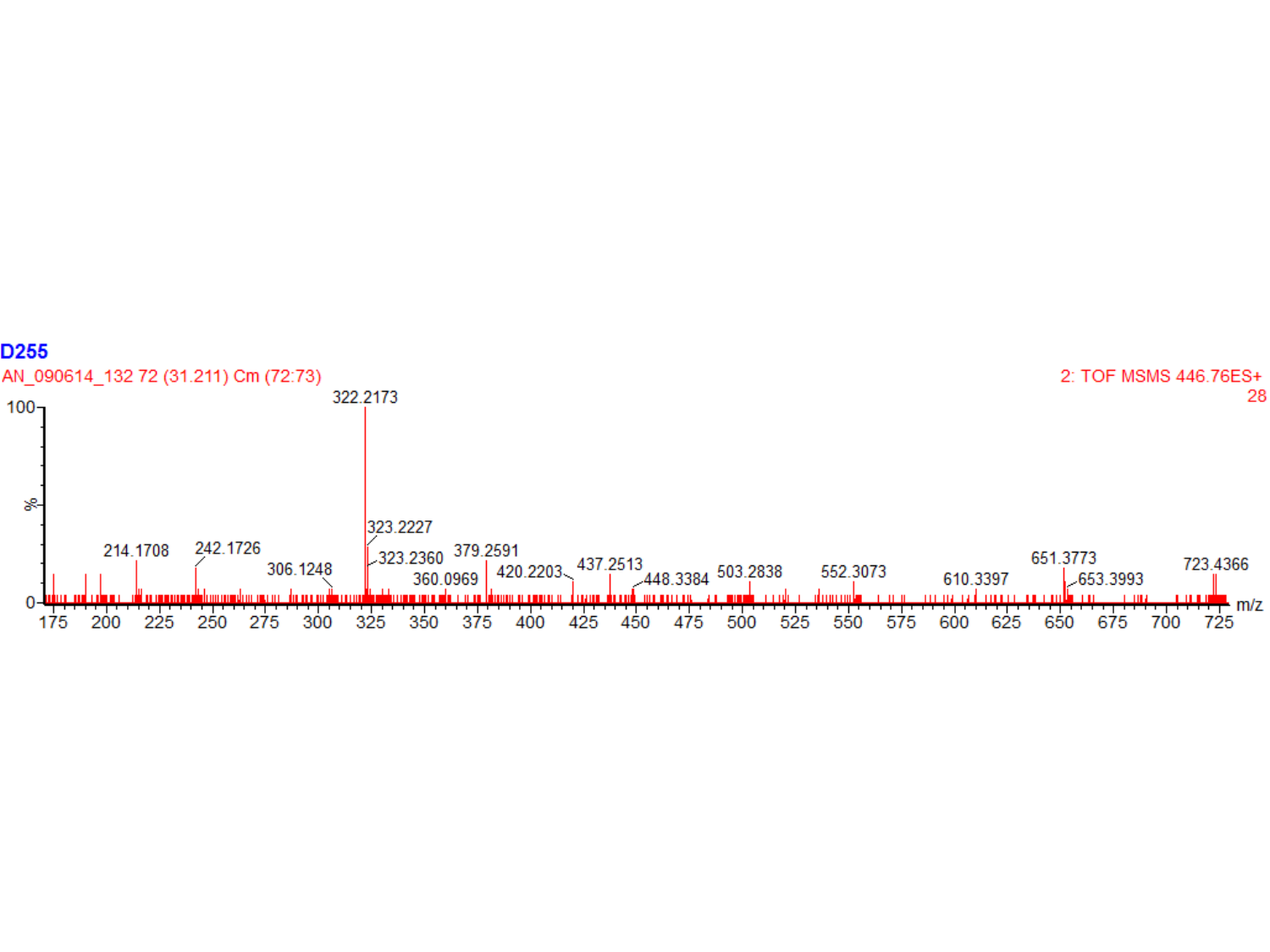

#

## Slide 88
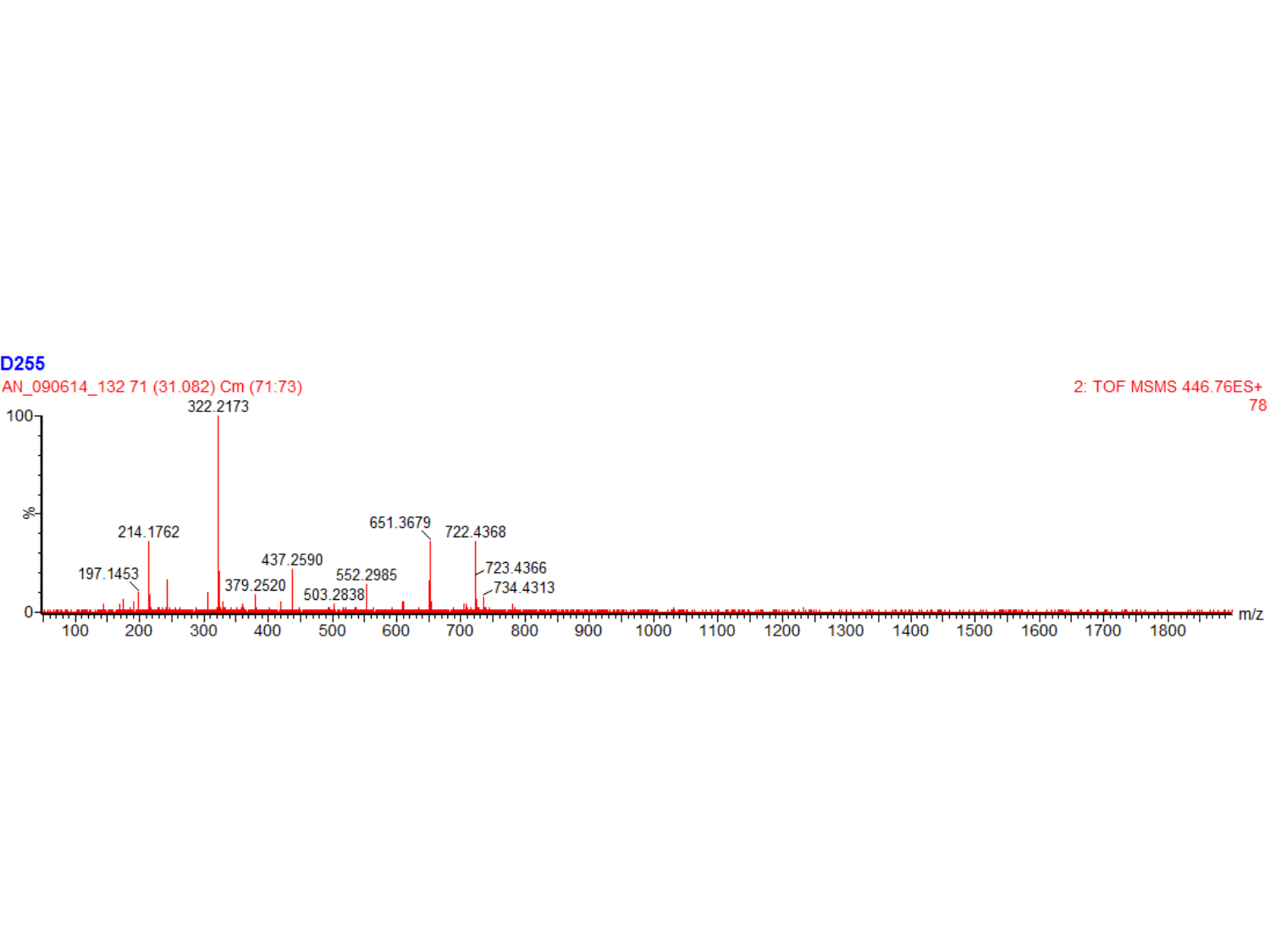

#

## Slide 89
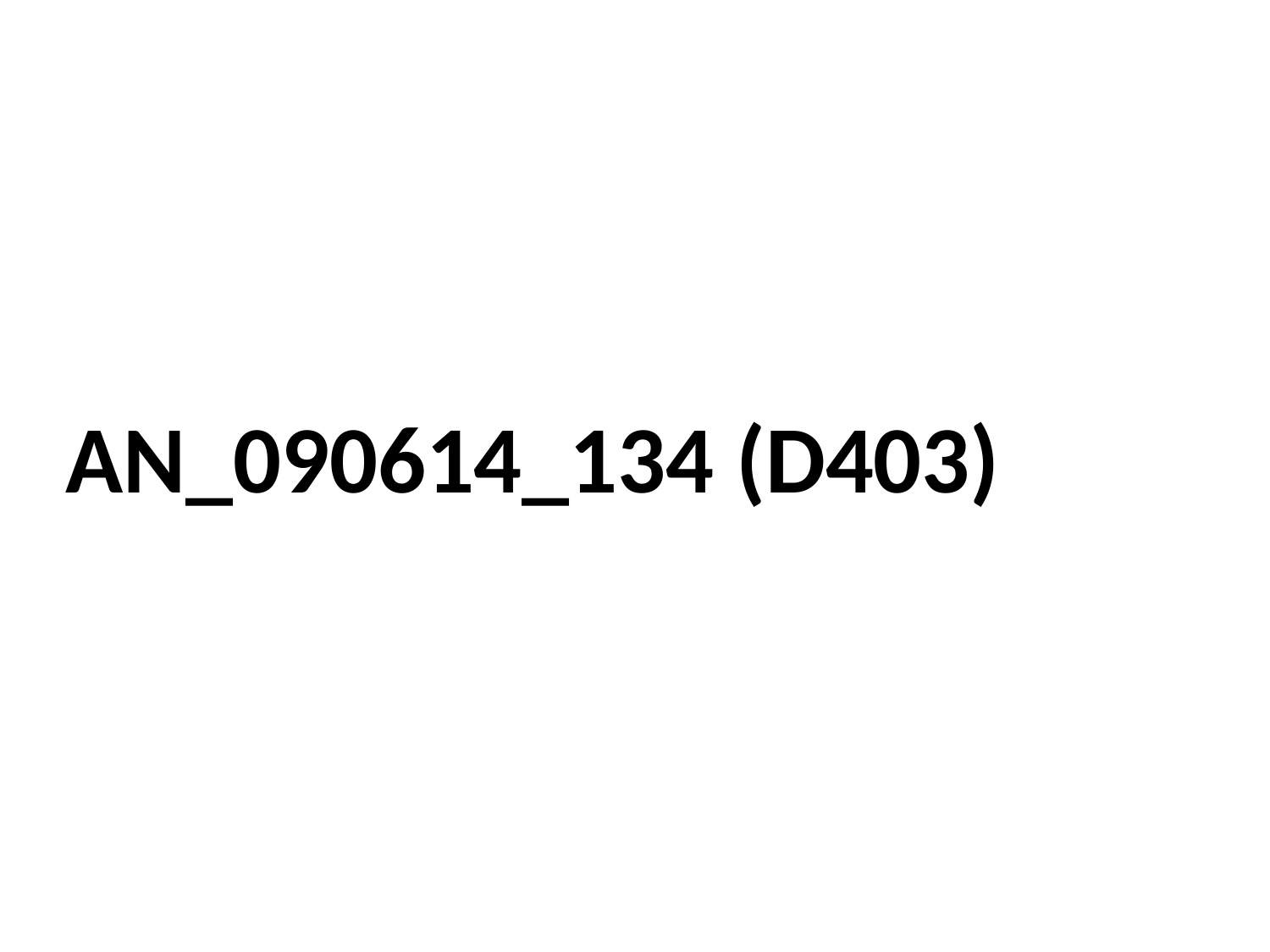

#
AN_090614_134 (D403)

## Slide 90
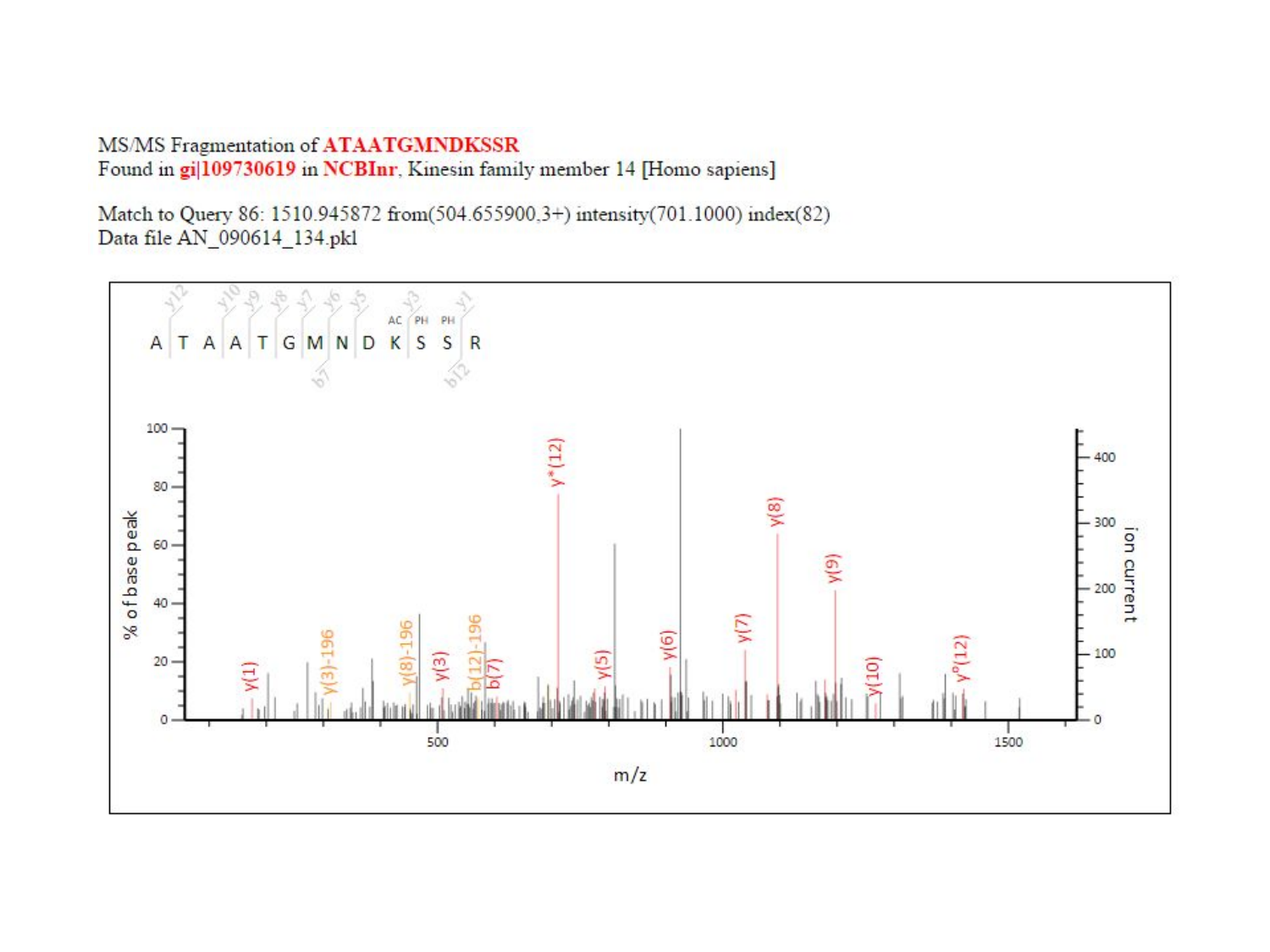

#

## Slide 91
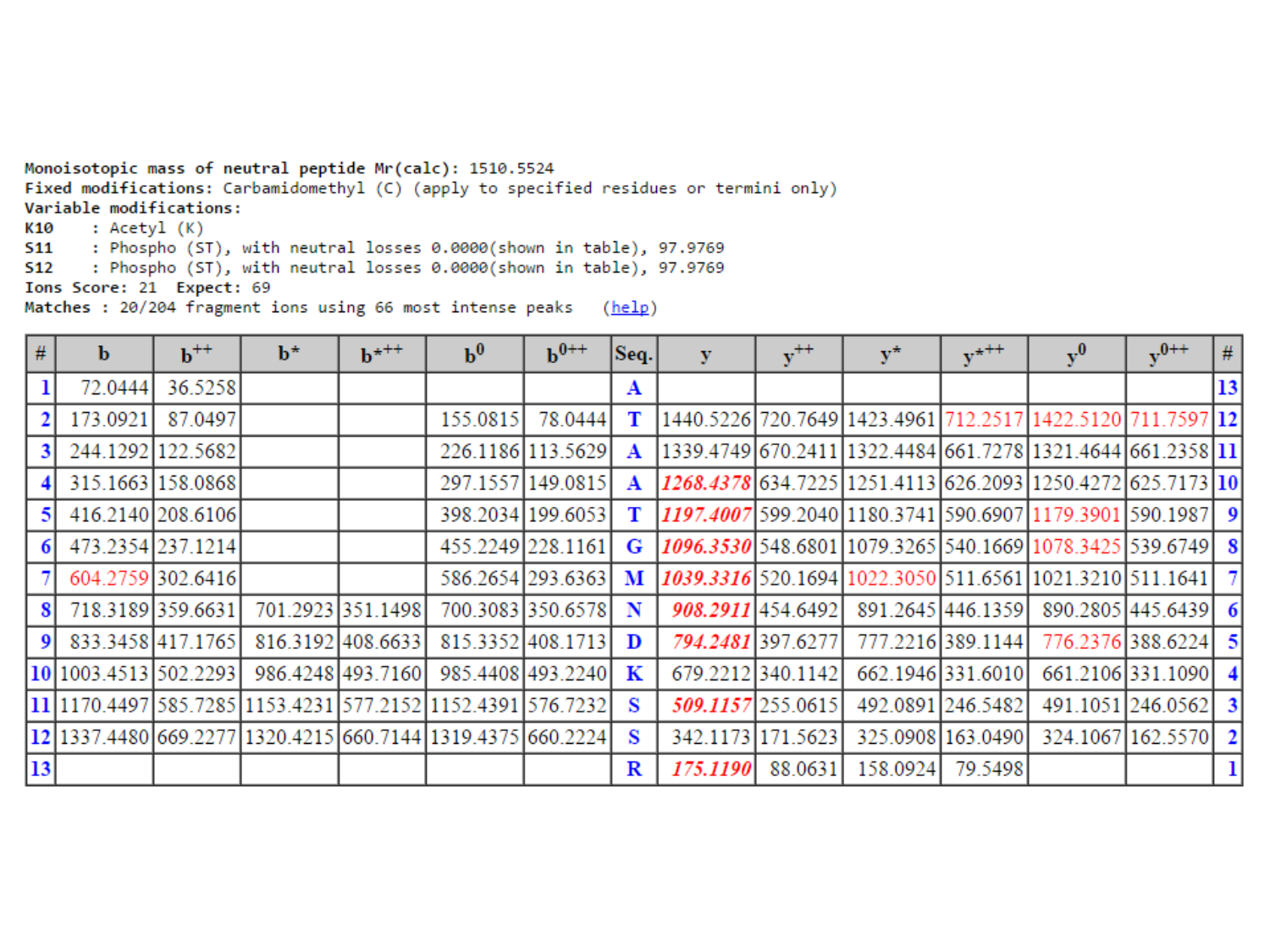

#

## Slide 92
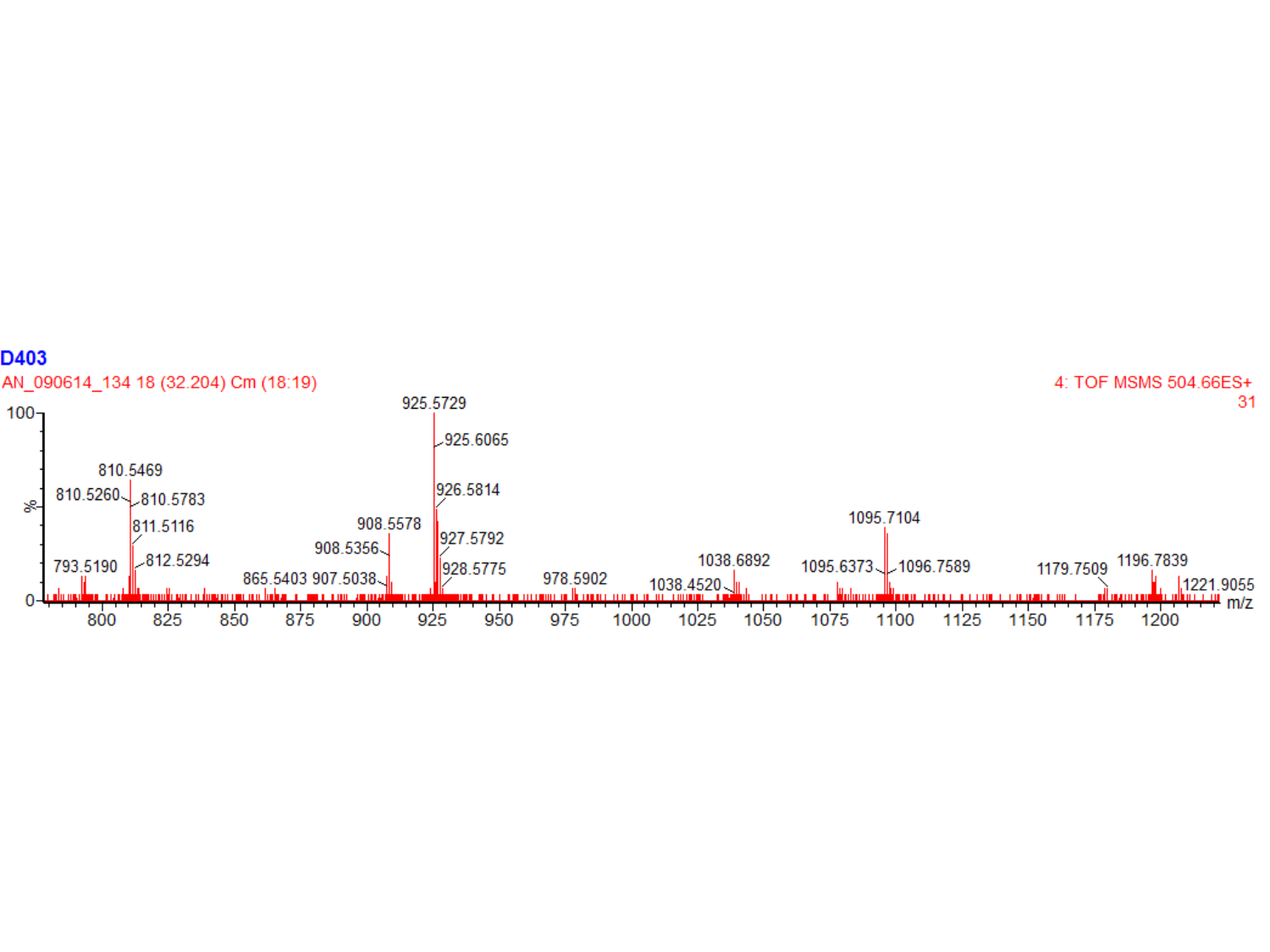

#

## Slide 93
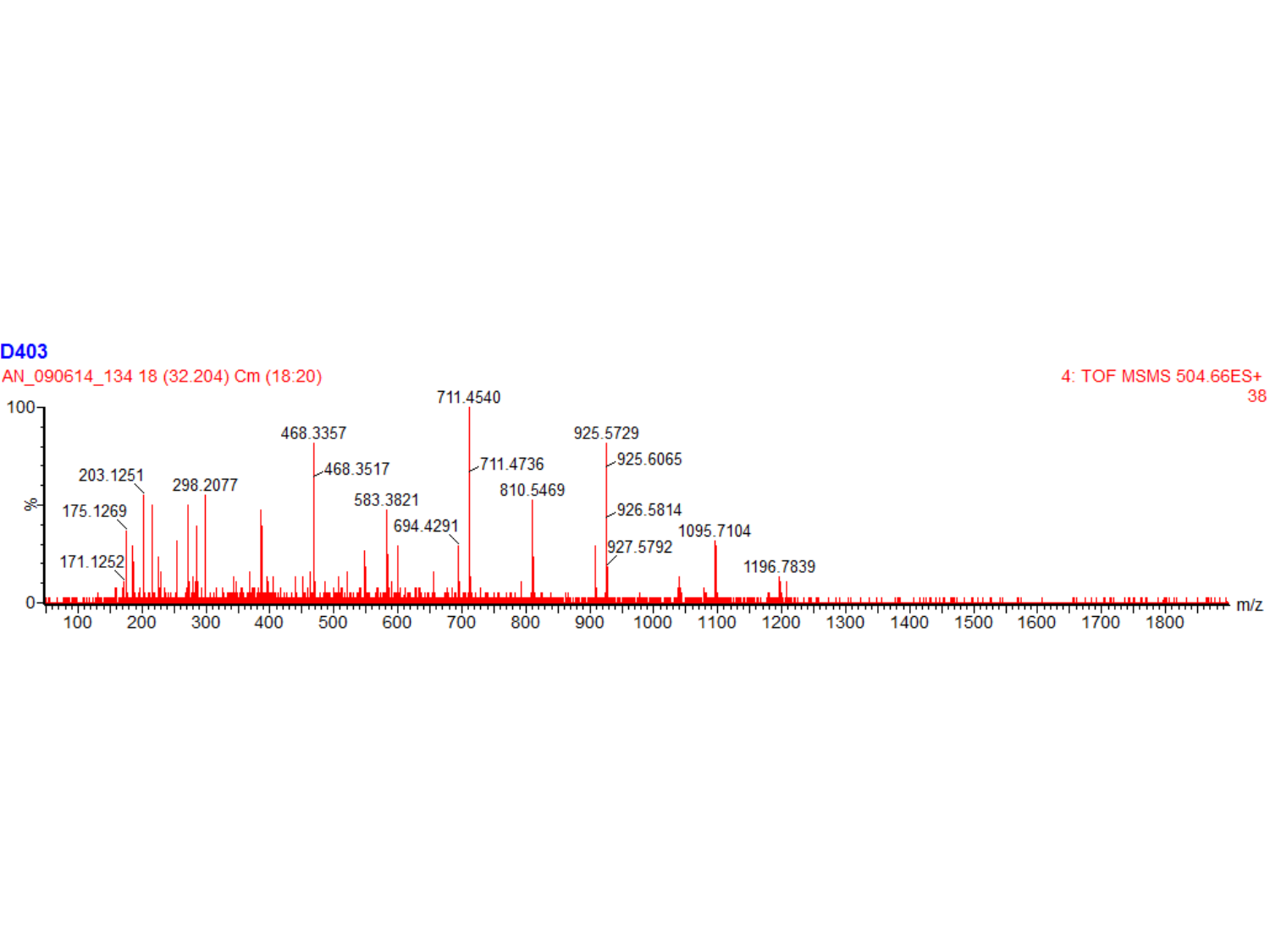

#

## Slide 94
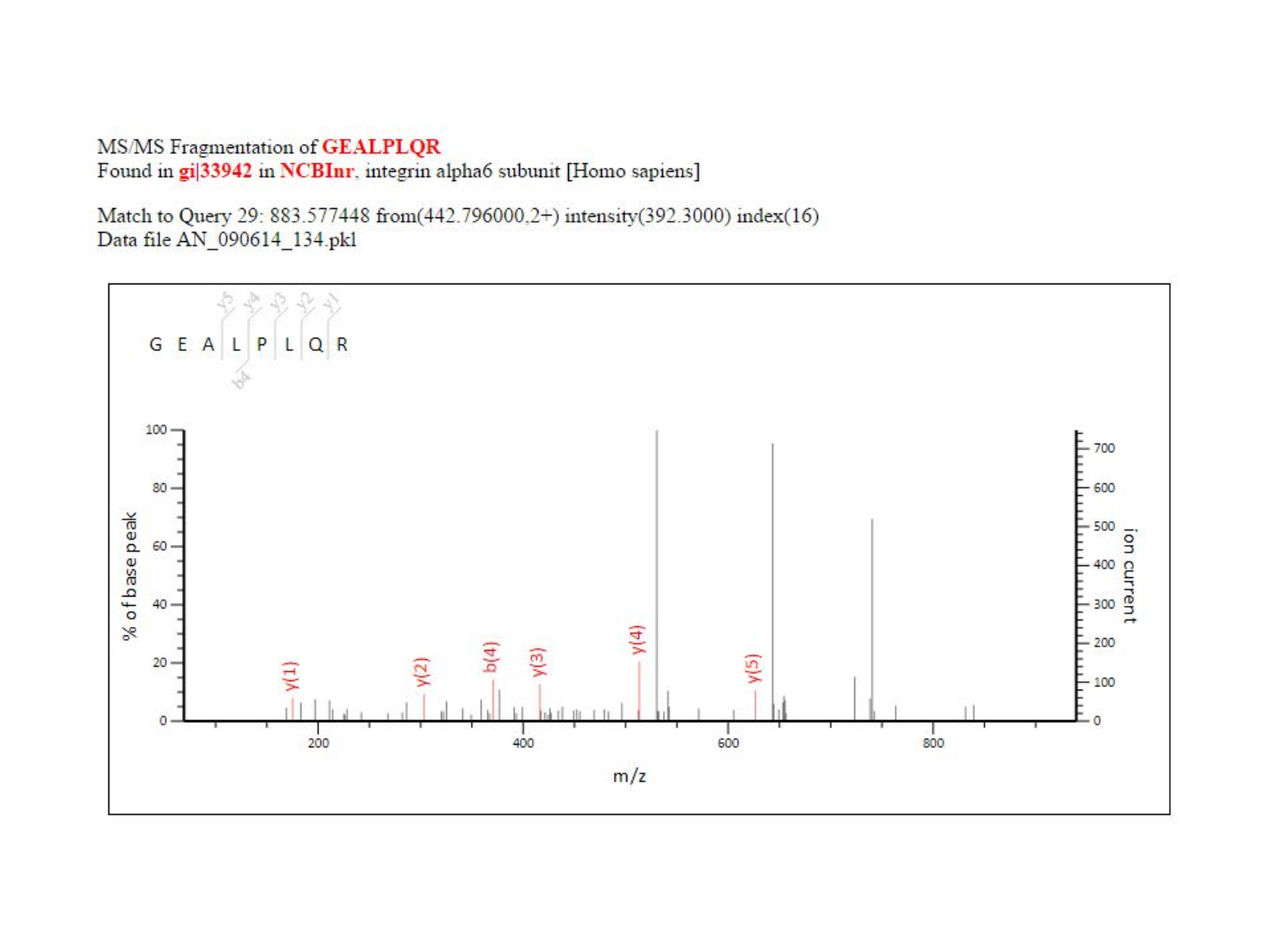

#

## Slide 95
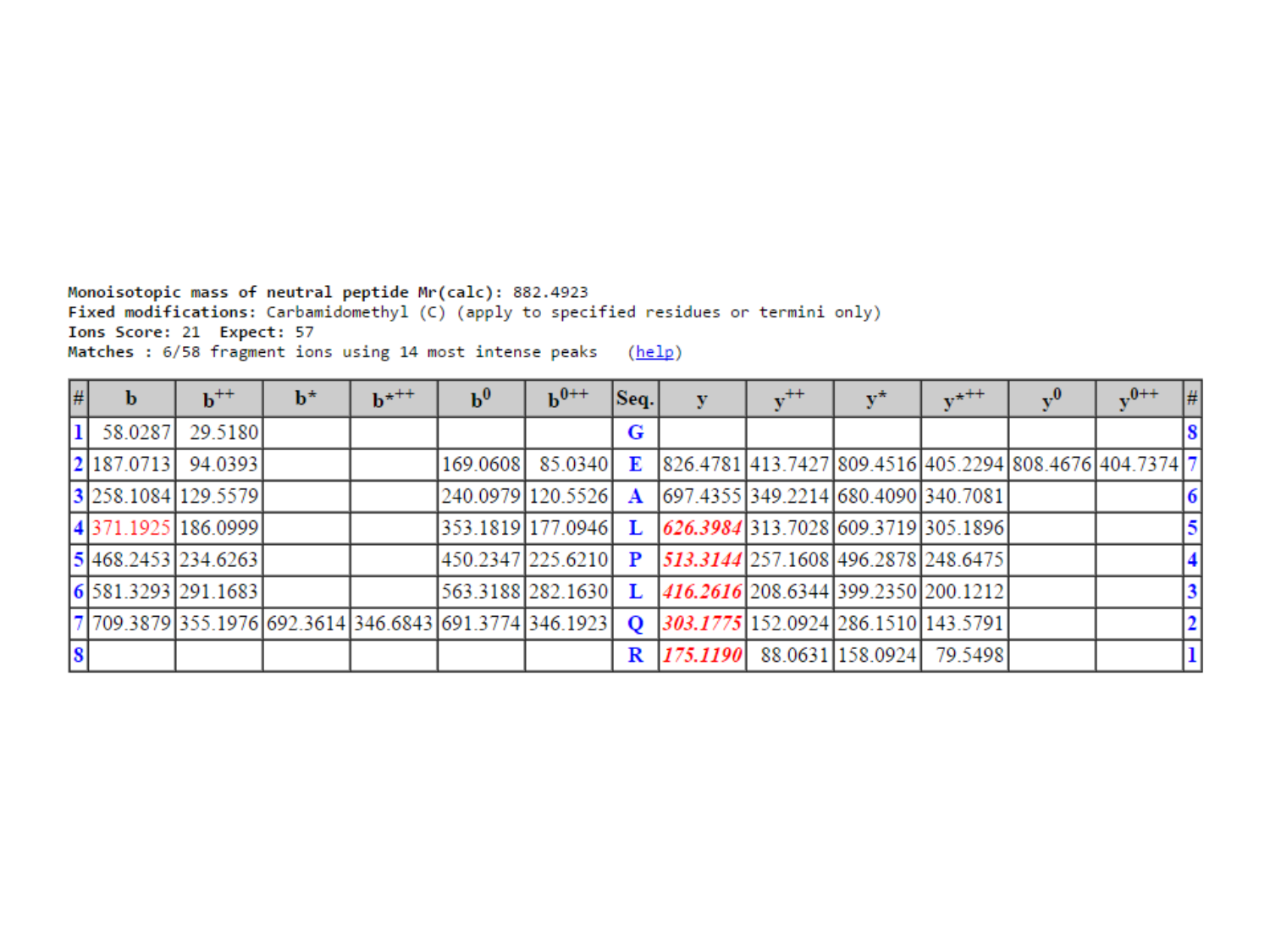

#

## Slide 96
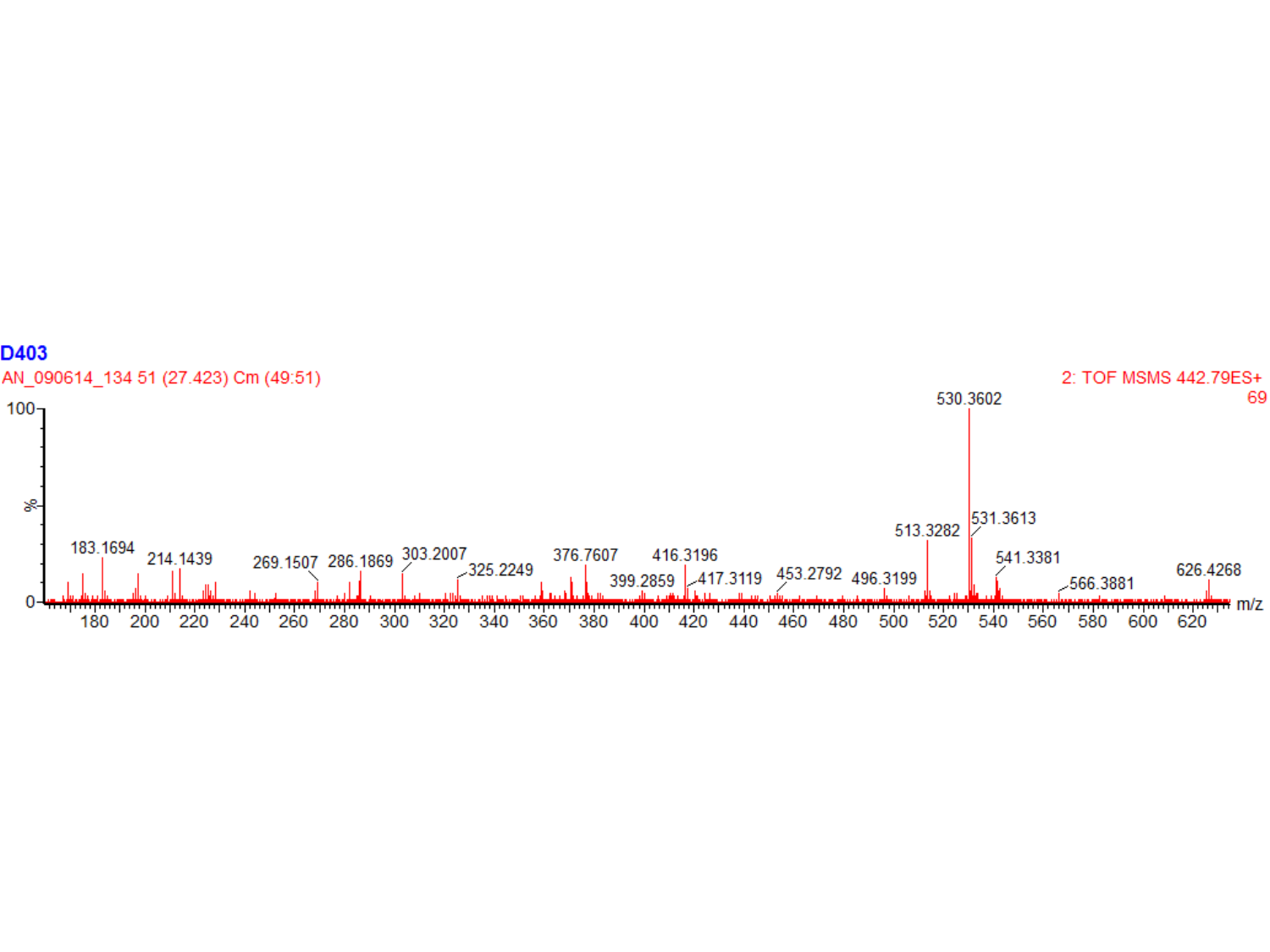

#

## Slide 97
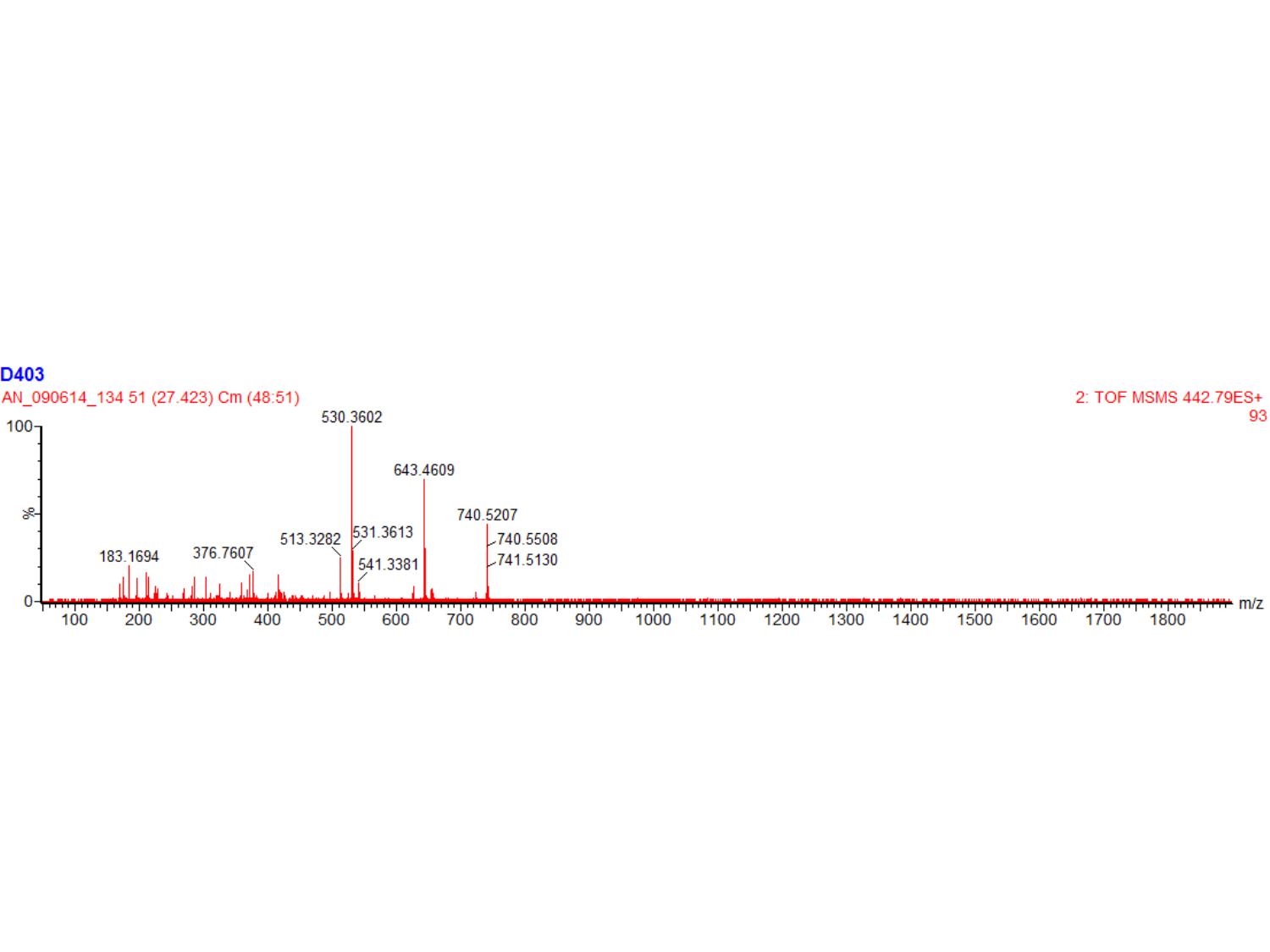

#

## Slide 98
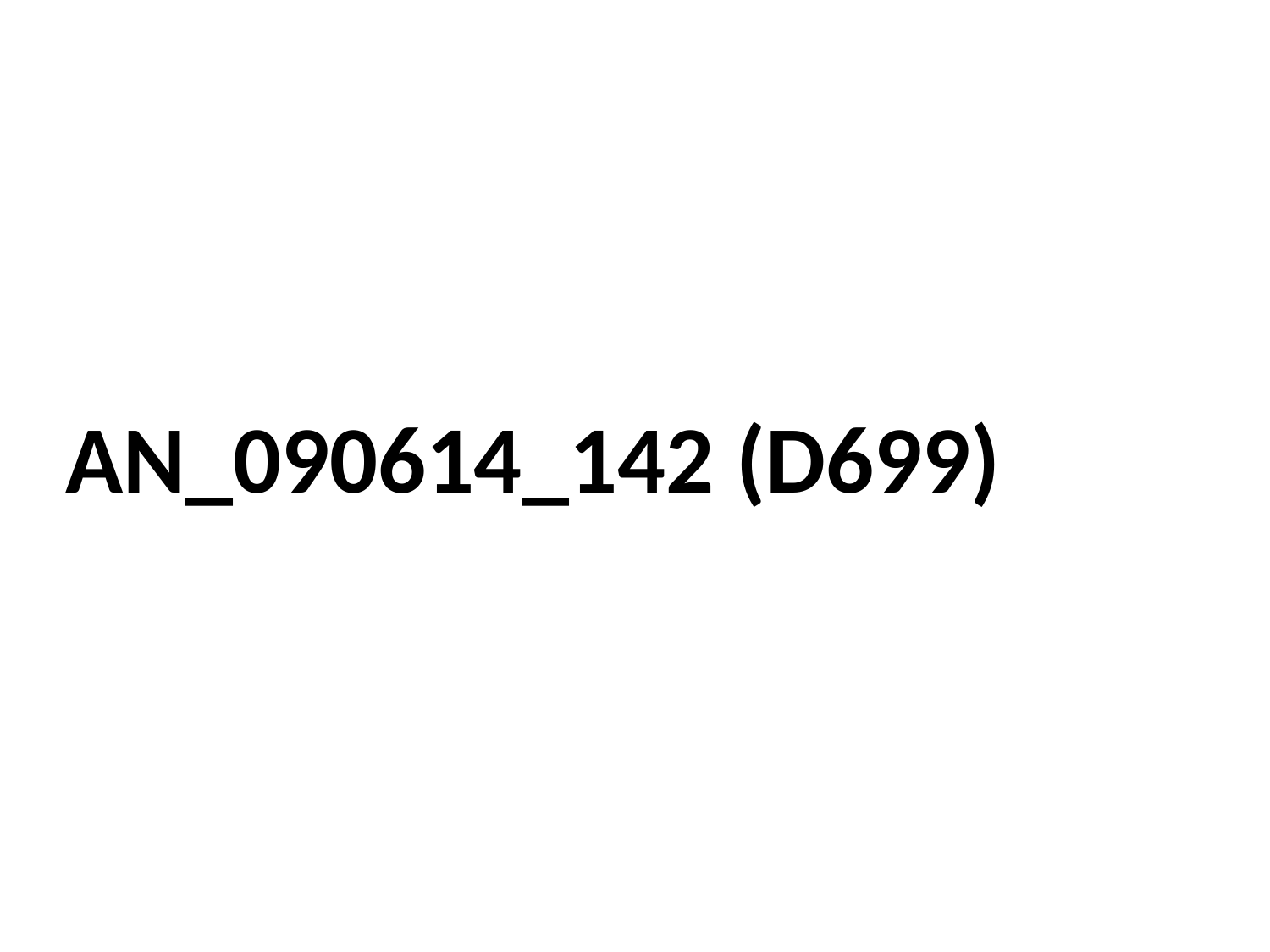

#
AN_090614_142 (D699)

## Slide 99
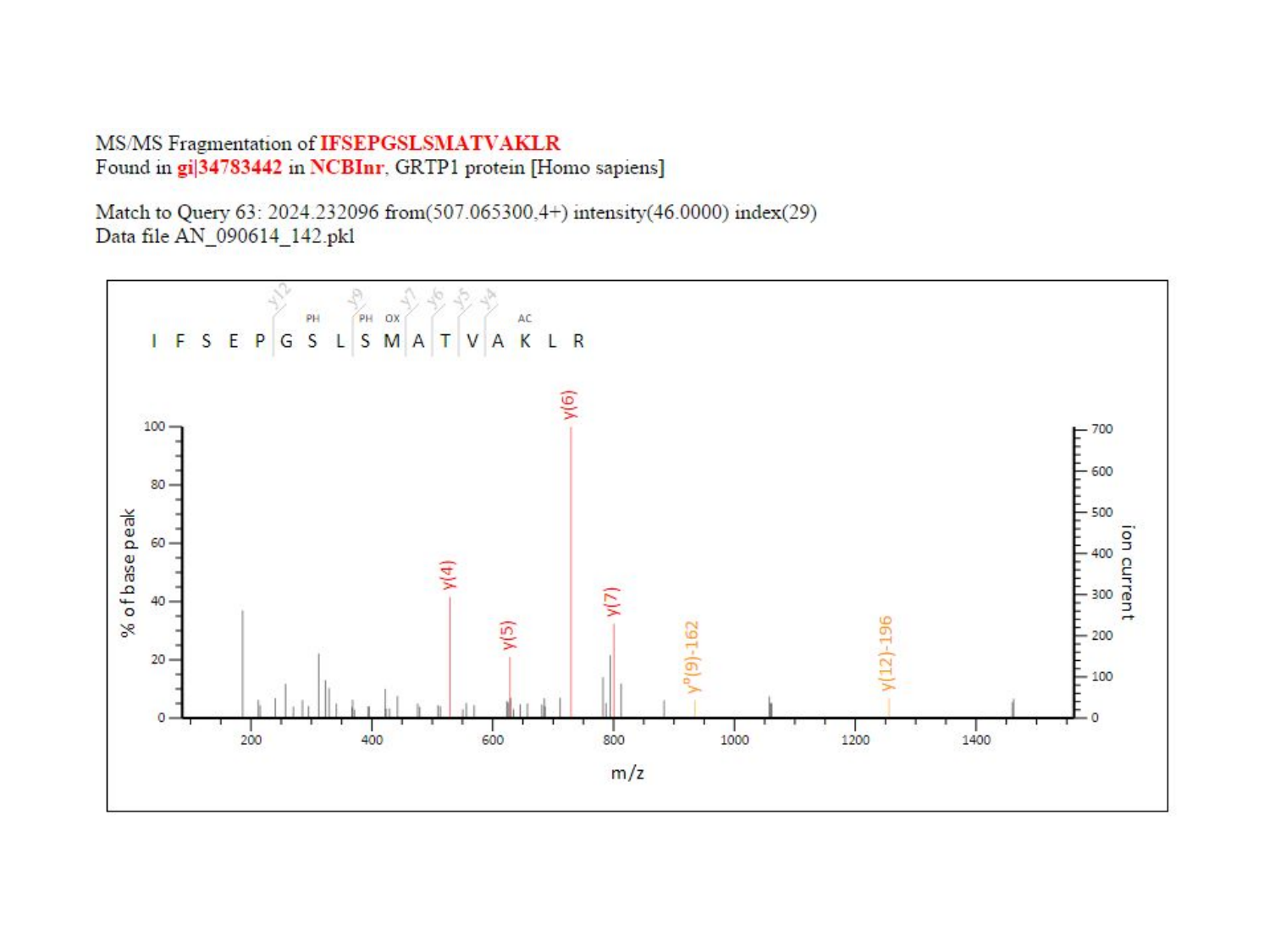

#

## Slide 100
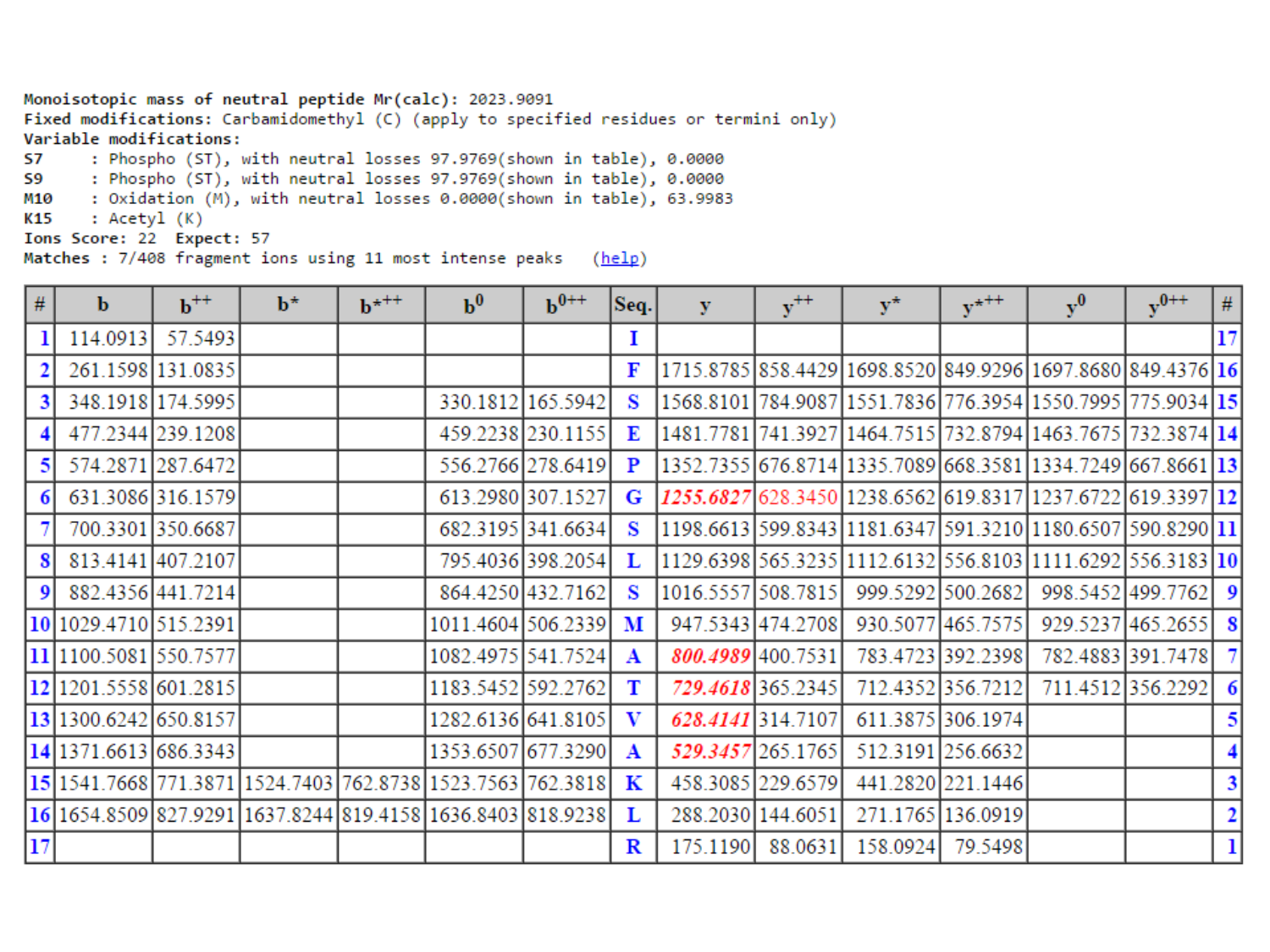

#

## Slide 101
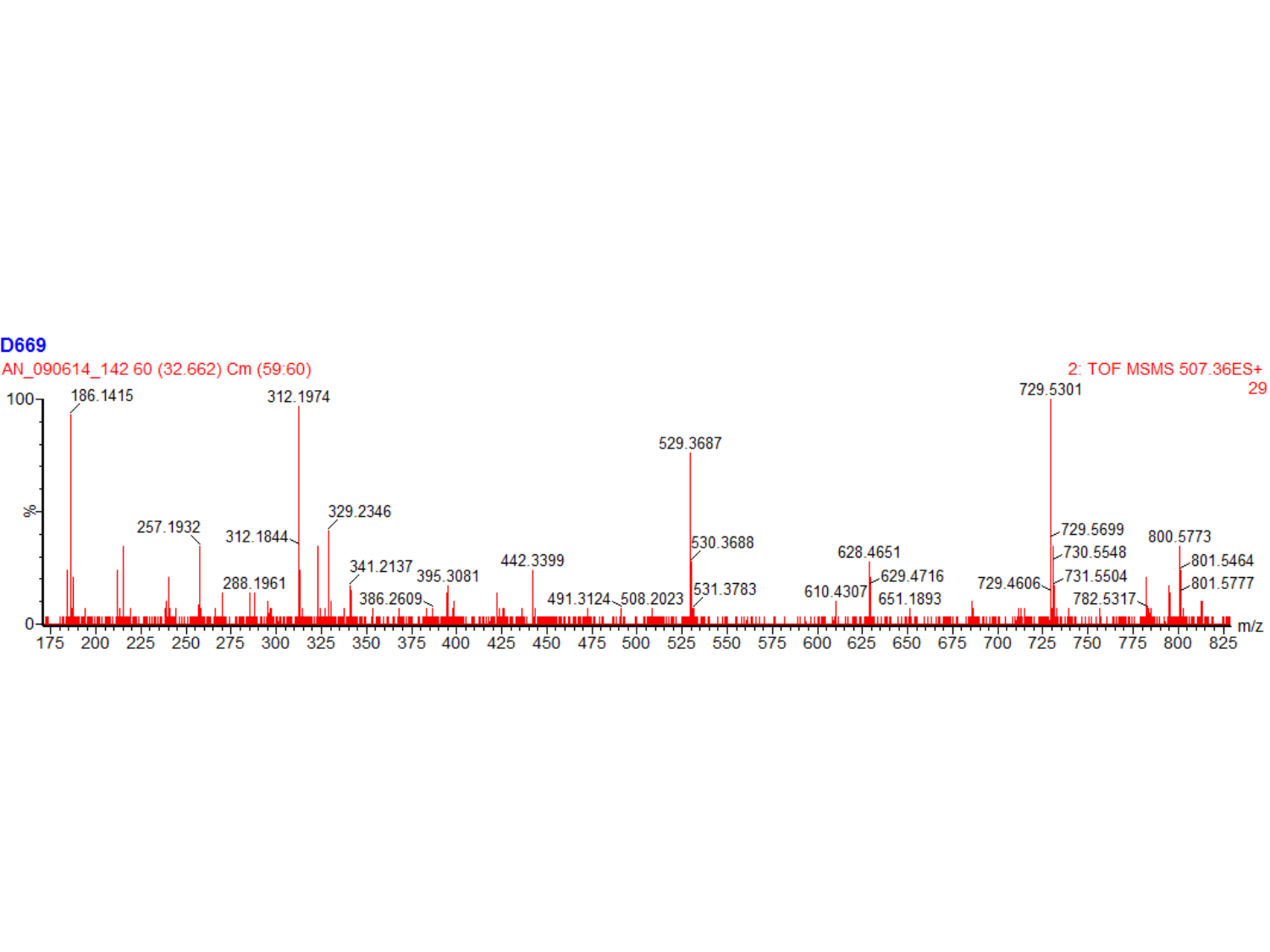

#

## Slide 102
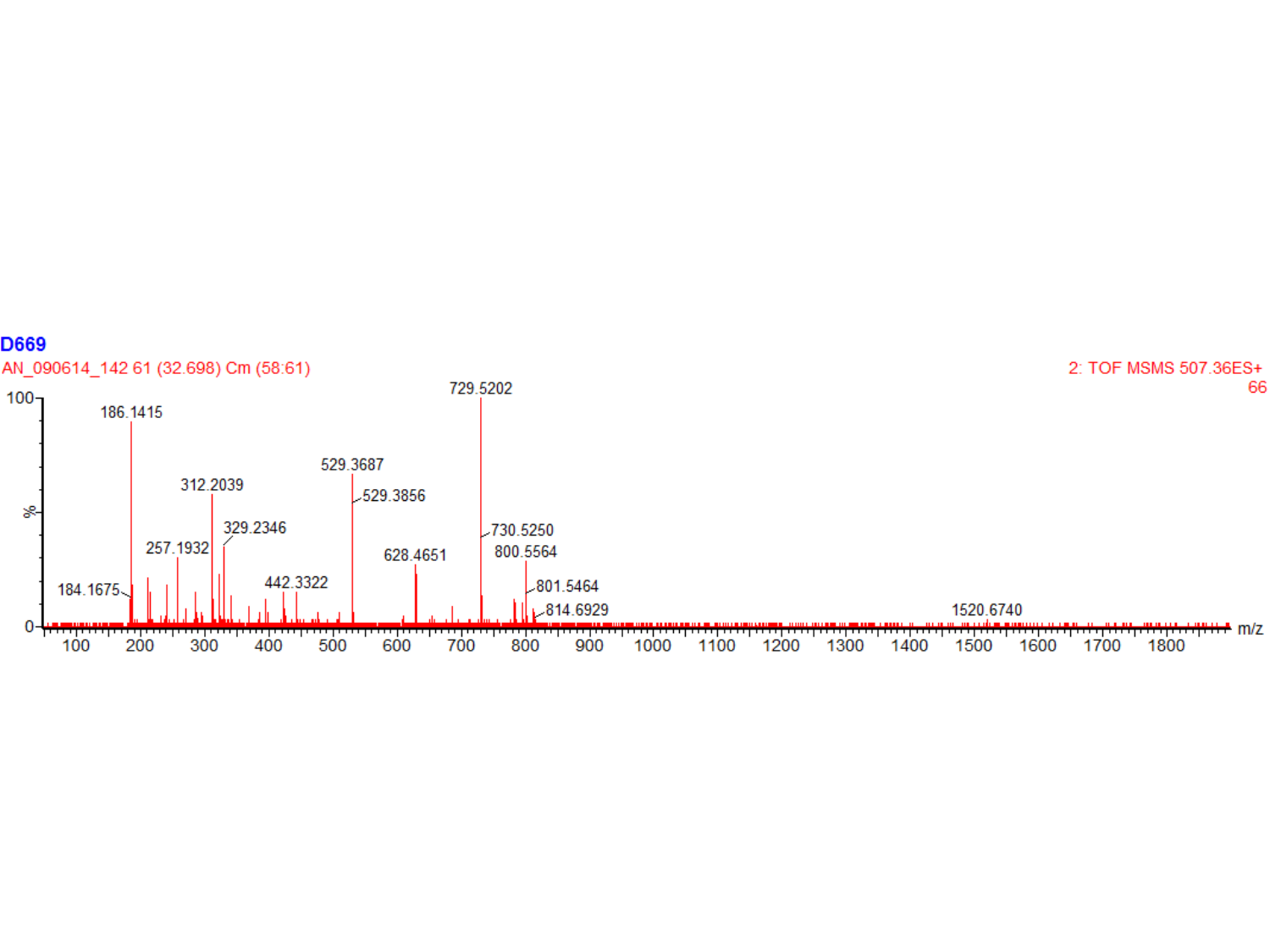

#

## Slide 103
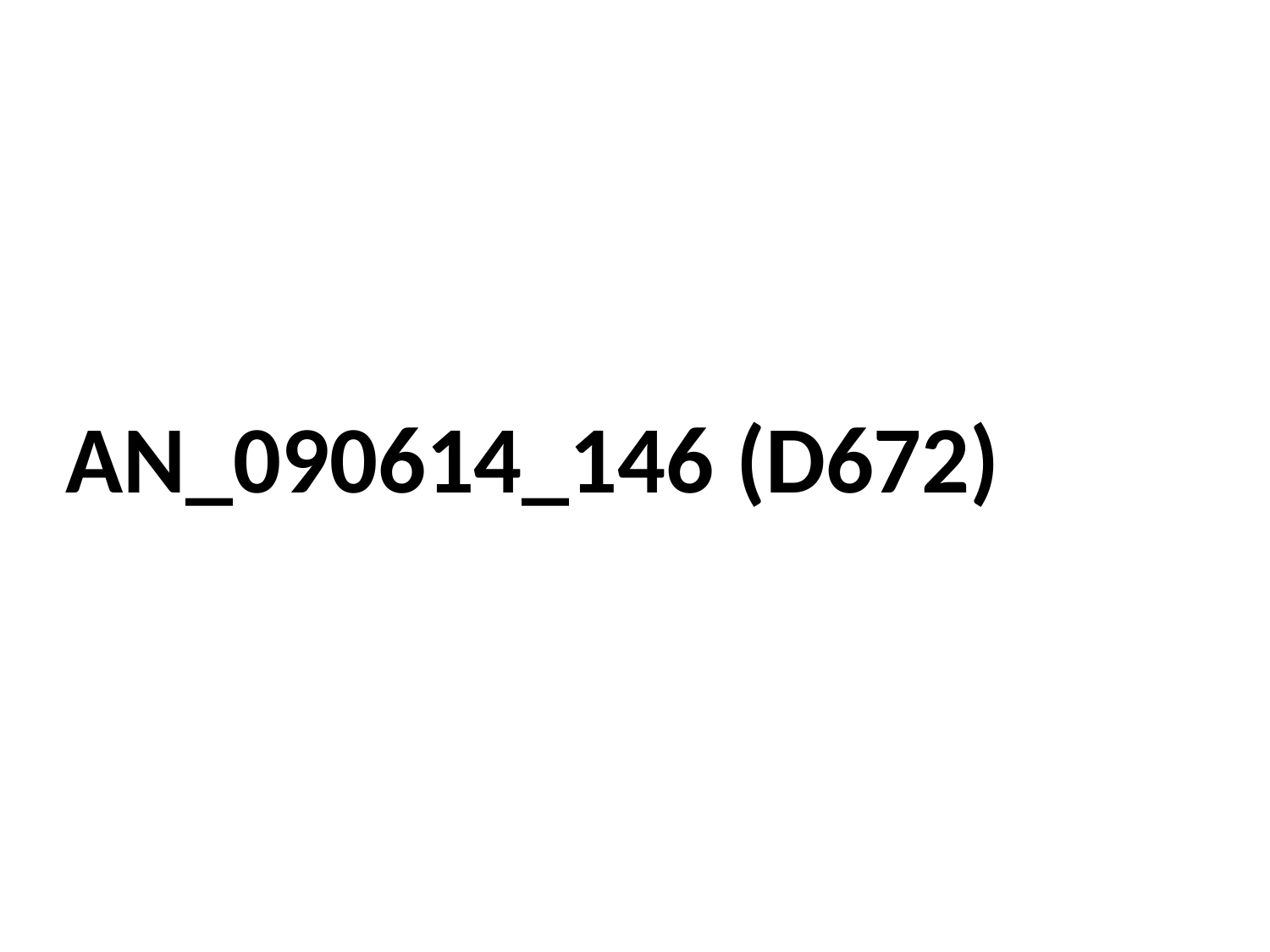

#
AN_090614_146 (D672)

## Slide 104
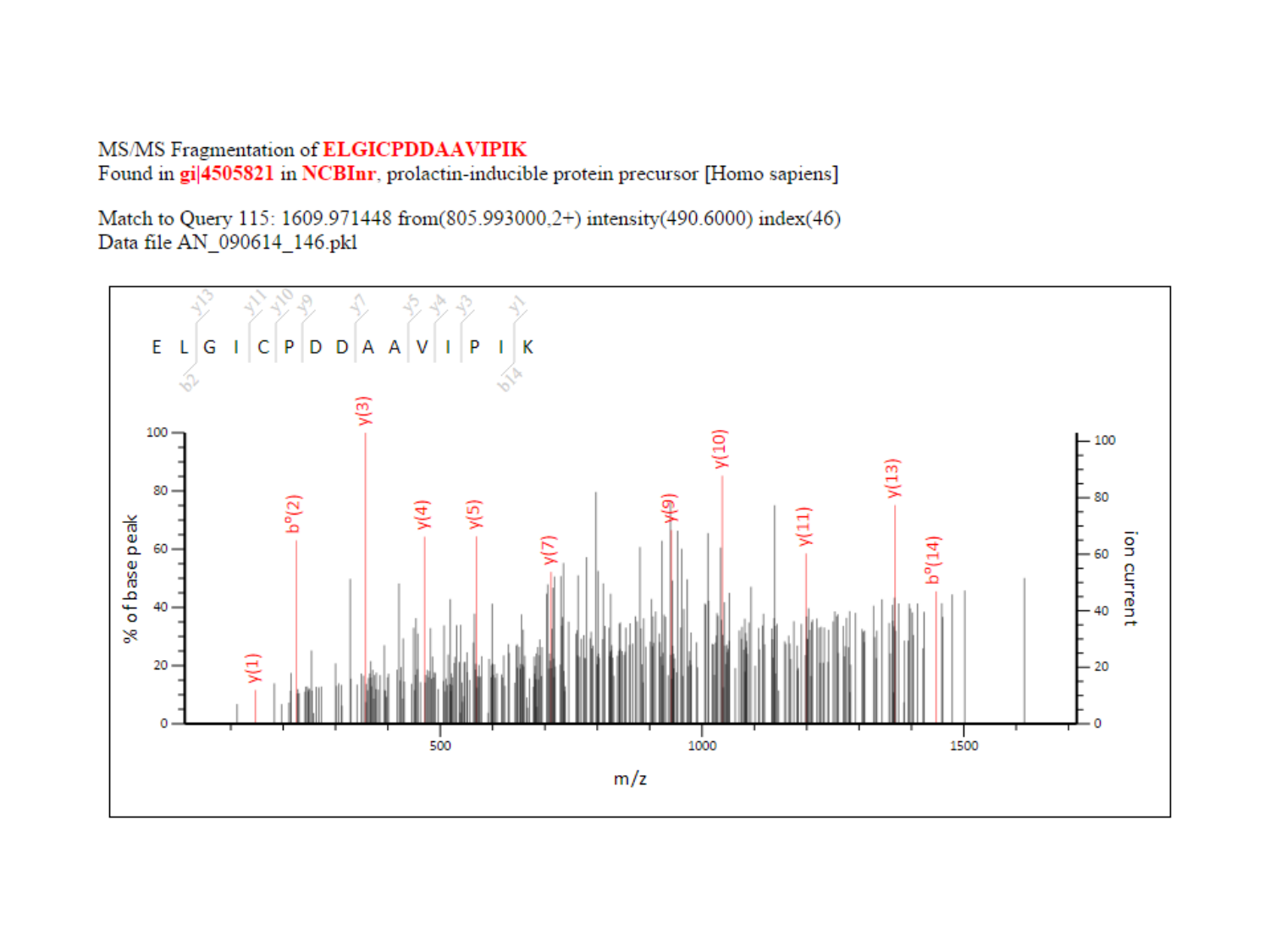

#

## Slide 105
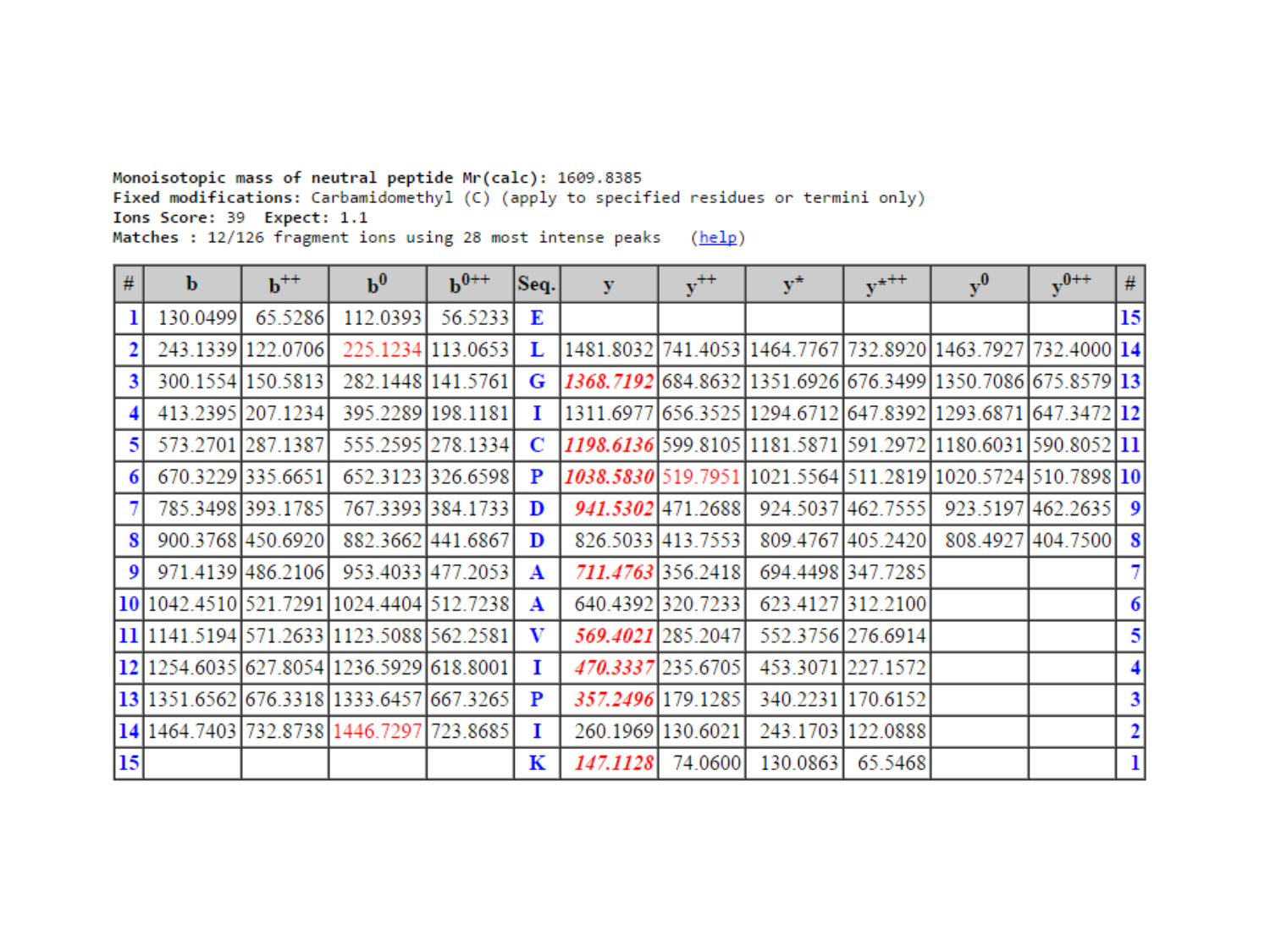

#

## Slide 106
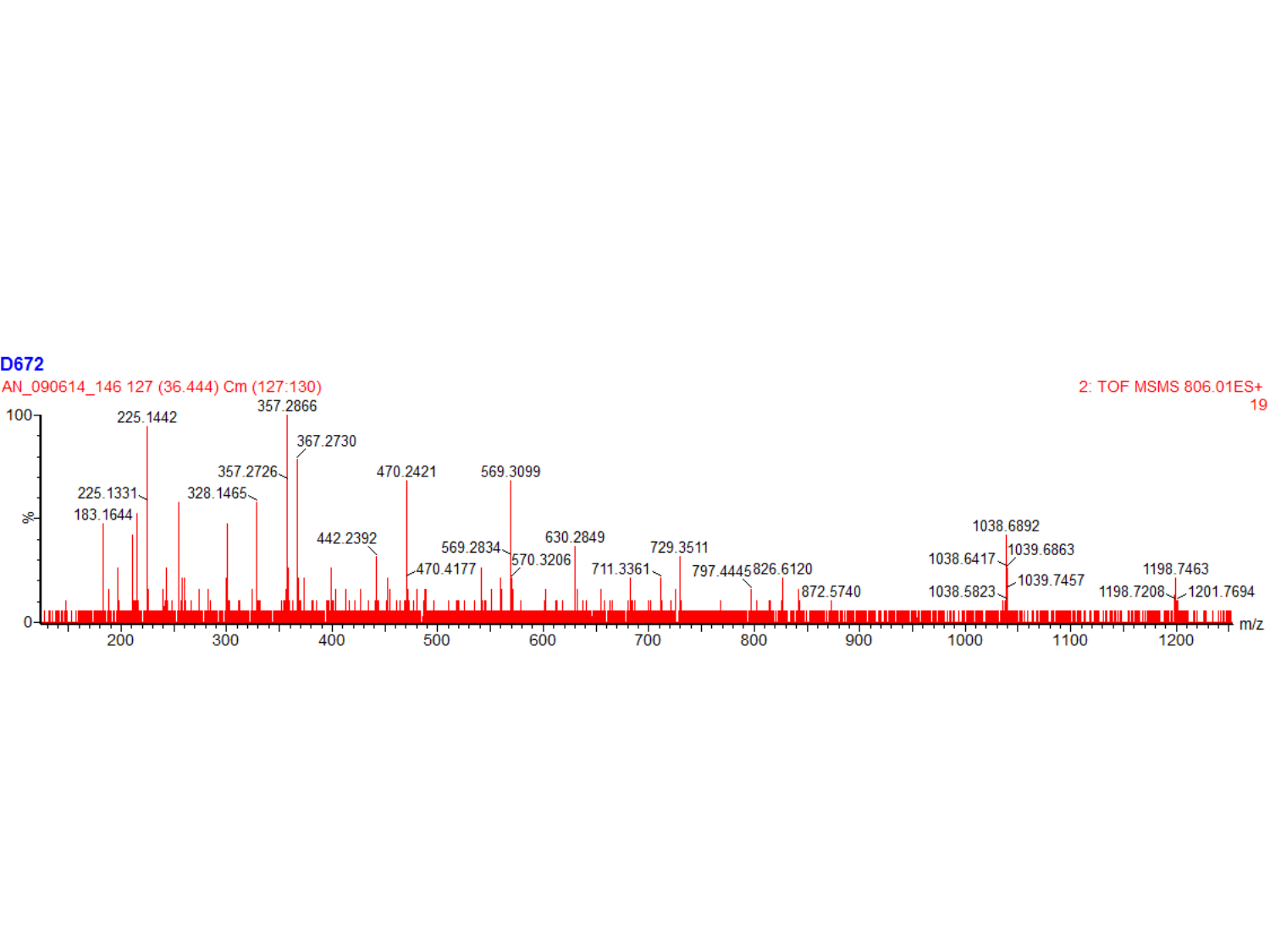

#

## Slide 107
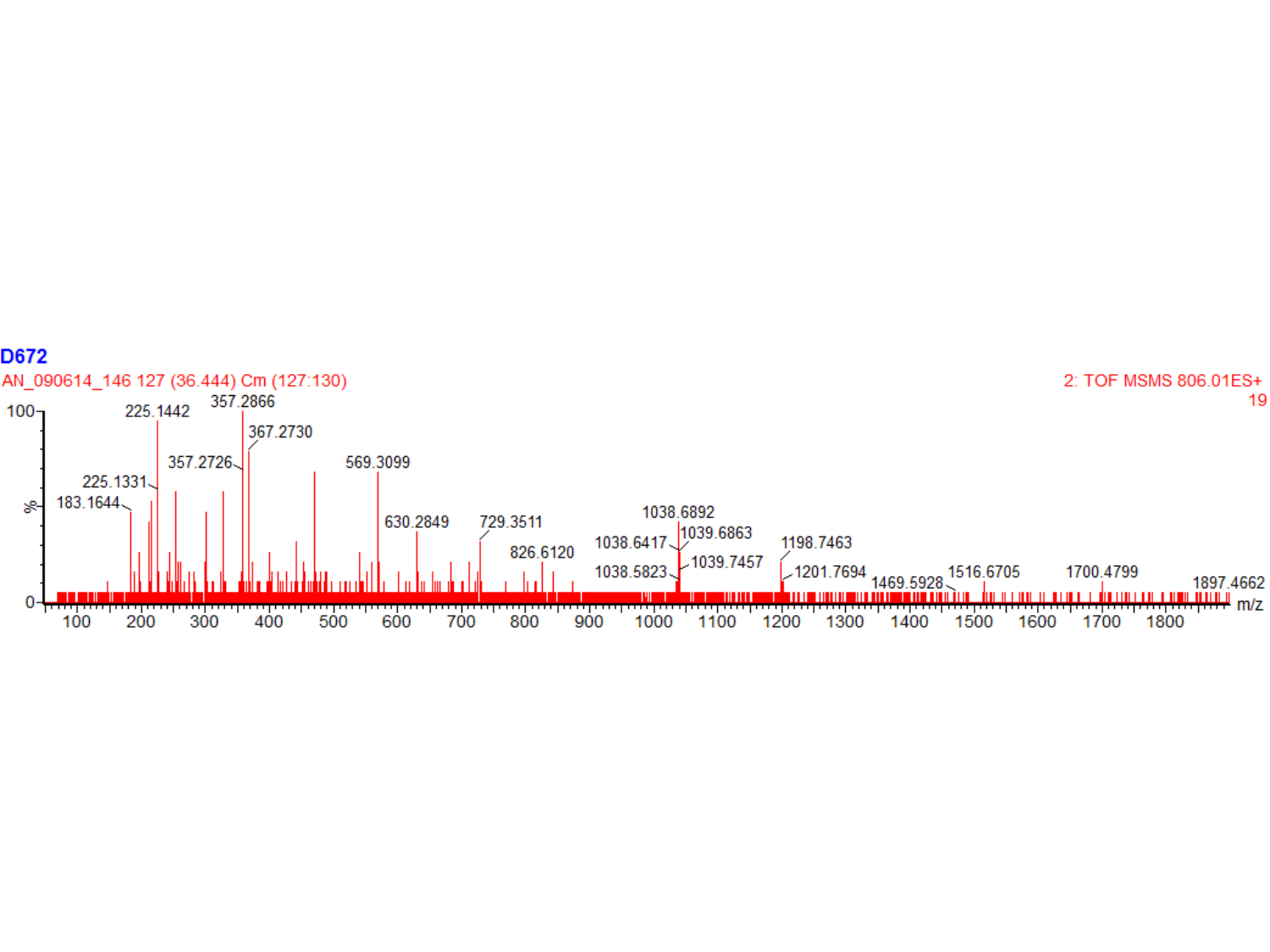

#

## Slide 108
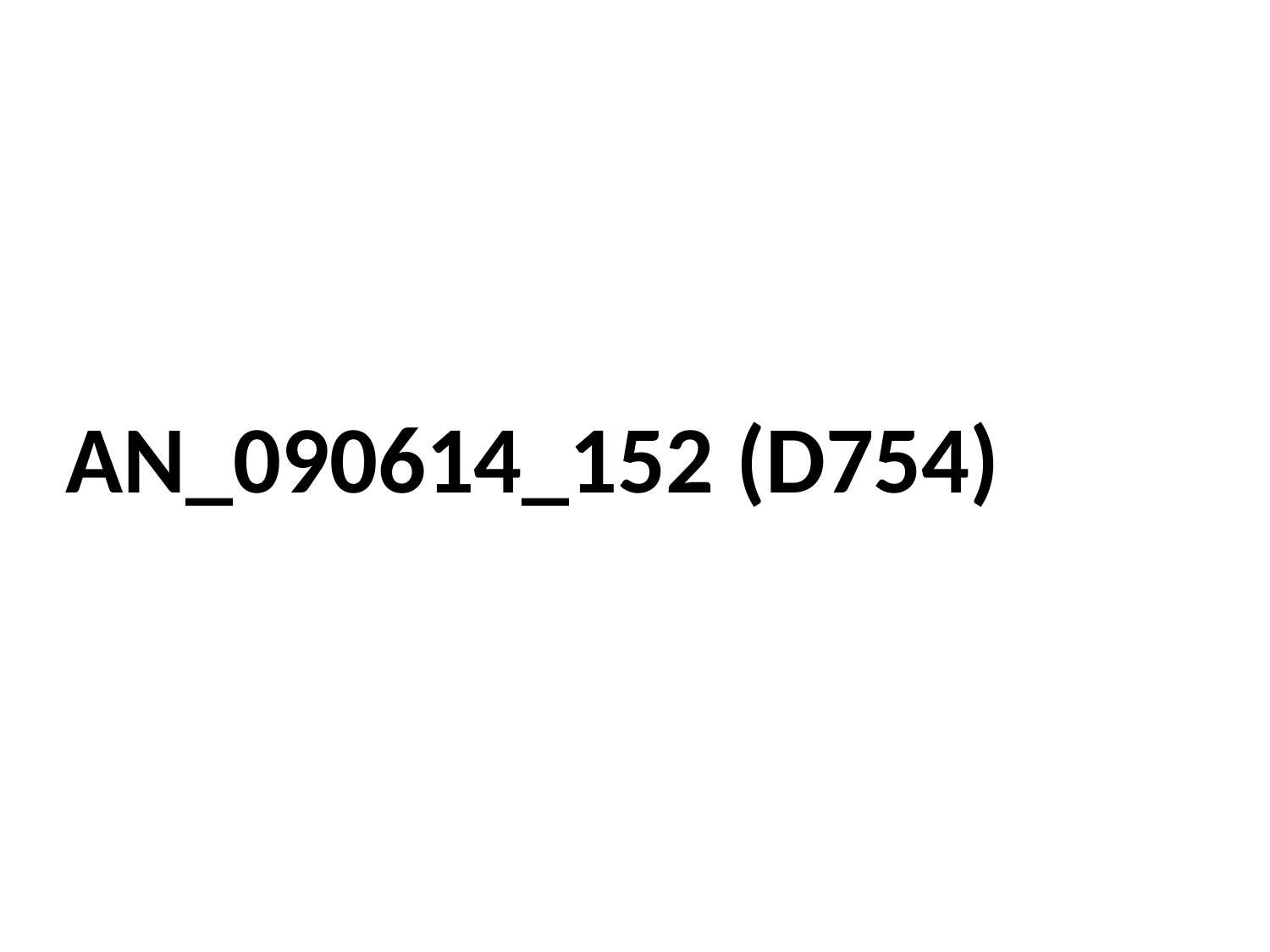

#
AN_090614_152 (D754)

## Slide 109
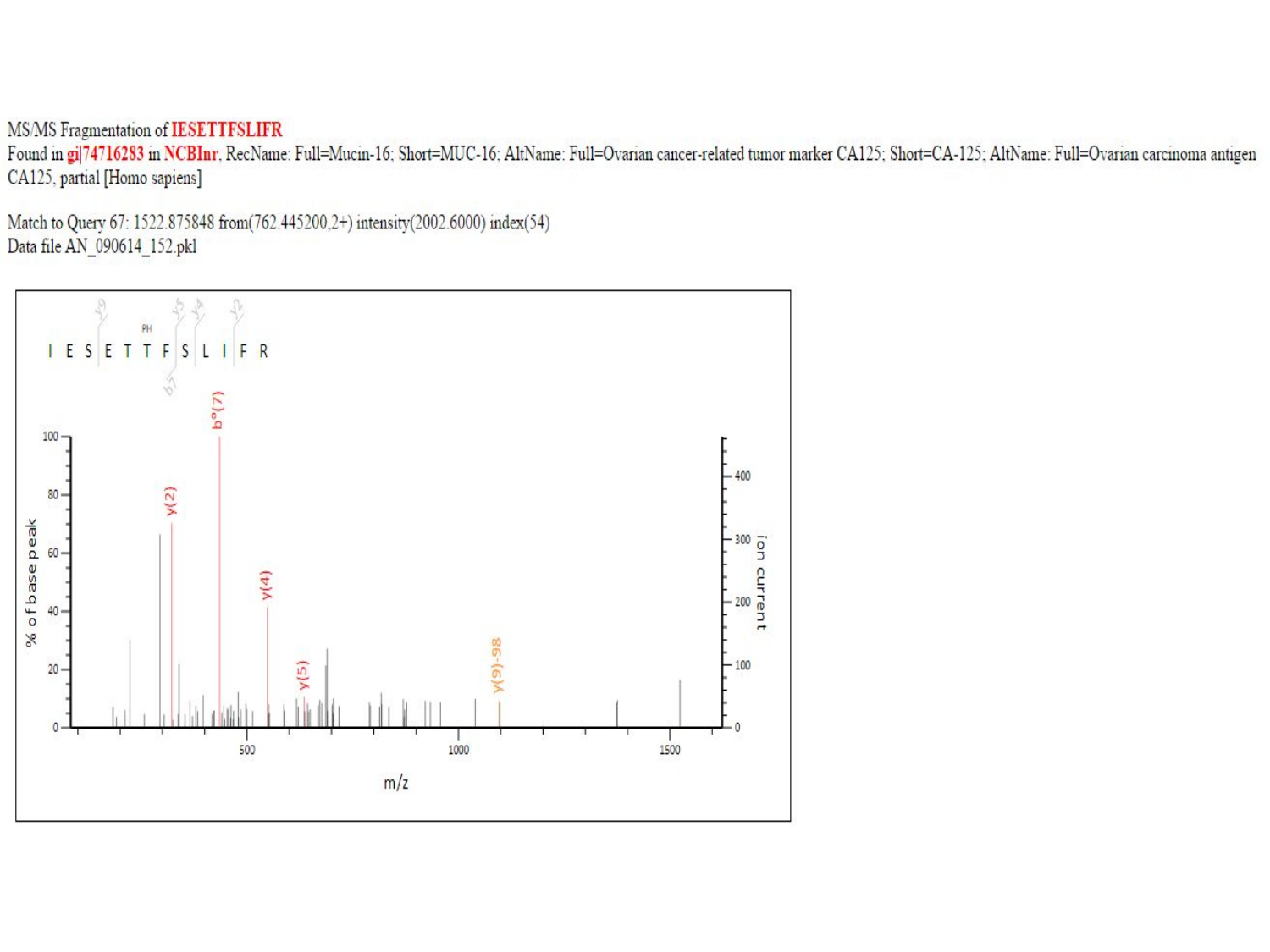

#

## Slide 110
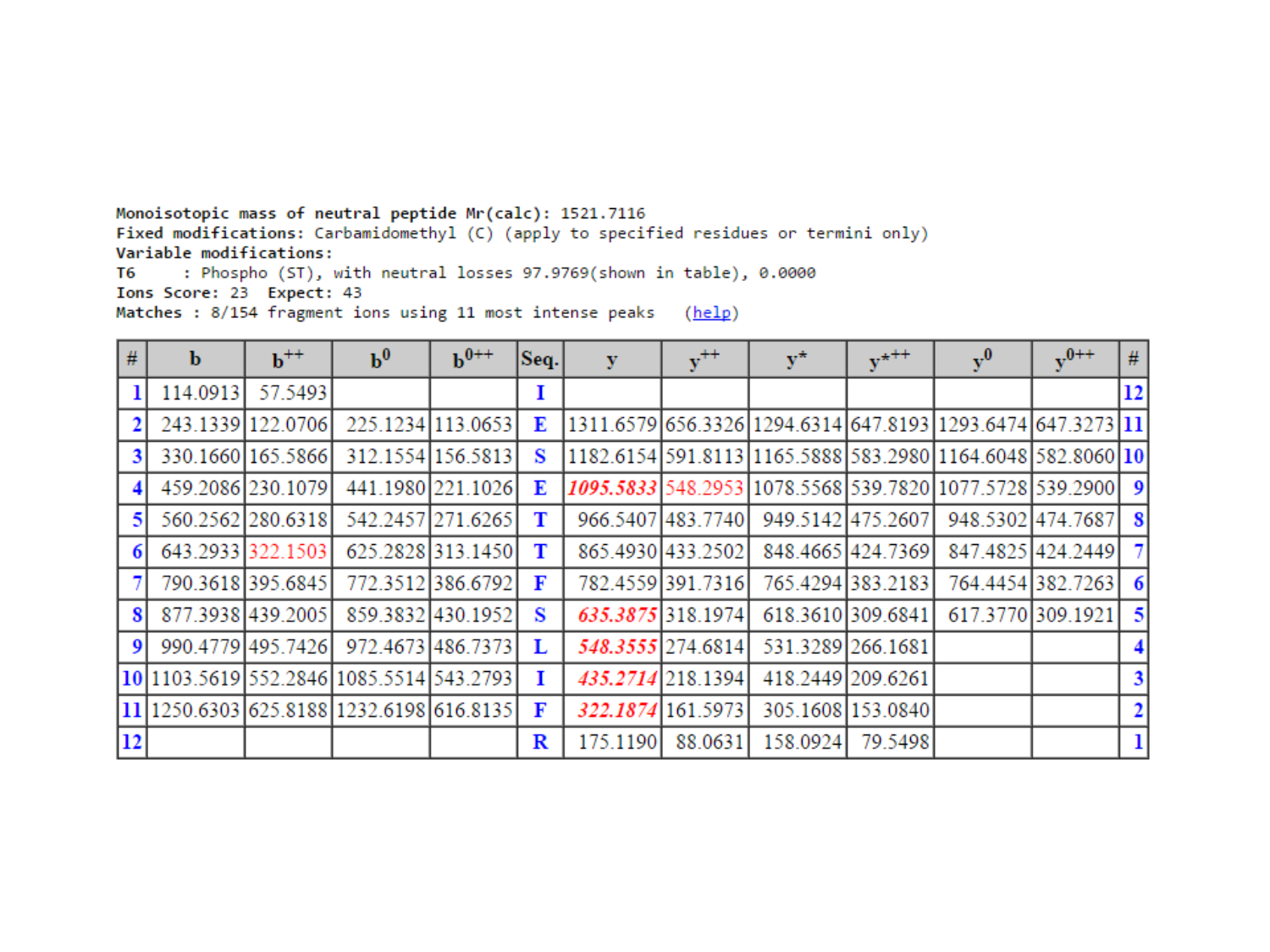

#

## Slide 111
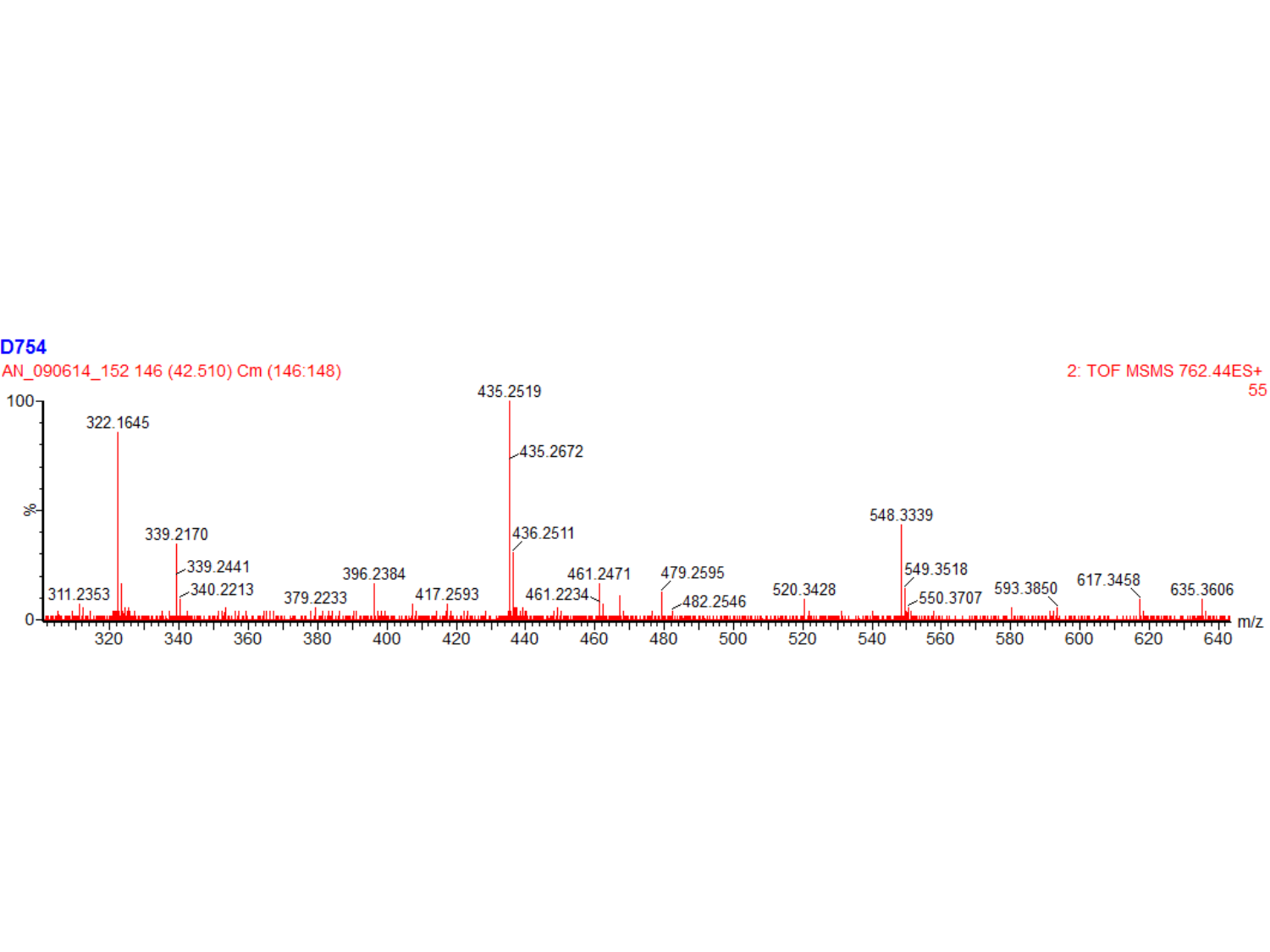

#

## Slide 112
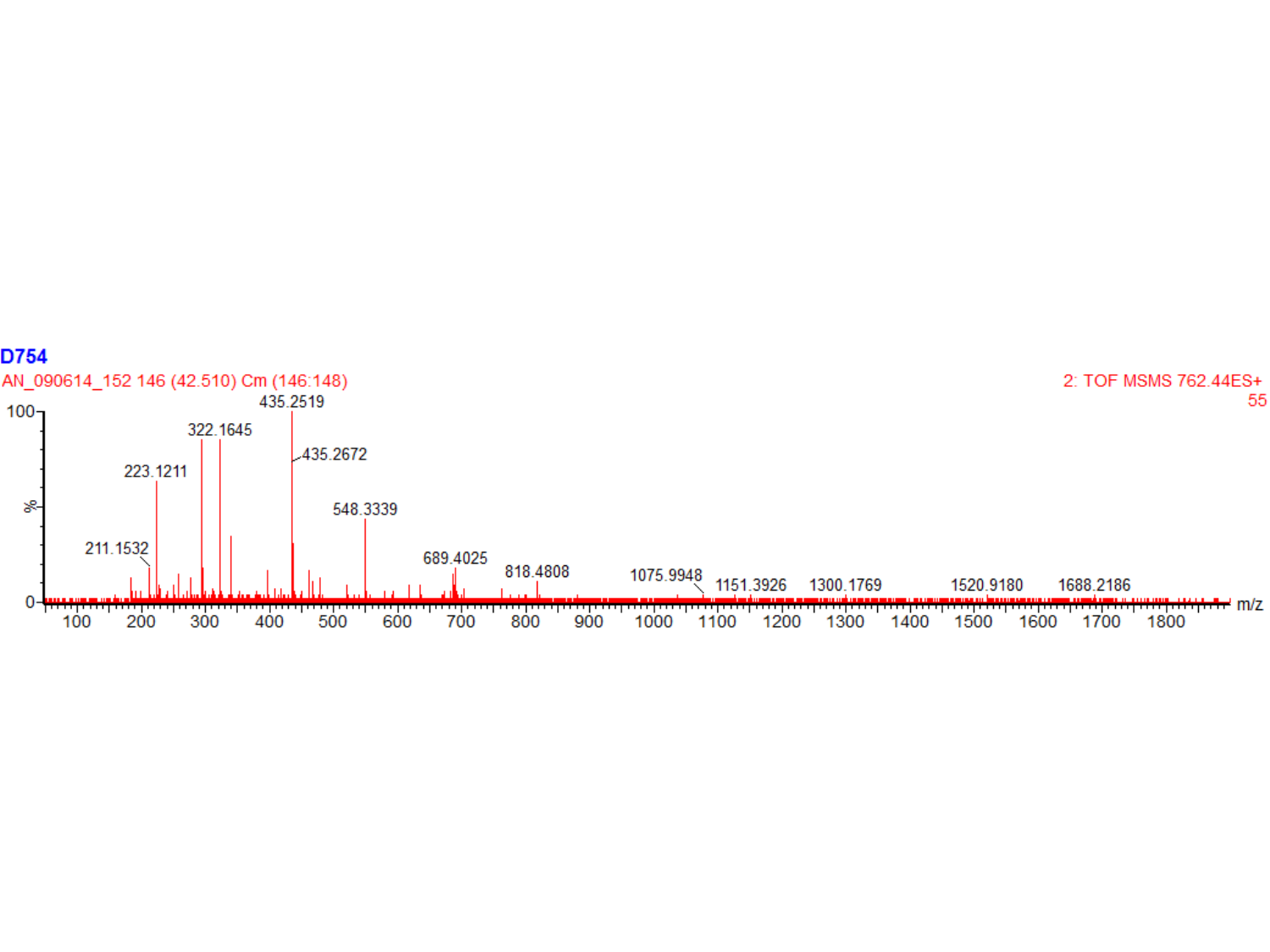

#
